# Electronic structure comparisons of isostructural early d- and f-block metal(iii) bis(cyclopentadienyl) silanide complexes†‡

**DOI:** 10.1039/d2sc04526e

**Published:** 2022-12-06

**Authors:** Gemma K. Gransbury, Benjamin L. L. Réant, Ashley J. Wooles, Jack Emerson-King, Nicholas F. Chilton, Stephen T. Liddle, David P. Mills

**Affiliations:** a Department of Chemistry, The University of Manchester Oxford Road Manchester M13 9PL UK nicholas.chilton@manchester.ac.uk steve.liddle@manchester.ac.uk david.mills@manchester.ac.uk

## Abstract

We report the synthesis of the U(iii) bis(cyclopentadienyl) hypersilanide complex [U(Cp′′)_2_{Si(SiMe_3_)_3_}] (Cp′′ = {C_5_H_3_(SiMe_3_)_2_-1,3}), together with isostructural lanthanide and group 4 M(iii) homologues, in order to meaningfully compare metal-silicon bonding between early d- and f-block metals. All complexes were characterised by a combination of NMR, EPR, UV-vis-NIR and ATR-IR spectroscopies, single crystal X-ray diffraction, SQUID magnetometry, elemental analysis and *ab initio* calculations. We find that for the [M(Cp′′)_2_{Si(SiMe_3_)_3_}] (M = Ti, Zr, La, Ce, Nd, U) series the unique anisotropy axis is conserved tangential to 
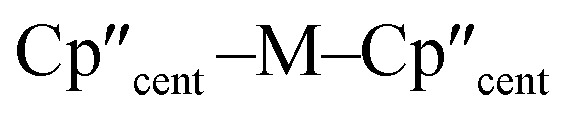
; this is governed by the hypersilanide ligand for the d-block complexes to give easy plane anisotropy, whereas the easy axis is fixed by the two Cp′′ ligands in f-block congeners. This divergence is attributed to hypersilanide acting as a strong σ-donor and weak π-acceptor with the d-block metals, whilst f-block metals show predominantly electrostatic bonding with weaker π-components. We make qualitative comparisons on the strength of covalency to derive the ordering Zr > Ti ≫ U > Nd ≈ Ce ≈ La in these complexes, using a combination of analytical techniques. The greater covalency of 5f^3^ U(iii) *vs.* 4f^3^ Nd(iii) is found by comparison of their EPR and electronic absorption spectra and magnetic measurements, with calculations indicating that uranium 5f orbitals have weak π-bonding interactions with both the silanide and Cp′′ ligands, in addition to weak δ-antibonding with Cp′′.

## Abstract 2

We report an isostructural series of early metal complexes containing cyclopentadienyl (Cp)-supported M–Si bonds. Differences in the extent of metal–ligand covalency causes a magnetic anisotropy switch between d- and f-block metals.

## Graphical abstract

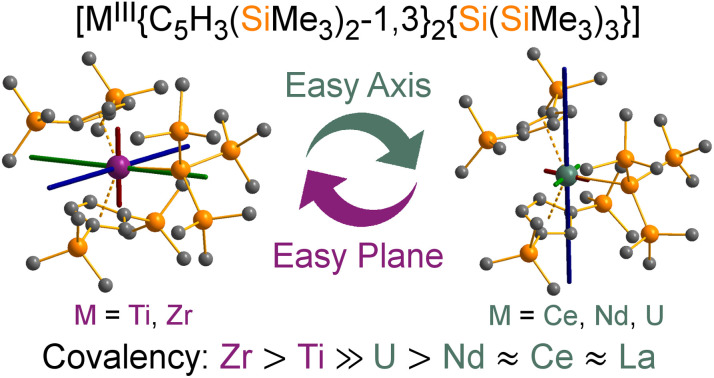

## Introduction

Transition metal (TM) silicon chemistry is well-established, with technological applications being actively developed^1^ for solid-state silicide materials used in microelectronics, ceramics and catalysis,^2^ and molecular silanide complexes that effect (hydro)silylation of unsaturated substrates.^3–5^ In comparison, f-block silicon chemistry is less well developed but shows promise,^6^ with lanthanide (Ln) silicides used to fortify low-alloy steels,^7^ Ln silanide catalysts employed in unsaturated hydrocarbon polymerisations,^8,9^ and actinide (An) silicides showing potential for use as high-density nuclear fuels.^10–13^ Given that the physicochemical properties of the f-elements have been exploited in numerous technologies,^14,15^ it follows that a deeper understanding of f-block silicon chemistry could lead to new applications that complement d-block silicon analogues.

f-Block silicon chemistry has continued to slowly develop and now includes multiple examples of cyclopentadienyl (Cp)-supported M–Si bonds.^6^ Schumann reported the first Ln(iii) examples, [Li(DME)_3_][Ln(Cp)_2_(SiMe_3_)_2_] (Ln = Dy, Ho, Er, Tm, Lu), in the late 1980s;^16–18^ since then, other Ln(iii) Cp-supported silanide complexes have included [Ln(C_5_Me_4_R)_2_{SiH(SiMe_3_)_2_}] (R = Me; Ln = Sc, Y, Nd, Sm, R = Et; Ln = Nd, Sm) by Tilley and Rheingold,^19,20^ [Lu(Cp*)_2_{SiH_2_(*o*-MeOC_6_H_4_)}] (Cp* = {C_5_Me_5_}) by Castillo and Tilley,^8^ [K(2.2.2-crypt)][Y(C_5_H_4_Me)_3_(SiH_2_Ph)] by Evans,^21^ and a series of [Ln(Cp)_3_(SiR_3_)]^−^ anions (Ln = La, Ce; SiR_3_ = Si(H)Mes_2_, Si(H)Ph_2_, Si(Me)Ph_2_, SiPh_3_; Mes = C_6_H_2_Me_3_-2,4,6) by Fang;^22^ Baumgartner and Marschner have reported a wide variety of this class of complex, including [K_2_(18-crown-6)_2_Cp][Ln(Cp)_2_{[Si(SiMe_3_)_2_SiMe_2_]_2_}] (Ln = Tm, Ho, Tb, Gd),^23^ [K(18-crown-6)][Ln(Cp)_3_{Si(SiMe_3_)_3_}] (Ln = Ho, Tm) and [{K(18-crown-6)}_2_Cp][Ln(Cp)_3_{Si(SiMe_3_)_3_}] (Ln = Ce, Sm, Gd, Tm),^24^ and the Y(iii) complexes [K(DME)_4_][Y(Cp)_2_(L)] (L = {[Si(SiMe_3_)_2_SiMe_2_]_2_O}^25^ or {[Si(SiMe_3_)_2_SiMe_2_]_2_}).^26^ Reports of complexes that contain structurally authenticated Cp-supported An–Si bonds are currently limited to the U(iii) silylenes [U(Cp′)_3_{Si(NMe_2_)[PhC(N^t^Bu)_2_]}] (Cp′ = {C_5_H_4_SiMe_3_}) and [U(Cp′)_3_{Si[PhC(N^i^Pr)_2_]_2_}] by Arnold,^27^ and the An(iv) silanides [An(Cp′)_3_{Si(SiMe_3_)_3_}] (An = Th, U) by some of us;^28^ Porchia,^29^ Tilley,^30^ and Marks^31^ have all reported examples of An silanide complexes that were not characterised in the solid state.

It has recently been demonstrated that the extent of covalency in f-block M–Si bonds can be established by a combination of ^29^Si NMR spectroscopy and density functional theory (DFT) calculations.^32^ However, this approach is currently limited to diamagnetic complexes and the vast majority of f-block complexes are paramagnetic; conversely, pulsed EPR spectroscopy has been applied to quantify An–C bond covalency in 5f^3^ U(iii) and 6d^1^ Th(iii) substituted Cp complexes.^33^ Although no U(iii) silanide complex has been structurally authenticated to date, we posited that a substituted Cp-supported system could provide the necessary kinetic stabilisation. Ti(iii) bis-Cp silanide complexes have been extensively studied by fluid solution continuous wave (CW) EPR spectroscopy, including mononuclear complexes with bidentate silanides,^34,35^ and monodentate silanides supported by a tethered donor atom or neutral co-ligand,^34,36–38^ as well as dinuclear Ti(iii) complexes;^39^ however, powder and frozen solution spectra are rare.^39^ The only EPR spectra of *n*d^1^ Zr(iii) and Hf(iii) bis-Cp silanides reported to date are of [K(18-crown-6)][M(Cp)_2_{[Si(SiMe_3_)_2_SiMe_2_]_2_}].^35^

We reasoned that a series of early d- and f-block M(iii) complexes containing M–Si bonds could be achieved by using two substituted Cp ligands and one bulky silanide. We decided to adapt our previous strategy where we prepared An(iv) silanide complexes with three Cp′ and one hypersilanide ligand, {Si(SiMe_3_)_3_};^28^ we were encouraged to continue using hypersilanide as this has provided the largest number of f-block silanide complexes to date,^6^ and to increase the size of the Cp′ ring to Cp′′ ({C_5_H_3_(SiMe_3_)_2_-1,3}) in an effort to maintain kinetic stabilization of the M–Si bonds when the number of coordinated ligands is reduced. The approach of using multiple silyl groups increases the number of signals to assign in ^29^Si NMR spectra, but this was preferred to the use of related alkyl-substituted silanide ligands that we have only previously found applicable to Ln(ii) systems.^32^ Here we report the synthesis of an isostructural family of M(iii) complexes, [M(Cp′′)_2_{Si(SiMe_3_)_3_}] (M = Ti, Zr, La, Ce, Nd, U), providing an opportunity to directly compare the electronic structures of early d- and f-block silanide bonds. This is predominantly achieved using a combination of CW EPR spectroscopy and complete active space self-consistent field-spin orbit (CASSCF-SO) calculations, complemented by supporting characterisation data including single crystal X-ray diffraction, elemental analysis, SQUID magnetometry, and NMR, UV-vis-NIR and ATR-IR spectroscopies. By comparing the electronic structures of 5f^3^ U(iii) with 4f^3^ Nd(iii), and *n*d^1^ Ti(iii) and Zr(iii) with 4f^1^ Ce(iii), we rationalise differences in magnetic anisotropy, d-orbital splitting, orbital mixing, and covalency in the complexes reported herein, and observe clear differences between early d-block, Ln and An metal-silicon bonding regimes.

## Results

### Synthesis

**Scheme 1:**
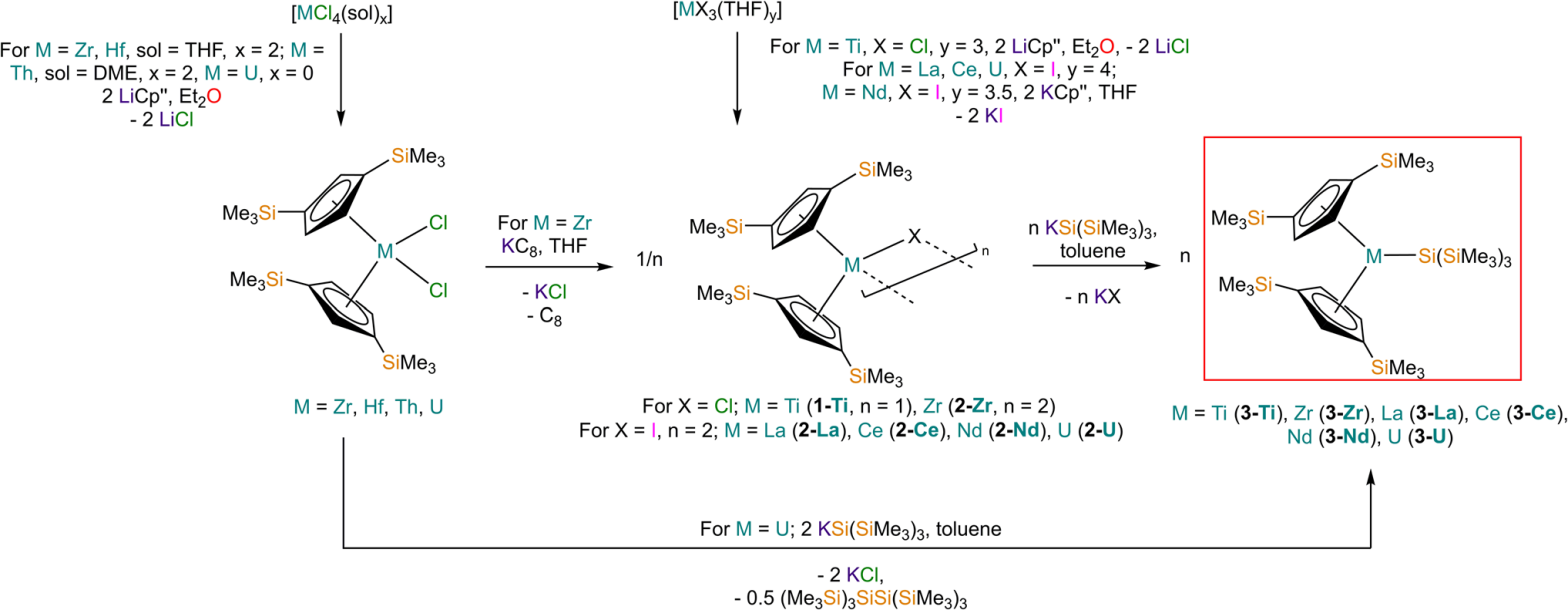
Synthetic routes to complexes 3-M.

Salt elimination reactions between [Ti(Cp′′)_2_Cl] (1-Ti) or [{M(Cp′′)_2_(μ-X)}_2_] (X = Cl, M = Zr (2-Zr); X = I, M = La (2-La), Ce (2-Ce), Nd (2-Nd), U (2-U)) with one or two equivalents of [K{Si(SiMe_3_)_3_}],^40^ respectively, in toluene gave the heteroleptic M(iii) silanide complexes [M(Cp′′)_2_{Si(SiMe_3_)_3_}] (3-M; M = Ti, Zr, La, Ce, Nd, U) in 34–77% yields following work-up and recrystallisation from pentane (Scheme 1). The Ti(iii) starting material 1-Ti was prepared directly from the reaction of two equivalents of LiCp′′^41,42^ with [TiCl_3_(THF)_3_],^43^ whilst 2-Zr was synthesised by reduction of [Zr(Cp′′)_2_Cl_2_]^44^ using KC_8_.^45^ The f-block precursors 2-M were generated,^45–47^ by the reaction of two equivalents of KCp′′^48^ with [MI_3_(THF)_*y*_] (*y* = 4, M = La,^49^ Ce,^49^ U,^50,51^*y* = 3.5, M = Nd;^49^ see ESI for full Experimental details‡). Physical characterisation data of 1-3-M support the proposed formulations.

We found that 3-U could also be prepared by the reaction of [U(Cp′′)_2_Cl_2_]^52^ with two equivalents of [K{Si(SiMe_3_)_3_}] in 77% yield following work-up and recrystallisation from pentane. The reaction of [U(Cp′′)_2_Cl_2_] with one equivalent of [K{Si(SiMe_3_)_3_}] exclusively resulted in reduction of the red U(iv) starting material to the dark green U(iii) complex [{U(Cp′′)_2_(μ-Cl)}_2_], with oxidative coupling of the silanide to give colourless crystals of (Me_3_Si)_3_SiSi(SiMe_3_)_3_; addition of a second equivalent of [K{Si(SiMe_3_)_3_}] to the reaction mixture gave 3-U. The facile reduction of [U(Cp′′)_2_Cl_2_] by [K{Si(SiMe_3_)_3_}] was expected, given the accessible U(iv) → U(iii) reduction potential (*E*^0^ = −0.63 V).^53^ We could not prepare Hf(iii) and Th(iii) homologues of 3-M using these procedures; although the M(iv) precursors [M(Cp′′)_2_Cl_2_] (M = Hf, Th,^52^ see ESI‡ for full Experimental details) can be prepared in appreciable yields. Lappert previously reported that the reduction of [Th(Cp′′)_2_Cl_2_] with Na/K alloy in THF gave [Th(Cp′′)_3_] by ligand scrambling;^54^ we found that some decomposition occurred when [M(Cp′′)_2_Cl_2_] (M = Hf, Th) were treated with KC_8_ in THF, and though no products could be identified from the Hf reaction, we identified crystals of [Th(Cp′′)_3_] by single crystal XRD.^54^

### Structural characterisation

**Fig. 1:**
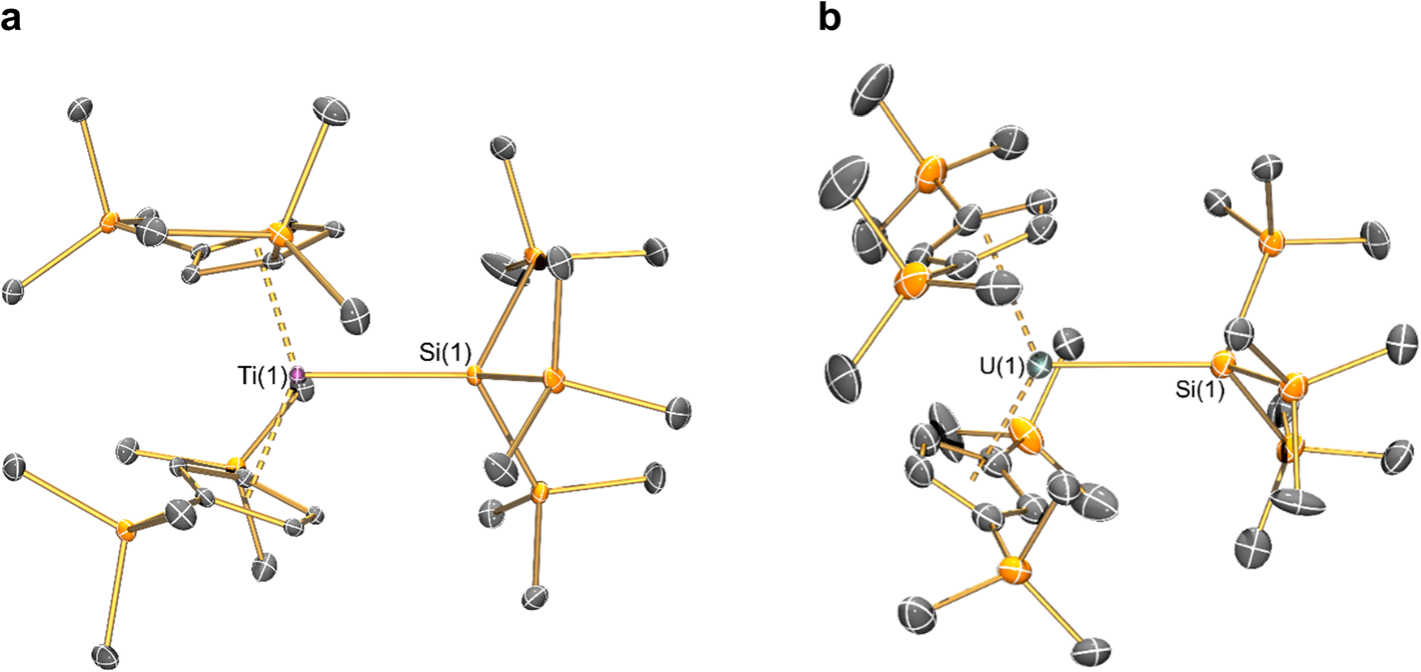
Molecular structures of: (a) 3-Ti determined at 100 K and (b) 3-U determined at 150 K, with selective atom labelling. Displacement ellipsoids set at 30% probability level and hydrogen atoms removed for clarity.

The solid-state molecular structures of [Hf(Cp′′)_2_Cl_2_], 2-Nd, 2-U and 3-M were verified by single crystal XRD, and only 3-M are discussed here for brevity; datasets for [Zr(Cp′′)_2_Cl_2_],^44^ [Th(Cp′′)_2_Cl_2_],^52^ [U(Cp′′)_2_Cl_2_],^52^1-Ti,^46^2-Zr,^45^2-La^47^ and 2-Ce^47^ have previously been reported. As 3-M are isostructural only 3-Ti and 3-U are shown in Fig. 1 and key metrical parameters for all 3-M are compiled in Table 1 (see ESI Fig. S80–S86‡ for depictions of the solid-state structures of other complexes). Treating the Cp′′ centroids as coordination points, 3-M can be described as exhibiting distorted pseudo-trigonal planar geometries. The M–Si distances are consistently ∼0.20–0.40 Å longer than the sums of the respective M–Si single-bond covalent radii reported by Pyykkö^55^ of 2.52 (3-Ti), 2.70 (3-Zr), 2.96 (3-La), 2.79 (3-Ce), 2.90 (3-Nd) and 2.86 Å (3-U). We attribute this to the combination of sterically encumbered Cp′′ and hypersilanide ligands, together with the SiMe_3_ groups in the latter withdrawing a substantial amount of charge density from the silanide centre by negative hyperconjugation.^56,57^

**Table 1:** M–Si and M–Cl bond lengths (Å), mean 
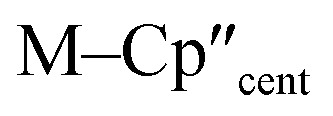
 distances (Å) and 
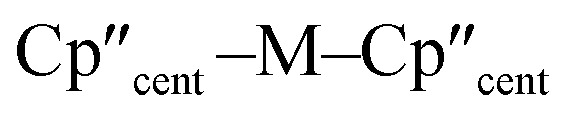
 angles (°) for 1-M and 3-M

Parameter	1-Ti^46^	1-Zr^a^	3-Ti	3-Zr	3-La	3-Ce	3-Nd	3-U
M–Si or M–Cl	2.347(3)	2.4534	2.7720(2)	2.902(2)	3.178(2)	3.153(2)	3.112(2)	3.116(2)
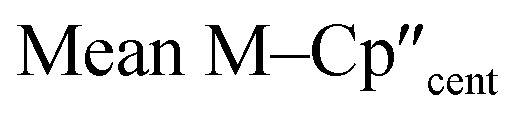	2.037(4)	2.1801	2.0508(2)	2.1841(4)	2.5215(2)	2.5005(2)	2.4489(9)	2.4726(2)
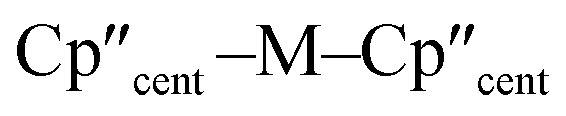	138.45(14)	134.58	141.04(3)	133.830(2)	132.54(5)	133.11(3)	132.37(2)	131.02(4)

a DFT-optimised structure.

Complex 3-U (Fig. 1b) exhibits a relatively short U–Si distance of 3.116(2) Å compared to [U(Cp′)_3_{Si(NMe_2_)[PhC(N^t^Bu)_2_]}] (3.1637(7) Å) and [U(Cp′)_3_{Si[PhC(N^i^Pr)_2_]_2_}] (3.1750(6) Å),^27^ which contain dative silylene U(iii)–Si bonds, in accord with the increased electrostatic attraction between the negatively charged hypersilanide and the U(iii) centre in 3-U. Additionally, the U–Si bond length of 3-U is *ca.* 0.05 Å longer than the previously reported complex [U(Cp′)_3_{Si(SiMe_3_)_3_}] (3.0688(8) Å),^28^ consistent with the increased ionic radii of U(iii) *vs.* U(iv) (six-coordinate U(iii) = 1.025 Å, whilst U(iv) = 0.89 Å).^58^ There are essentially negligible changes to the 
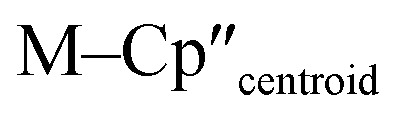
 distances and 
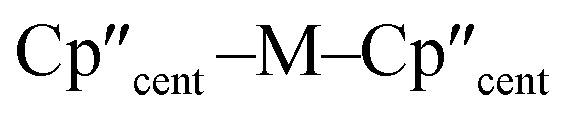
 angles when comparing 3-M to 1-Ti and 2-M for the same metal. The orientation of the Cp′′ ligands with respect to each other are typically invariant within each series, with the ring trimethylsilyl substituents arranged to minimise inter-ligand steric repulsions;^47^1-Ti and 3-Ti are outliers due to the high steric demands about the smaller Ti(iii) centre (six-coordinate Ti(iii) = 0.67 Å).^58^

Previously reported neutral Ti(iii) bis-Cp silanide complexes contain shorter Ti–Si bonds than that found for 3-Ti (Fig. 1a), though these all contain less sterically demanding Cp ligands and a PMe_3_ ancillary ligand: [Ti(Cp)_2_(SiH_3_)(PMe_3_)] (2.594(2) Å),^34^ [Ti(Cp)_2_(SiH_2_Ph)(PMe_3_)] (2.635(7) Å),^59^ and [Ti(Cp)_2_(SiHPh_2_)(PMe_3_)] (2.652(2) Å).^36^ However, the mean Ti–Si bond length of the anionic Ti(iii) complex [K(18-crown-6)][Ti(Cp)_2_{[Si(SiMe_3_)_2_SiMe_2_]_2_}] (2.770(3) Å),^60^ which features a bidentate silanide ligand, is statistically equivalent to that of 3-Ti; similarly, the Zr–Si bond length of 3-Zr is comparable to those present in [K(18-crown-6)][Zr(Cp)_2_{[Si(SiMe_3_)_2_SiMe_2_]_2_}] (Zr–Si: 2.8503(11), 2.8950(10) Å)^35^ and the Ce–Si distance of 3-Ce is invariant to that of [{K(18-crown-6)}_2_Cp][Ce(Cp)_3_{Si(SiMe_3_)_3_}] (Ce–Si: 3.155(5) Å).^24^ Conversely, the La–Si distance in 3-La is intermediate to those seen in [La{μ-η:^5^κ^1^-SiC_4_(SiMe_3_)_2_-1,4-Ph_2_-2,3}(μ-η^8^:η^8^-C_8_H_8_)K(THF)_3_]_2_ for the η^5^ – (3.0888(5) Å) and κ^1^ – (3.2908(6) Å) bound silole.^61^ Complex 3-Nd contains the first structurally authenticated Nd–Si bond, precluding a literature comparison; however, the Ln–Si distances for the 3-Ln series vary in accord with the expected periodic trend for Ln(iii) ionic radii.^58^

### NMR spectroscopy

Multinuclear NMR spectroscopy was performed on 1-Ti, 2-M and 3-M (see ESI Fig. S1–S42‡ for annotated NMR spectra); we focus here on the spectra of 3-M. With the exception of diamagnetic 3-La, the collection of reliable NMR spectra for 3-M was challenging due to paramagnetism. C_6_D_6_ solutions of 3-Ti and 3-Zr also showed decomposition at ambient temperatures (*t*_½_*ca.* 2 h); *δ*_Si_ resonances at −116.11 and −11.97 ppm grew in intensity during data collection (see ESI Fig. S25 and S27‡) that were assigned to the organosilane HSi(SiMe_3_)_3_ by comparison with an authentic sample.^62^ An additional resonance observed in the ^29^Si NMR spectrum of 3-Ti at −21.83 ppm was attributed to silicone grease, whilst in 3-Zr a signal at *δ*_Si_ = −9.83 ppm could not be confidently assigned. Previous reports of complexes containing Ti(iii)–Si and Zr(iii)–Si bonds showed that solution decomposition processes are commonly observed by NMR spectroscopy.^24,35,60^ The rest of the 3-M series were stable in C_6_D_6_ solution at ambient temperature for a sufficient duration for multinuclear NMR spectra to be acquired (*ca.* 1 h; experiments had to be performed at relatively fast acquisition times to obtain data that are representative of freshly prepared solutions, *e.g.* 1D ^29^Si INEPT 128 scans).

For diamagnetic 3-La the ^1^H NMR spectrum showed the four expected resonances: two inequivalent Cp-H signals for the 4,5- and 2-Cp-H positions at 7.17 and 7.46 ppm, respectively, and two resonances in a ratio of 4 : 3 for the chemically inequivalent trimethylsilyl environments, *δ*_H_: 0.25 (Cp-SiC*H*_3_) and 0.55 ppm (Si(SiC*H*_3_)_3_); these correlated with five resonances in the ^13^C NMR spectrum. The ^29^Si NMR spectra of 3-La revealed three resonances; those at −6.57 and −10.32 ppm were respectively assigned as Si(*Si*CH_3_)_3_ and Cp-*Si*CH_3_*via* a ^1^H-^29^Si HMBC experiment, whilst a weak signal at −130.25 ppm correlated with the ^1^H resonances of the hypersilanide ligand; the latter signal can only be tentatively assigned as the quaternary metal-bound silicon atom due to quadrupolar broadening from coupling to 99.9% abundant *I* = 7/2 ^139^La nuclei.

The ^1^H NMR spectra of paramagnetic 3-M all exhibited signals for the two chemically inequivalent trimethylsilyl environments; resonances for the two Cp′′ ring proton environments were not observed due to paramagnetic line-broadening. As with 3-La, Cp-SiC*H*_3_ resonances in all cases are upfield of those assigned to Si(SiC*H*_3_)_3_, albeit paramagnetically shifted (Cp-SiC*H*_3_*δ*_H_/ppm: 0.14 (3-Ti), 0.88 (3-Zr), −8.33 (3-Ce), −11.64 (3-Nd), −14.79 (3-U); Si(SiC*H*_3_)_3_*δ*_H_/ppm: 1.60 (3-Ti), 2.97 (3-Zr), −1.11 (3-Ce), −2.02 (3-Nd), −5.90 (3-U)). Both of the trimethylsilyl environments were present in the ^13^C NMR spectrum of 3-U (*δ*_C_/ppm: −28.48, Cp-SiMe_3_; 17.11, Si(SiMe_3_)), whilst only the hypersilanide resonances were seen for 3-Ce (8.39 ppm) and 3-Nd (19.79 ppm) and no signals could be assigned for 3-Ti and 3-Zr; in all cases assignments were confirmed by ^1^H-^13^C HSQC correlation experiments. Finally, only one signal was observed in the ^29^Si NMR spectrum of 3-Nd (22.18 ppm), whilst no ^29^Si NMR signals could be seen for 3-Ti, 3-Zr, 3-Ce or 3-U. The experimental parameters of the ^1^H-^29^Si HMBC experiment prohibited correlation with ^1^H NMR resonances, therefore we cannot confidently assign the ^29^Si NMR resonance observed for 3-Nd; however, this is unlikely to be due to the metal-bound silicon atom, as this resonance is not observed in the 1D^29^Si NMR spectra of diamagnetic 3-La. To the best of our knowledge there have not been any previous reports of ^29^Si NMR chemical shifts for paramagnetic M(iii)–Si complexes in the literature for the metals studied here.^63^

### Magnetism

**Fig. 2:**
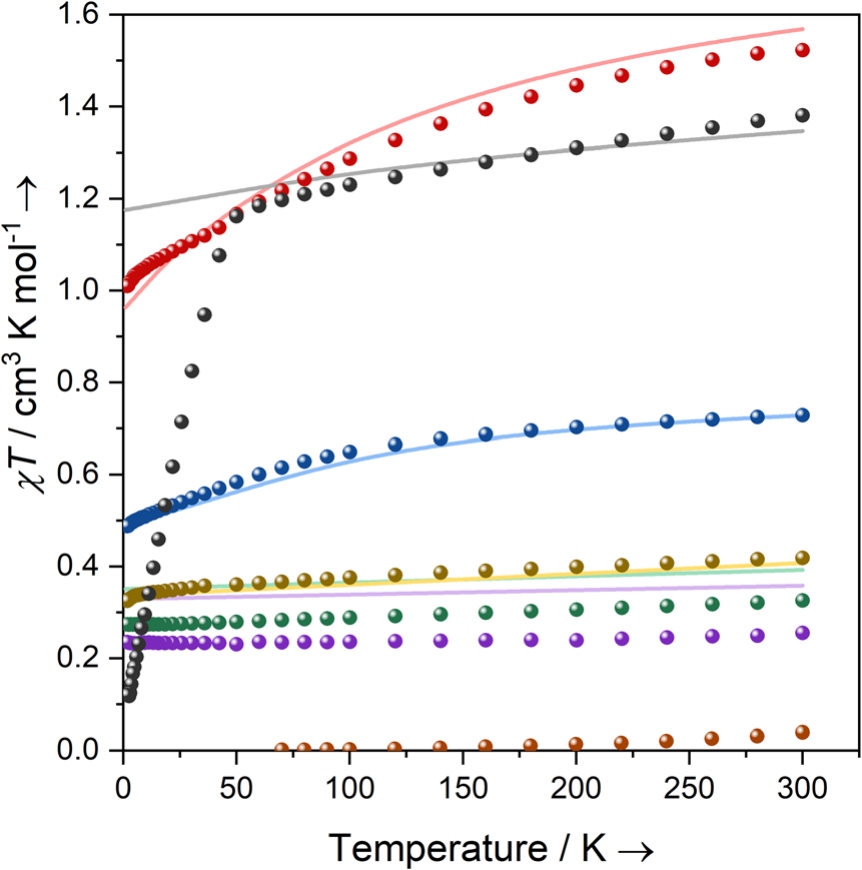
Temperature dependence of the product of magnetic susceptibility and temperature (*χT*) for powder samples of 1-Ti (green), 2-Zr (orange), and 3-M: M = Ti (yellow), Zr (purple), Ce (blue), Nd (red), U (black) with CASSCF-calculated curves of mononuclear complexes in corresponding colours (solid lines). CASSCF of 3-U uses an active space of 7 × 5f orbitals averaging over 35 spin quartets and 112 spin doublets.

Solutions of 1-Ti, 2-Zr and 3-M (M = Ti, Zr, Ce, Nd, U) in C_6_D_6_ were prepared at 0 °C and the effective magnetic moments (*μ*_eff_) and molar magnetic susceptibilities (*χ*_M_) were measured by the Evans method immediately upon warming to 300 K (Table 2, ESI Fig. S43–S52‡).^64^ Solution magnetic susceptibilities for 1-Ti and 3-M are in good agreement with the corresponding data obtained from powdered samples examined by variable-temperature SQUID magnetometry, and CASSCF calculations (Table 2, Fig. 2 and ESI Fig. S94a–S99a‡). Magnetic susceptibility and field-dependent magnetisation data (ESI Table S5 and Fig. S94–S96‡) for 1-Ti, 3-Ti and 3-Zr indicate isolated *S* = 1/2 systems, as expected for mononuclear *n*d^1^ complexes. Previous studies of 2-Zr have reported that this complex is essentially diamagnetic based on NMR chemical shifts,^65^ but also that it exhibits an EPR spectrum in solution.^45^ We find near-zero magnetic susceptibility for a powder sample of 2-Zr, implying that the two *S* = 1/2 centres are strongly antiferromagnetically coupled in the solid state (Fig. 2). A solution of 2-Zr in C_6_D_6_ at 300 K was found to be paramagnetic by the Evans method, but less than expected for two uncoupled *S* = 1/2 (0.26 compared to 0.75 cm^3^ K mol^−1^), indicating weaker antiferromagnetic coupling than in the solid state, some minor sample decomposition, and/or the presence of some monomeric [Zr(Cp′′)_2_Cl] (1-Zr) in solution as proposed by Antiñolo *et al.*^65^ SQUID magnetometry and EPR spectra of 2-Ce, 2-Nd and 2-U showed extensive exchange interactions between the metal ions; these data are challenging to model,^66^ and will be communicated in a separate publication as they are outside the main focus of this study.

**Table 2:** Magnetic moment, *μ*_eff_ (*μ*_B_), and product of the molar susceptibility and temperature, *χ*_M_*T* (cm^3^ K mol^−1^), of 1-Ti and 3-M at 300 K. Determined by Evans method on solutions in C_6_D_6_, SQUID magnetometry on powder samples, CASSCF calculations and free ion values for monomeric ions

Complex	Solution		Powder		CASSCF calculations		Free ion^14^	
	*μ*_eff_	*χ*_M_ *T*	*μ*_eff_	*χ*_M_ *T*	*μ*_eff_	*χ*_M_ *T*	*μ*_eff_	*χ*_M_ *T*
1-Ti	1.63	0.33	1.61	0.33	1.77	0.39	1.73	0.38
2-Zr	1.43	0.26	0.56	0.04	—	—	—	—
3-Ti	1.93	0.47	1.83	0.42	1.81	0.41	1.73	0.38
3-Zr	1.71	0.37	1.43	0.26	1.69	0.36	1.73	0.38
3-Ce	2.34	0.69	2.41	0.73	2.41	0.73	2.54	0.81
3-Nd	3.54	1.56	3.49	1.52	3.54	1.57	3.62	1.64
3-U	3.16	1.25	3.33	1.38	3.28^a^	1.35^a^	3.62	1.64

a CAS(3,7) active space averaging over all 5f^3^ configurations.

Complexes 3-Ce and 3-Nd are more strongly magnetic than 3-Ti and 3-Zr due to the presence of orbital angular momentum; the smooth decrease in *χ*_M_*T* with reducing temperature arises from crystal field splitting of the lowest total angular momentum multiplet. Assuming well-isolated ground Kramers doublets for 3-Ce and 3-Nd, the 2 K magnetisation data suggest |±5/2〉 and |±9/2〉 ground states, respectively (ESI Table S5, Fig. S97b and S98b;‡ in an axial crystal field, *M*_sat_ = ½*g*_J_*m*_J_, where *g*_J_ is the Landé *g*-factor^67^). For 3-U the *μ*_eff_ (*χ*_M_*T*) of 3.32 *μ*_B_ (1.38 cm^3^ K mol^−1^) at 300 K is characteristic of a ^4^I_9/2_ U(iii) ion;^27^ this value smoothly decreases to 3.05 *μ*_B_ (1.16 cm^3^ K mol^−1^) at 50 K, then rapidly decreases to 0.98 *μ*_B_ (0.12 cm^3^ K mol^−1^) at 1.8 K. The sharp decrease below 50 K occurs due to slow thermalisation of the sample on cooling (see ESI for details‡). The *M*_sat_ value at 2 K and 7 T is diagnostic of a |±9/2〉 ground state (ESI Fig. S99b‡). Alternating current susceptibility measurements on 3-U show out-of-phase signals owing to slow relaxation of magnetisation below 5 K (ESI Fig. S102–S105‡), which modelling suggests arises due to Raman and quantum tunnelling of magnetisation (QTM) processes (ESI Fig. S106 and Tables S6, S7‡). Slow relaxation is common for U(iii) complexes,^68–75^ however, as there is no effective barrier observed for the reversal of magnetisation and closed hysteresis loops around zero field (ESI Fig. S101‡), we do not refer to 3-U as a single-molecule magnet, following several literature definitions.^76,77^

### UV-vis-NIR spectroscopy

**Fig. 3:**
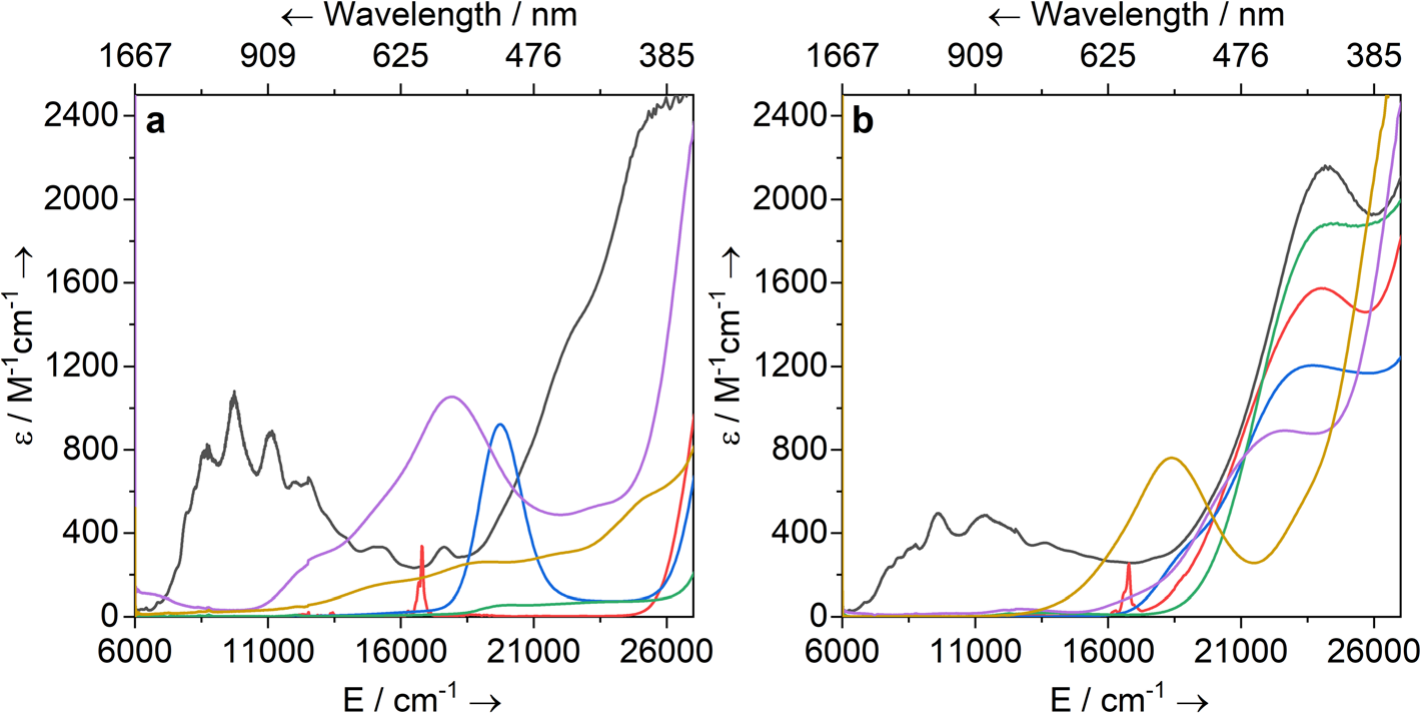
UV-vis-NIR spectra of (a) 1-Ti and 2-M (M = Zr, La, Ce, Nd, U) and (b) 3-M (M = Ti, Zr, La, Ce, Nd, U) in toluene (2 mM) between 6000 and 27 000 cm^−1^ (1667–370 nm). Legend: M = Ti (yellow), Zr (purple), La (green), Ce (blue), Nd (red), U (black).

Solutions of 1-Ti, 2-M and 3-M were prepared at 0 °C in toluene (2 mM concentration for all complexes) and warmed to room temperature to immediately record UV-vis-NIR spectra (Fig. 3; see ESI Fig. S65–S79‡ for individual spectra). Some spectra, most notably 2-Nd and 2-U, contain several jagged features; this is attributed to a combination of the most intense absorption maxima being close to the detector limit at the concentrations used, and the spectral resolution of 1 nm. Intense charge transfer (CT) absorptions tailing in from the UV region are found for 3-Zr (*ῡ*_max_ = 22 400 cm^−1^; *ε* = 890 M^−1^ cm^−1^), 3-La (*ῡ*_max_ = 22 200 cm^−1^; *ε* = 1880 M^−1^ cm^−1^), 3-Ce (*ῡ*_max_ = 23 500 cm^−1^; *ε* = 1200 M^−1^ cm^−1^), 3-Nd (*ῡ*_max_ = 24 000 cm^−1^; *ε* = 1570 M^−1^ cm^−1^) and 3-U (*ῡ*_max_ = 24 200 cm^−1^; *ε* = 2160 M^−1^ cm^−1^) (Fig. 3b). These transitions were not observed for 1-Ti or 2-M (Fig. 3a), and therefore can be assigned to changes in CT upon replacing a halide with hypersilanide. Apart from this CT band, the spectra of 2-La, 3-Zr and 3-La are otherwise essentially featureless.

Complex 3-Nd has an additional absorption in the visible region (*ῡ*_max_ = 16 800 cm^−1^; *ε* = 260 M^−1^ cm^−1^), as does 2-Nd (*ῡ*_max_ = 16 800 cm^−1^; *ε* = 330 M^−1^ cm^−1^; ESI Fig. S71 and S78‡), thus we propose these bands are Nd(iii) f–f transitions of the ^4^I_9/2_ → ^4^G_5/2_ states.^78,79^ Complex 3-Ce is red and has a shoulder on the CT band at 18 700 cm^−1^, while 2-Ce is bright pink and has an intense, broad absorption at *ῡ*_max_ = 19 700 (*ε* = 920 M^−1^ cm^−1^); these are assigned to 4f^1^ → 5d^1^ transitions, as observed for other Ce(iii) complexes with Cp ligands.^80–84^ The NIR region of 3-U studied here (*ῡ*_max_ > 6000 cm^−1^) is populated with a broad set of absorptions (*ε* = 200–500 M^−1^ cm^−1^) corresponding to normally Laporte-forbidden f–f transitions of significant intensity (Fig. 3a). The increased intensity is attributed to mixing of 5f orbitals with 6d and ligand orbitals allowing for intensity-stealing;^53,85,86^ similar transitions are observed in 2-U (Fig. 3b) and previously reported U(iii) complexes.^27,74,87^

The UV-vis-NIR spectrum of 3-Ti features an intense CT band that tails into the visible region up to *ca.* 22 000 cm^−1^ and a second broad absorption band at *ῡ*_max_ = 18 300 cm^−1^ (*ε* = 760 M^−1^ cm^−1^) that is assigned to overlapping d–d transitions with substantial charge-transfer character, accounting for their higher than expected intensities.^88^ The spectrum for 1-Ti also exhibits multiple overlapping d–d transitions >14 000 cm^−1^. Finally, the spectrum for 2-Zr shows a broad absorption at 17 900 cm^−1^ (*ε* = 1060 M^−1^ cm^−1^) with a weaker shoulder at 13 100 cm^−1^ (*ε* = 300 M^−1^ cm^−1^), and a broad low intensity NIR feature at *ca.* 6300 cm^−1^ (*ε* = 110 M^−1^ cm^−1^). We assign the former two absorptions as d–d transitions based on CASSCF calculations (see below), which also suggests the latter is not a 2-Zr d–d transition; this low-energy feature could arise from a fraction of 1-Zr in toluene solution, as suggested by the Evans method magnetic moment (see above).

### EPR spectroscopy

EPR spectra have been recorded at two frequencies where possible and simulations have modelled both spectra simultaneously in EasySpin;^89^ full details of simulations are included in the ESI (Tables S8–S12 and Fig. S107–S121‡). EPR spectra of 2-Ce, 2-Nd and 2-U show significant exchange interactions between the metal ions, and these will be studied in a separate publication.

#### Titanium and zirconium, *n*d^1^

A fluid solution spectrum of 1-Ti gives *g*_iso_ = 1.9620 (ESI Fig. S107‡), in agreement with the literature value in toluene (*g*_iso_ = 1.961)^90^ and confirming that 1-Ti is stable in solution (*g*_ave_ from frozen solution is 1.9624, see below). In contrast, 3-Ti and 3-Zr decompose over several hours at room temperature in fluid aromatic solutions, consistent with *n*d^1^ complexes being kinetically labile, particularly when coordinatively unsaturated.^91^ Reproducible frozen solution spectra for 2-Zr, 3-Ti and 3-Zr could be obtained for these complexes by dissolving powders in a solvent mixture (9 : 1 toluene : hexane) that was pre-cooled to *ca.* 250 K, then frozen at 77 K and measured immediately. The *g*-values obtained for monomeric *n*d^1^ complexes (1-Ti, 3-Ti and 3-Zr) with this method are generally in excellent agreement with the powder spectra (Table 4).

The powder EPR spectrum of 2-Zr is isotropic with *g* = 1.9825 and a half-field transition, indicating that these transitions arise from a triplet state, reflecting the dimeric structure in the solid state (ESI Fig. S112–S114‡). There is one previous report of an EPR spectrum of 2-Zr with *g*_iso_ = 1.9506 and *A*_iso_ = 60 MHz in fluid toluene solution at 300 K.^45^ Measurement of a frozen solution sample of 2-Zr (see Section 12 of the ESI‡) gives clearly anisotropic spectra (*g*_1_ = 1.9961, *g*_2_ = 1.9834, *g*_3_ = 1.8618; the *g*_3_ resonance is at 360 and 1307 mT at X- and Q-band frequencies, respectively, far outside the spectral range of the powder spectrum of 2-Zr, ESI Fig. S115 *cf.* S112‡) and the half-field transition is absent (ESI Fig. S114‡). These data suggest that the dimer breaks apart in solution to form monomeric 1-Zr (*S* = 1/2), in accord with solution magnetic susceptibility and UV-vis-NIR data (see above); there may be some 2-Zr still present in the frozen solution; however, at 50 K the signal (arising from Boltzmann population of the triplet state) is dwarfed by the *S* = 1/2 spectrum. Thus, we henceforth discuss these frozen solution results as representing 1-Zr.

**Fig. 4:**
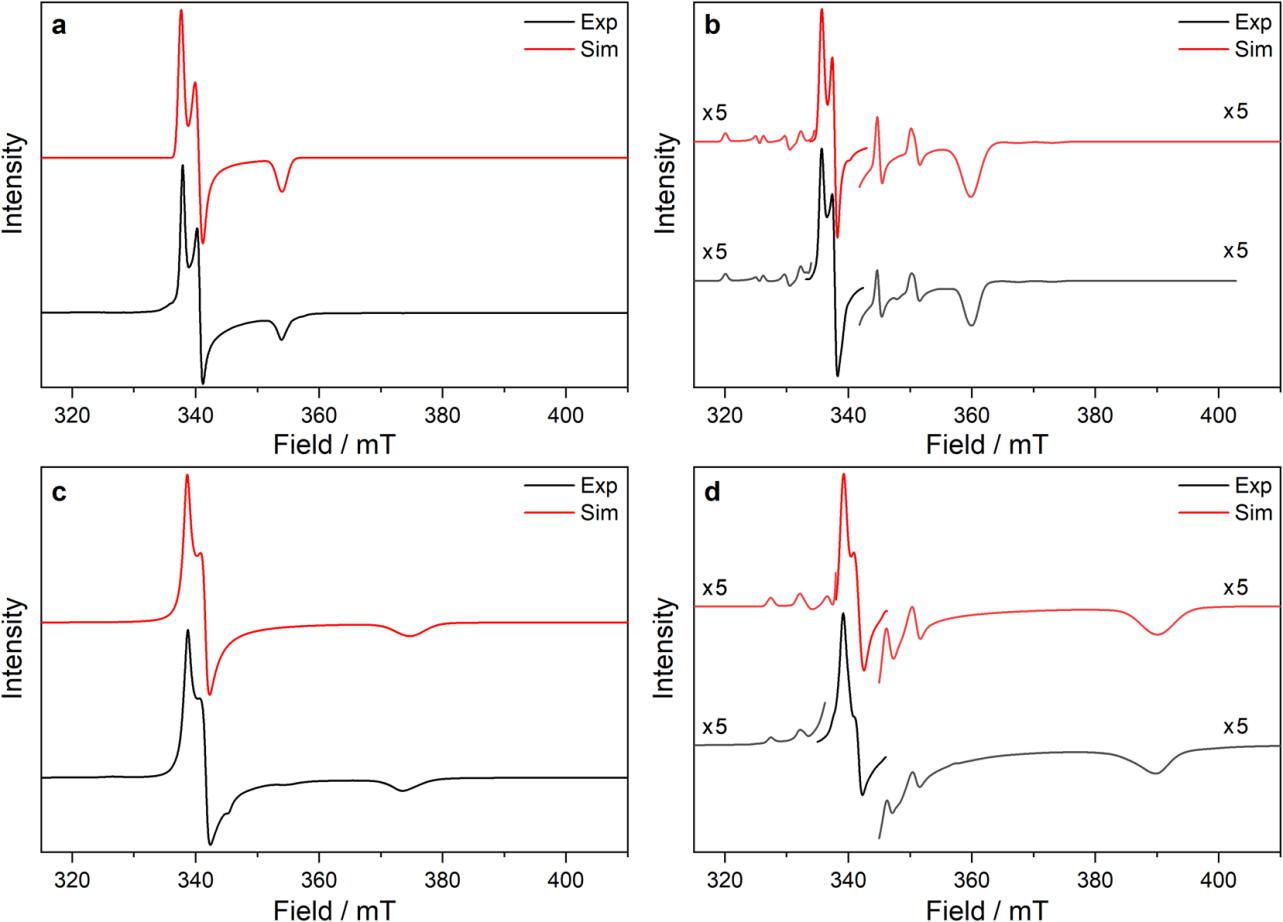
Frozen solution CW X-band EPR spectra of (a) 1-Ti at 130 K and 5 mM, (b) 1-Zr at 50 K and 5 mM, (c) 3-Ti at 130 K and 1 mM, (d) 3-Zr at 130 K and 5 mM in 9 : 1 toluene : hexane. Simulations using parameters from ESI Tables S9 and S10‡ are shown in red.

Ti(iii) and Zr(iii) are *S* = 1/2 and are expected to have anisotropic *g*-values (*g*_1_, *g*_2_, *g*_3_) close to the free electron value (*g*_e_ ≈ 2.0023), with deviations from *g*_e_ (Δ*g*_*i*_ = *g*_*i*_ − *g*_e_) reflecting second order spin–orbit coupling with low energy crystal field states.^92^ X-band EPR spectra of frozen solutions of 1/3-Ti clearly show three *g*-features (^47^Ti/^49^Ti hyperfine coupling not resolved), while spectra of 1/3-Zr show a similar rhombic pattern superimposed with hyperfine coupling to the ^91^Zr nuclear spin (11% abundant *I* = 5/2; Table 4, Fig. 4, ESI Fig. S115 and S117‡). Superhyperfine coupling to α-^29^Si and β-^29^Si have been observed before,^35^ but are not resolved here. Complexes 1/3-Ti/Zr have a consistent axial pattern of *g*-values reflecting their similar structures; Δ*g*_1_ is −0.003 to −0.017, Δ*g*_2_ is −0.02 to −0.03, and Δ*g*_3_ is −0.09 to −0.28 (Fig. 4, Table 3 and ESI Table S11‡). The *g*_3_ value is most sensitive to the different complexes, with larger Δ*g*_3_ for 3-Ti/Zr*vs.*1-Ti/Zr, and also larger Δ*g*_3_ for Zr(iii) *vs.* Ti(iii) in isostructural complexes. Complexes 3-Ti/Zr are significantly more anisotropic than previous reports of Ti(iii) and Zr(iii) bis-Cp silanide complexes, which are pseudo-tetrahedral: Δ*g*_ave_ values (*g*_ave_ = (*g*_1_ + *g*_2_ + *g*_3_)/3) of 3-Ti/Zr are −0.076 to −0.109 compared to Δ*g*_iso_ = −0.004 to −0.021.^34–39^ The EPR spectra of 1/3-Ti/Zr are more comparable to pseudo-trigonal planar bent metallocenes [M(Cp^R^)_2_X].^88^ The pattern of ^91^Zr hyperfine constants (Table 4; one large and two small) are typical for 4d_*z*^2^_^1^ ground states with *z* aligned along *g*_1_,^92^ which also suggests an electronic structure tending to pseudo-trigonal planar environments.

**Table 3:** CW EPR *g*-values of studied complexes as solid powders or in toluene : hexane (9 : 1) frozen solutions (FS). Band frequency: *X* = 9.37–9.47 GHz, *K* = 24 GHz, *Q* = 34 GHz

Complex	State/temperature	Band(s)	*g*_1_	*g*_2_	*g*_3_
1-Ti	FS/130 K	X, Q	1.9990	1.9818	1.9065
	Powder/50 K	X, K	1.9957	1.9800	1.8964
	CASSCF	—	1.9994	1.9655	1.8370
3-Ti	FS/130, 50 K	X, Q	1.9955	1.9781	1.8030
	Powder/55, 50 K	X, Q	1.9946	1.9778	1.7914
	CASSCF	—	1.9950	1.9595	1.7173
1-Zr	FS/50 K	X, Q	1.9961	1.9834	1.8618
	CASSCF	—	1.9973	1.9524	1.8214
2-Zr	Powder/50 K	X, Q	1.9825^a^	1.9825^a^	1.9825^a^
	CASSCF	—	1.9944	1.9224	1.8262
3-Zr	FS/130 K	X, Q	1.9874	1.9728	1.7280
	Powder/130 K	X, Q	1.9853	1.9758	1.7176
	CASSCF	—	1.9897	1.9438	1.6719
3-Ce	FS/7 K	X	3.907	<0.4	<0.4
	Powder/8, 5 K	X, Q	3.884	0.888	0.493
	CASSCF	—	3.775	1.016	0.562
3-Nd	FS/7 K	X	5.225	0.360	<0.4
	Powder/7, 5 K	X, Q	5.490	<0.4	<0.4
	CASSCF	—	5.526	0.296	0.146
3-U	FS/5 K	X	5.949 (44%), 5.536 (56%)^b^	<0.4	<0.4
	Powder/7, 5 K	X, Q	6.055	<0.4	<0.4
	CASSCF	—	6.130	0.076	0.007

a Anisotropy in *g*-values not resolved.

b Two species observed in solution, relative abundance given in brackets.

**Table 4:** CW EPR hyperfine coupling constants (MHz) of 1-Zr and 3-Zr as toluene : hexane (9 : 1) frozen solutions (FS). Band frequency: *X* = 9.37–9.47 GHz, *Q* = 34 GHz

Complex	State/temperature	Band	*A*_1_	*A*_2_	*A*_3_
1-Zr	FS/50 K	X, Q	173.5	137	138
3-Zr	FS/130 K	X, Q	131	91	80^a^

a Upper limit of *A*_3_: hyperfine not resolved within linewidth.

Powder spectra of ground samples of 1-Ti, 3-Ti, 2-Zr and 3-Zr are considerably broadened relative to frozen solution spectra (likely owing to unresolved intermolecular dipolar interactions), resulting in overlap of the *g*_1_ and *g*_2_ features (see ESI Fig. S108, S110, S112 and S116‡).

#### Cerium 4f^1^, neodymium 4f^3^ and uranium 5f^3^

**Fig. 5:**
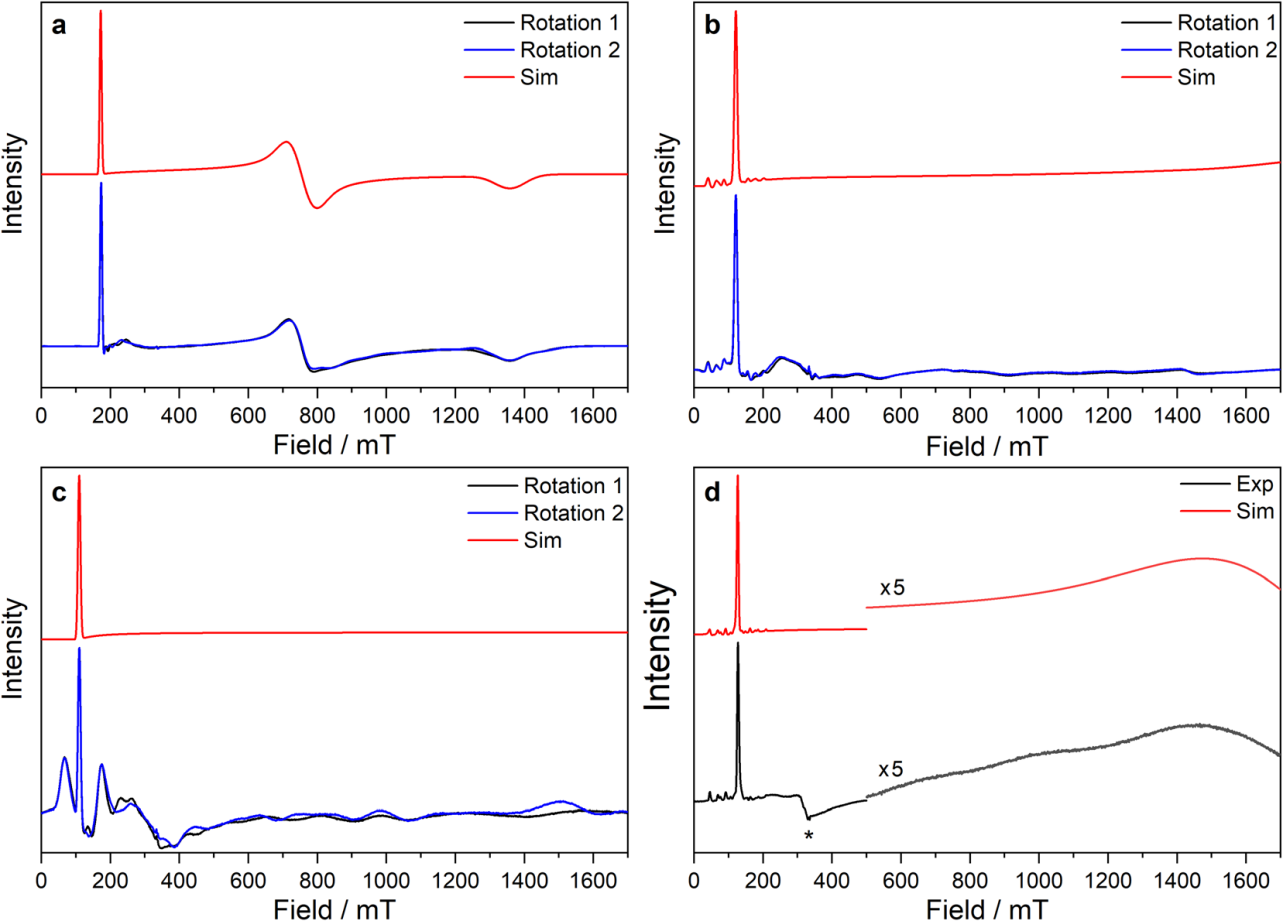
CW X-band EPR spectra of (a) 3-Ce powder at 8 K, (b) 3-Nd powder at 7 K, (c) 3-U powder at 7 K, (d) 15 mM frozen solution of 3-Nd in 9 : 1 toluene : hexane at 7 K. Two perpendicular orientations of powder spectra are shown in black and blue. Simulations using parameters from ESI Table S12‡ are shown in red. Asterisk (*) denotes feature intrinsic to cavity.

The powder EPR spectra of 3-Ce at 7 K are characteristic of a rhombic *S*_eff_ = 1/2 with three *g*-features characterised at X-band and one at Q-band (Fig. 5a, ESI Fig. S118a and b‡). Simultaneous modelling of the spectra give *g*_1_, *g*_2_ and *g*_3_ of 3.884, 0.888 and 0.493, respectively. The large *g*_1_ suggests majority |±5/2〉 component of the ground state, consistent with the *M*_sat_ value. The frozen solution X-band EPR spectrum of 3-Ce in toluene : hexane (9 : 1) has *g*_1_ of 3.907 and transverse *g*-values less than 0.4 (ESI Fig. S118c‡), indicating a geometry change in solution to form a more axial ground state.

The X-band powder EPR spectrum of 3-Nd at 7 K revealed only a single *g*_1_ feature of the ground Kramers doublet with resolved ^143^Nd and ^145^Nd hyperfine coupling within the accessible field range (Fig. 5b and ESI Fig. S119a–c‡). The *g*_1_ value of 5.490 is less than the value of 6.55 expected for a pure |±9/2〉, but is larger than the maximum *g* value for a pure |±7/2〉 state, implying a mixed |±9/2〉 ground doublet, consistent with the *M*_sat_ value. Whilst *g*_2_ and *g*_3_ were not visible in the powder spectrum, a frozen solution X-band spectrum showed part of a *g*_2_ feature at the highest fields, estimated as *g*_2_ = 0.360 (Fig. 5d).

The powder X-band EPR spectrum of 3-U at 7 K exhibits a sharp feature at *g* = 6.055 (Fig. 5c), assigned as the *g*_1_ feature of an axial *S*_eff_ = 1/2 ground state with *g*_2_, *g*_3_ < 0.4. This indicates a majority |±9/2〉 ground state, consistent with the *M*_sat_ value. Two broader peaks were observed at 67 and 175 mT (*g* = 9.94 and 3.82), however, the Q-band EPR spectrum was too weak to establish whether these peaks behaved as true *g*-features. The extra features cannot be explained by dipolar interactions (ESI Fig. S121‡), and we have not been able to assign them. Solution X-band spectra of 3-U at 5 K showed two sharp *g*_1_ features at 5.949 and 5.536, indicating two very similar axial species present in solution (ESI Fig. S120c and d‡).

### CASSCF calculations

We have investigated the electronic structures of 1-Ti/Zr, 2-Zr and 3-M by CASSCF-SO calculations (performed in OpenMolcas,^93,94^ see ESI for details‡). For discussion of the data we adopt the coordinate system of Petersen and Dahl, with *x* along the M–Si/Cl axis, *y* tangential to Cp′′–M–Cp′′ and *z* perpendicular to the plane defined by the Cp′′ centroids and coordinating Si/Cl atom.^95,96^

#### Titanium and zirconium, *n*d^1^

For 1-Ti, 3-Ti and 3-Zr we used the single crystal XRD structures, while for 1-Zr we used a DFT-optimised geometry (ESI Table S13‡). State-averaged (SA) CAS(7,8)SCF calculations were performed averaging over five doublets, with an active space of the five 3d or 4d orbitals and three almost doubly occupied M–L bonding orbitals with considerable (17–25%) d_*xy*_, d_*yz*_, and d_*x*^2^−y^2^_ character (ESI Fig. S122–S125‡). Calculations for 2-Zr used the crystal structure with one Zr(iii) centre replaced with diamagnetic Y(iii) (2-Zr′), averaging over 5 doublets in an active space of five 4d orbitals, three 4p orbitals and four almost doubly occupied M–L bonding orbitals with 15–19% d character (ESI Fig. S126‡). The active space differs for 2-Zr′ because the symmetry match of the Zr 4d_*xz*_ orbital and chloride σ-orbitals results in stronger bonding and anti-bonding interactions compared to 1/3-Ti/Zr, requiring a pair of orbitals to be included in the active space. Furthermore, 4p orbitals were included as they displaced the M–L bonding orbitals in orbital optimisation if not included initially.

**Fig. 6:**
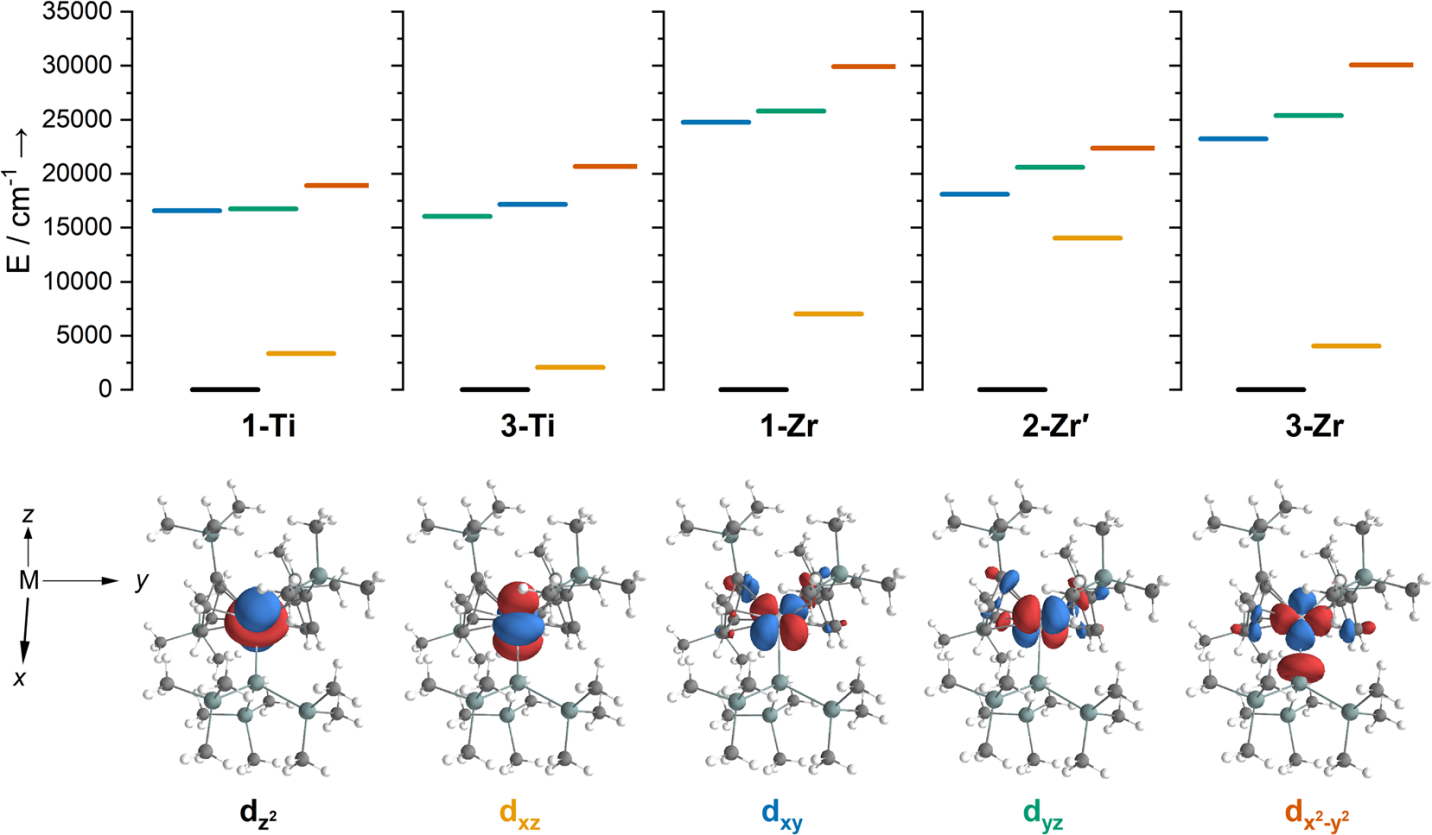
Top – state energies for (left to right) 1-Ti, 3-Ti, 1-Zr, 2-Zr′ and 3-Zr, coloured according to majority d-orbital contribution to singly occupied state-specific natural orbital: d_*z*^2^_ (black), d_*xz*_ (yellow), d_*xy*_ (blue), d_*yz*_ (green) and d_*x*^2^−y^2^_ (orange). Near-degenerate 3d_*xy*_ and 3d_*yz*_ orbitals are significantly mixed for 1-Ti. Bottom – corresponding singly occupied state-specific natural orbitals for 3-Ti with an isosurface of 0.04 e Å^−3^, labels indicate the majority d-orbital contribution to the orbital.

Complexes 1/3-Ti/Zr showed similar results, with the *n*d^1^ electrons located in their respective *n*d_*z*^2^_ orbitals. A d_*z*^2^_^1^ ground state is standard for bent metallocenes [M(Cp^R^)_2_X] and is consistent with our analysis of the ^91^Zr hyperfine coupling (see above).^88^ The excited states place d–d transitions for 1-Ti and 3-Ti between 16 000 and 21 000 cm^−1^ (Fig. 6, ESI Tables S14 and S15‡), in reasonable agreement with experiment (Fig. 3). For 3-Zr, the d–d transitions are calculated at higher energies (23 224, 25 402 and 30 058 cm^−1^, ESI Table S18‡) and are thus obscured by the CT band in agreement with experiment (Fig. 3).

The dimeric structure of 2-Zr results in the lowest energy d–d transition calculated for 2-Zr′ shifting from below 5000 cm^−1^ for 1-Ti, 3-Ti and 3-Zr to 14 031 cm^−1^, with the other d–d transitions at 18 101, 20 609 and 22 362 cm^−1^ (Fig. 6 and ESI Table S17‡); the former band corresponds well with the low energy shoulder at 13 100 cm^−1^ (*ε* = 300 M^−1^ cm^−1^; Fig. 3), while the latter three agree well with the broad absorption at 17 900 cm^−1^ (*ε* = 1060 M^−1^ cm^−1^; Fig. 3). There are no predicted d–d transitions in this region for 1-Zr, which has a low-energy band predicted at 7010 cm^−1^ and all other d–d transitions would be hidden beneath the CT band tailing from the UV region (ESI Table S16‡). These calculations agree with experimental data (see above) that both 1-Zr and 2-Zr are present in solution.

**Fig. 7:**
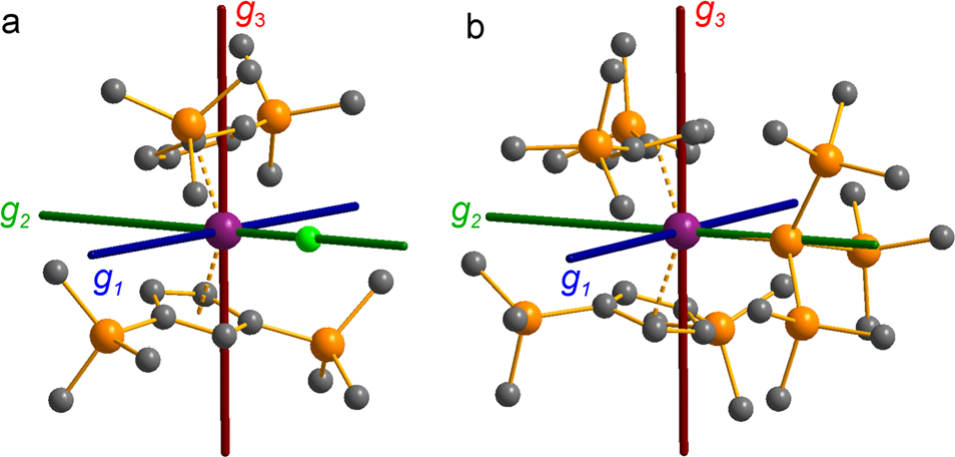
CASSCF-calculated magnetic axes (blue: *g*_1_, most magnetic; green: *g*_2_, intermediate; red: *g*_3_, least magnetic) for (a) 1-Ti and (b) 3-Ti. Transition metal, silicon, chlorine and carbon shown as purple, orange, green and grey respectively. Hydrogen atoms are excluded for clarity.

The pattern of calculated *g*-values for 1/3-Ti/Zr well-reproduces the experimental data (Table 3, ESI Tables S14–S16 and S18‡), while the absolute shifts in *g*_2_ and *g*_3_ are overestimated (ESI Table S11‡). The CASSCF calculations also give insight into the orientations of the *g*-values: for all 1/3-Ti/Zr, the largest value *g*_1_ is along the *z* direction (pseudo-three-fold), the intermediate value *g*_2_ is along *x* (M–Cl/Si bond), and the smallest value *g*_3_ is along *y* (tangential to Cp′′–M–Cp′′; Fig. 7 and ESI Fig. S134‡). While orbital energies do not strictly exist in a SA-CASSCF calculation, in this case the five states are each dominated by a single configuration with the unpaired electron located in one of the five d-orbitals, so approximate d-orbital energies can be determined by assigning the state energies to the energy of the singly occupied natural orbital for that state (Fig. 6, ESI Fig. S127, S128, S130–S132 and ESI Tables S14–S18‡).

We find that the d_*z*^2^_ orbital remains lowest in energy in all cases, followed by the d_*xz*_ orbital, which varies significantly in energy between complexes. The d_*xy*_ and d_*yz*_ orbitals lie far higher in energy (16 000–26 000 cm^−1^) and are relatively close in energy to each other, whilst the d_*x*^2^−y^2^_ orbital is highest in energy as chloride and hypersilanide are both strong σ-donor ligands. This is consistent with the Lauher-Hoffmann bonding model of a naked bent metallocene,^88,97^ with an additional monodentate ligand. With these orbital energies we can rationalise the observed *g*-values. Spin–orbit coupling along *z* cannot mix in any excited state into a ground state with the electron in the d_*z*^2^_ orbital,^92^ and so Δ*g*_*z*_ is approximately zero which agrees well with experiment (ESI Table S11‡; small deviations away from *g*_e_ are ascribed to mixing of d_*x*^2^−y^2^_ into the ground state^95^). Spin–orbit coupling along *x* (*y*) can mix in a state with an unpaired electron in d_*yz*_ (d_*xz*_) into the ground state to shift *g*_*x*_ (*g*_*y*_), where Δ*g*_*x*_ (Δ*g*_*y*_) is inversely proportional to the energy of the excited orbital (ESI eqn (S4) and (S5)‡). As d_*xz*_ is much lower in energy than d_*yz*_, Δ*g*_*y*_ is larger than Δ*g*_*x*_, and as the d shell is less than half-filled all Δ*g* are negative; hence *g*_3_ is along *y* and *g*_2_ is along *x*; this explains the obtained *ab initio* orientations (Fig. 7 and ESI Fig. S134‡).

The value of Δ*g*_3_ reflects the energy of the d_*xz*_ orbital and therefore the π-bonding character of the X ligand; this has been used previously to construct a π-donor spectrochemical series for [Ti(Cp*)_2_X].^88^ Between 1-Ti/Zr and 3-Ti/Zr Δ*g*_3_ doubles, reflecting the d_*xz*_ orbital being 40% lower in energy for 3-Ti/Zr; this is because chloride is a π-donor and d_*xz*_ is formally π-antibonding in 1-Ti/Zr (the nominal d_*xz*_ orbitals have 1.9% and 3.1% Cl 2p_*z*_ character for 1-Ti and 1-Zr, respectively; ESI Fig. S127 and S130‡). In contrast, hypersilanide is a weak π-acceptor, and such interactions can be seen with a very low isosurface value (ESI Fig. S129 and S133‡). Upon moving from Ti to Zr there is more effective overlap of the 4d and ligand orbitals, leading to a larger crystal field splitting (Fig. 6), and a decrease in the metal contribution to the singly occupied natural orbitals (ESI Tables S14–S16 and S18‡). Spin–orbit coupling also increases moving from Ti to Zr, which acts to increase |Δ*g*|, whilst increased d-orbital splitting and ligand–metal mixing oppose this; as all Δ*g* become more negative upon going from 1/3-Ti to 1/3-Zr, spin–orbit coupling is the dominant effect.

#### Cerium 4f^1^

**Fig. 8:**
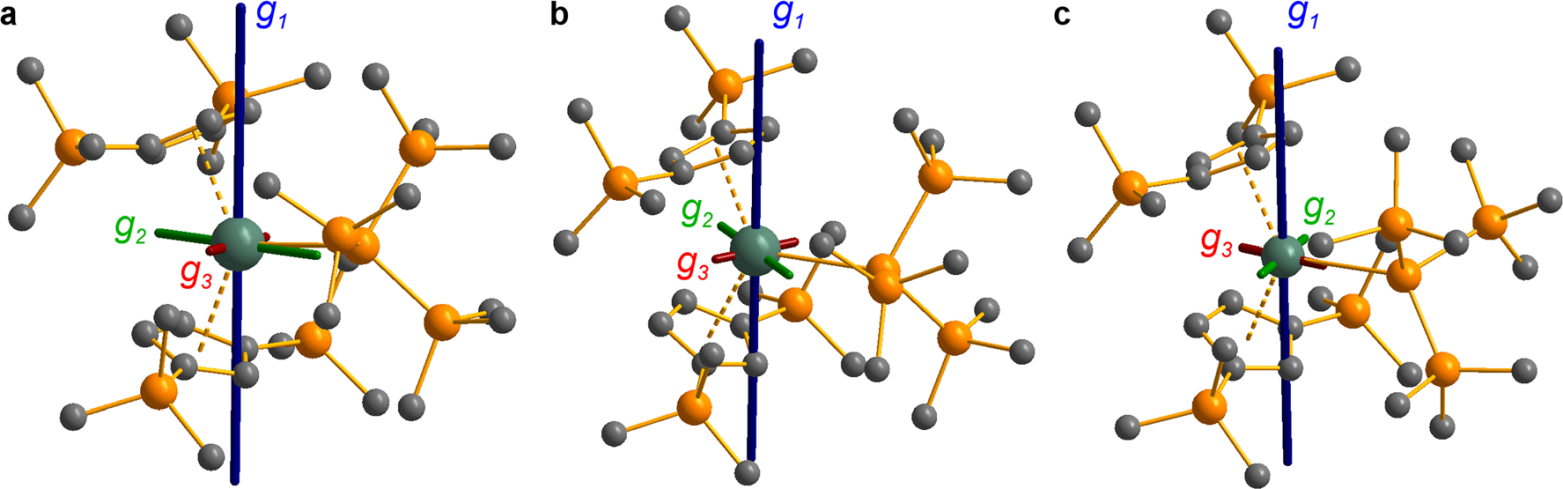
CASSCF-calculated magnetic axes (blue: *g*_1_, easy; green: *g*_2_, intermediate; red: *g*_3_, hard) for complexes 3-Ce (a), 3-Nd (b) and 3-U (c). Length of the magnetic axes reflects the size of the *g*-values. Metal, silicon and carbon shown as metallic green, orange and grey respectively. Hydrogen atoms are omitted for clarity.

For 3-Ce, a CAS(1,7)SCF calculation averaged over seven spin doublets was performed for an active space containing seven 4f orbitals. The resulting spin-free states were mixed with spin–orbit calculation to obtain the states of the ^2^F_5/2_ ground term (ESI Table S19‡). The ground state for 3-Ce is predicted to be 90% |±5/2〉 and 8% |±1/2〉, consistent with the slightly reduced *M*_sat_ and *g*_1_ values from those expected for a pure |±5/2〉 ground doublet; the calculated *g*-values are in good agreement with experiment (Table 3). Differing from 1/3-Ti/Zr, the easy axis (*g*_1_) is oriented tangential to the Cp′′–M–Cp′′ direction (*y*), with the intermediate axis (*g*_2_) along the Ce–Si bond (*x*) and the hard axis (*g*_3_) along *z* (Fig. 8a). The magnetic anisotropy of 4f ions is often dictated by pure electrostatic considerations,^98,99^ and so the dominance of a |±5/2〉 ground state (which has an oblate spheroidal 4f electron density) with its easy axis along *y* indicates the pair of sandwich-like Cp′′ ligands are stronger influences than the hypersilanide. This is similar to the situation in [CeCp^ttt^_2_Cl] (Cp^ttt^ = {C_5_H_2_*t*Bu_3_-1,2,4}), which has a ground state of 97% |±5/2〉 and 3% |∓1/2〉, compared to [CeCp^ttt^_2_{(C_6_F_5_-κ^1^-F)B(C_6_F_5_)_3_}], which has a 100% |±5/2〉 ground state.^78^ The larger extent of |±1/2〉 mixing in the ground state of 3-Ce compared to [CeCp^ttt^_2_Cl] (reflected also in the experimental *g*_1_-values of these two complexes: 3.884 *vs.* 4.19,^78^ respectively) argues for a stronger crystal field of hypersilanide over chloride; note that is the opposite of the discussion above for 1-Ti/Zr*vs.*3-Ti/Zr as the bonding interactions with 4f orbitals are irrelevant and the more diffuse chloride ligand provides a weaker electrostatic field.

#### Neodymium and uranium, *n*f^3^

The electronic structures of 3-Nd and 3-U were investigated by CAS(3,7)SCF calculations using an active space consisting of the seven *n*f orbitals and averaging over 35 spin quartets and 112 spin doublets (entire 5f^3^ configuration), and then mixing with spin–orbit coupling (Fig. 8b, c, ESI Tables S20 and S21‡). The crystal field splitting of the ^4^I_9/2_ ground term for 3-Nd gives a mixed ground doublet with 76% |±9/2〉 + 15% |±5/2〉 + 5% |±1/2〉, whose *g*-values match reasonably well with experiment (Table 3): the mixed ground state composition explains the lower *g*_1_ and *M*_sat_ values than predicted for a pure |±9/2〉 state. Despite the large mixing, the pattern of *g*-values appears more axial with smaller and similar *g*_2_ and *g*_3_ values. Like for 3-Ce, the easy axis (*g*_1_) is aligned tangentially to the Cp′′–M–Cp′′ direction (*y*), with *g*_2_ and *g*_3_ in the perpendicular *xz* plane (Fig. 8b). Also likewise to 3-Ce, the dominant |±9/2〉 ground state and the easy axis orientation are indicative of the Cp′′ ligands dominating the electrostatic potential as |±9/2〉 also has an oblate spheroidal 4f electron density.^98,99^

CASSCF calculations on 3-U averaging over all 5f^3^ states reproduce well the experimental susceptibility and *g*_1_ value, and suggest a 95% |±9/2〉 ground state. Like 3-Ce and 3-Nd, the oblate spheroidal electron density of |±9/2〉 in 3-U is orientated with *g*_1_ tangential to Cp′′–M–Cp′′ (Fig. 8c).^98,99^ However, this CASSCF calculation overestimates *M*_sat_; averaging instead over only the ^4^I_9/2_ ground term (13 spin quartets) gives a more accurate reproduction of M_sat_ but underestimates *g*_1_ and the magnetic susceptibility (ESI Fig. S99 and ESI Table S22‡), suggesting a slightly more mixed ground state of 89% |±9/2〉 + 6% |±5/2〉. The true ground state composition of 3-U is likely between these values, but it is certainly less mixed than that of 3-Nd.

The CAS(3,7)SCF averaged orbitals for 3-U (35 quartets and 112 doublets) showed small 6d orbital contributions (∼5%), so we extended the active space to also include two low-lying 6d orbitals (6d_*z*^2^_ and 6d_*xz*_, ESI Fig. S136,‡ in accordance with 3-Ti and 3-Zr, see above). Averaging over all f^3^ and f^2^d^1^ configurations in this active space, the f^2^d^1^ states lie at ∼7000 cm^−1^ (ESI Fig. S138‡), suggesting that spin- and Laporte-allowed f → d transitions contribute to the broad band from 6000 to 17 000 cm^−1^ in the UV-vis-NIR spectrum (Fig. 3). Examining the averaged molecular orbitals with a low isosurface value showed that the 5f orbitals participate in weak δ-antibonding and π-bonding with Cp′′, and weak π-bonding with low lying vacant orbitals on Si (ESI Fig. S137‡).

## Discussion

From the single crystal X-ray diffraction and DFT-optimised structural data it can be seen that the M–Si bonds are 0.26–0.30 Å longer than the corresponding M–Cl bonds in 1/3-Ti/Zr after correcting for the difference in single-bond covalent radii (Table 1).^55^ The steric bulk of the hypersilanide ligand imposes the longer M–Si bonds, which leads to weaker M–Si bonding interactions through reduced orbital overlap. The bonding in the {M(Cp′′)_2_}^+^ fragments are expected to be similar in 1-M*vs.*3-M. Whilst the orientation of Cp′′ rings changes, the key 
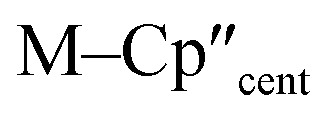
 distances and 
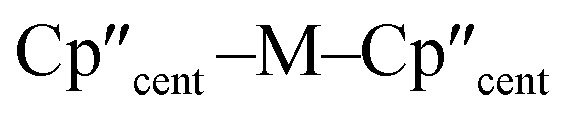
 angles for Zr(iii) are virtually unchanged, and for Ti(iii) there are only modest respective increases of these metrics of 0.014 Å and 2.6°.

The 3d^1^ and 4d^1^ systems 3-Ti and 3-Zr exhibit *n*d_*z*^2^_^1^ ground state occupancies, with spin density perpendicular to the 
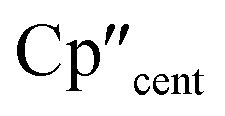
, 
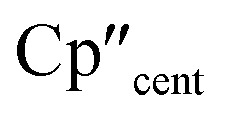
, Si plane. The *n*d_*z*^2^_^1^ ground state is non-bonding and is favoured by strong anti-bonding interactions of the remaining *n*d-orbitals with the two Cp′′ ligands as well as with the hypersilanide ligand, which acts as a σ-donor. The hypersilanide ligand also acts as a weak π-acceptor, giving rise to a low energy *n*d_*xz*_^1^ excited state in both cases (<10 000 cm^−1^). The stabilisation of *n*d_*z*^2^_ and *n*d_*xz*_ orbitals is echoed in 3-U, where low energy f^3^ → f^2^d^1^ transitions are seen above 6000 cm^−1^. However, for 3-Ce and 3-Nd the f^*n*^ → f^*n*−1^d^1^ transitions are not proximate to the ground states, as expected.

Due to the orbitally non-degenerate ground state, the anisotropy of the *g*-tensors in 3-Ti and 3-Zr are dominated by second-order spin–orbit coupling with the *n*d_*xz*_^1^ excited state. This results in a large shift of *g*_*y*_ away from *g*_e_, where *y* is tangential to Cp′′–M–Cp′′. In f-block complexes with near-complete f-orbital degeneracy, anisotropy in the ground state is dictated by electrostatics, and we find that the bis-Cp′′ crystal field dominates over the hypersilanide contribution. This favours oblate f-electron density in the ground state, giving ground states dominated by |±5/2〉, |±9/2〉 and |±9/2〉, for 3-Ce, 3-Nd and 3-U, respectively, with the magnetic easy axis tangential to Cp′′–M–Cp′′. These data indicate that the M–Si bonds in 3-Ti and 3-Zr are both more covalent than the M–Si bonds in 3-Ln and 3-U, whilst the greater covalency of the M–Si bonds in 3-Zr*vs.*3-Ti is also evident from the respective magnitudes of 4d *vs.* 3d crystal field splitting.

The presence of slow magnetic relaxation for 3-U and the lack of such behaviour for 3-Nd is an outlier compared to literature examples, where analogous f^3^ compounds tend to show more similar behaviour.^75,100^ It is likely that faster magnetic relaxation occurs for 3-Nd due to the lower purity of the ground state, which itself is likely to arise due to mixing with low energy spin–orbit states at 65 and 176 cm^−1^: the more-pure ground state in 3-U on the other hand, is well-separated from the lowest excited states at 330 and 524 cm^−1^, which likely arises from a larger crystal field effect due to 5f *vs.* 4f orbitals. This is in accord with the UV-vis-NIR spectrum of 3-U, where the f → d transitions are low energy for U(iii),^53^ and are a hallmark of polarised covalent metal–ligand bonding.^15^ The greater involvement of 5f *vs.* 4f orbitals in M–Si bonds was also seen in *ab initio* calculations. It follows that the M–Si bond in 3-U has greater covalency than that of 3-Nd, with the M–Si bonds of 3-La, 3-Ce and 3-Nd assumed to show similar predominantly electrostatic character due to their valence 4f orbitals.

## Conclusions

We have reported the synthesis and characterisation of a series of isostructural early d- and f-block M(iii) bis(cyclopentadienyl) hypersilanide complexes, providing the first structurally authenticated examples of U(iii) and Nd(iii) silanides. By using a combination of CW EPR spectroscopy and CASSCF calculations we have shown that the d-block complexes herein have 3/4d_*z*^2^_^1^ ground states aligned perpendicular to the coordination plane, with the hypersilanide ligand acting as a strong σ-donor and weak π-acceptor to impart axial anisotropy through π-bonding with low-lying *n*d_*xz*_ orbitals; as expected, the orbital splitting is greater for 4d_*z*^2^_^1^ Zr(iii) *vs.* 3d_*z*^2^_^1^ Ti(iii). In contrast, the early f-block Ln/U(iii) silanide 4/5f^*n*^ complexes exhibit predominantly ionic bonding, with the dominant crystal field imparted by the Cp′′ ligands favouring oblate spheroidal f-electron densities and magnetic easy axes tangential to Cp′′–M–Cp′′.

The uranium silanide complex was found to exhibit increased covalency over Ln congeners, with calculations showing weak π-bonding between the 5f orbitals and both the silanide and Cp′′ ligands, and weak δ-antibonding between 5f orbitals and Cp′′. The greater crystal field imposed for 5f^3^ U(iii) *vs.* 4f^3^ Nd(iii) gave a purer ground state due to the energies of low-lying excited states being raised to the extent that they can no longer mix, switching on slow magnetic relaxation in the former complex below 5 K. The U(iii) congener additionally displayed low energy 5f^3^ → 5f^2^6d^1^ electronic transitions from 6000 to 17 000 cm^−1^, signifying that the *n*d_*z*^2^_ and *n*d_*xz*_ orbitals have been stabilised in a similar manner to Ti(iii) and Zr(iii) homologues. Together, the combination of data acquired herein show the qualitative ordering of the extent of covalency to be Zr > Ti ≫ U > Nd ≈ Ce ≈ La, and reveal clear differences between the compositions of early d-block, Ln and An M–Si bonds.

## Data availability

Research data files supporting this publication are available from FigShare at: https://doi.org/10.6084/m9.figshare.20459439.

## Author contributions

B. L. L. R., S. T. L. and D. P. M. provided the original concept. B. L. L. R. synthesised and characterised the compounds and solved and refined the crystal structures. G. K. G. collected and interpreted EPR and magnetic data and performed calculations. A. J. W. further refined the crystallographic data and finalised CIFs. J. E.-K. carried out supporting synthetic and characterisation work. N. F. C. supervised the EPR, magnetism and calculations components. D. P. M. and S. T. L. supervised the synthetic component and directed the research. B. L. L. R., G. K. G., D. P. M., S. T. L. and N. F. C. wrote the manuscript, with contributions from all authors.

## Conflicts of interest

There are no conflicts to declare.

## Supplementary Material

SC-014-D2SC04526E-s001

SC-014-D2SC04526E-s002
