# Supplementary material for: Electronic structure comparisons of isostructural early d- and f-block metal(iii) bis(cyclopentadienyl) silanide complexes
Source: Chem Sci. 2022 Dec 6;14(3):621–34. doi: 10.1039/d2sc04526e (PMC9847655; doi:10.1039/d2sc04526e)
Supplement: SC-014-D2SC04526E-s001 [file SC-014-D2SC04526E-s001.pdf]

Electronic Supplementary Material (ESI) for Chemical Science.
This journal is © The Royal Society of Chemistry 2022

- Electronic Supporting Information -

Electronic Structure Comparisons of Isostructural Early d- and f-Block Metal(III) Bis(cyclopentadienyl) Silanide Complexes

Gemma K. Gransbury,[‡] Benjamin L. L. Réant,[‡] Ashley J. Wooles, Jack Emerson-King, Nicholas F. Chilton,* Stephen T. Liddle,* and David P. Mills*

Department of Chemistry, The University of Manchester, Oxford Road, Manchester, M13 9PL, U.K.

Contents

1. General Procedures S2
2. Experimental Details S4
3. NMR Spectroscopy S14
4. Evans Method Magnetic Susceptibility NMR Spectra S34
5. ATR-IR Spectroscopy S39
6. UV-Vis-NIR Spectroscopy S45
7. General Crystallographic Methods S53
8. Crystallographic Data S54
9. Molecular Structures S57
10. Static SQUID Magnetometry S61
11. Dynamic SQUID Magnetometry on **3-U** S74
12. EPR Spectroscopy S81
13. Density Functional Theory (DFT) Calculations S99
14. Complete Active Space Self-Consistent Field (CASSCF) Calculations S101
15. References S120

1. General Procedures

All manipulations were conducted under argon with the strict exclusion of oxygen and water by using Schlenk line and glove box techniques. THF, Et_2O, toluene and pentane were purged with ultra-high-purity grade argon (Airgas) and passed through columns containing alumina catalyst and molecular sieves before use. Hexane was dried by refluxing over potassium and stored over a potassium mirror, then degassed before use. For NMR spectroscopy, C_6D_6 was dried by refluxing over K, and was vacuum transferred and degassed by three freeze-pump-thaw cycles before use. NMR solution samples were prepared at 0 °C in J Young tap-appended NMR tubes and warmed immediately before transfer to the spectrometer, with the ^{1}H NMR spectra collected within 10 mins of dissolution. NMR spectra were recorded on a Bruker AVIII HD 400 spectrometer operating at 400.07 (^{1}H), 100.60 (^{13}C) or 79.48 (^{29}Si) MHz; all spectra were referenced to tetramethylsilane. ATR-IR spectra of microcrystalline powders were collected on a Bruker Alpha spectrometer with Platinum-ATR module. UV-Vis-NIR solution samples were prepared at 0 °C in toluene (2 mM) in screw top 1 mm path length cuvettes and were warmed immediately before data collection. UV-Vis-NIR spectra were recorded on a Perkin Elmer Lambda 750 spectrometer over *ca*. 5 mins, using toluene as a blank. Elemental analysis was carried out by Mr Martin Jennings and Mrs Anne Davies at the Microanalytical service, Department of Chemistry, the University of Manchester. Results generally showed good agreement with expected compositions. Although low carbon values were consistently obtained for **3-Ce** and **3-Zr**, we previously made similar observations for other f-block silanide complexes and attributed this to silicon carbide formation;[1,2] this phenomenon has previously been commented upon by others.[3] $[ZrCl_4(THF)_2]$ was purchased from Merck and was used without further purification. $[HfCl_4(THF)_2]$,[4] $[ThCl_4(DME)_2]$,[5] UCl_4,[6] $[TiCl_3(THF)_3]$,[7] $[MI_3(THF)_x]$ (M = La, Ce, x = 4; M = Nd, x = 3.5),[8] $[UI_3(THF)_4]$,[9] KC_8,[10] KCp″,[11] LiCp″,[12,13] $[K\{Si(SiMe_3)_3\}]$,[14] $[Zr(Cp'')_2Cl_2]$,[15] $[M(Cp'')_2Cl_2]$ (M = Th, U),[16]

[Ti(Cp″)$_2$Cl] (**1-Ti**),[17] [{Zr(Cp″)$_2$(μ-Cl)}$_2$] (**2-Zr**),[18] [{La(Cp″)$_2$(μ-I)}$_2$] (**2-La**),[19] [{Ce(Cp″)$_2$(μ-I)}$_2$] (**2-Ce**),[19] were prepared according to literature procedures. Synthetic procedures and analytical details for [M(Cp″)$_2$Cl$_2$] (M = Zr, Hf, Th, U), **1-Ti**, **2-Zr**, **2-La**, **2-Ce** are provided below. *Caution: Natural abundance thorium and uranium are weak α-emitters, thus we recommend the use of suitable designated radiochemical laboratories with α-counting equipment available for safe manipulation of compounds containing these elements*.

2. Experimental Details

2.1. Synthesis of [M(Cp″)$_2$Cl$_2$] (M = Zr, Hf, Th, U)

General procedure for the synthesis of series [M(Cp″)$_2$Cl$_2$]. A Schlenk flask was charged with [MCl$_4$(sol)$_x$] (M = Zr, Hf, sol = THF, x = 2; M = U, sol = none) and LiCp″ (2 eq.) and cooled to –78 °C. Et$_2$O (20 mL/mmol) was added and the white (M = Zr, Hf, Th) or red-brown (M = U) suspension was warmed to room temperature and stirred for 18 hours. All volatiles were subsequently removed *in vacuo*, and the solids were extracted with hot hexane (20 mL/ mmol, 50 °C). Upon cooling, needles of [M(Cp″)$_2$Cl$_2$] formed, which were isolated at room temperature. Further crops of [M(Cp″)$_2$Cl$_2$] were obtained on storage of the concentrated mother liquor at –25 °C.

Preparation of [Zr(Cp″)$_2$Cl$_2$]. Prepared according to the general procedure and adapting literature protocols[15] with [ZrCl$_4$(THF)$_2$] (1.886 g, 5 mmol) and LiCp″ (2.164 g, 10 mmol); [Zr(Cp″)$_2$Cl$_2$] was obtained as colourless needles (1.835 g, 3.16 mmol, 63 %). All analytical data are consistent with literature values.[15] ^{29}Si{^{1}H} NMR (79.48 MHz, C$_6$D$_6$, 298 K): δ = –6.81 (Cp-*Si*CH$_3$). FTIR ν/ cm^{-1}: 2956 (w), 2898 (w), 1448 (w), 1409 (w), 1244 (m), 1088 (m), 918 (m), 817 (s), 752 (m), 698 (m), 639 (m), 465 (m).

Preparation of [Hf(Cp″)$_2$Cl$_2$]. Prepared according to the general procedure and adapting literature protocols[15] with [HfCl$_4$(THF)$_2$] (2.323 g, 5 mmol) and LiCp″ (2.164 g, 10 mmol); [Hf(Cp″)$_2$Cl$_2$] was obtained as colourless needles (2.732 g, 4.10 mmol, 82 %). Anal. Calcd for C$_{22}$H$_{42}$Cl$_2$HfSi$_4$: C, 39.54; H, 6.33. Found: C, 39.28; H, 6.33. ^{1}H NMR (400.07 MHz, C$_6$D$_6$, 298 K): δ = 0.34 (s, 36H, Cp-SiC*H*$_3$), 6.38 (d, $^3J_{HH}$ = 1.8 Hz, 4H, Cp-4,5-*H*), 7.10 (t, $^3J_{HH}$ = 1.8 Hz, 2H, Cp-2-*H*). ^{13}C{^{1}H} NMR (100.60 MHz, C$_6$D$_6$, 298 K): δ = 0.29 (Cp-Si*C*H$_3$), 119.48 (Cp-4,5-*C*H), 126.99 (Cp-*C*), 142.34 (Cp-2-*C*H). ^{29}Si{^{1}H} NMR (79.48 MHz, C$_6$D$_6$, 298 K):

δ = –6.97 (Cp-*Si*CH_3). FTIR ν/ cm^{-1}: 2956 (w), 2897 (w), 1447 (w), 1409 (w), 1254 (m), 1088 (m), 922 (m), 821 (s), 756 (m), 635 (m), 470 (m).

Preparation of [Th(Cp″)$_2$Cl$_2$]. Prepared according to the general procedure and adapting literature protocols[16] with [$ThCl_4$(DME)$_2$] (2.770 g, 5 mmol) and LiCp″ (2.164 g, 10 mmol); [Th(Cp″)$_2$Cl$_2$] was obtained as colourless needles (2.635 g, 3.65 mmol, 73 %). All analytical data are consistent with literature values.[16] ^{29}Si{^{1}H} NMR (79.48 MHz, C_6D_6, 298 K): δ = –8.83 (Cp-*Si*CH_3).

Preparation of [U(Cp″)$_2$Cl$_2$]. Prepared according to the general procedure and adapting literature protocols[16] with UCl_4 (1.580 g, 5 mmol) and LiCp″ (2.164 g, 10 mmol); [U(Cp″)$_2$Cl$_2$] was obtained as red needles (1.877 g, 2.58 mmol, 52 %). All analytical data are consistent with literature values.[16] ^{29}Si{^{1}H} NMR (79.48 MHz, C_6D_6, 298 K): δ = –53.74 (Cp-*Si*CH_3).

2.2. Synthesis of 1-Ti and 2-Zr

Preparation of [Ti(Cp″)$_2$Cl] (1-Ti). [Synthesis modified from literature procedure[17]]. A Schlenk flask was charged with [$TiCl_3$(THF)$_3$] (1.853 g, 5 mmol) and LiCp″ (2.164 g, 10 mmol) and cooled to –78 °C. Et_2O (100 mL) was added, and the turquoise suspension was warmed to room temperature to gradually become purple over a one hour period. All volatiles were subsequently removed *in vacuo* after stirring for 18 hours, and the solids were extracted with hexane (120 mL). Concentration and storage of the filtrate at –25 °C led to the formation of purple needles of [Ti(Cp″)$_2$Cl] (**1-Ti**, 2.011 g, 4.00 mmol, 80 %). Anal. Calcd for $C_{22}H_{42}ClSi_4Ti$: C, 52.61; H, 8.43. Found: C, 53.15; H, 8.74. The ^{1}H NMR spectrum could not be interpreted, and no resonances attributed to **1-Ti** were observed by ^{13}C{^{1}H} and ^{29}Si{^{1}H} NMR spectroscopy due to paramagnetic broadening. Evans Method (C_6D_6, 298 K) 1.63 μ_B.

FTIR ν/ cm^{-1}: 2956 (m), 2897 (w), 1448 (w), 1409 (w), 1254 (s), 1088 (m), 922 (m), 821 (s), 748 (s), 694 (m), 635 (s), 477 (m).

Preparation of [{Zr(Cp″)$_2$(μ-Cl)}$_2$] (2-Zr). [Synthesis modified from literature procedure[18]] A THF (25 mL) solution of [Zr(Cp″)$_2$Cl$_2$] (1.743 g, 3 mmol) was added to a pre-cooled (–78 °C) suspension of KC_8 (0.426 g, 3.15 mmol) in THF (10 mL). The dark blue suspension was warmed to room temperature and stirred for one hour. Volatiles were removed *in vacuo* and the residue was extracted with toluene (100 mL). Concentration and storage of the filtrate at –25 °C led to the formation of dark blue blocks of [{Zr(Cp″)$_2$(μ-Cl)}$_2$] (**2-Zr**, 1.017 g, 0.93 mmol, 62 %). Anal. Calcd for $C_{44}H_{84}Cl_2Si_8Zr_2$: C, 48.43; H, 7.76. Found: C, 48.53; H, 7.67. ^{1}H NMR (400.07 MHz, C_6D_6, 298 K): δ = 0.33 (s, 72H, Cp-SiC*H*$_3$), 6.45 (d, $^3J_{HH}$ = 2.3 Hz, 8H, Cp-4,5-*H*), 7.21 (s, 4H, Cp-2-*H*). ^{13}C{^{1}H} NMR (100.60 MHz, C_6D_6, 298 K): δ = 0.23 (Cp-Si*C*H$_3$), 120.32 (Cp-4,5-*C*H), 129.09 (Cp-*C*), 143.38 (Cp-2-*C*H). ^{29}Si{^{1}H} NMR (79.48 MHz, C_6D_6, 298 K): δ = –6.81 (Cp-*Si*CH$_3$). Evans Method (C_6D_6, 298 K) 1.43 μ_B. FTIR ν/ cm^{-1}: 2952 (m), 2894 (w), 1444 (w), 1405 (w), 1247 (s), 1084 (m), 922 (m), 821 (s), 751 (s), 694 (s), 632 (s), 473 (m). UV-Vis-NIR $\acute{\upsilon}_{max}$/ cm^{-1}: 13,100sh (ε = 300 M^{-1} cm^{-1}), 17,900 (ε = 1,060 M^{-1} cm^{-1}).

2.3. Synthesis of 2-M (M = La, Ce, Nd, U)

General procedure for the synthesis of 2-M. A Schlenk flask was charged with [MI$_3$(THF)$_x$] (M = La, Ce, U, x = 4; M = Nd, x = 3.5) and KCp″ (2 eq.) and cooled to –78 °C. THF (10 mL/mmol) was added and the cream (M = La), yellow (M = Ce), sky blue (M = Nd), or green (M = U) suspension was warmed to room temperature and stirred for 18 hours. All volatiles were subsequently removed *in vacuo*, and the solids were extracted with toluene (20

mL/mmol). Concentration and storage of the filtrate at –25 °C led to the formation of needles of [M(Cp″)$_2$(μ-I)}$_2$] (**2-M**).

Preparation of [{La(Cp″)$_2$(μ-I)}$_2$] (2-La). Prepared according to the general procedure and adapting literature protocols[19] with [$LaI_3(THF)_4$] (4.040 g, 5 mmol) and KCp″ (2.486 g, 10 mmol); **2-La** was obtained as colourless blocks (1.570 g, 1.15 mmol, 46 %). All analytical data are consistent with literature values.[19]

Preparation of [{Ce(Cp″)$_2$(μ-I)}$_2$] (2-Ce). Prepared according to the general procedure and adapting literature protocols[19] with [$CeI_3(THF)_4$] (4.046 g, 5 mmol) and KCp″ (2.486 g, 10 mmol); **2-Ce** was obtained as pink blocks (2.867 g, 2.09 mmol, 84 %). All analytical data are consistent with literature values.[19] $^{13}C\{^1H\}$ NMR (100.60 MHz, C_6D_6, 298 K): δ = –0.53 (Cp-Si*C*H$_3$). Resonances associated with Cp-*C* and Cp-*C*H were not observed due to paramagnetic broadening. $^{29}Si\{^1H\}$ NMR (79.48 MHz, C_6D_6, 298 K): δ = –20.55 (Cp-*Si*CH$_3$). Evans Method (C_6D_6, 298 K) 3.30 μ_B. UV-Vis-NIR $\acute{\upsilon}_{max}$/ cm^{-1}: 19,700 (ε = 920 M^{-1} cm^{-1}).

Preparation of [{Nd(Cp″)$_2$(μ-I)}$_2$] (2-Nd). Prepared according to the general procedure with [$NdI_3(THF)_{3.5}$] (3.887 g, 5 mmol) and KCp″ (2.486 g, 10 mmol); **2-Nd** was obtained as blue blocks (2.113 g, 1.53 mmol, 61 %). Anal. Calcd for $C_{44}H_{84}I_2Nd_2Si_8$: C, 38.29; H, 6.14. Found: C, 38.26; H, 6.20. ^1H NMR (400.07 MHz, C_6D_6, 298 K): δ = –4.80 (s, 72H, Cp-SiC*H*$_3$), Resonances associated with Cp-C*H* were not observed due to paramagnetic broadening. $^{13}C\{^1H\}$ NMR (100.60 MHz, C_6D_6, 298 K): δ = 4.09 (Cp-Si*C*H$_3$). Resonances associated with Cp-*C* and Cp-*C*H were not observed due to paramagnetic broadening. $^{29}Si\{^1H\}$ NMR (79.48 MHz, C_6D_6, 298 K): δ = –58.62 (Cp-*Si*CH$_3$). Evans Method (C_6D_6, 298 K) 5.49 μ_B. FTIR ν/ cm^{-1}: 2956 (w), 2894 (w), 1440 (w), 1401 (w), 1242 (m), 1080 (m), 918 (m), 829 (s), 786 (s),

749 (s), 690 (m), 635 (m), 620 (m), 473 (m). UV-Vis-NIR $\acute{\upsilon}_{max}$/ cm^{-1}: 16,800 (ε = 330 M^{-1} cm^{-1}, $^4I_{9/2} \rightarrow {}^4G_{5/2}$).

Preparation of [{U(Cp″)$_2$(μ-I)}$_2$] (2-U). Prepared according to the general procedure with [UI$_3$(THF)$_4$] (4.536 g, 5 mmol) and KCp″ (2.486 g, 10 mmol); **2-U** was obtained as green blocks (2.628 g, 1.68 mmol, 67 %). Anal. Calcd for $C_{44}H_{84}I_2Si_8U_2$: C, 33.71; H, 5.40. Found: C, 33.27; H, 5.28. ^{1}H NMR (400.07 MHz, C_6D_6, 298 K): δ = –7.15 (s, 72H, Cp-SiC*H*$_3$), Resonances associated with Cp-C*H* were not observed due to paramagnetic broadening. ^{13}C{^{1}H} NMR (100.60 MHz, C_6D_6, 298 K): δ = –13.58 (Cp-Si*C*H$_3$). Resonances associated with Cp-*C* and Cp-*C*H were not observed due to paramagnetic broadening. ^{29}Si{^{1}H} NMR (79.48 MHz, C_6D_6, 298 K): not observed due to paramagnetic broadening. Evans Method (C_6D_6, 298 K) 4.17 μ_B. FTIR ν/ cm^{-1}: 2952 (w), 2898 (w), 1436 (w), 1405 (w), 1251 (m), 1077 (m), 926 (m), 825 (s), 751 (s), 693 (m), 635 (m), 624 (m), 469 (m). UV-Vis-NIR $\acute{\upsilon}_{max}$/ cm^{-1}: 6,000–16,000 (ε = 290–1,080 M^{-1} cm^{-1}).

2.4. Synthesis of 3-M (M = Ti, Zr, La, Ce, Nd, U)

General procedure for the synthesis of series 3-M. [K{Si(SiMe$_3$)$_3$}] (1 eq. for n = 1, 2 eq. for n = 2) was dissolved in toluene (10 mL/mmol) and added dropwise to a solution of **1-M** or **2-M** in toluene (15 mL/mmol) cooled to –78 °C. The purple (M = Ti), dark red (M = Zr), orange (M = La), red (M = Ce, Nd), or green (M = U) solution was warmed to room temperature and stirred for 1 hour. All volatiles were subsequently removed *in vacuo*, and the solids were extracted with pentane (40 mL/mmol). Concentration and storage of the filtrate at –25 °C led to the formation of needles of [M(Cp″)$_2${Si(SiMe$_3$)$_3$}] (**3-M**).

Preparation of [Ti(Cp″)$_2${Si(SiMe$_3$)$_3$}] (3-Ti). Prepared according to the general procedure with **1-Ti** (0.502 g, 1 mmol) and [K{Si(SiMe$_3$)$_3$}] (0.287 g, 1 mmol); **3-Ti** was obtained as purple needles (0.245 g, 0.34 mmol, 34 %). Anal. Calcd for $C_{31}H_{69}Si_8Ti$: C, 52.12; H, 9.74. Found: C, 52.11; H, 9.91. ^{1}H NMR (400.07 MHz, C_6D_6, 298 K): δ = 0.14 (s, 36H, Cp-SiC*H*$_3$), 1.60 (s, 27H, Si(SiC*H*$_3$)$_3$). Resonances associated with Cp-C*H* were not observed due to paramagnetic broadening. No resonances attributed to **3-Ti** were observed by ^{13}C{^{1}H} and ^{29}Si{^{1}H} NMR spectroscopy due to paramagnetic broadening. Evans Method (C_6D_6, 298 K) 1.93 μ_B. FTIR ν/ cm^{-1}: 2952 (m), 2894 (w), 1440 (w), 1409 (w), 1250 (m), 1084 (m), 922 (m), 821 (s), 752 (s), 674 (m), 636 (m), 620 (s), 481 (m), 431 (m). UV-Vis-NIR $\acute{\upsilon}_{max}$/ cm^{-1}: 18,300 (ε = 760 M^{-1} cm^{-1}).

Preparation of [Zr(Cp″)$_2${Si(SiMe$_3$)$_3$}] (3-Zr). Prepared according to the general procedure with **2-Zr** (0.818 g, 0.75 mmol) and [K{Si(SiMe$_3$)$_3$}] (0.430 g, 1.5 mmol); **3-Zr** was obtained as dark red needles (0.455 g, 0.60 mmol, 40 %). Anal. Calcd for $C_{31}H_{69}Si_8Zr$: C, 49.13; H, 9.18. Found: C, 48.47; H, 9.14. ^{1}H NMR (400.07 MHz, C_6D_6, 298 K): δ = 0.88 (s, 36H, Cp-SiC*H*$_3$), 2.97 (s, 27H, Si(SiC*H*$_3$)$_3$). No Cp-*H* resonances were seen in the ^{1}H NMR spectrum and no resonances attributed to **3-Zr** were observed by ^{13}C{^{1}H} and ^{29}Si{^{1}H} NMR spectroscopy due to paramagnetic broadening. Evans Method (C_6D_6, 298 K) 1.71 μ_B. FTIR ν/ cm^{-1}: 2948 (m), 2894 (w), 1436 (w), 1394 (w), 1247 (m), 1081 (m), 919 (m), 825 (s), 752 (s), 686 (m), 635 (m), 620 (m), 473 (m). UV-Vis-NIR $\acute{\upsilon}_{max}$/ cm^{-1}: 22,400 (ε = 890 M^{-1} cm^{-1}).

Preparation of [La(Cp″)$_2${Si(SiMe$_3$)$_3$}] (3-La). Prepared according to the general procedure with **2-La** (1.027 g, 0.75 mmol) and [K{Si(SiMe$_3$)$_3$}] (0.430 g, 1.5 mmol); **3-La** was obtained as orange needles (0.758 g, 0.95 mmol, 63 %). Anal. Calcd for $C_{31}H_{69}LaSi_8$: C, 46.23; H, 8.63. Found: C, 46.02; H, 8.85. ^{1}H NMR (400.07 MHz, C_6D_6, 298 K): δ = 0.25 (s, 36H, Cp-SiC*H*$_3$),

0.55 (s, 27H, Si(SiCH_3)$_3$), 7.17 (s, 4H, Cp-4,5-*H*), 7.46 (s, 2H, Cp-2-*H*). ^{13}C{^{1}H} NMR (100.60 MHz, C_6D_6, 298 K): δ = 0.32 (Cp-Si*C*H$_3$), 6.39 (Si(Si*C*H$_3$)$_3$), 126.29 (Cp-4,5-*C*H), 129.57 (Cp-2-*C*H), 132.98 (Cp-*C*). ^{29}Si{^{1}H} NMR (79.48 MHz, C_6D_6, 298 K): δ = –10.32 (Cp-*Si*CH$_3$), –6.57 (Si(*Si*CH$_3$)$_3$), La*Si* resonance not observed due to quadrupolar broadening. FTIR ν/ cm^{-1}: 2948 (m), 2894 (w), 1429 (w), 1402 (w), 1255 (m), 1235 (m), 1076 (m), 918 (m), 817 (s), 744 (m), 671 (m), 617 (m), 473 (m). UV-Vis-NIR $\acute{\upsilon}_{max}$/ cm^{-1}: 22,200 (ε = 1,880 M^{-1} cm^{-1}).

Preparation of [Ce(Cp″)$_2${Si(SiMe$_3$)$_3$}] (3-Ce). Prepared according to the general procedure with **2-Ce** (1.029 g, 0.75 mmol) and [K{Si(SiMe$_3$)$_3$}] (0.430 g, 1.5 mmol); **3-Ce** was obtained as red needles (0.762 g, 0.95 mmol, 63 %). Anal. Calcd for $C_{31}H_{69}CeSi_8$: C, 46.16; H, 8.62. Found: C, 44.06; H, 8.52. ^{1}H NMR (400.07 MHz, C_6D_6, 298 K): δ = –8.33 (s, 36H, Cp-SiCH_3), –1.11 (s, 27H, Si(SiCH_3)$_3$). Resonances associated with Cp-C*H* were not observed due to paramagnetic broadening. ^{13}C{^{1}H} NMR (100.60 MHz, C_6D_6, 298 K): δ = 8.39 (Si(Si*C*H$_3$)$_3$). Resonances associated with Cp-Si*C*H$_3$, Cp-*C* and Cp-*C*H were not observed due to paramagnetic broadening. ^{29}Si{^{1}H} NMR (79.48 MHz, C_6D_6, 298 K): not observed due to paramagnetic broadening. Evans Method (C_6D_6, 298 K) 2.34 μ_B. FTIR ν/ cm^{-1}: 2956 (m), 2894 (w), 1436 (m), 1405 (w), 1250 (m), 1081 (m), 1023 (m), 922 (m), 814 (s), 748 (s), 674 (m), 616 (m), 473 (m). UV-Vis-NIR $\acute{\upsilon}_{max}$/ cm^{-1}: 23,500 (ε = 1,200 M^{-1} cm^{-1}).

Preparation of [Nd(Cp″)$_2${Si(SiMe$_3$)$_3$}] (3-Nd). Prepared according to the general procedure with **2-Nd** (1.035 g, 0.75 mmol) and [K{Si(SiMe$_3$)$_3$}] (0.430 g, 1.5 mmol); **3-Nd** was obtained as red needles (0.938 g, 1.16 mmol, 77 %). Anal. Calcd for $C_{31}H_{69}NdSi_8$: C, 45.92; H, 8.58. Found: C, 45.78; H, 8.75. ^{1}H NMR (400.07 MHz, C_6D_6, 298 K): δ = –11.64 (s, 36H, Cp-SiCH_3), –2.02 (s, 27H, Si(SiCH_3)$_3$). Resonances associated with Cp-C*H* were not observed due to paramagnetic broadening. ^{13}C{^{1}H} NMR (100.60 MHz, C_6D_6, 298 K): δ = 19.79

(Si(SiCH_3)$_3$). Cp-SiCH_3, Cp-*C* and Cp-*C*H resonances were not observed due to paramagnetic broadening. ^{29}Si{^{1}H} NMR (79.48 MHz, C_6D_6, 298 K): δ = –22.18. Evans Method (C_6D_6, 298 K) 3.54 μ_B. FTIR ν/ cm^{-1}: 2952 (m), 2890 (w), 1436 (w), 1247 (m), 1076 (m), 919 (m), 821 (s), 744 (m), 671 (m), 616 (m), 473 (m). UV-Vis-NIR $\acute{\upsilon}_{max}$/ cm^{-1}: 24,000 (ε = 1,570 M^{-1} cm^{-1}), 16,800 (ε = 260 M^{-1} cm^{-1}, $^4I_{9/2} \rightarrow {}^4G_{5/2}$).

Preparation of [U(Cp″)$_2${Si(SiMe$_3$)$_3$}] (3-U). *Method A)* Prepared according to the general procedure with **2-U** (1.176 g, 0.75 mmol) and [K{Si(SiMe$_3$)$_3$}] (0.430 g, 1.5 mmol); **3-U** was obtained as green needles (0.961 g, 1.06 mmol, 71 %). *Method B)* **3-U** can alternatively be prepared directly from [U(Cp″)$_2$Cl$_2$] (0.728 g, 1 mmol) using two eq. of [K{Si(SiMe$_3$)$_3$}] (0.574 g, 2 mmol) with a synthetic procedure identical to the general procedure for series **3-M**, giving **3-U** as green needles (0.696 g, 0.77 mmol, 77 %); Anal. Calcd for $C_{31}H_{69}Si_8U$: C, 41.16; H, 7.69. Found: C, 41.09; H, 7.82. ^{1}H NMR (400.07 MHz, C_6D_6, 298 K): δ = –14.79 (s, 36H, Cp-SiC*H*$_3$), –5.90 (s, 27H, Si(SiC*H*$_3$)$_3$). Resonances associated with Cp-C*H* were not observed due to paramagnetic broadening. ^{13}C{^{1}H} NMR (100.60 MHz, C_6D_6, 298 K): δ = –28.48 (Cp-Si*C*H$_3$), 17.11 (Si(Si*C*H$_3$)$_3$). Cp-*C* and Cp-*C*H resonances were not observed due to paramagnetic broadening. ^{29}Si{^{1}H} NMR (79.48 MHz, C_6D_6, 298 K): not observed due to paramagnetic broadening. Evans Method (C_6D_6, 298 K) 3.16 μ_B. FTIR ν/ cm^{-1}: 2948 (m), 2890 (w), 1432 (w), 1235 (m), 1072 (m), 918 (m), 817 (s), 744 (m), 671 (m), 620 (m), 470 (m). UV-Vis-NIR $\acute{\upsilon}_{max}$/ cm^{-1}: 24,200 (ε = 2,160 M^{-1} cm^{-1}), 6,000–16,000 (ε = 200–500 M^{-1} cm^{-1}).

2.5. Synthesis of MCp″ (M = Li, K) salts

Notes. Here we have included adapted synthetic procedures of LiCp″ and KCp″ from dicyclopentadiene; these are previously reported syntheses across multiple steps.[11–13,20–22]

Upon NMR spectroscopic analysis, in $CDCl_3$, of HCp′ and HCp″ the ratio of HCp^R:THF should be determined to ensure the correct quantity of ^nBuLi or KH is added.

HCp′. [Synthesis modified from literature procedures[20,22]] A solution of *n*-BuLi in hexanes (2.5 M, 65 ml, 166 mmol) was added dropwise over a 30 minute period to a cold (0 °C) solution of freshly cracked CpH (11.544 g, 175 mmol) in THF (120 ml). The resulting suspension of a white solid was allowed to warm to room temperature and stirred for three hours. The reaction mixture was cooled to 0 °C and trimethylsilyl chloride (21 ml, 166 mmol) was added dropwise. The resulting suspension of a white solid was allowed to warm to room temperature and stirred for 18 hours. The solvent was removed under reduced pressure (initially 300 to 100 mBar) to give an oily white solid. The product was isolated by distillation from this solid under reduced pressure (10 mBar, oil bath 100 °C) to give $HCp'\cdot(THF)_{0.875}$ (30.575 g, 118 mmol, 71%) as a colourless oil.

HCp″. [Synthesis modified from literature procedures[21,22]] A solution of *n*-BuLi in hexanes (2.5 M, 47 ml, 118 mmol) was added dropwise to a cold (0 °C) solution of HCp′ (16.310 g, 118 mmol) in THF (120 ml). The resulting pale yellow solution was allowed to warm to room temperature and stirred for three hours. The reaction mixture was cooled to 0 °C and trimethylsilyl chloride (15 ml, 118 mmol) was added. The resulting suspension of a white solid was allowed to warm to room temperature and stirred for 18 hours. The solvent was removed under reduced pressure (initially 300 to 100 mBar) to give an oily white solid. The product was isolated by distillation from this solid under reduced pressure (0.05 mbar, oil bath 80 °C) to give $HCp''\cdot(THF)_{0.188}$ (27.563 g, 110 mmol, 94%) as a colourless oil.

LiCp″. [Synthesis modified from literature procedures[12,13,20–22]] A solution of *n*-BuLi in hexanes (2.5 M, 19.5 ml, 48 mmol) was added dropwise to a cold (0 °C) solution of HCp″ (9.654 g, 46 mmol) in THF (80 mL). The resulting pale yellow solution was warmed to room temperature and stirred for 18 hours. Volatiles were removed *in vacuo* to give a white solid, which was washed with pentane (2 x 100 mL) and dried *in vacuo* to afford a free-flowing white powder of LiCp″ (8.872 g, 41 mmol, 90 %).

KCp″. [Synthesis modified from literature procedures[11,21,22]] A solution of HCp″ (11.944 g, 56.7 mmol) in THF (100 mL) was added dropwise to a cold (0 °C) suspension of KH (2.162 g, 53.9 mmol). The resulting colourless solution was warmed to room temperature and stirred for 18 hours. Volatiles were removed *in vacuo* to give a white solid, which was thoroughly washed with pentane (2 x 100 mL) and dried *in vacuo* to afford a free-flowing white powder of KCp″ (9.244 g, 37 mmol, 69 %).

3. NMR Spectroscopy

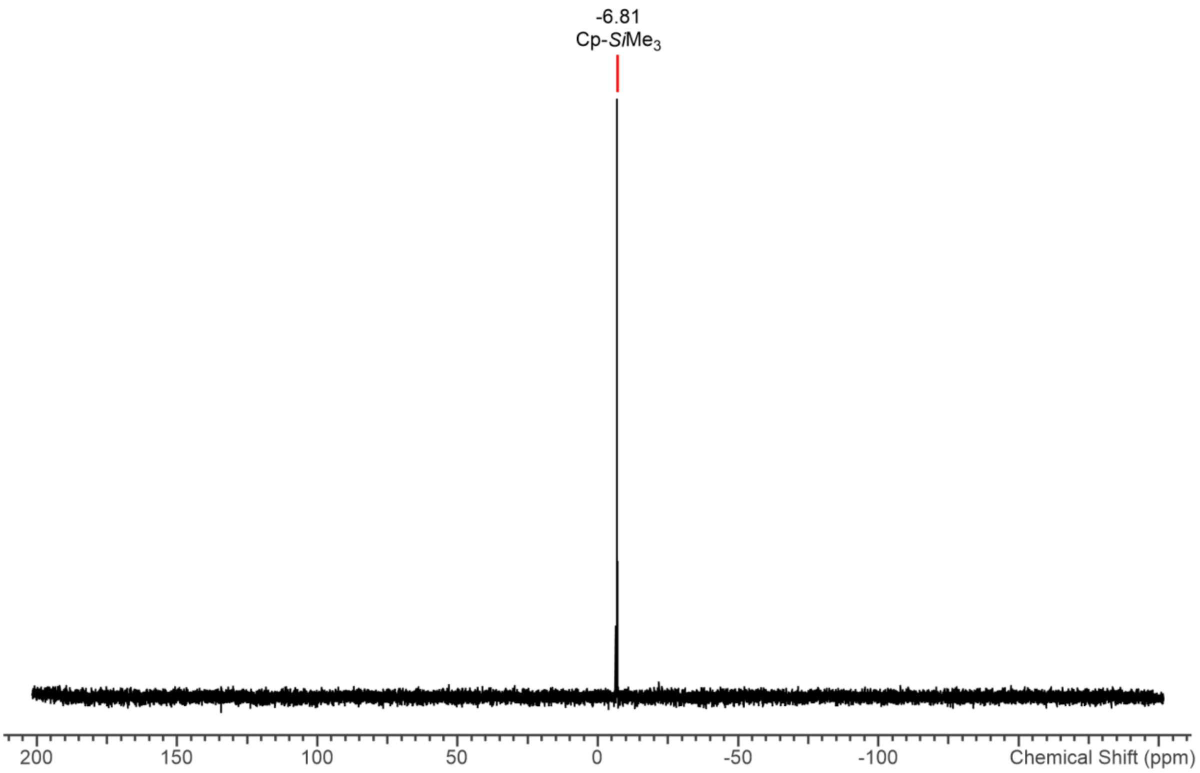

Figure S1. $^{29}Si\{^{1}H\}$ NMR spectrum (79.48 MHz) of [Zr(Cp″)$_2$Cl$_2$] in C_6D_6.

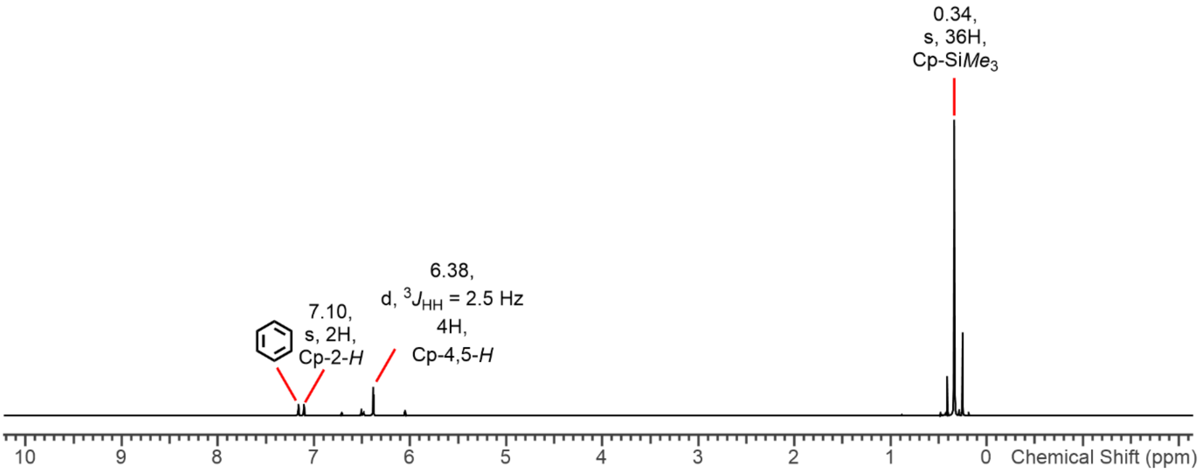

Figure S2. ^{1}H NMR spectrum (400.07 MHz) of [Hf(Cp″)$_2$Cl$_2$] in C_6D_6.

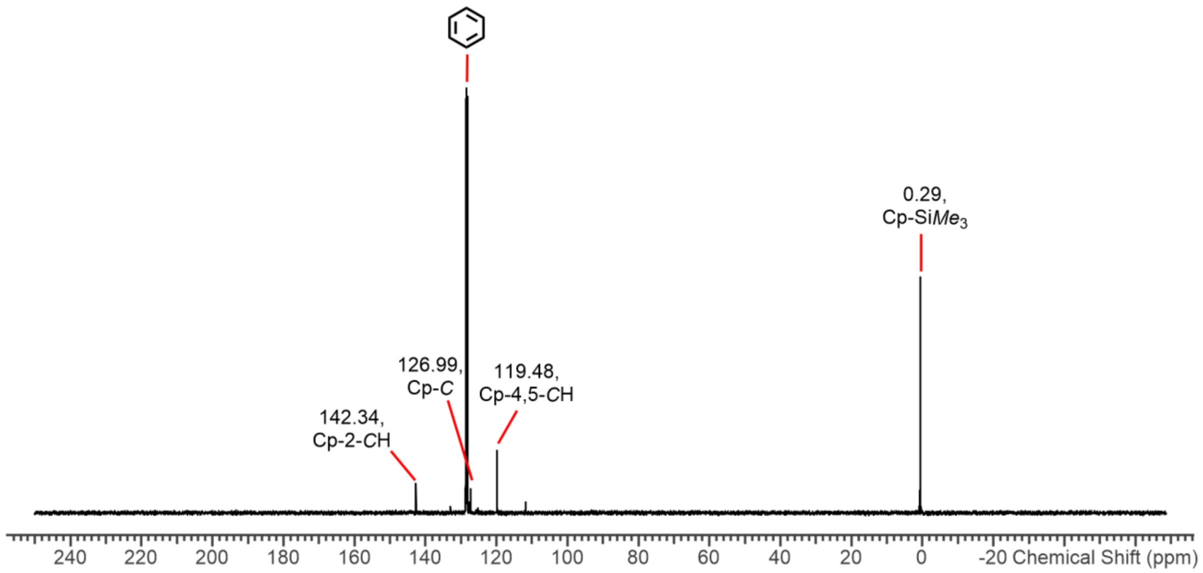

Figure S3. $^{13}C\{^1H\}$ NMR spectrum (100.60 MHz) of $[Hf(Cp'')_2Cl_2]$ in C_6D_6.

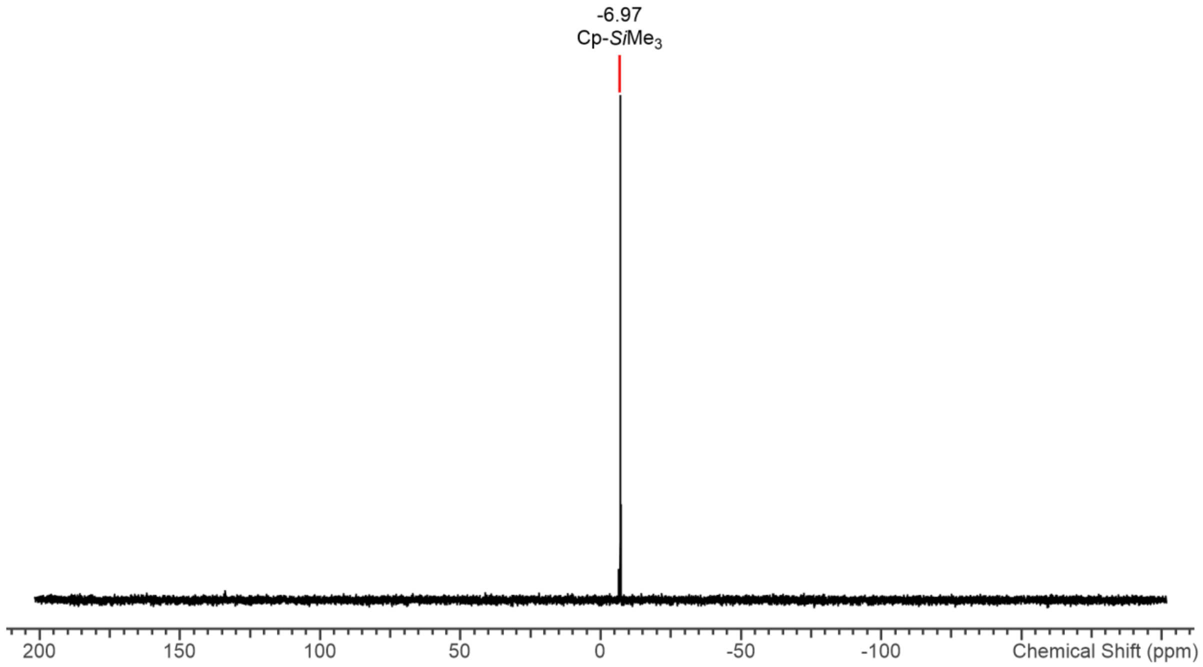

Figure S4. $^{29}Si\{^1H\}$ NMR spectrum (79.48 MHz) of $[Hf(Cp'')_2Cl_2]$ in C_6D_6.

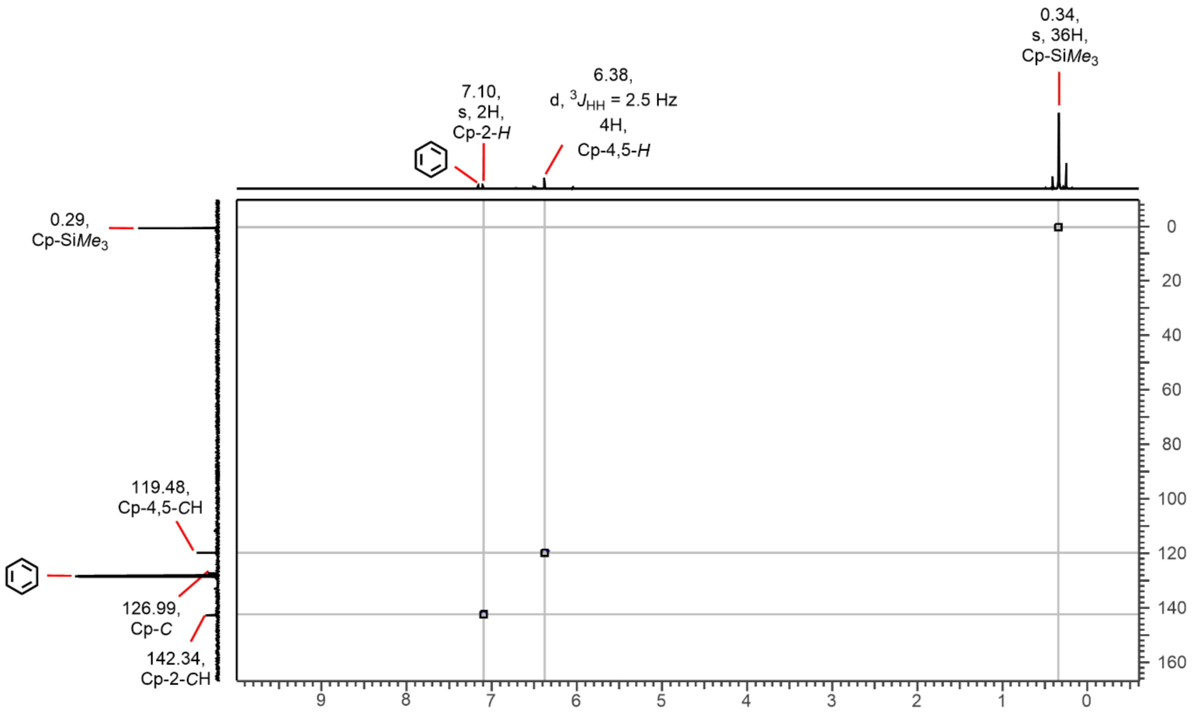

Figure S5. ^{1}H-^{13}C HSQC NMR spectrum of [Hf(Cp″)$_2$Cl$_2$] in C_6D_6.

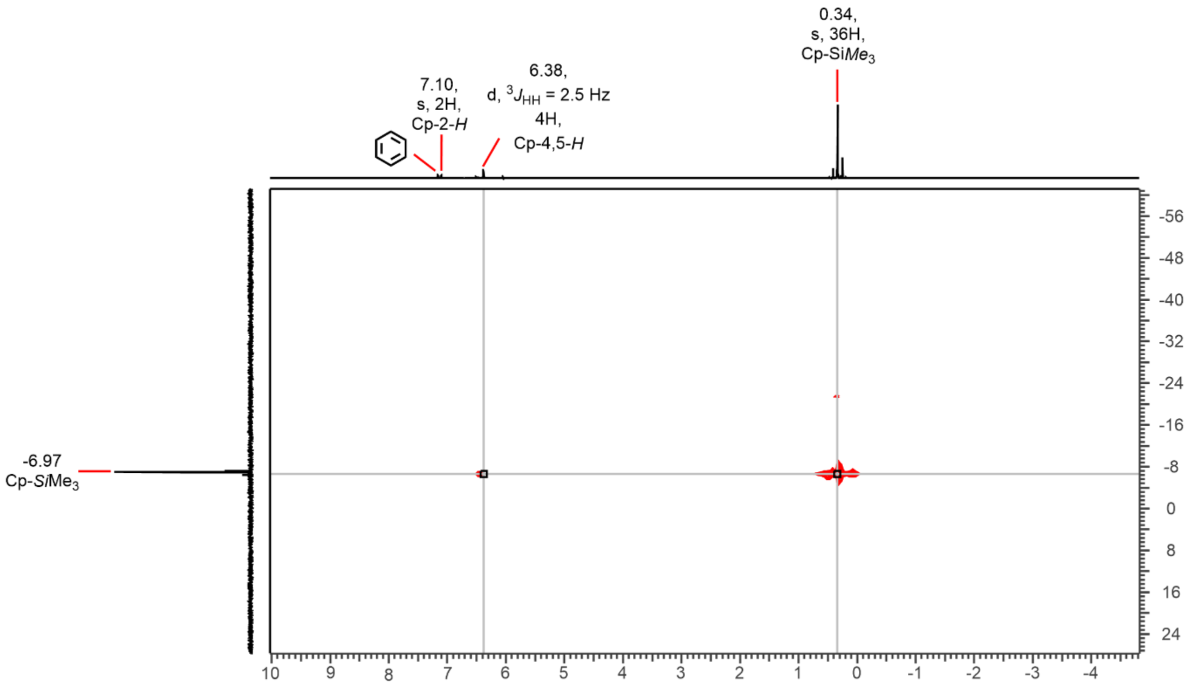

Figure S6. ^{1}H-^{29}Si HMBC NMR spectrum of [Hf(Cp″)$_2$Cl$_2$] in C_6D_6.

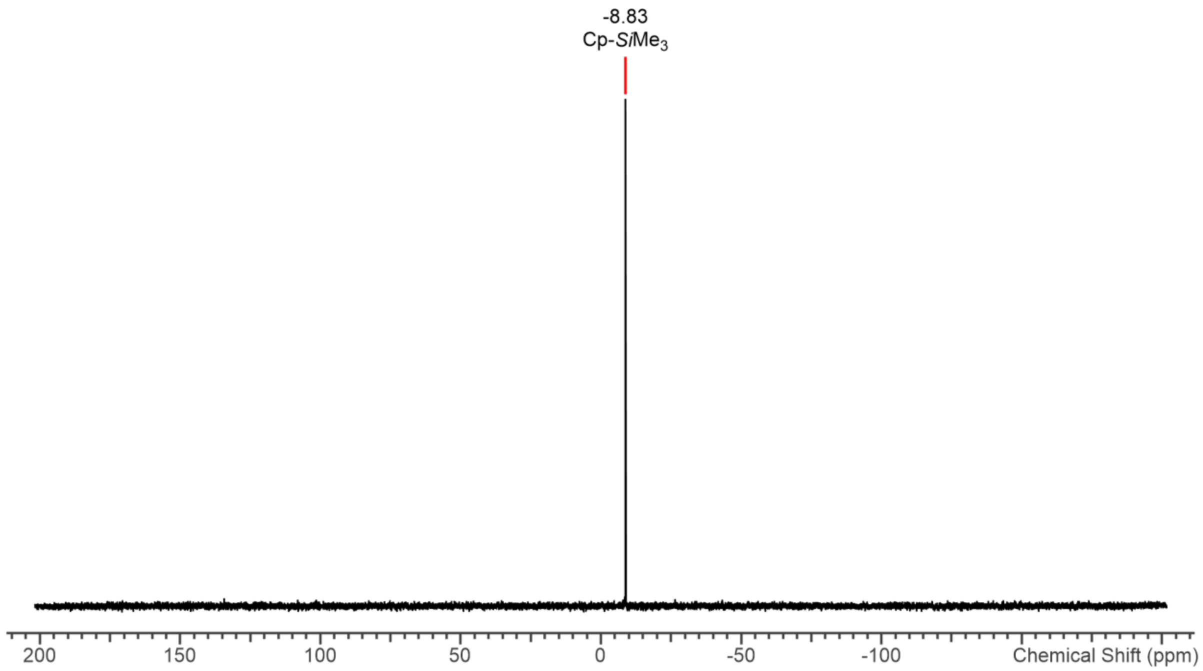

Figure S7. ^{29}Si{^{1}H} NMR spectrum (79.48 MHz) of [Th(Cp″)$_2$Cl$_2$] in C_6D_6.

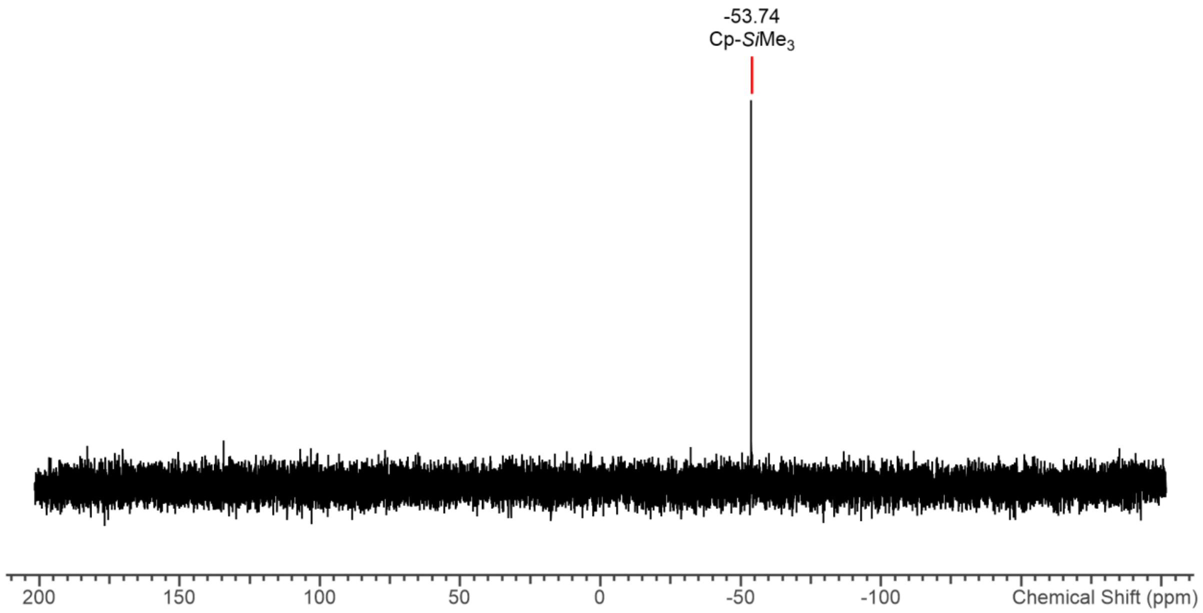

Figure S8. ^{29}Si{^{1}H} NMR spectrum (79.48 MHz) of [U(Cp″)$_2$Cl$_2$] in C_6D_6.

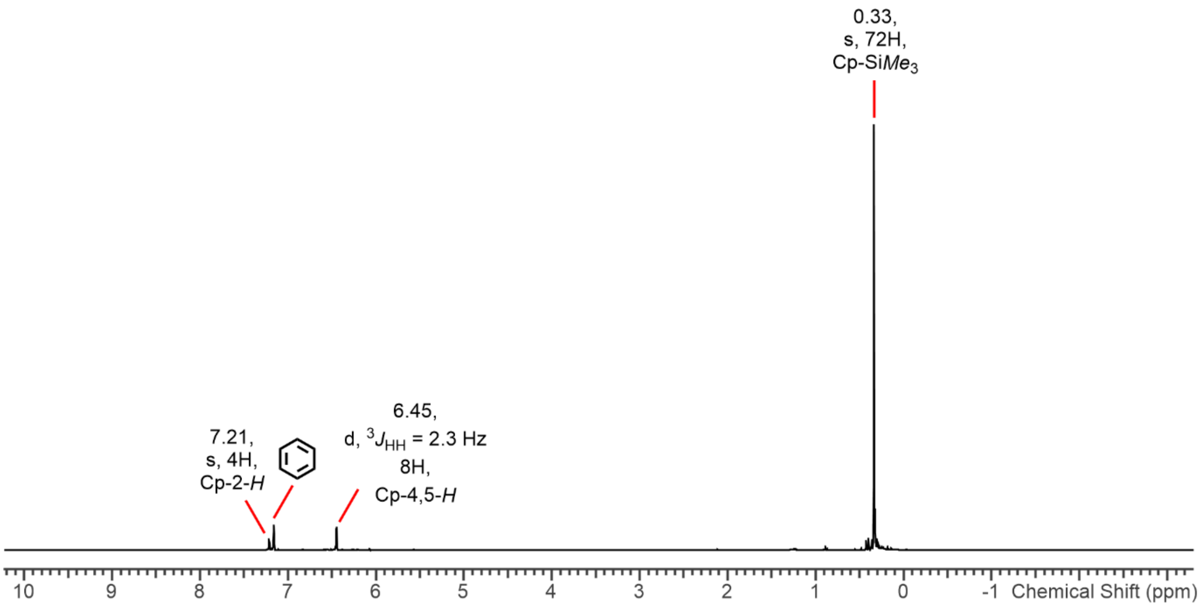

Figure S9. ^{1}H NMR spectrum (400.07 MHz) of **2-Zr** in C_6D_6.

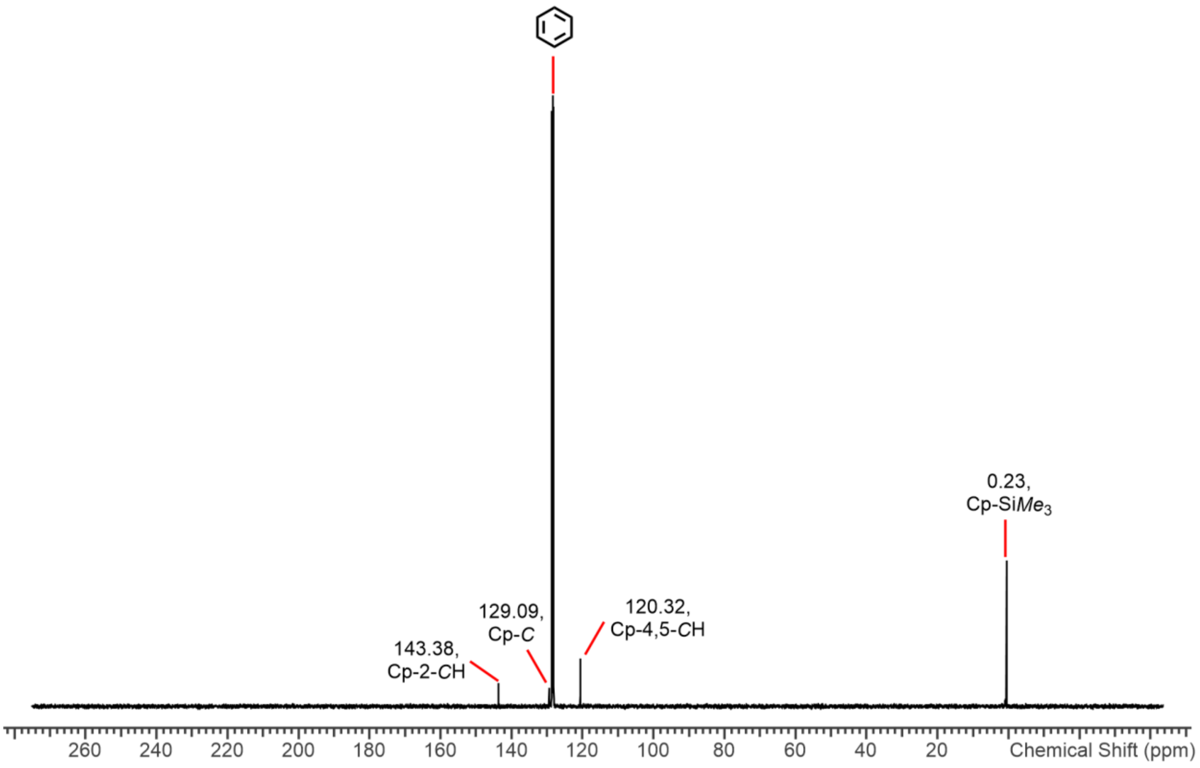

Figure S10. ^{13}C{^{1}H} NMR spectrum (100.60 MHz) of **2-Zr** in C_6D_6.

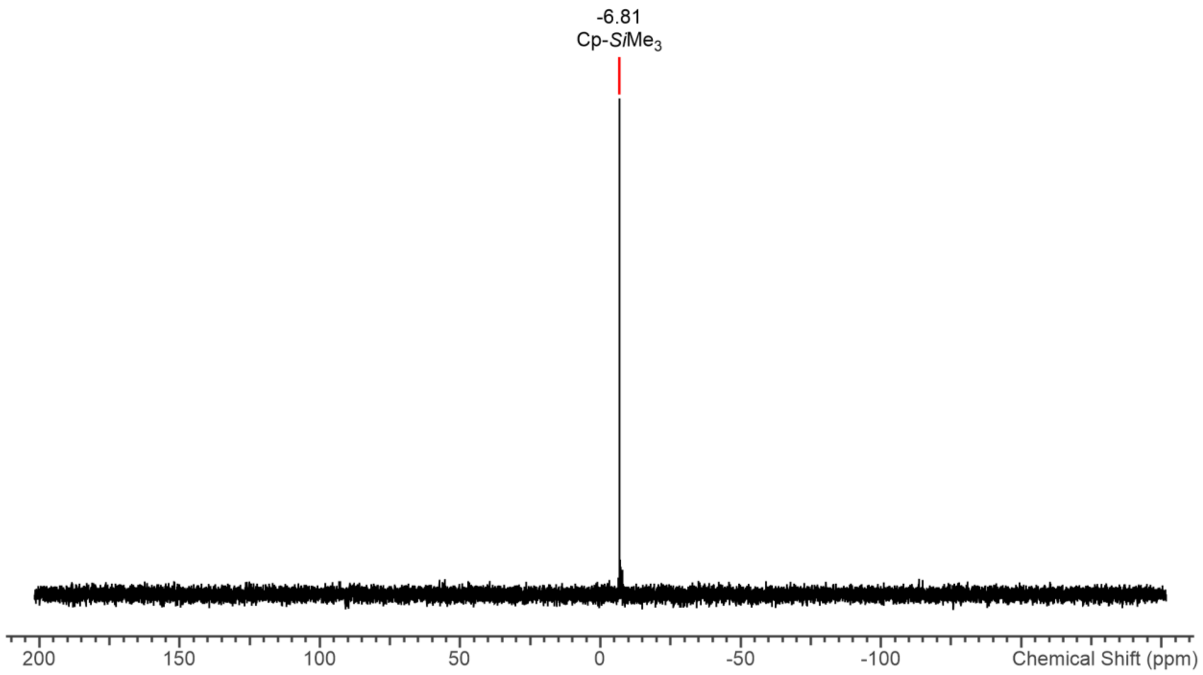

Figure S11. $^{29}Si\{^{1}H\}$ NMR spectrum (79.48 MHz) of **2-Zr** in C_6D_6.

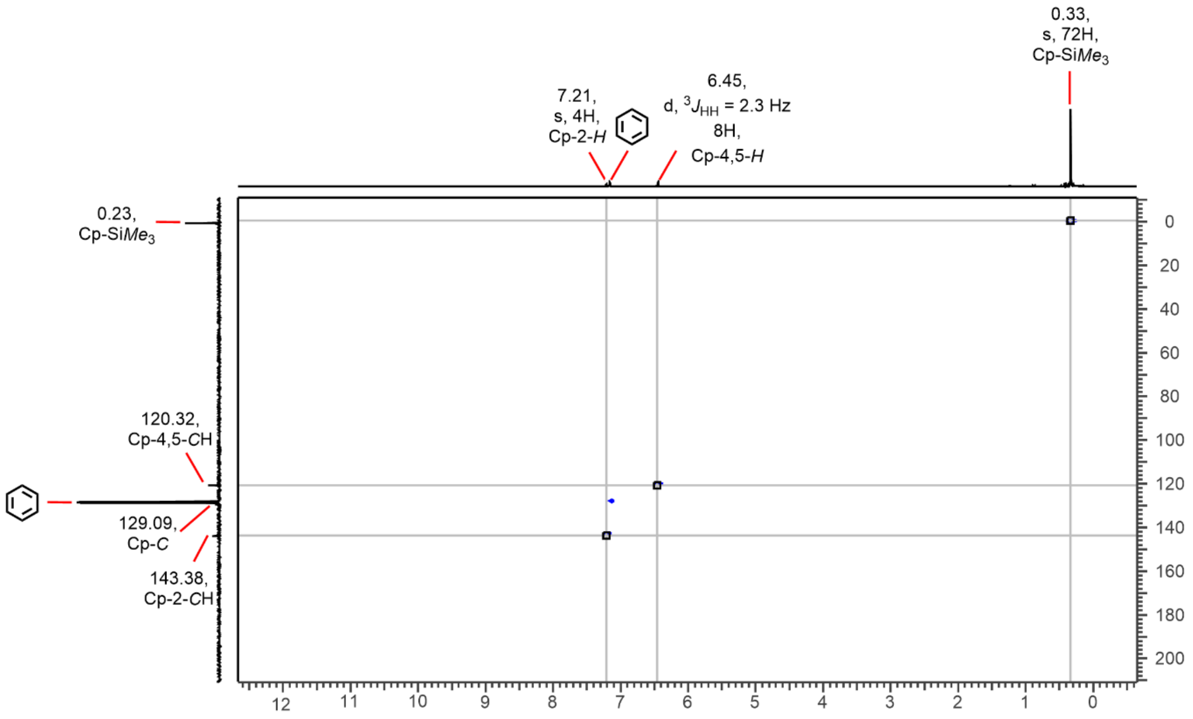

Figure S12. ^{1}H-^{13}C HSQC NMR spectrum of **2-Zr** in C_6D_6.

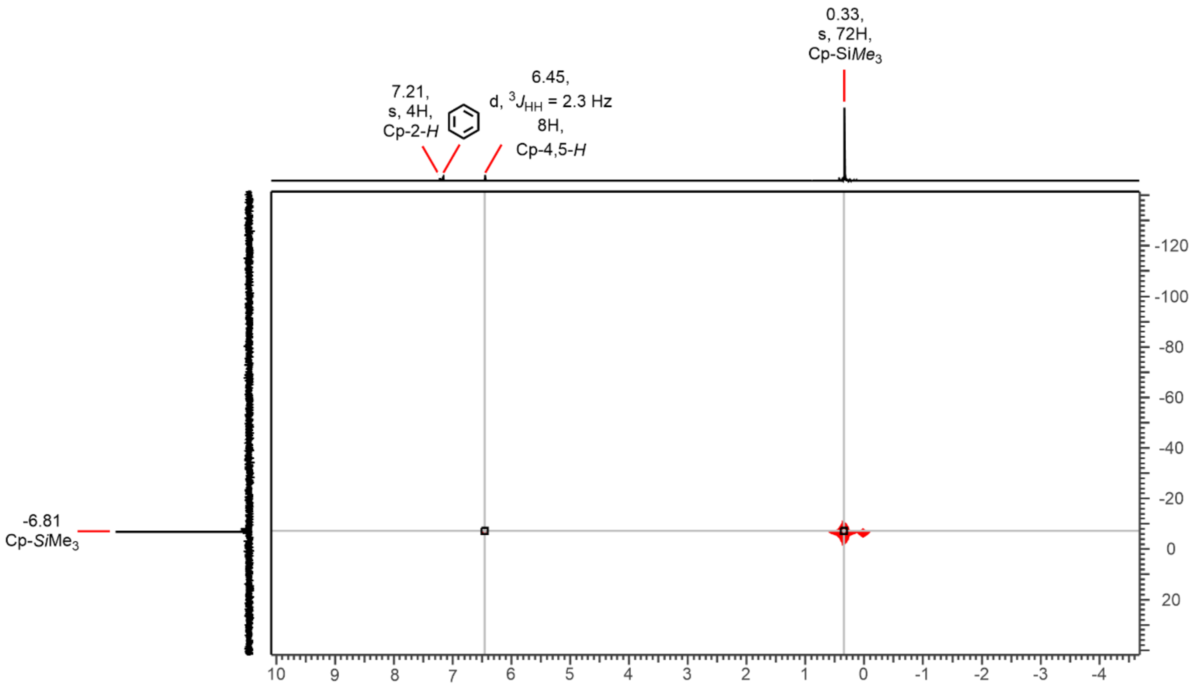

Figure S13. ^{1}H-^{29}Si HMBC NMR spectrum of **2-Zr** in C_6D_6.

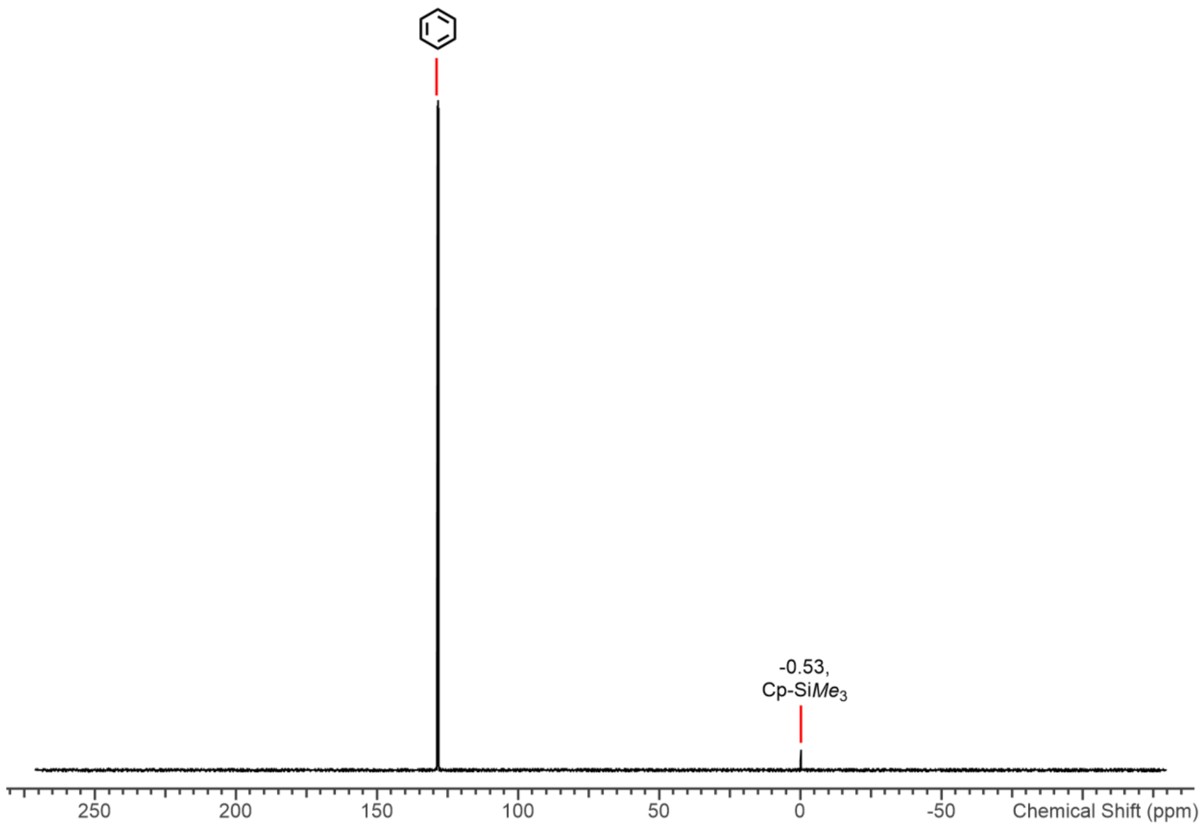

Figure S14. ^{13}C{^{1}H} NMR spectrum (100.60 MHz) of **2-Ce** in C_6D_6.

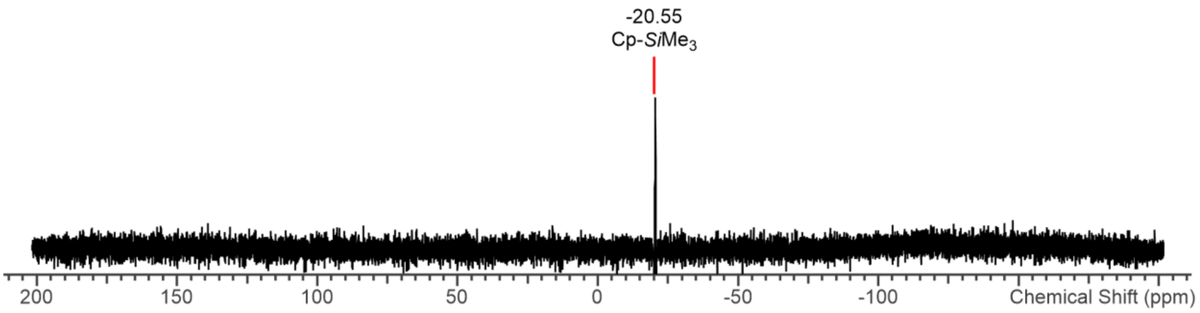

Figure S15. $^{29}Si\{^{1}H\}$ NMR spectrum (79.48 MHz) of **2-Ce** in C_6D_6.

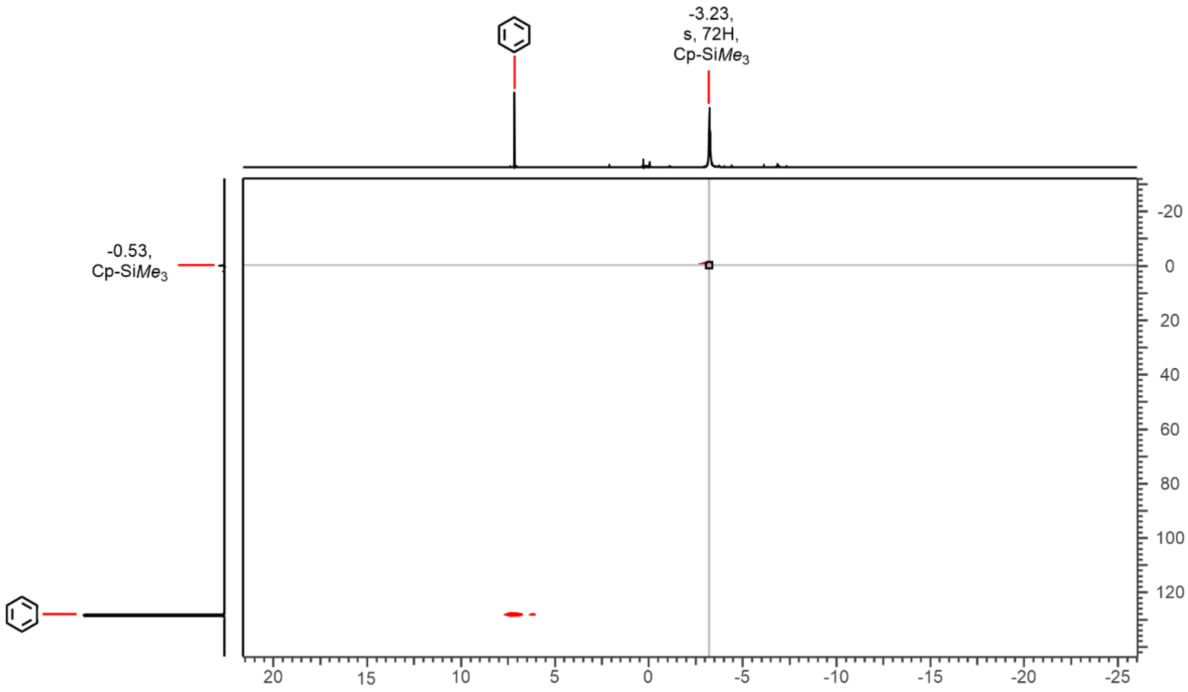

Figure S16. ^{1}H-^{13}C HSQC NMR spectrum of **2-Ce** in C_6D_6.

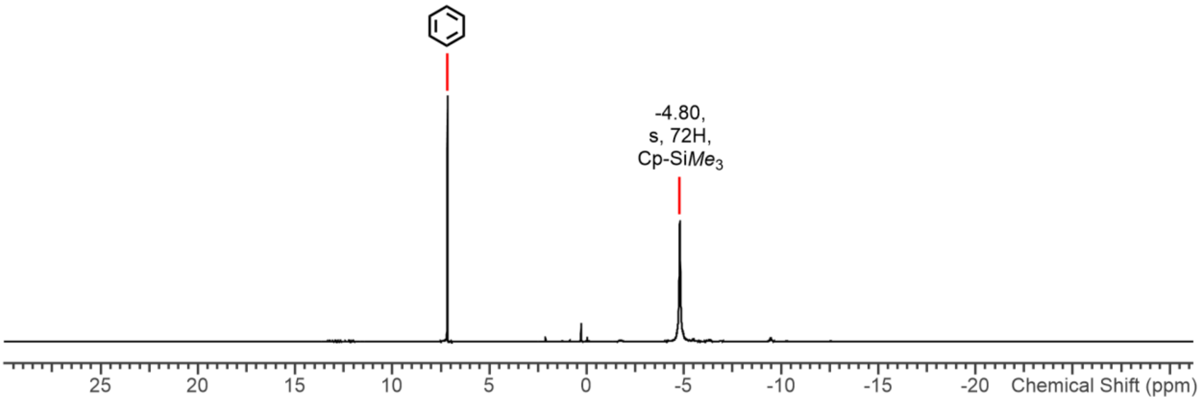

Figure S17. ^{1}H NMR spectrum (400.07 MHz) of **2-Nd** in C_6D_6.

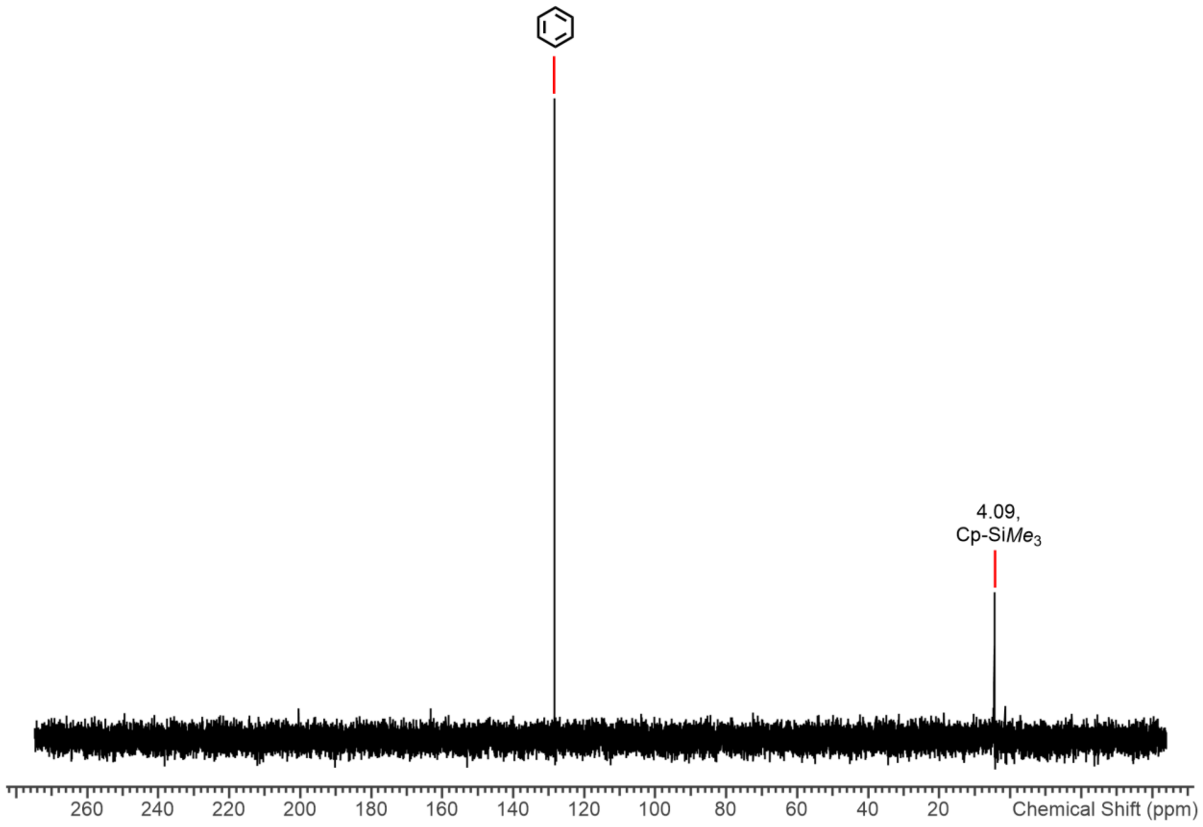

Figure S18. ^{13}C DEPT135 NMR spectrum (100.60 MHz) of **2-Nd** in C_6D_6.

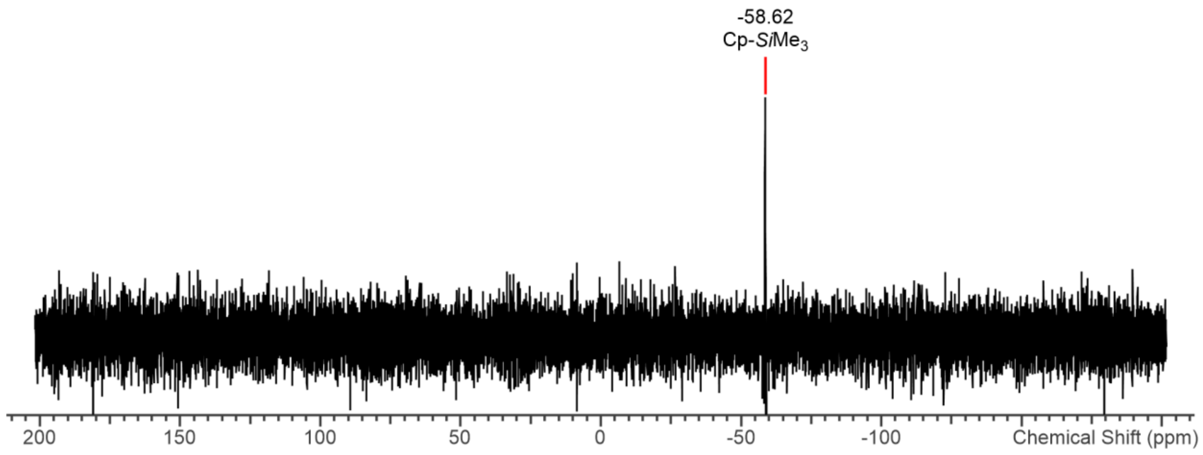

Figure S19. ^{29}Si{^{1}H} NMR spectrum (79.48 MHz) of **2-Nd** in C_6D_6.

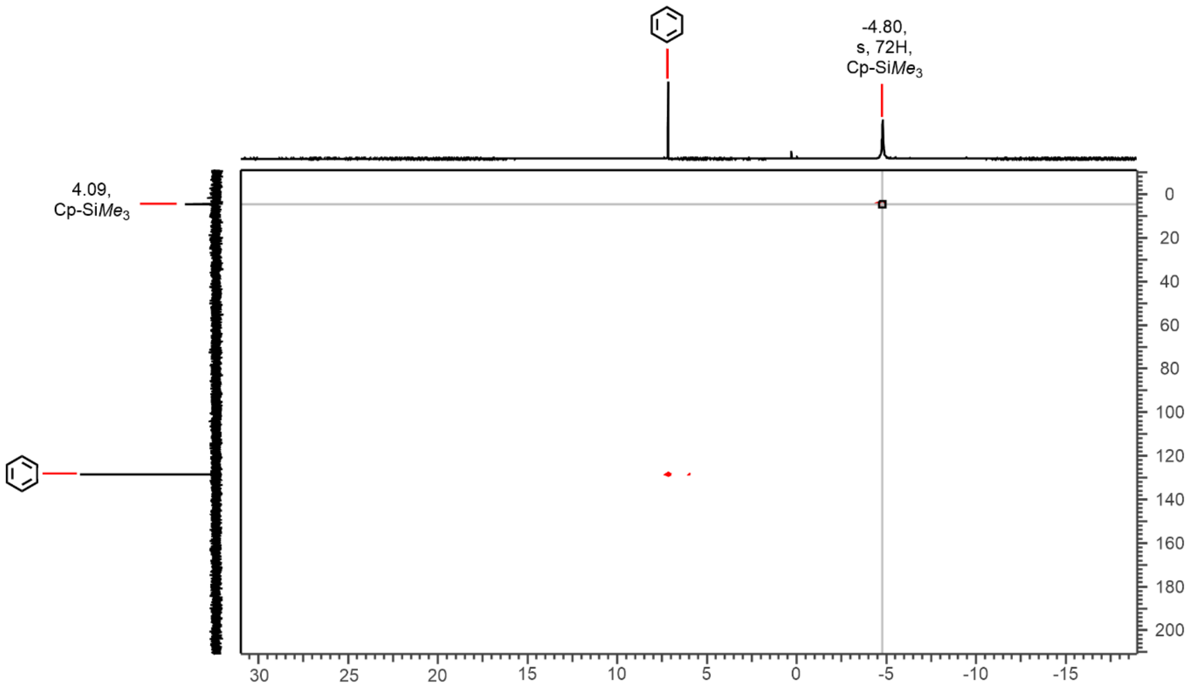

Figure S20. ^{1}H-^{13}C HSQC NMR spectrum of **2-Nd** in C_6D_6.

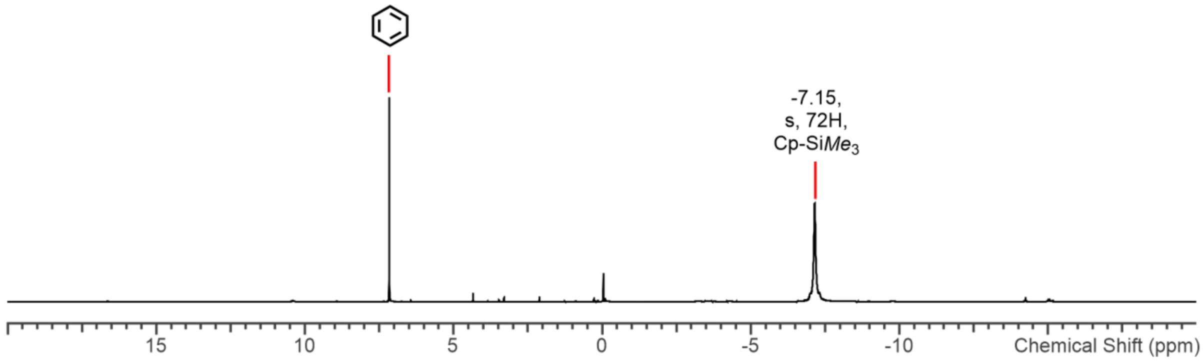

Figure S21. ^{1}H NMR spectrum (400.07 MHz) of **2-U** in C_6D_6.

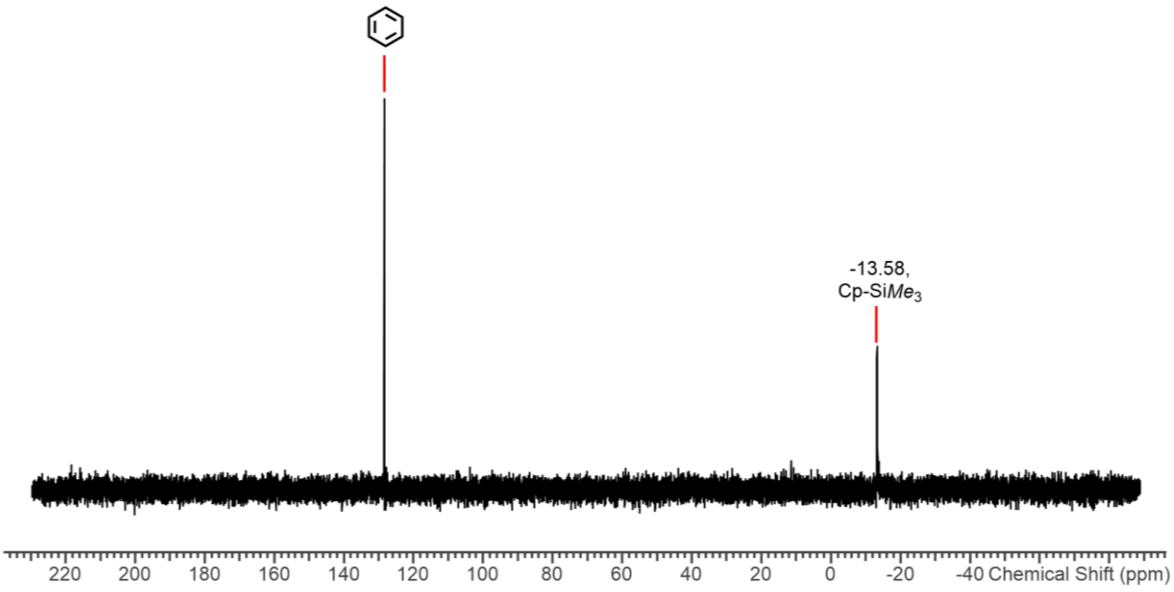

Figure S22. ^{13}C DEPT135 NMR spectrum (100.60 MHz) of **2-U** in C_6D_6.

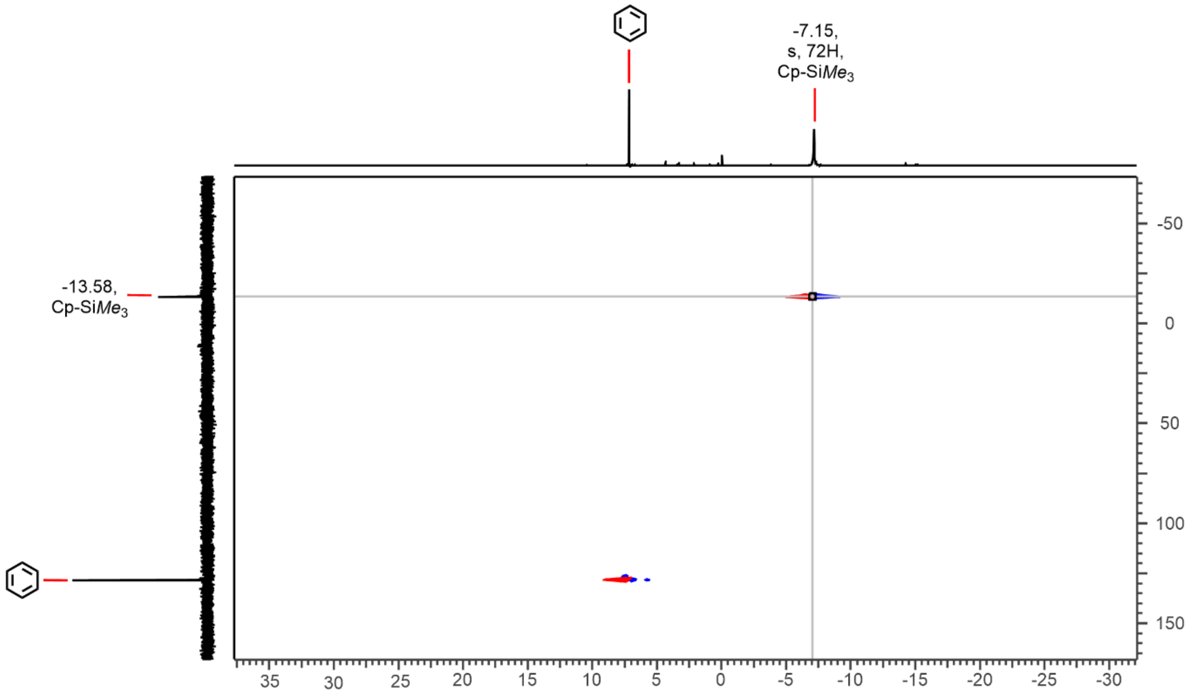

Figure S23. ^{1}H-^{13}C HSQC NMR spectrum of **2-U** in C_6D_6.

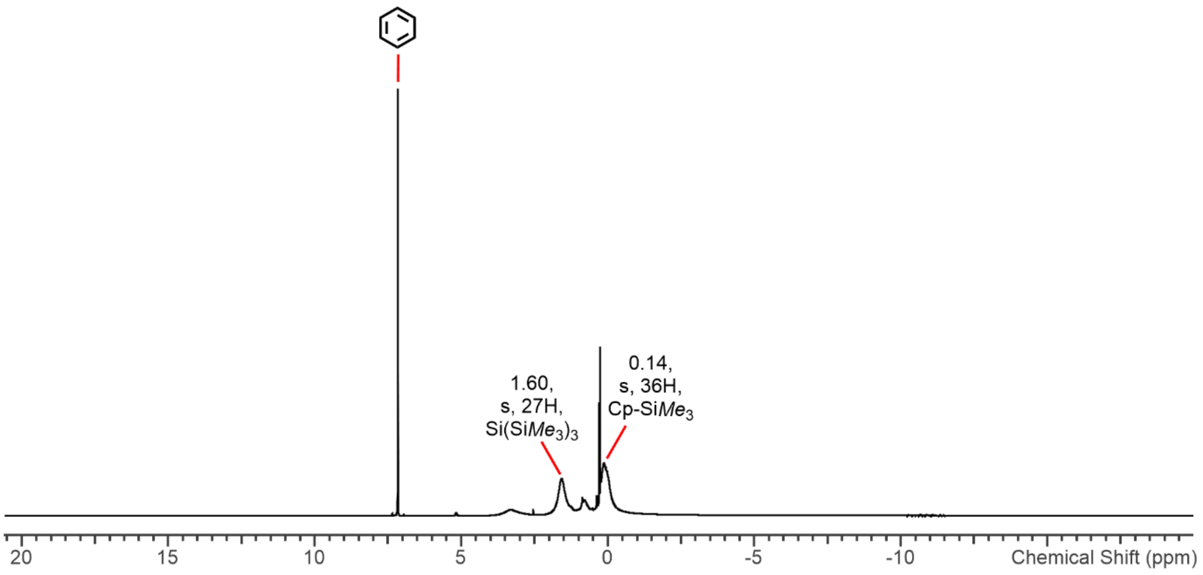

Figure S24. ^{1}H NMR spectrum (400.07 MHz) of **3-Ti** in C_6D_6.

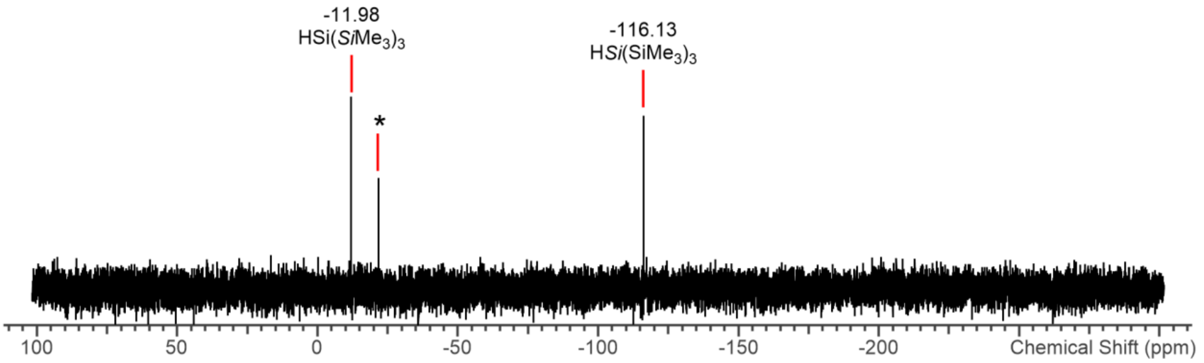

Figure S25. ^{29}Si{^{1}H} NMR spectrum (79.48 MHz) of **3-Ti** in C_6D_6. * denotes silicone grease impurity.

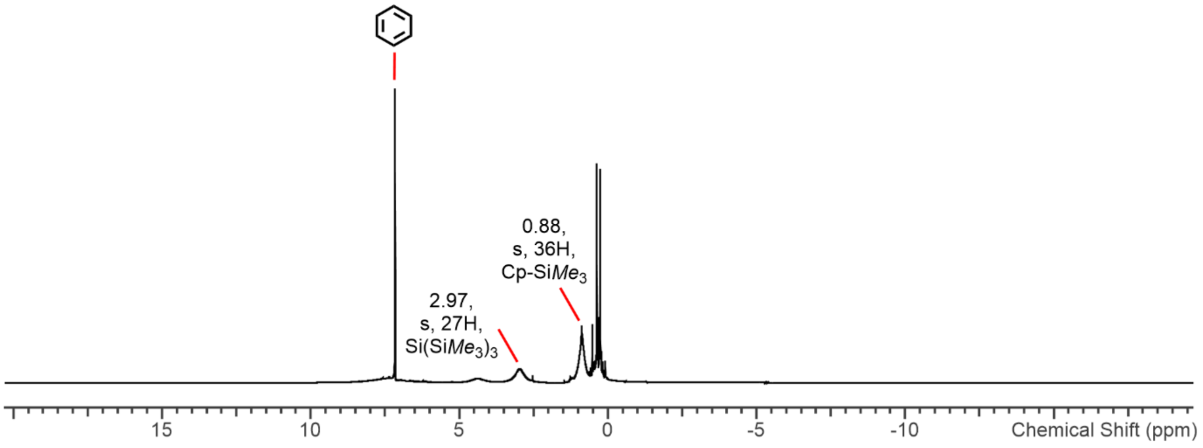

Figure S26. ^{1}H NMR spectrum (400.07 MHz) of **3-Zr** in C_6D_6.

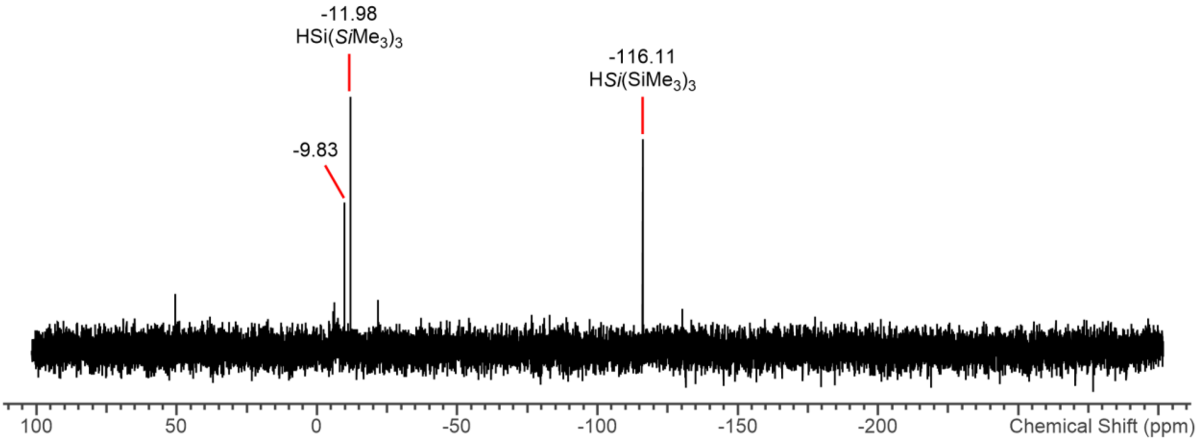

Figure S27. $^{29}Si\{^{1}H\}$ NMR spectrum (79.48 MHz) of **3-Zr** in C_6D_6.

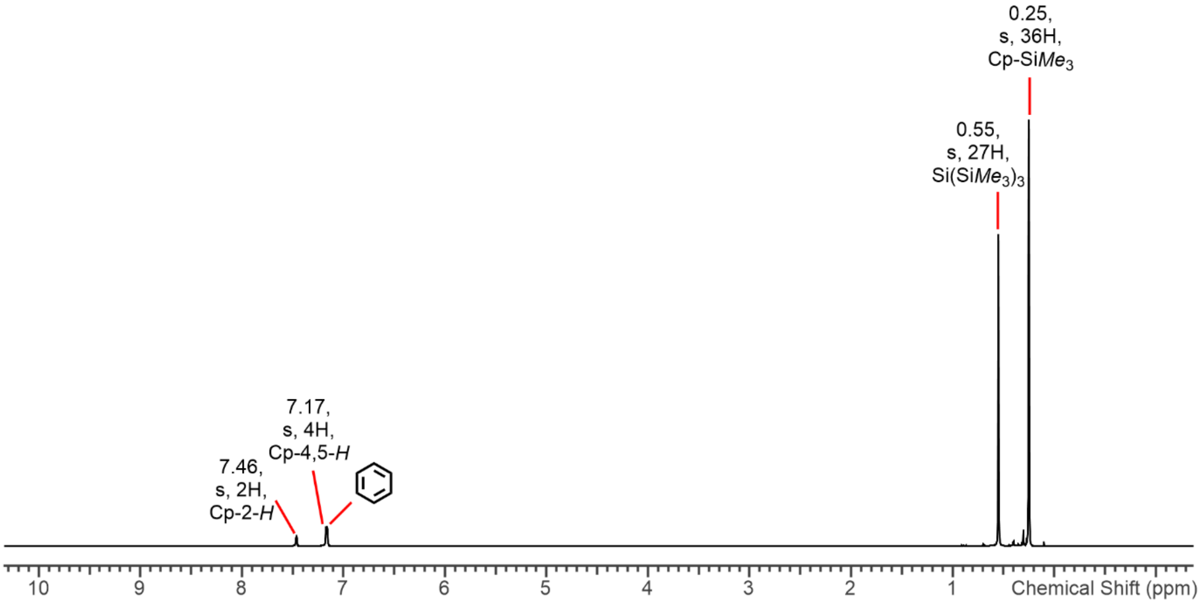

Figure S28. ^{1}H NMR spectrum (400.07 MHz) of **3-La** in C_6D_6.

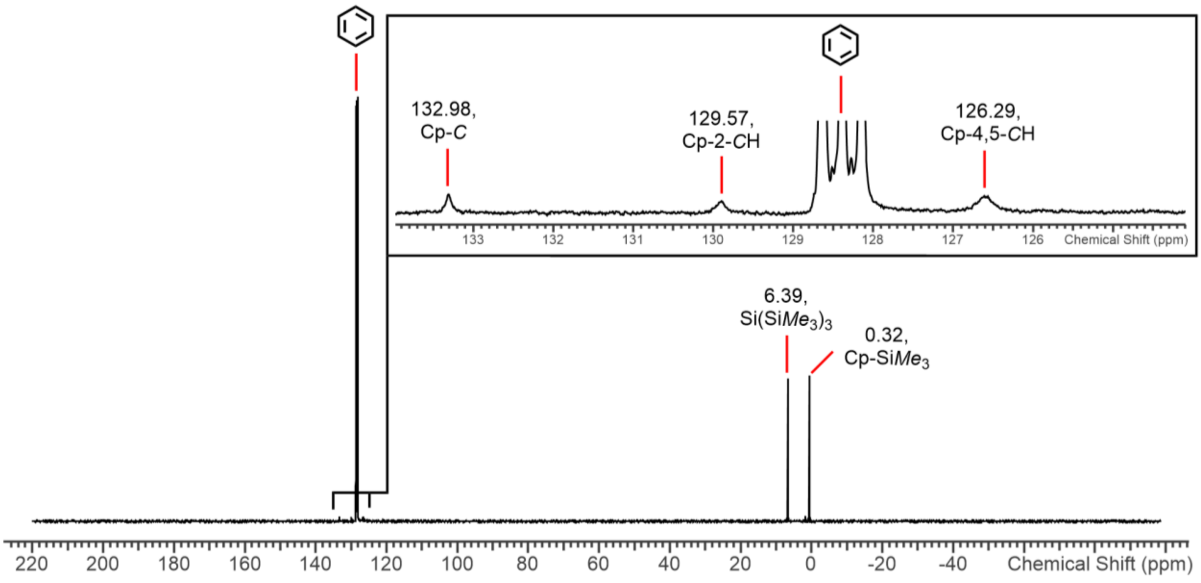

Figure S29. $^{13}C\{^1H\}$ NMR spectrum (100.60 MHz) of **3-La** in C_6D_6.

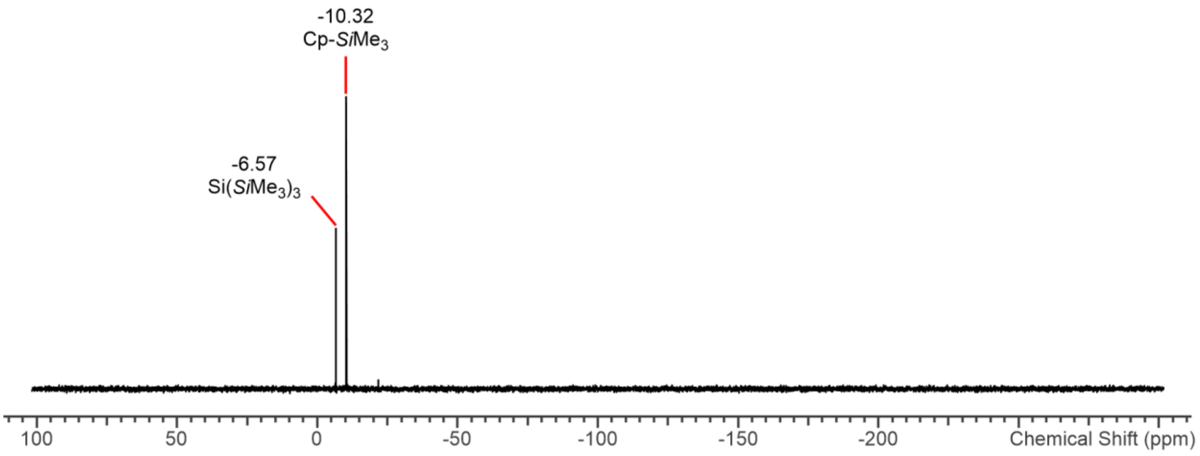

Figure S30. $^{29}Si\{^1H\}$ NMR spectrum (79.48 MHz) of **3-La** in C_6D_6.

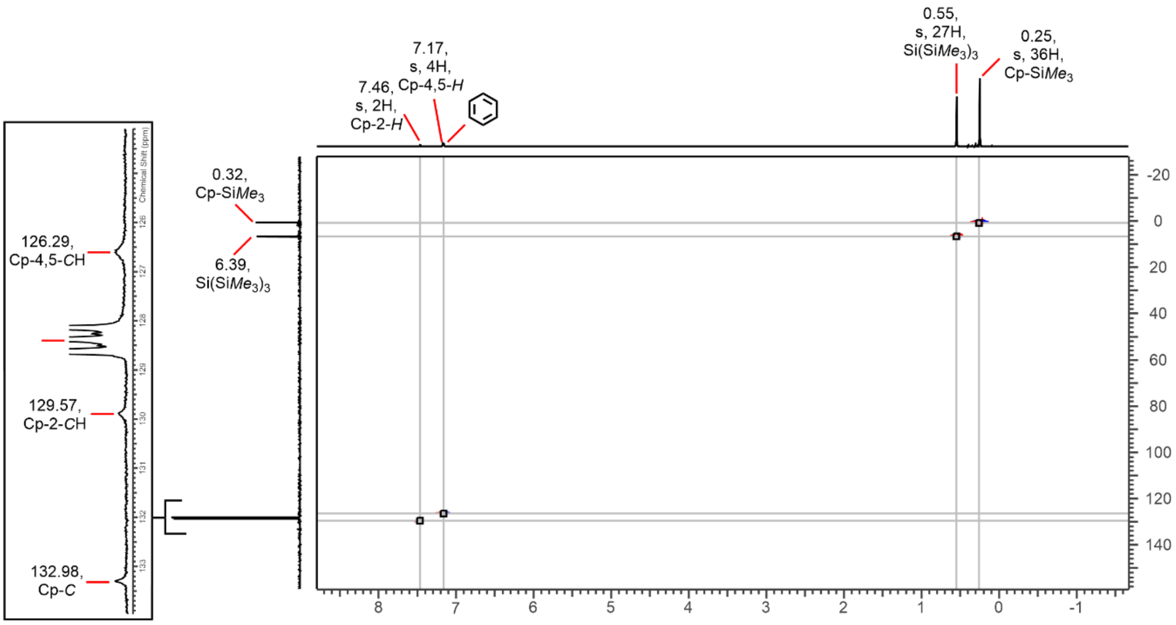

Figure S31. ^{1}H-^{13}C HSQC NMR spectrum of **3-La** in C_6D_6.

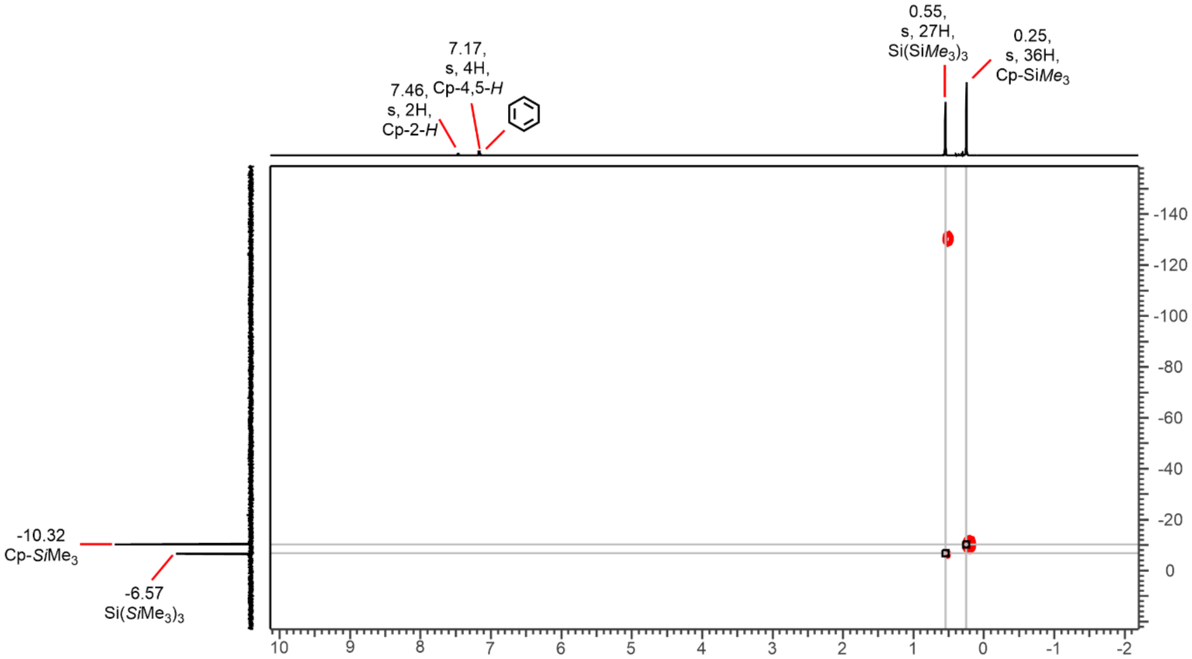

Figure S32. ^{1}H-^{29}Si HMBC NMR spectrum of **3-La** in C_6D_6.

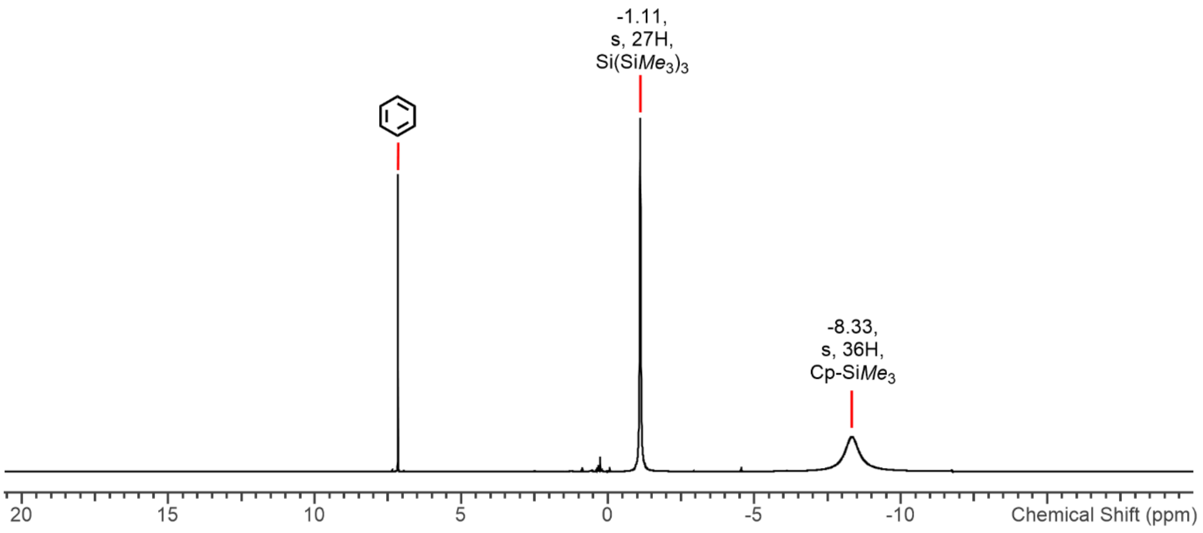

Figure S33. ^{1}H NMR spectrum (400.07 MHz) of **3-Ce** in C_6D_6.

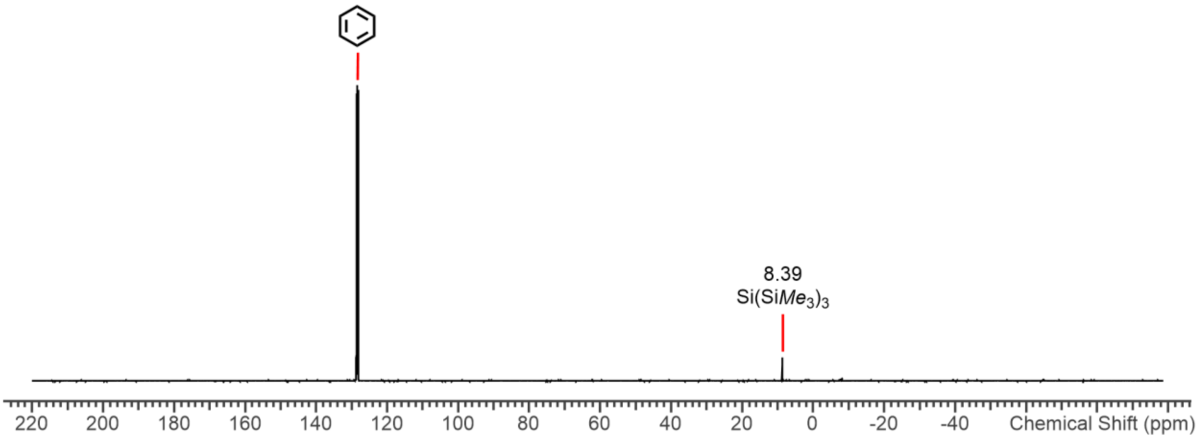

Figure S34. $^{13}C\{^{1}H\}$ NMR spectrum (100.60 MHz) of **3-Ce** in C_6D_6.

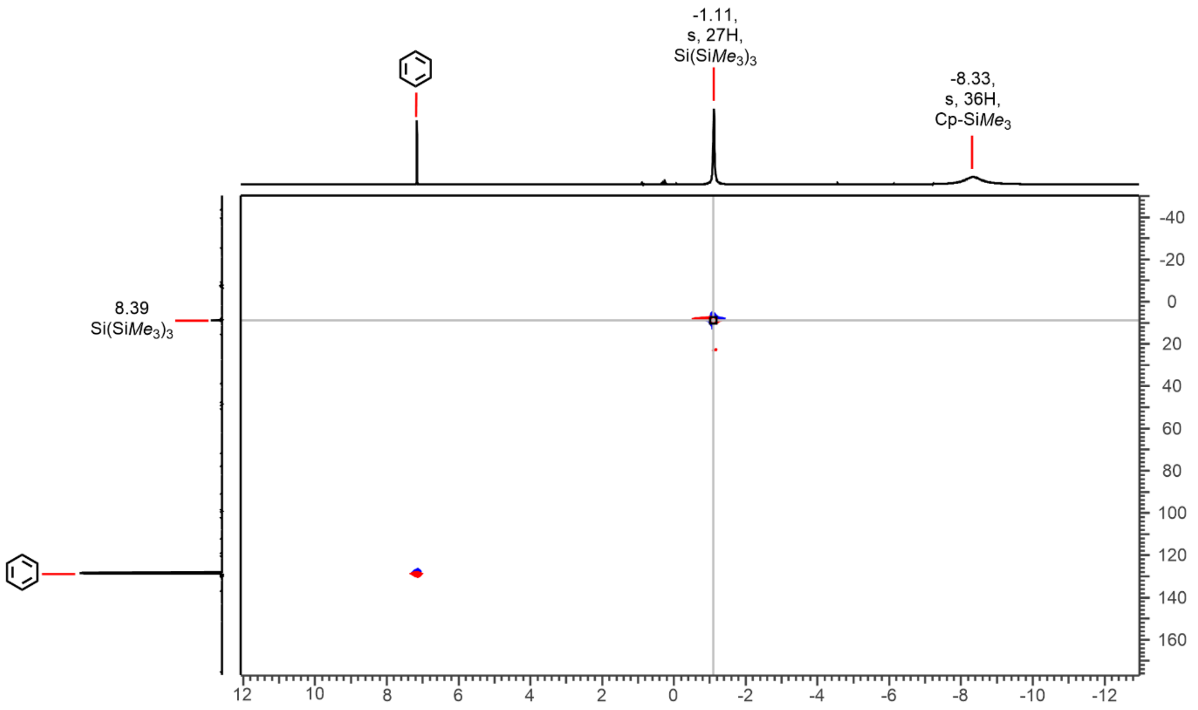

Figure S35. ^{1}H-^{13}C HSQC NMR spectrum of **3-Ce** in C_6D_6.

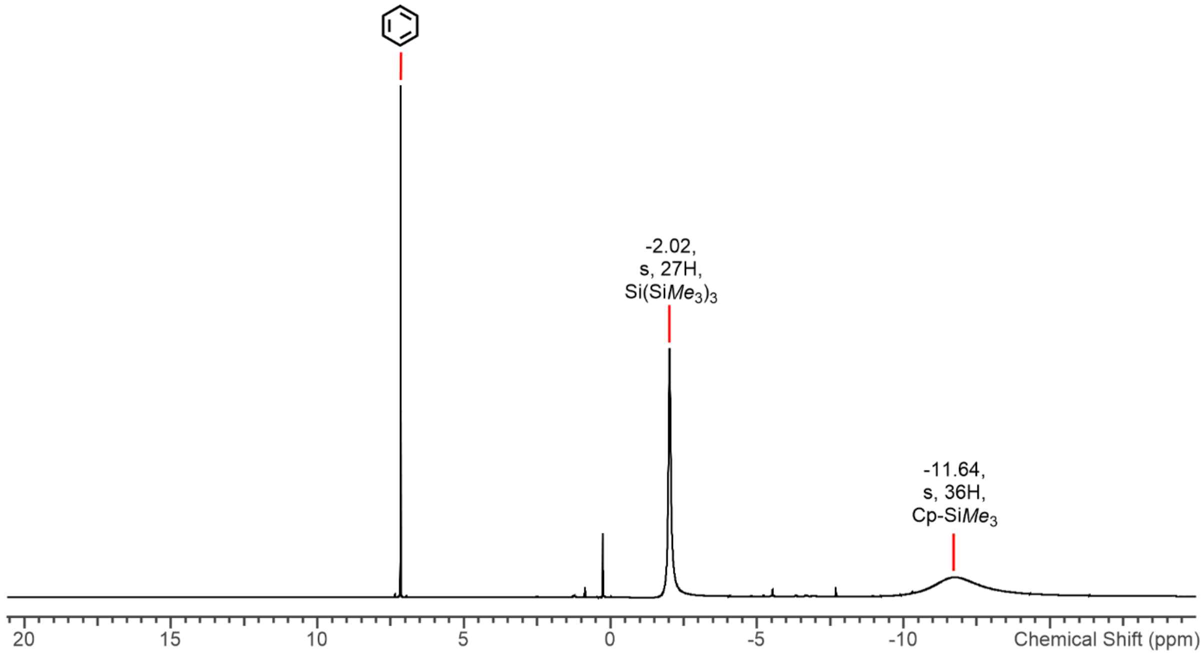

Figure S36. ^{1}H NMR spectrum (400.07 MHz) of **3-Nd** in C_6D_6.

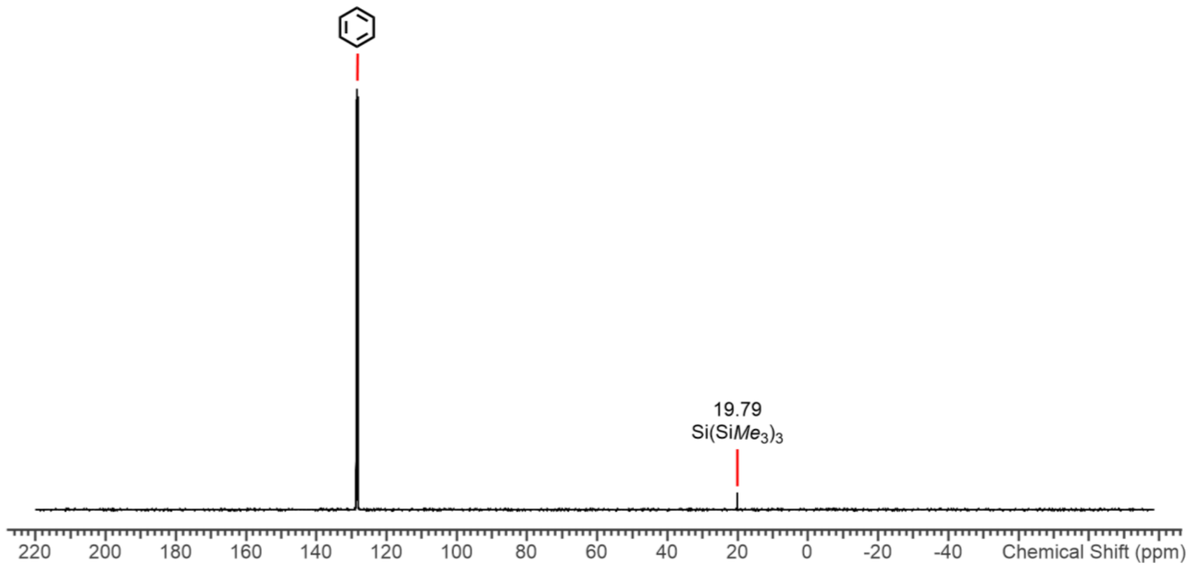

Figure S37. $^{13}C\{^{1}H\}$ NMR spectrum (100.60 MHz) of **3-Nd** in C_6D_6.

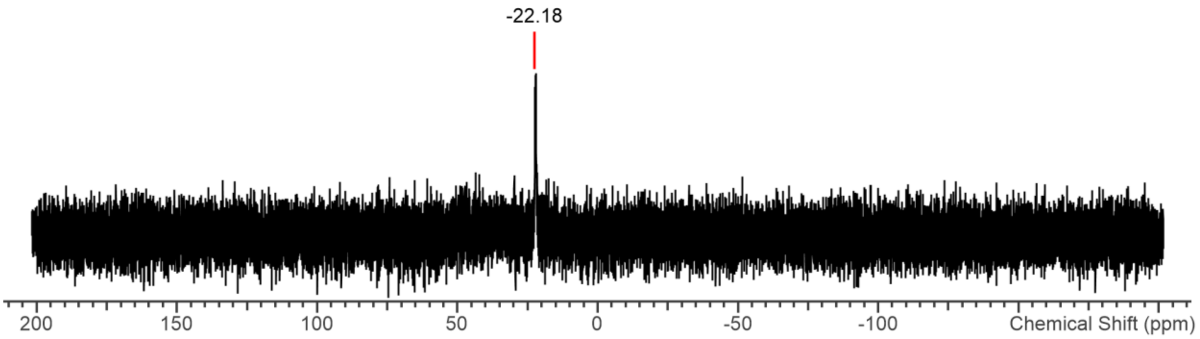

Figure S38. $^{29}Si\{^{1}H\}$ NMR spectrum (79.48 MHz) of **3-Nd** in C_6D_6.

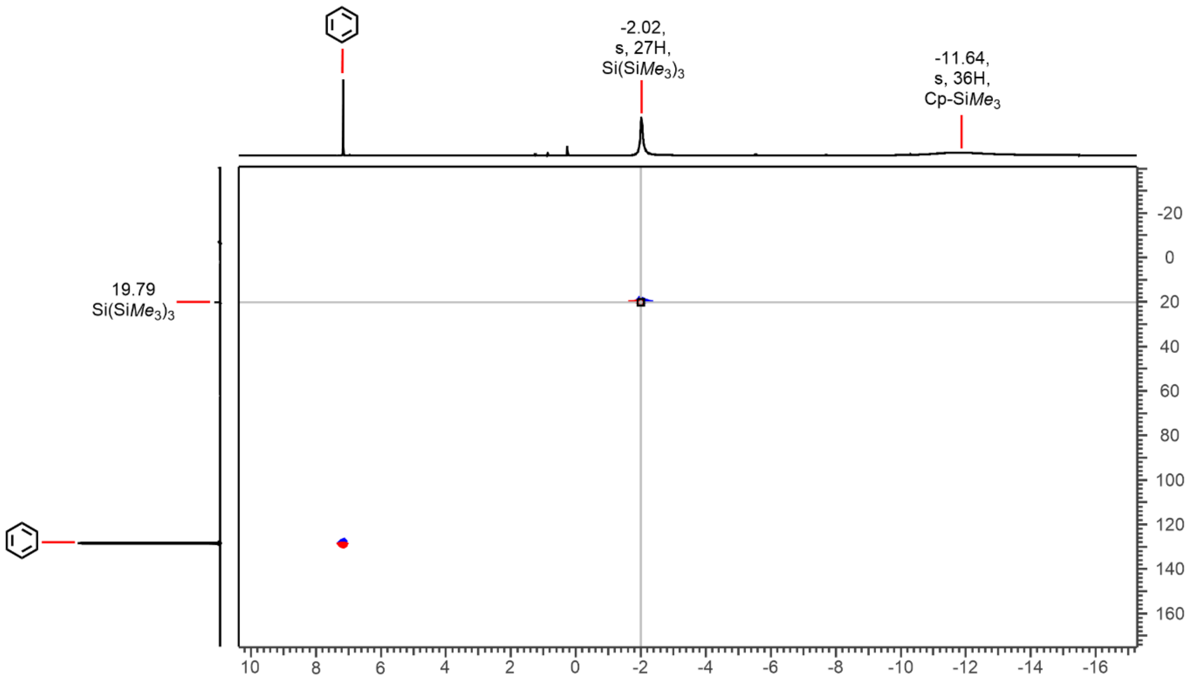

Figure S39. ^{1}H-^{13}C HSQC NMR spectrum of **3-Nd** in C_6D_6.

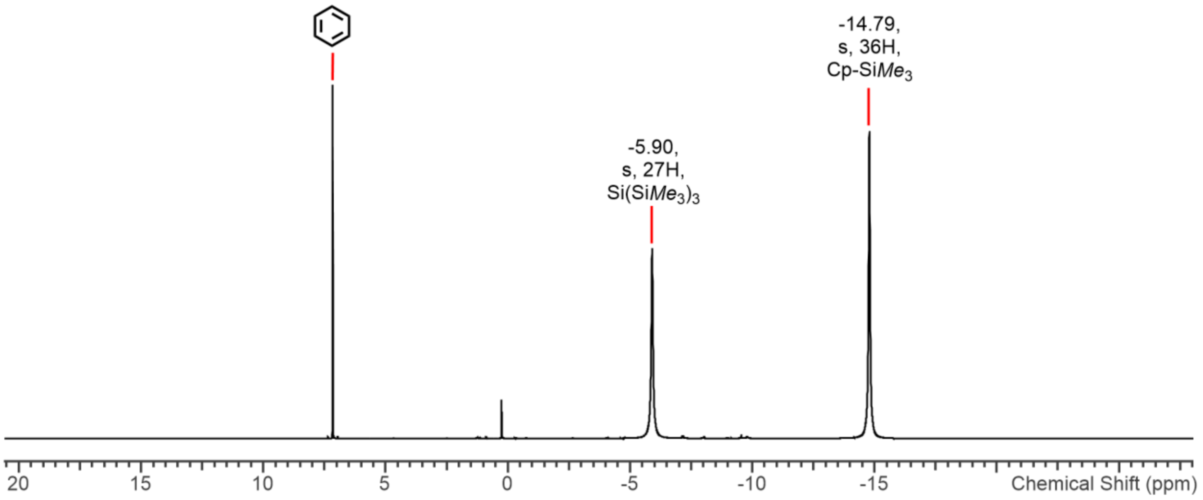

Figure S40. ^{1}H NMR spectrum (400.07 MHz) of **3-U** in C_6D_6.

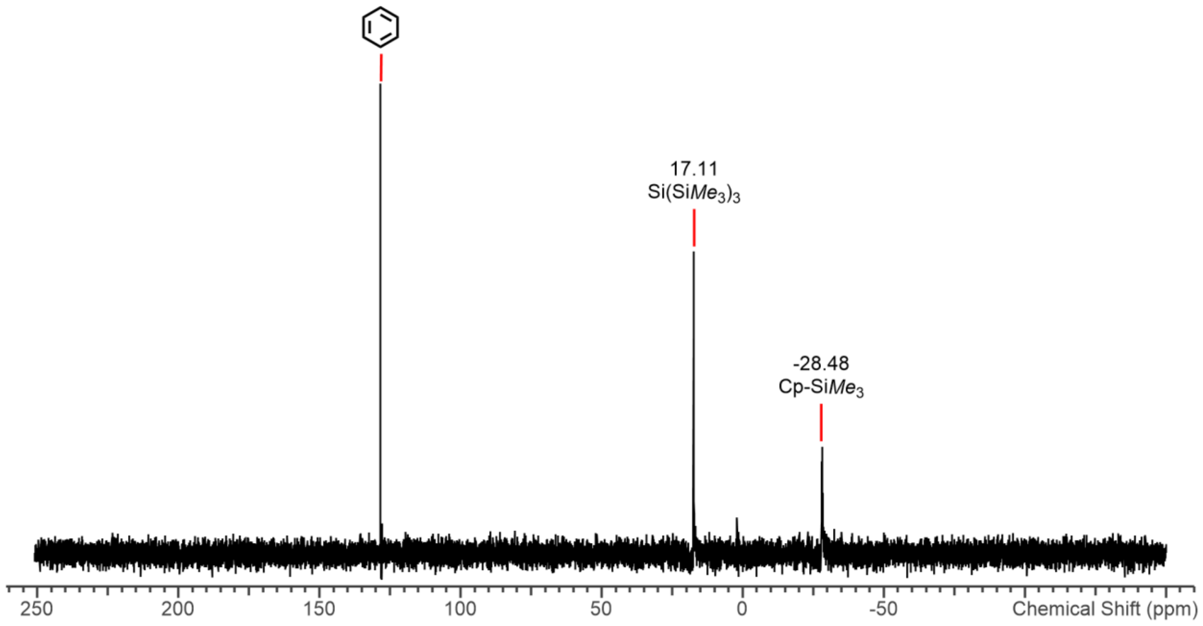

Figure S41. ^{13}C DEPT135 NMR spectrum (100.60 MHz) of **3-U** in C_6D_6.

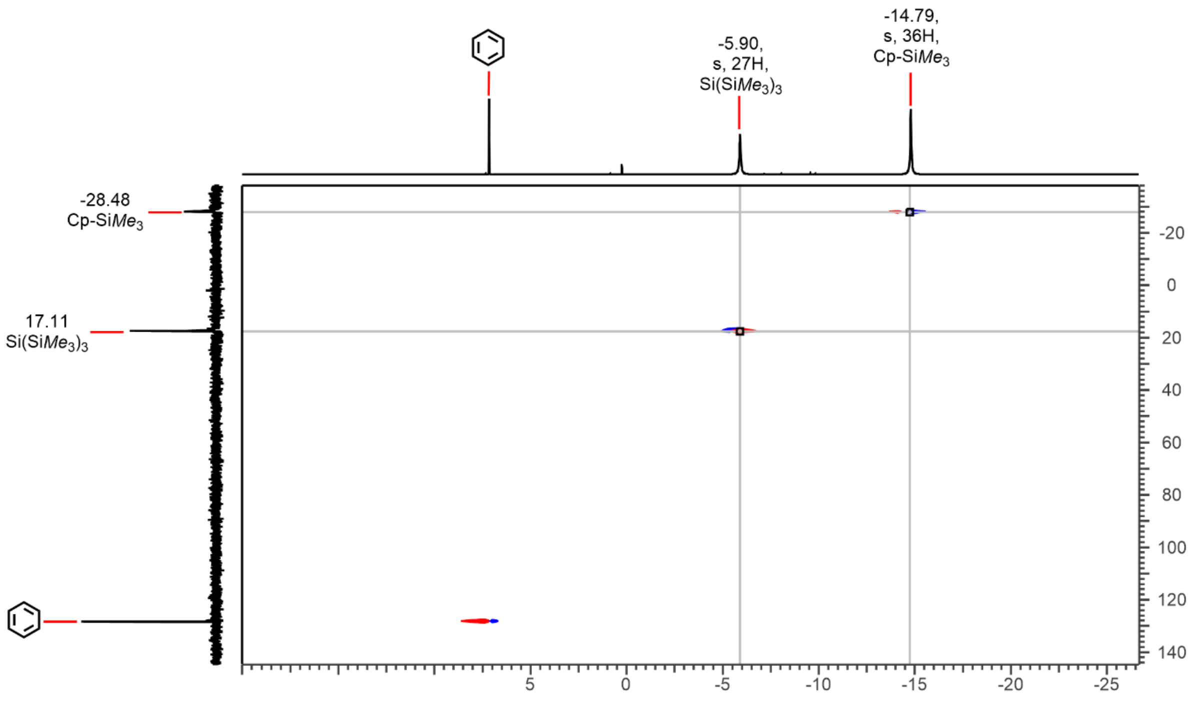

Figure S42. ^{1}H-^{13}C HSQC NMR spectrum of **3-U** in C_6D_6.

4. Evans Method Magnetic Susceptibility NMR Spectra

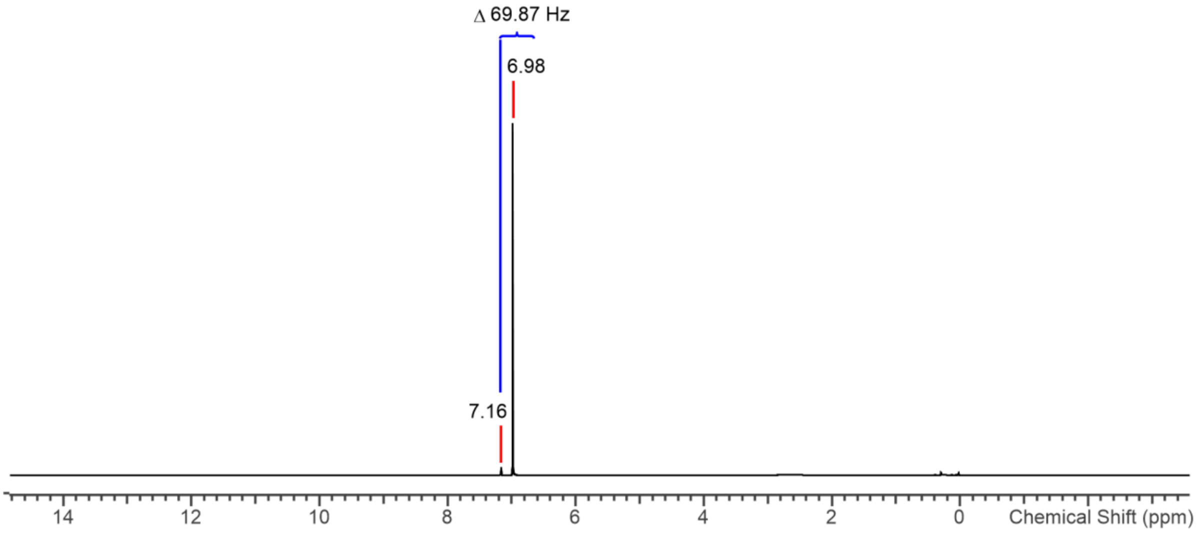

Figure S43. ^{1}H NMR spectrum (400.07 MHz) of **1-Ti** in C_6D_6 with a glass capillary containing 1:1 C_6H_6:C_6D_6 used to determine the magnetic susceptibility in solution at 300 K.

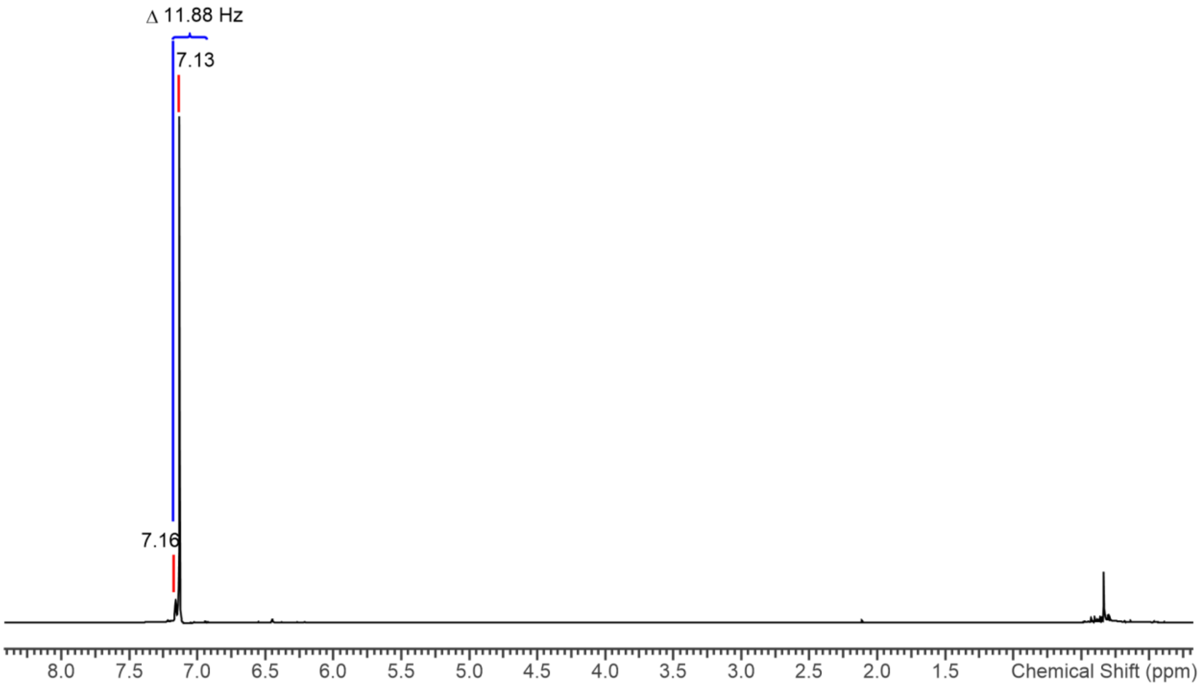

Figure S44. ^{1}H NMR spectrum (400.07 MHz) of **2-Zr** in C_6D_6 with a glass capillary containing 1:1 C_6H_6:C_6D_6 used to determine the magnetic susceptibility in solution at 300 K.

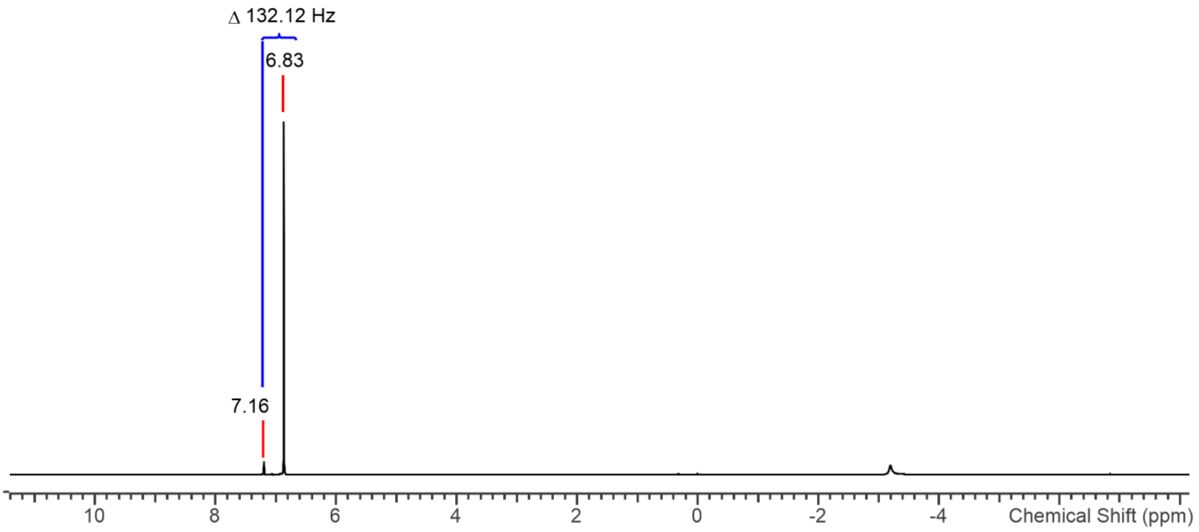

Figure S45. ^{1}H NMR spectrum (400.07 MHz) of **2-Ce** in C_6D_6 with a glass capillary containing 1:1 C_6H_6:C_6D_6 used to determine the magnetic susceptibility in solution at 300 K.

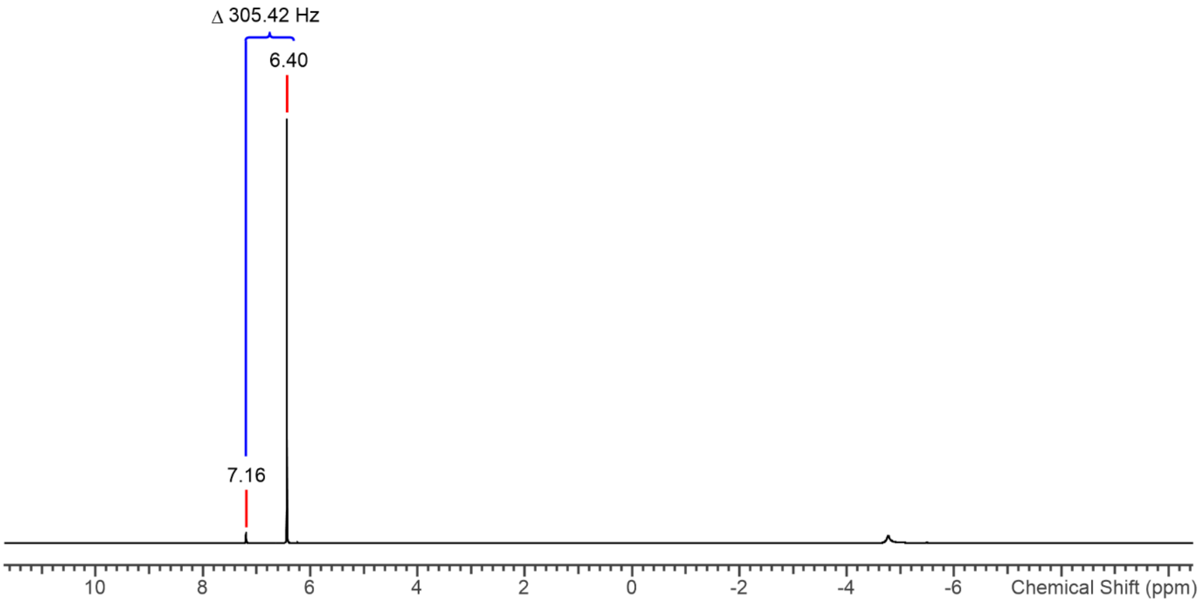

Figure S46. ^{1}H NMR spectrum (400.07 MHz) of **2-Nd** in C_6D_6 with a glass capillary containing 1:1 C_6H_6:C_6D_6 used to determine the magnetic susceptibility in solution at 300 K.

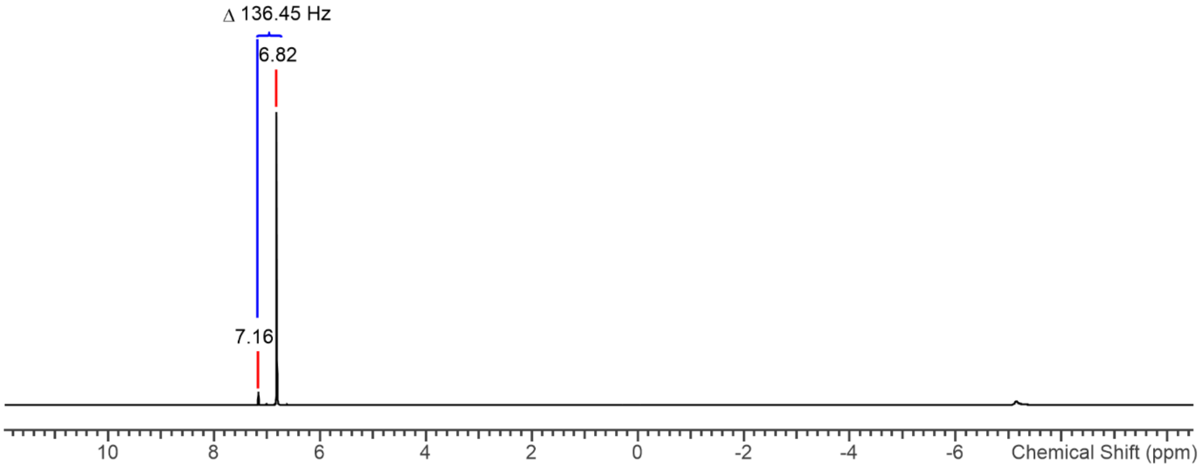

Figure S47. ^{1}H NMR spectrum (400.07 MHz) of **2-U** in C_6D_6 with a glass capillary containing 1:1 C_6H_6:C_6D_6 used to determine the magnetic susceptibility in solution at 300 K.

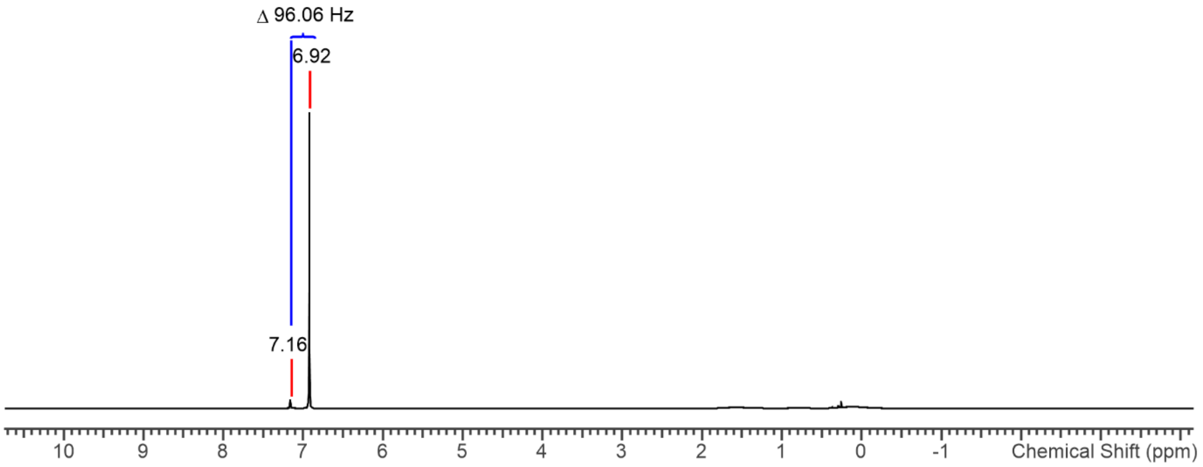

Figure S48. ^{1}H NMR spectrum (400.07 MHz) of **3-Ti** in C_6D_6 with a glass capillary containing 1:1 C_6H_6:C_6D_6 used to determine the magnetic susceptibility in solution at 300 K.

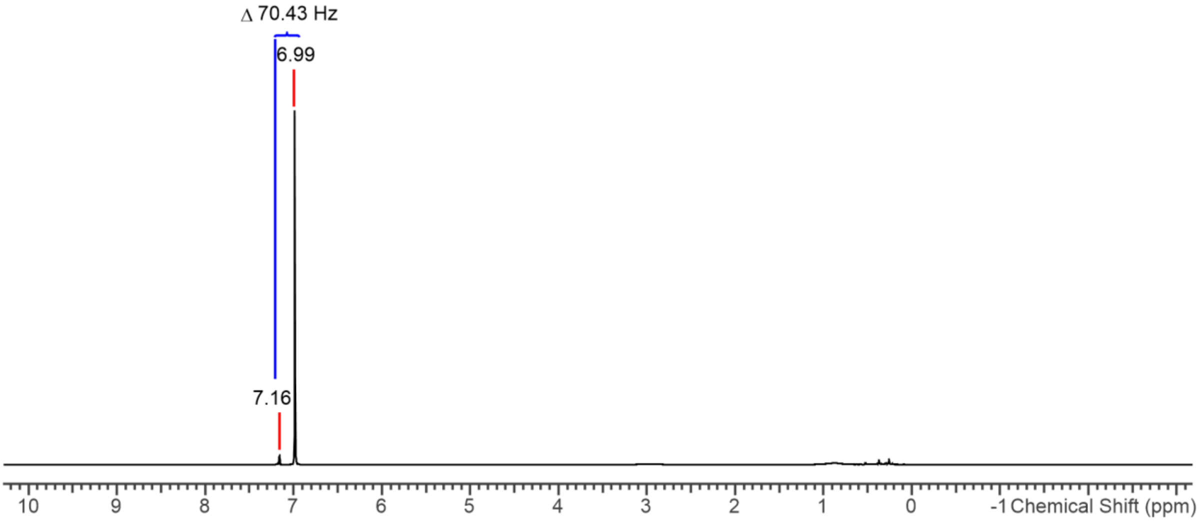

Figure S49. ^{1}H NMR spectrum (400.07 MHz) of **3-Zr** in C_6D_6 with a glass capillary containing 1:1 C_6H_6:C_6D_6 used to determine the magnetic susceptibility in solution at 300 K.

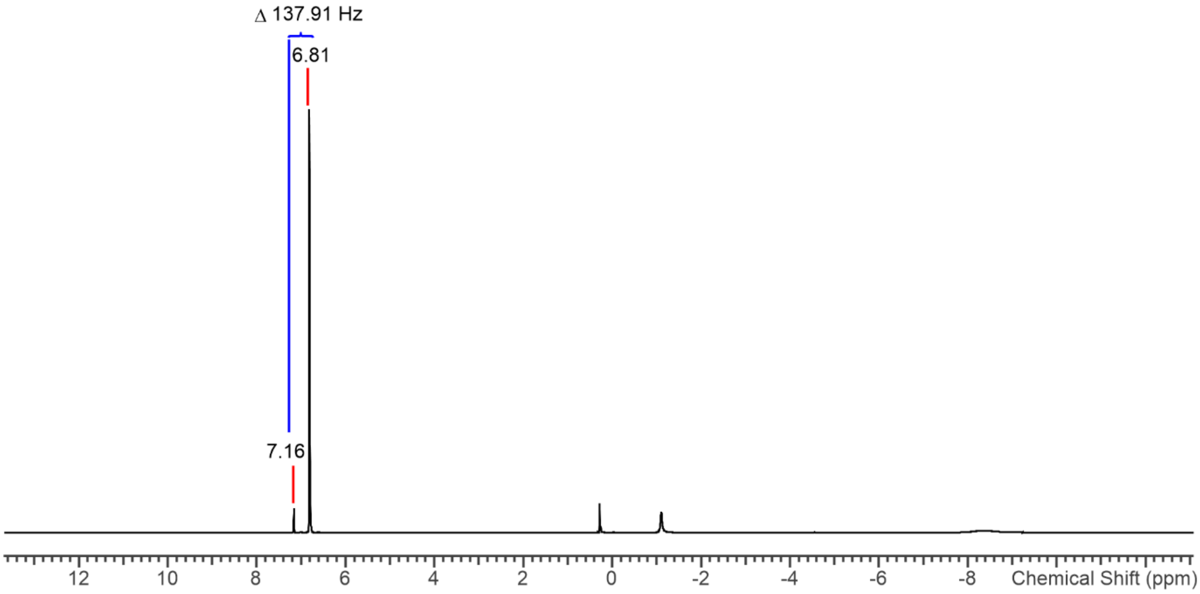

Figure S50. ^{1}H NMR spectrum (400.07 MHz) of **3-Ce** in C_6D_6 with a glass capillary containing 1:1 C_6H_6:C_6D_6 used to determine the magnetic susceptibility in solution at 300 K.

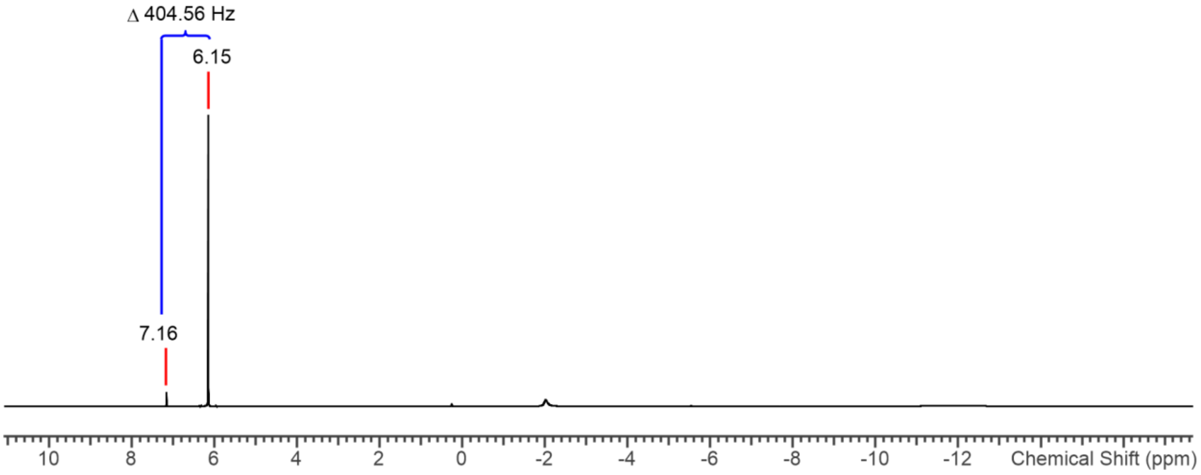

Figure S51. ^{1}H NMR spectrum (400.07 MHz) of **3-Nd** in C_6D_6 with a glass capillary containing 1:1 C_6H_6:C_6D_6 used to determine the magnetic susceptibility in solution at 300 K.

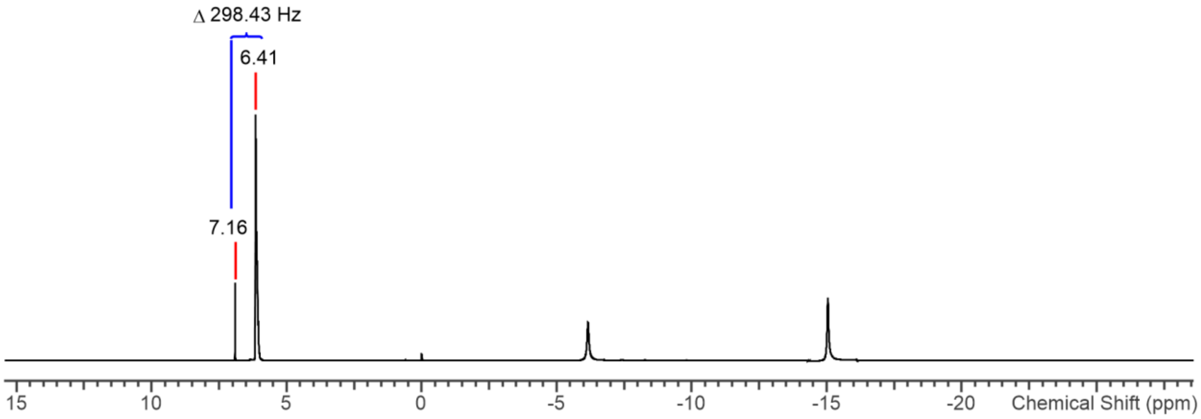

Figure S52. ^{1}H NMR spectrum (400.07 MHz) of **3-U** in C_6D_6 with a glass capillary containing 1:1 C_6H_6:C_6D_6 used to determine the magnetic susceptibility in solution at 300 K.

5. ATR-IR Spectroscopy

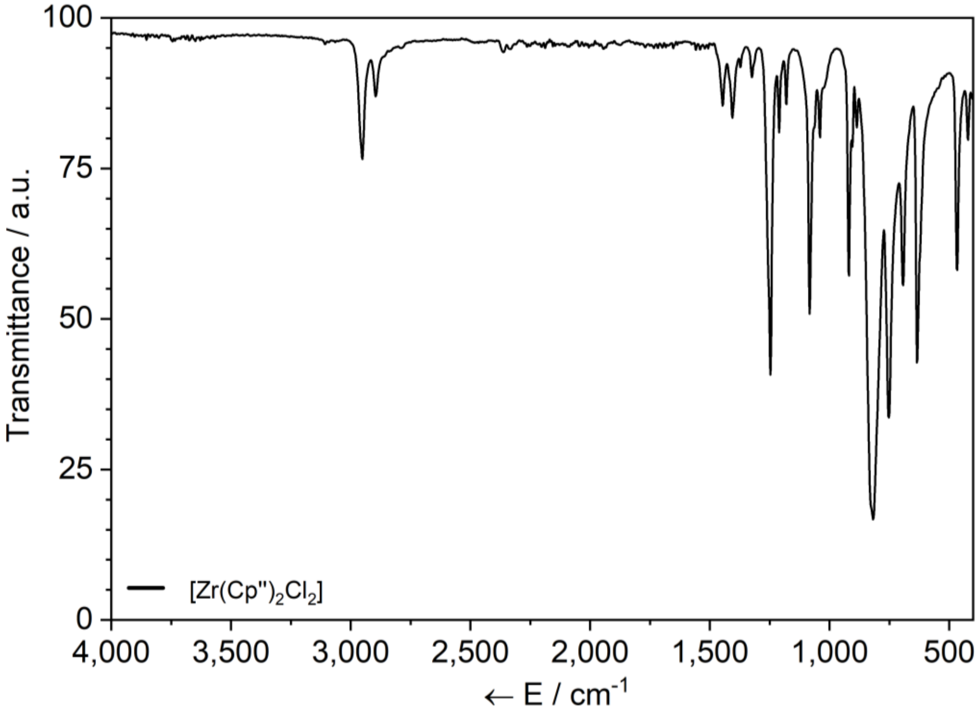

Figure S53. ATR-IR spectrum of [Zr(Cp′′)$_2$Cl$_2$] between 398–4,000 cm^{-1}.

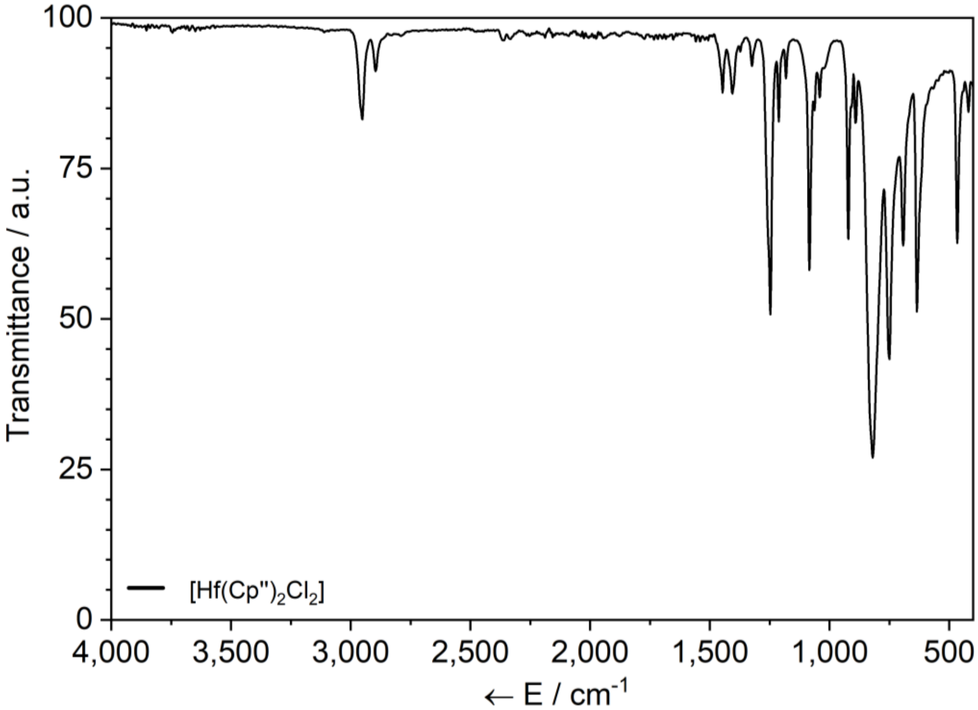

Figure S54. ATR-IR spectrum of [Hf(Cp′′)$_2$Cl$_2$] between 398–4,000 cm^{-1}.

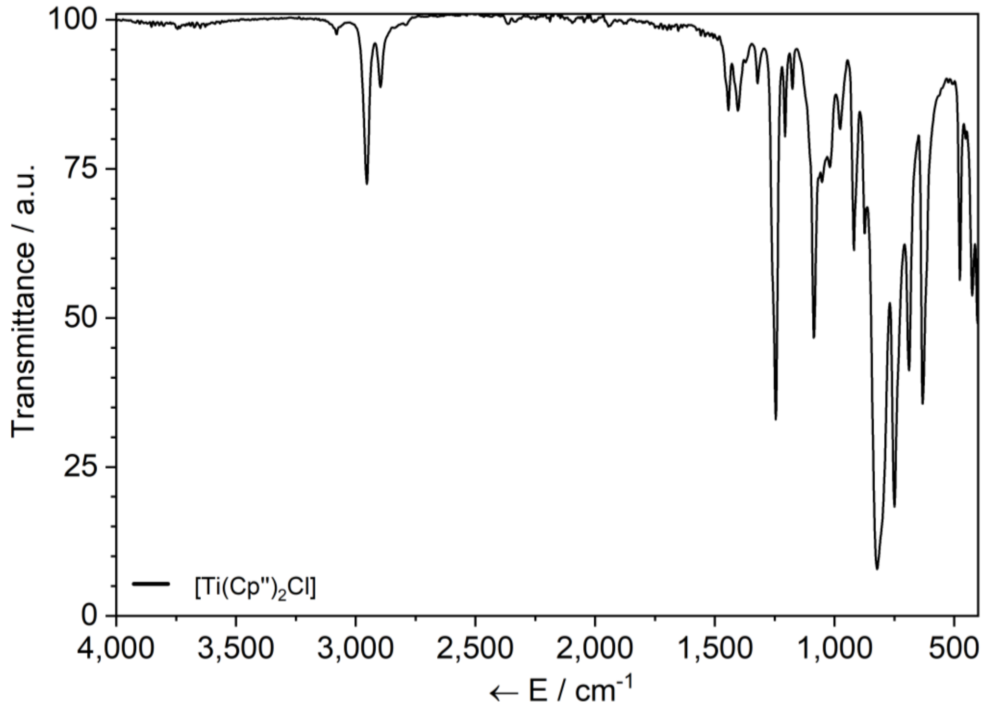

Figure S55. ATR-IR spectrum of **1-Ti** between 398–4,000 cm^{-1}.

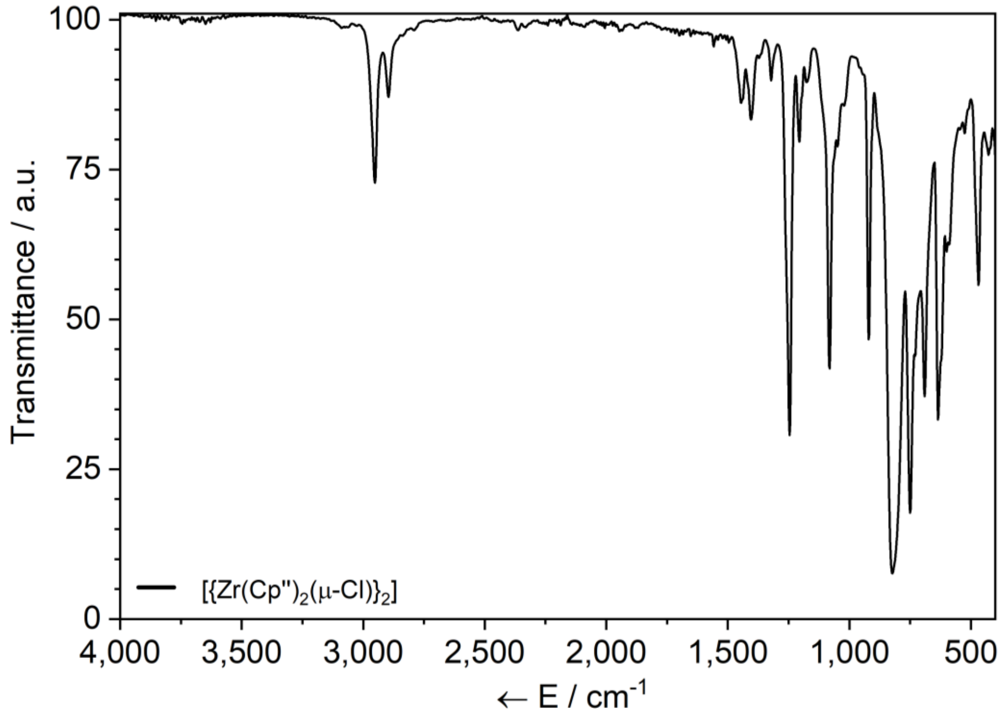

Figure S56. ATR-IR spectrum of **2-Zr** between 398–4,000 cm^{-1}.

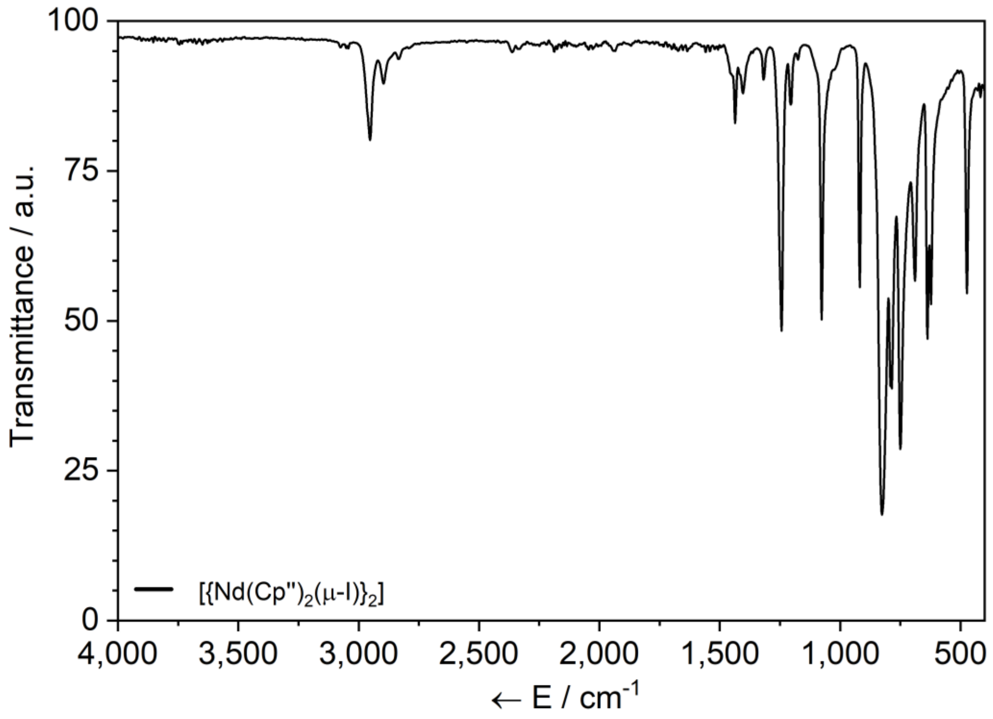

Figure S57. ATR-IR spectrum of **2-Nd** between 398–4,000 cm^{-1}.

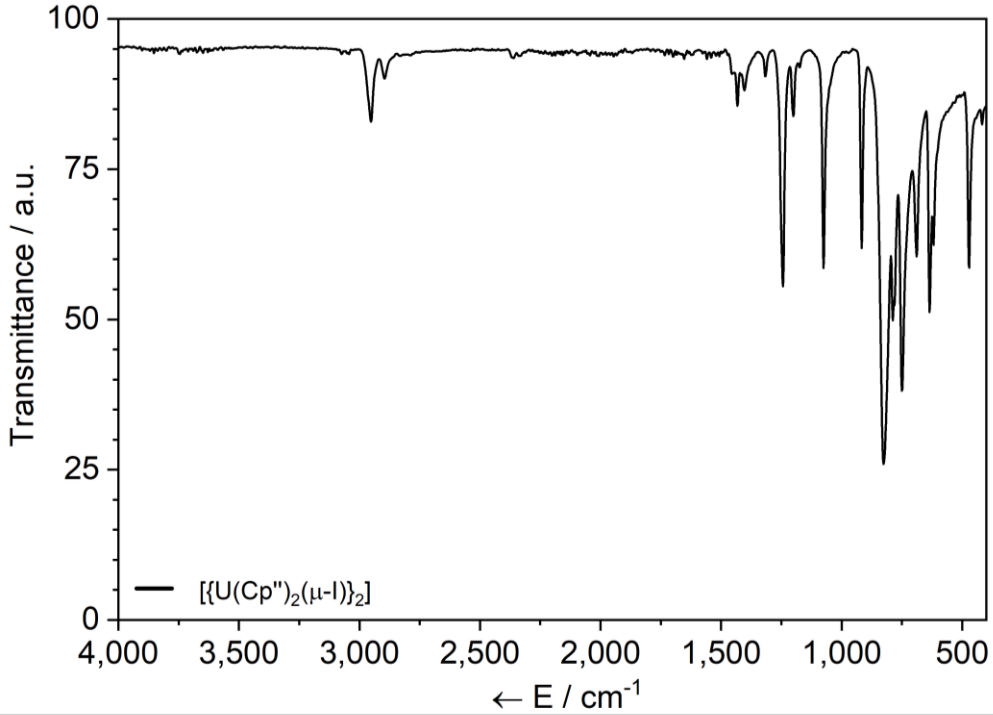

Figure S58. ATR-IR spectrum of **2-U** between 398–4,000 cm^{-1}.

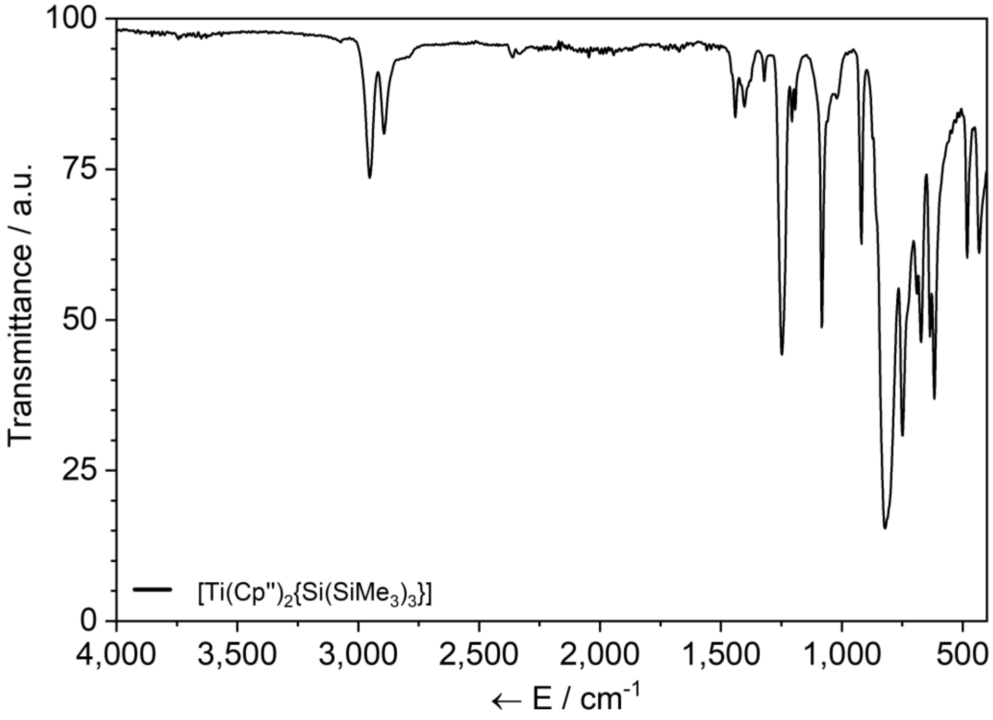

Figure S59. ATR-IR spectrum of **3-Ti** between 398–4,000 cm^{-1}.

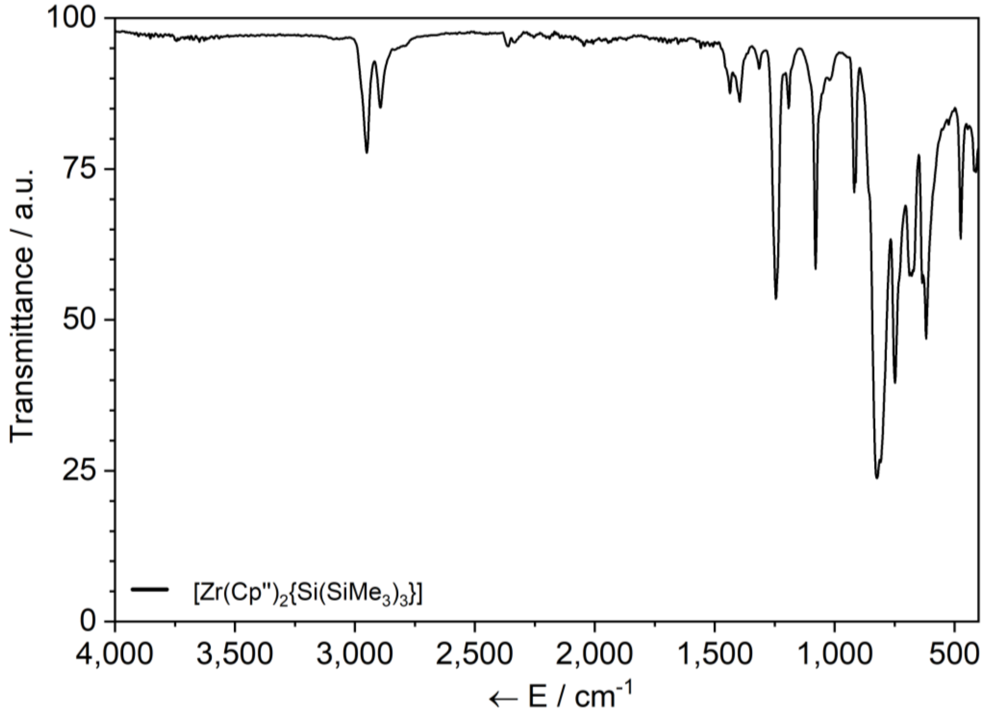

Figure S60. ATR-IR spectrum of **3-Zr** between 398–4,000 cm^{-1}.

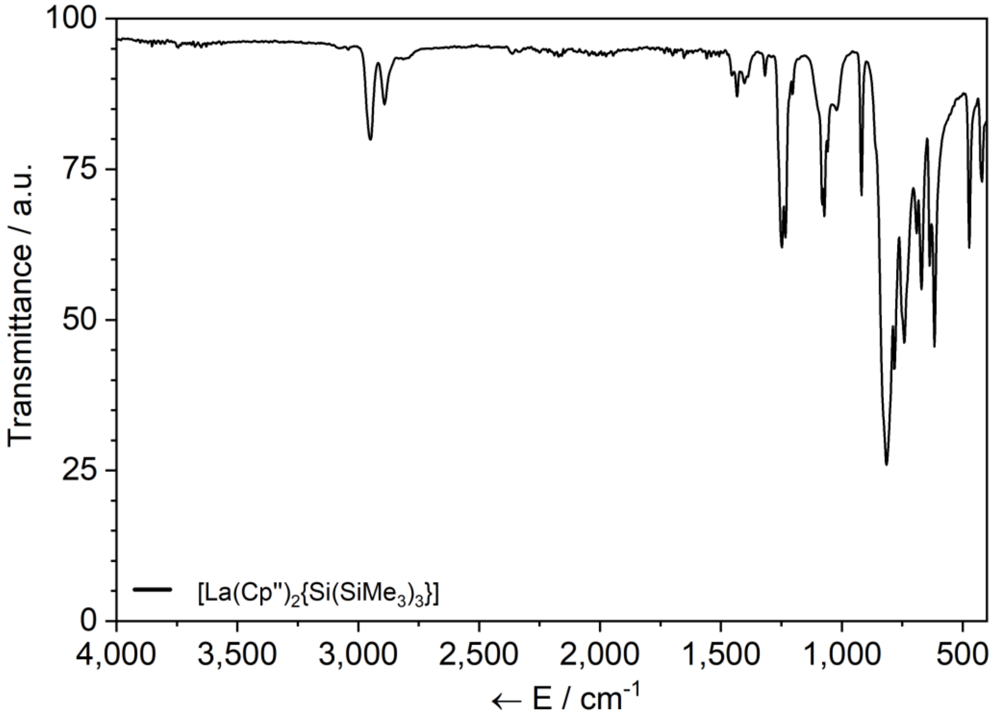

Figure S61. ATR-IR spectrum of **3-La** between 398–4,000 cm^{-1}.

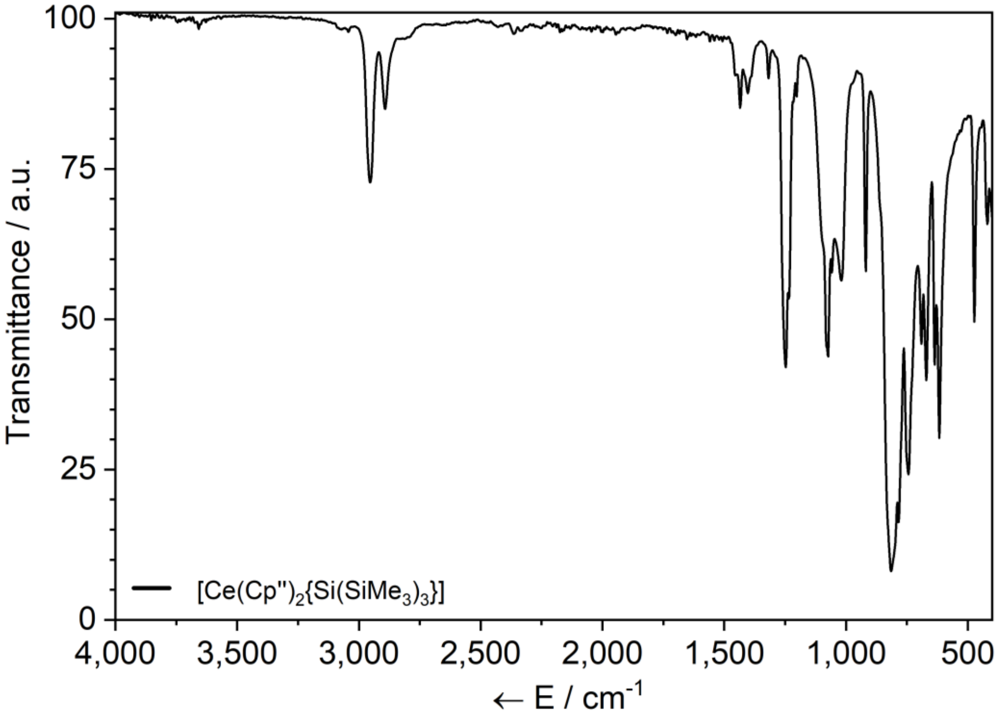

Figure S62. ATR-IR spectrum of **3-Ce** between 398–4,000 cm^{-1}.

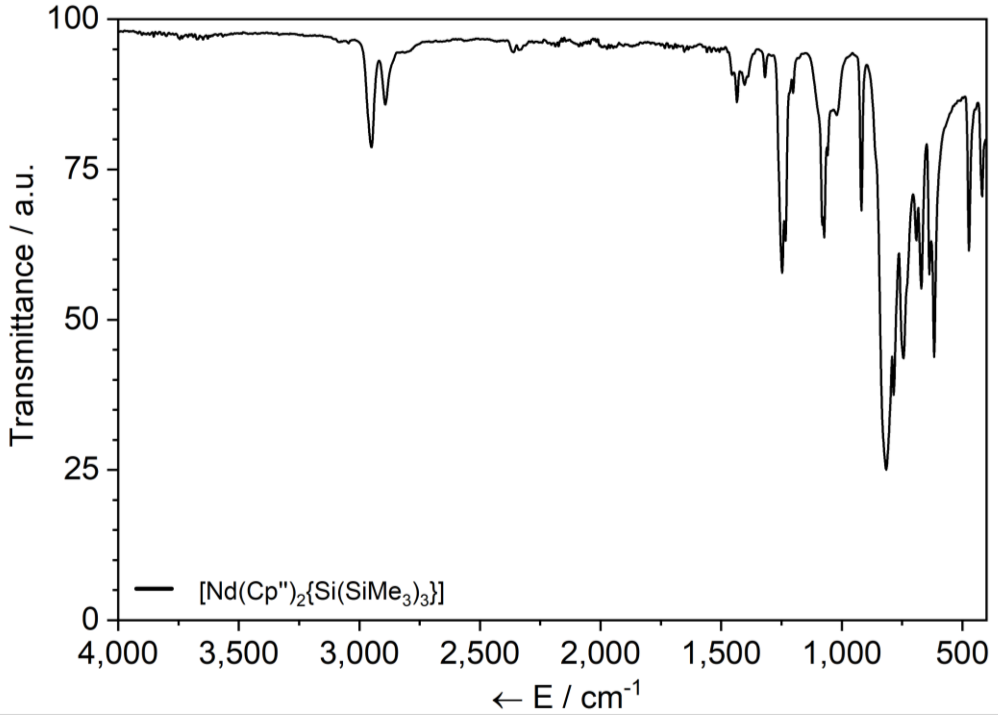

Figure S63. ATR-IR spectrum of **3-Nd** between 398–4,000 cm^{-1}.

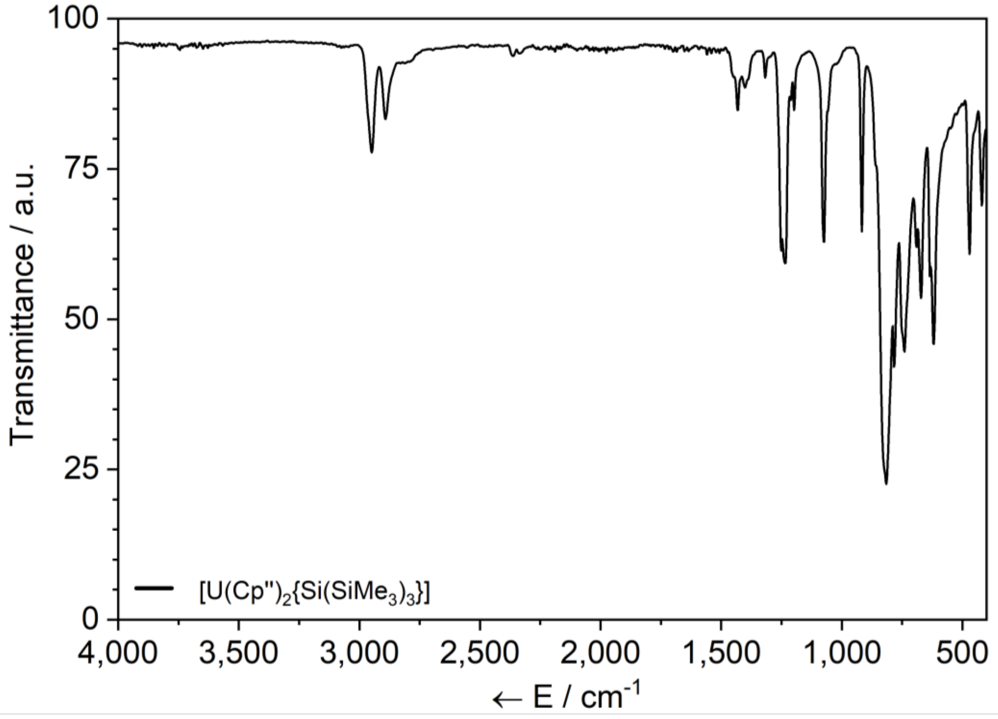

Figure S64. ATR-IR spectrum of **3-U** between 398–4,000 cm^{-1}.

6. UV-Vis-NIR Spectroscopy

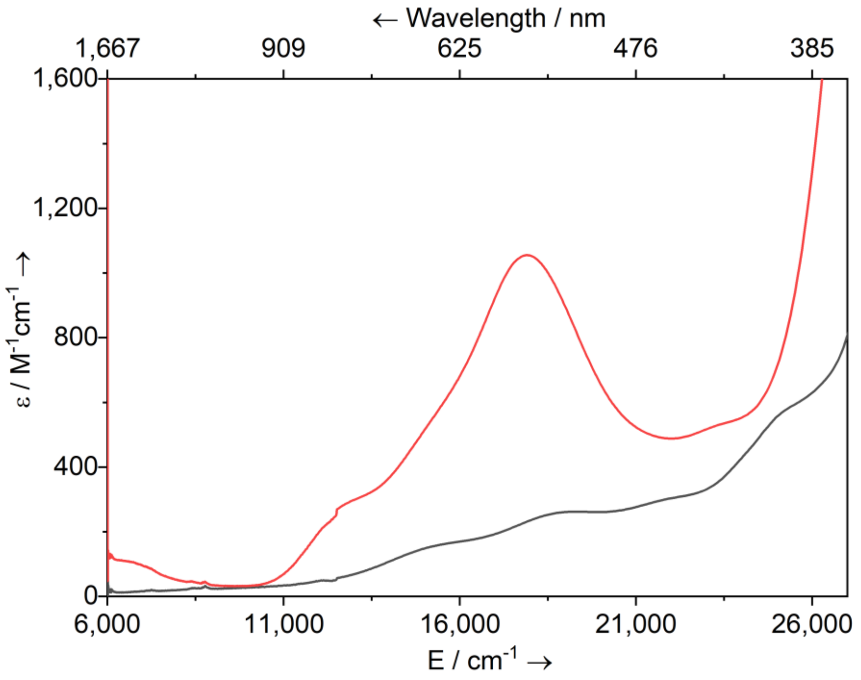

Figure S65. UV-Vis-NIR spectra of **1-Ti** (black) and **2-Zr** (red) in toluene (2 mM) between 6,000-27,000 cm^{-1} (1,667-370 nm).

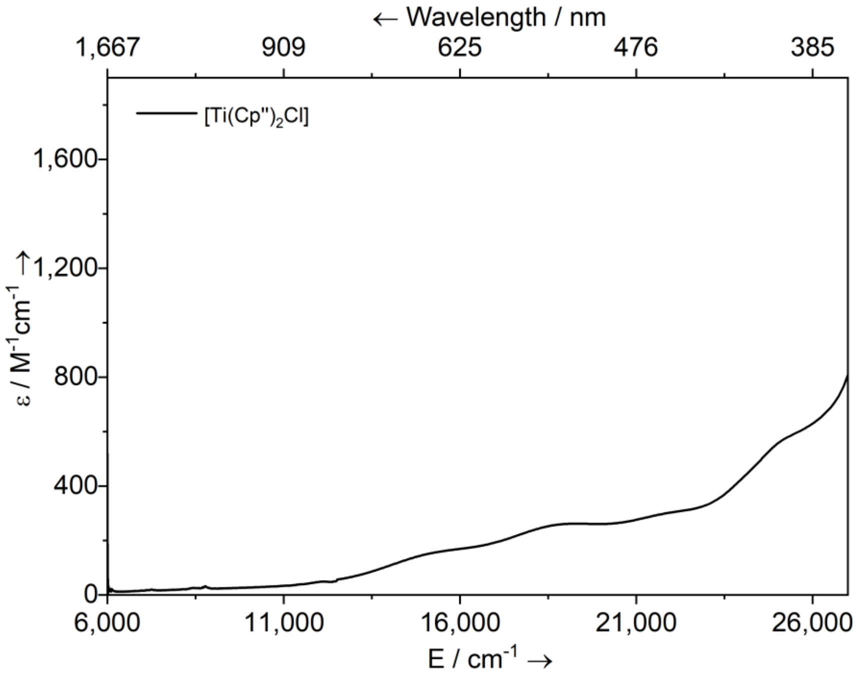

Figure S66. UV-Vis-NIR spectrum of **1-Ti** in toluene (2 mM) between 6,000-27,000 cm^{-1} (1,667-370 nm).

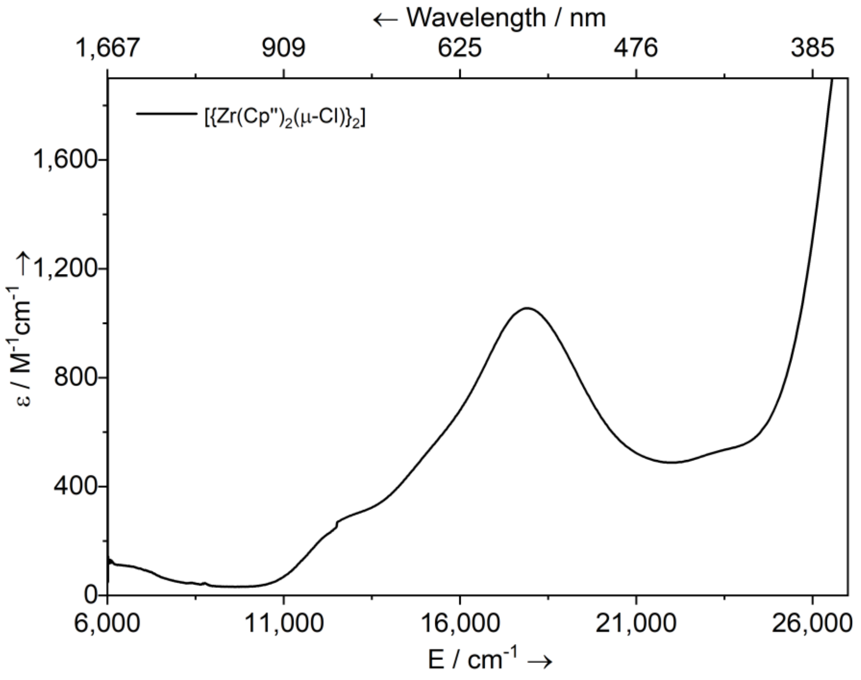

Figure S67. UV-Vis-NIR spectrum of **2-Zr** in toluene (2 mM) between 6,000-27,000 cm^{-1} (1,667-370 nm).

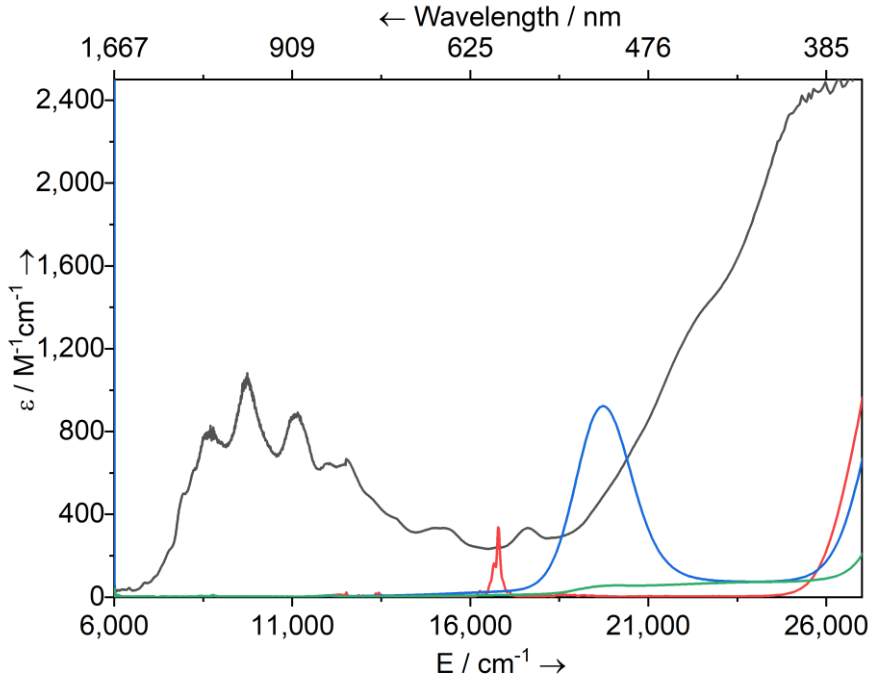

Figure S68. UV-Vis-NIR spectra of **2-M** in toluene (2 mM) between 6,000-27,000 cm^{-1} (1,667-370 nm). Legend: **2-La** (green), **2-Ce** (blue), **2-Nd** (red), **2-U** (black).

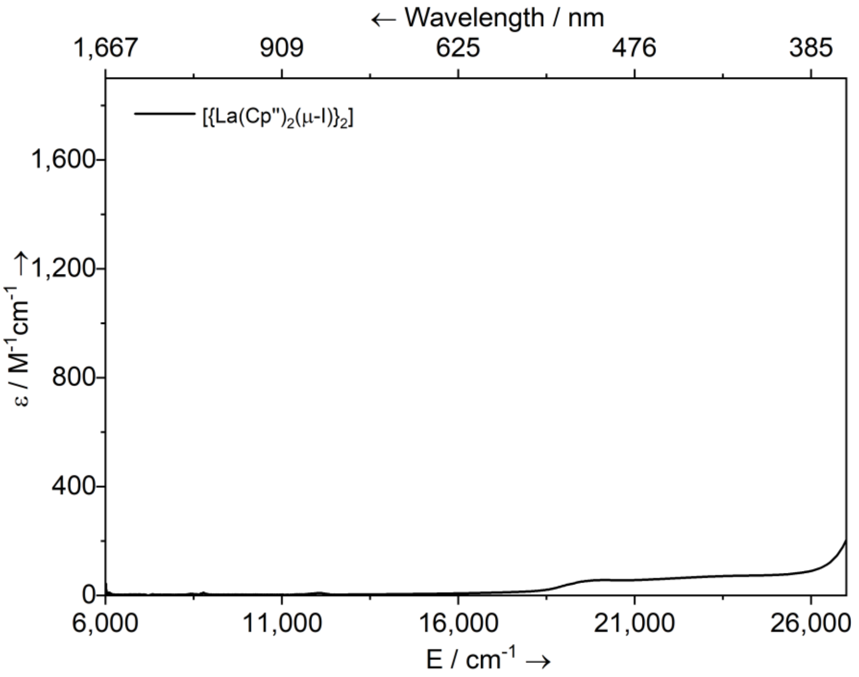

Figure S69. UV-Vis-NIR spectrum of **2-La** in toluene (2 mM) between 6,000-27,000 cm^{-1} (1,667-370 nm).

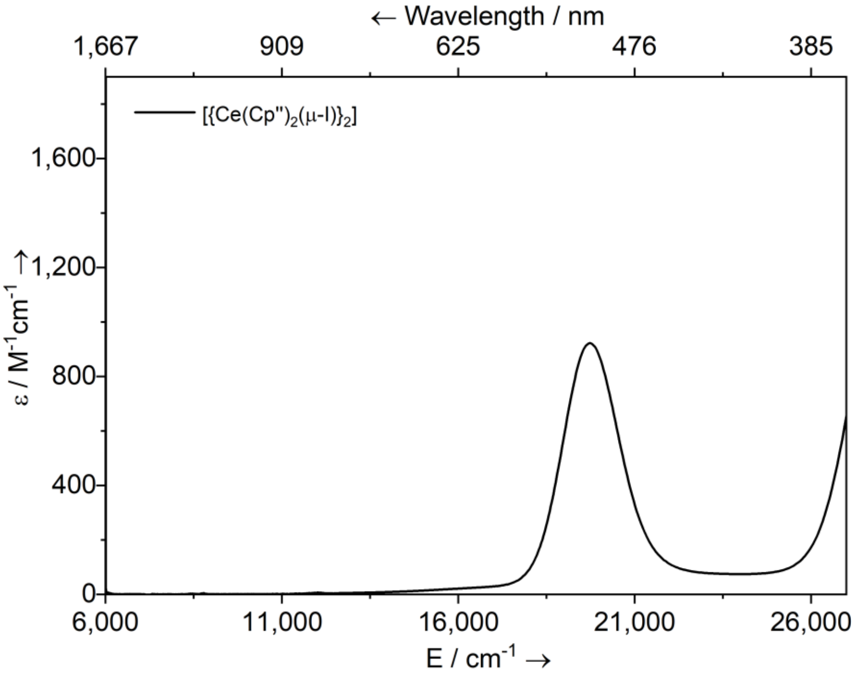

Figure S70. UV-Vis-NIR spectrum of **2-Ce** in toluene (2 mM) between 6,000-27,000 cm^{-1} (1,667-370 nm).

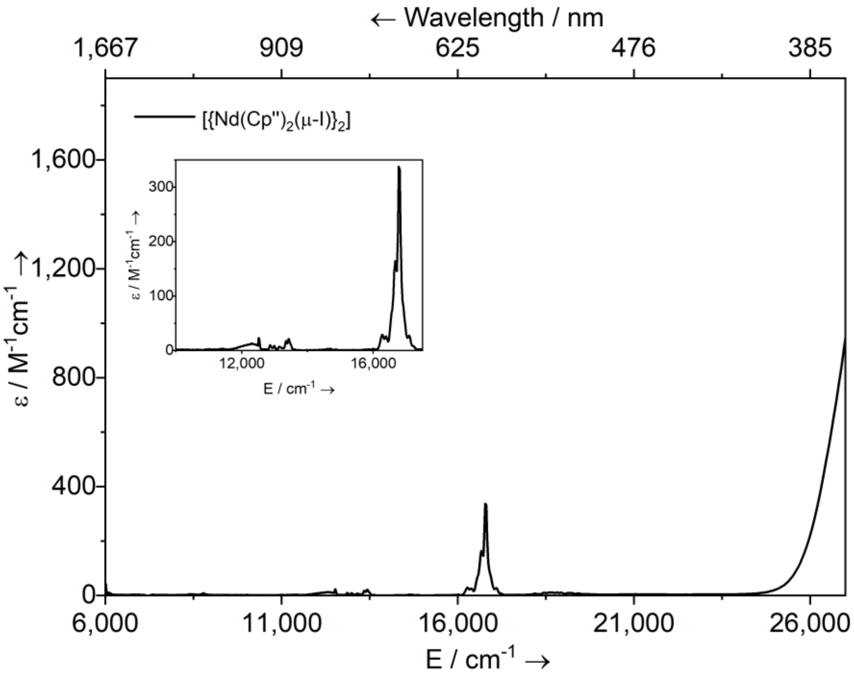

Figure S71. UV-Vis-NIR spectrum of **2-Nd** in toluene (2 mM) between 6,000-27,000 cm^{-1} (1,667-370 nm), inset shows zoomed in region between 10,000–17,500 (1,000-571 nm).

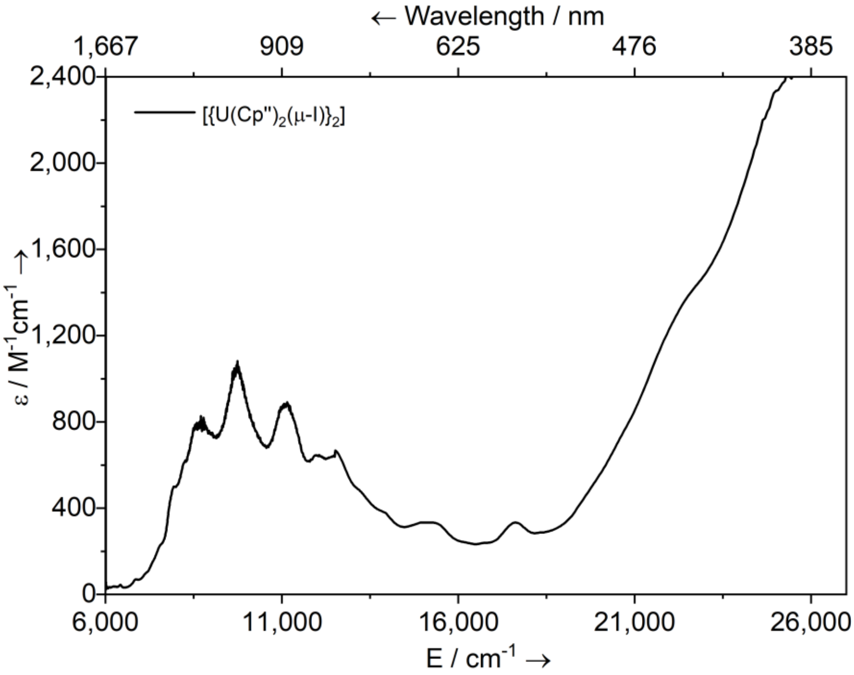

Figure S72. UV-Vis-NIR spectrum of **2-U** in toluene (2 mM) between 6,000-27,000 cm^{-1} (1,667-370 nm).

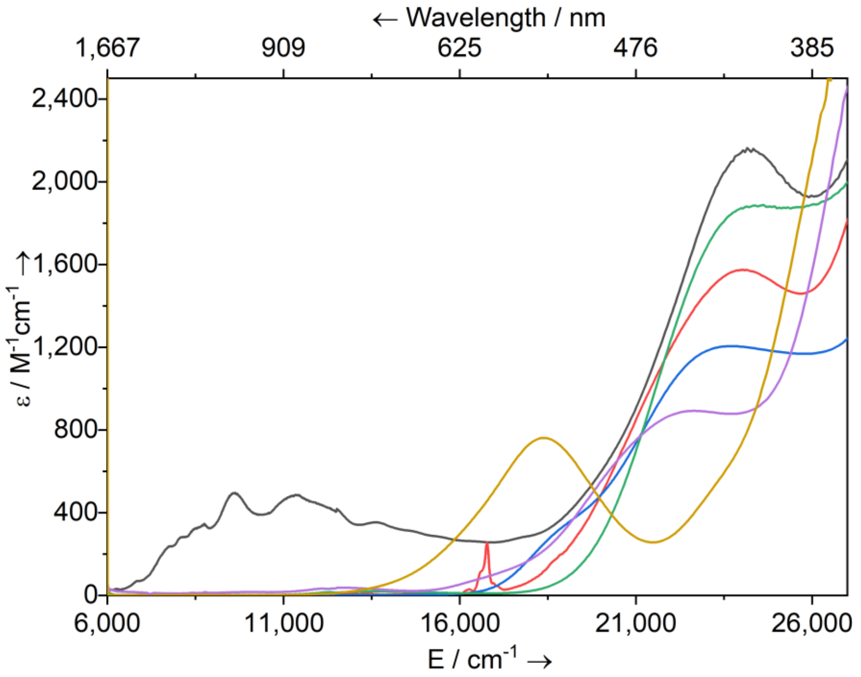

Figure S73. UV-Vis-NIR spectra of **3-M** in toluene (2 mM) between 6,000-27,000 cm^{-1} (1,667-370 nm). Legend: **3-Ti** (orange), **3-Zr** (purple), **3-La** (green), **3-Ce** (blue), **3-Nd** (red), **3-U** (black).

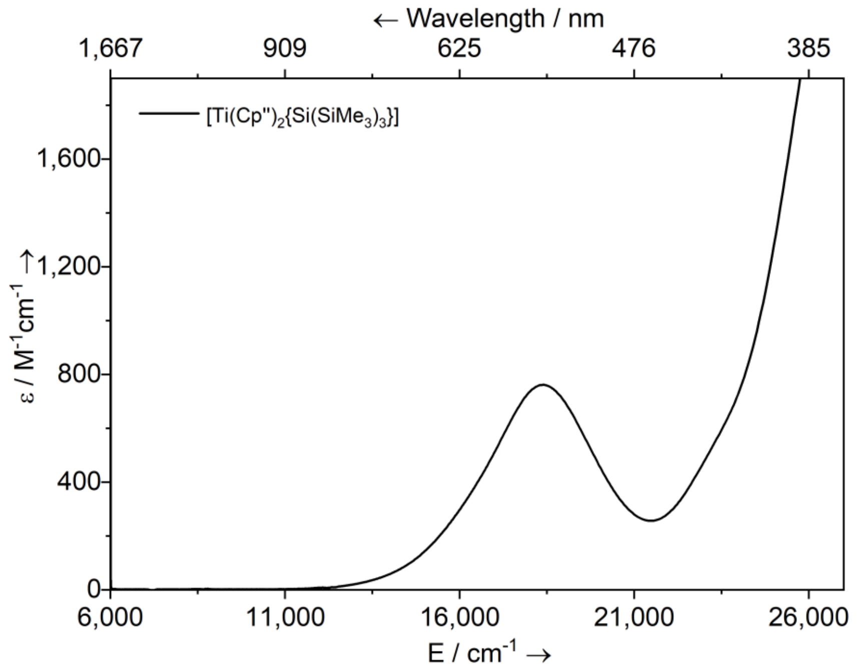

Figure S74. UV-Vis-NIR spectrum of **3-Ti** in toluene (2 mM) between 6,000-27,000 cm^{-1} (1,667-370 nm).

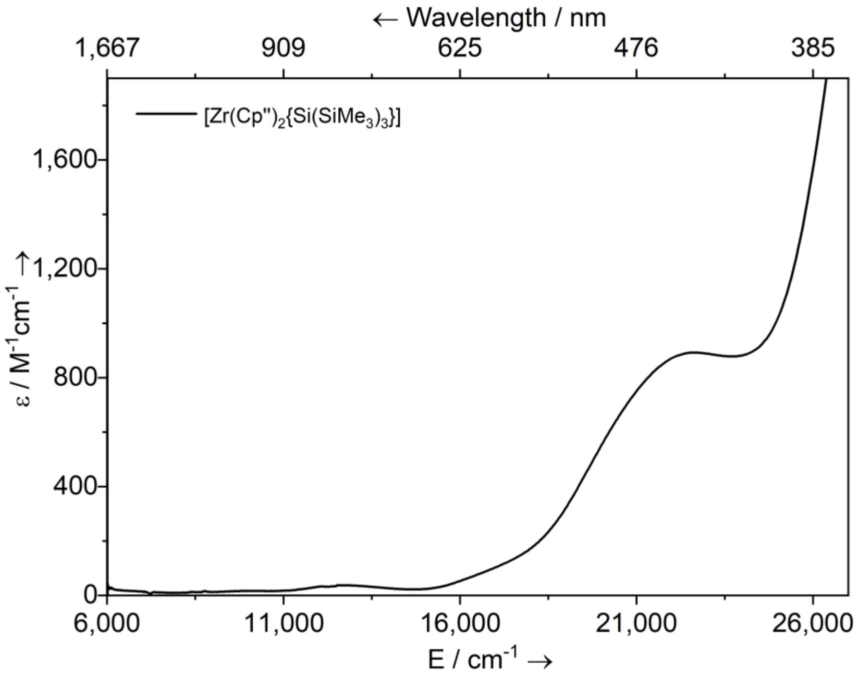

Figure S75. UV-Vis-NIR spectrum of **3-Zr** in toluene (2 mM) between 6,000-27,000 cm^{-1} (1,667-370 nm).

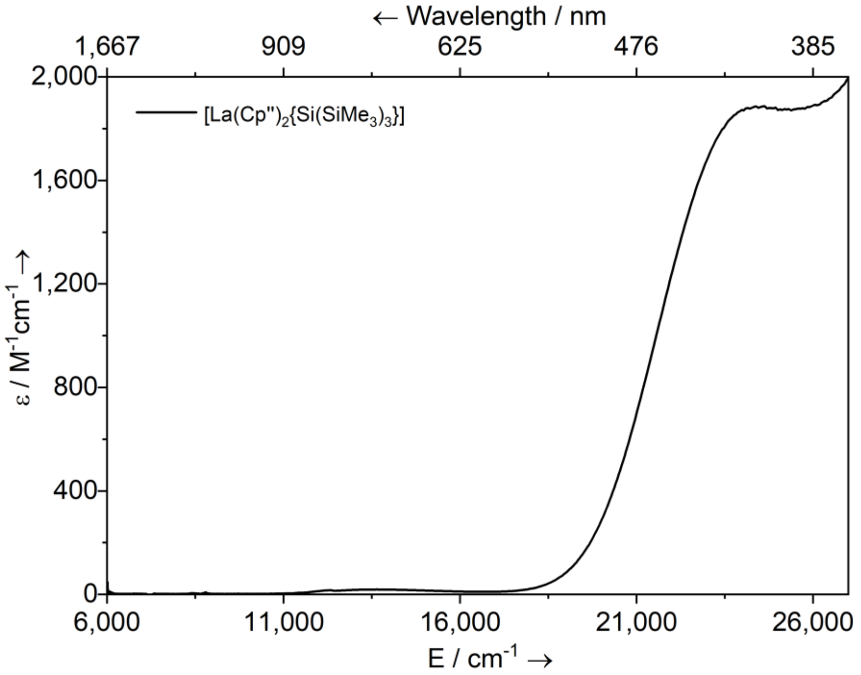

Figure S76. UV-Vis-NIR spectrum of **3-La** in toluene (2 mM) between 6,000-27,000 cm^{-1} (1,667-370 nm).

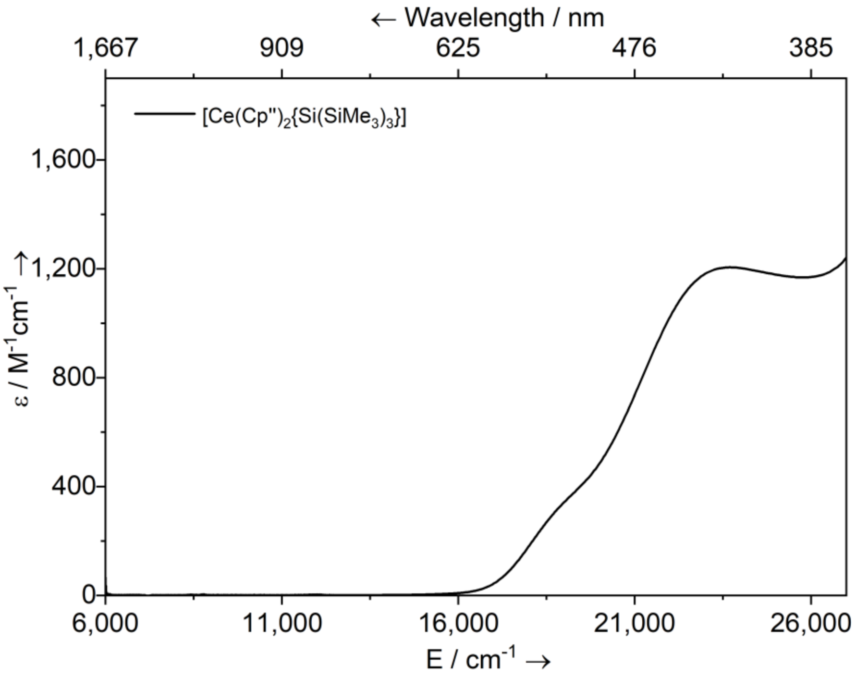

Figure S77. UV-Vis-NIR spectrum of **3-Ce** in toluene (2 mM) between 6,000-27,000 cm^{-1} (1,667-370 nm).

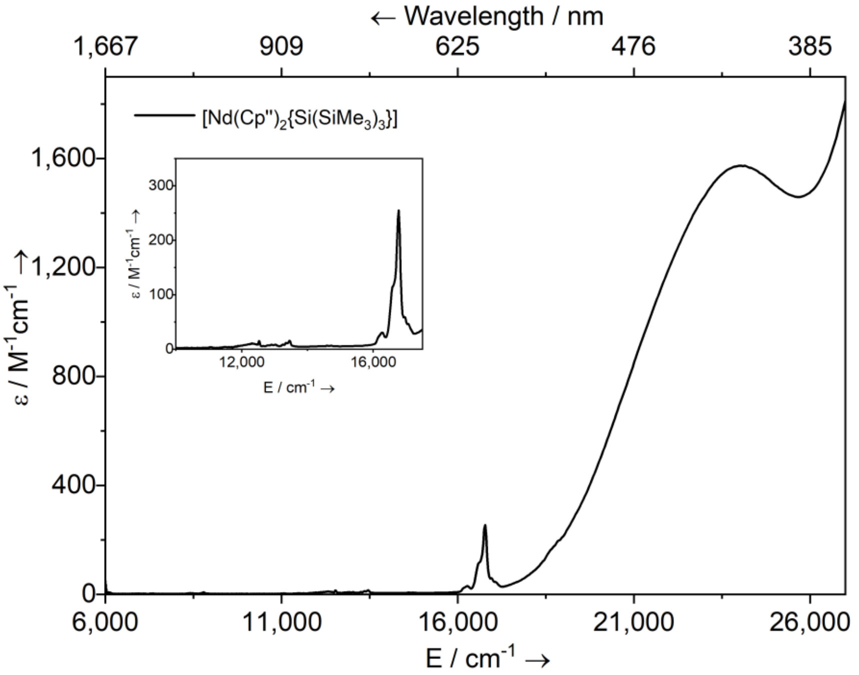

Figure S78. UV-Vis-NIR spectrum of **3-Nd** in toluene (2 mM) between 6,000-27,000 cm^{-1} (1,667-370 nm), inset shows zoomed in region between 10,000–17,500 (1,000-571 nm).

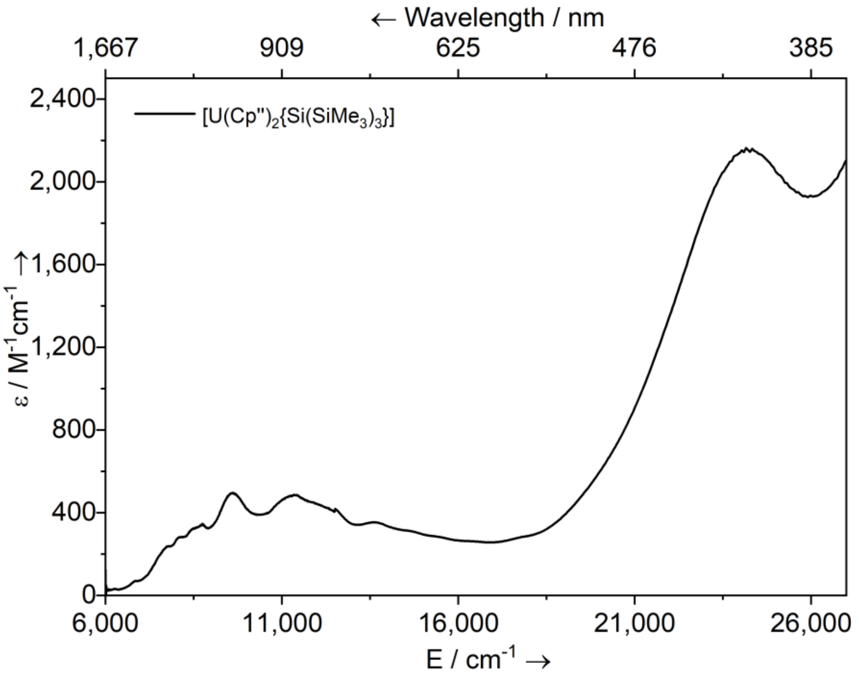

Figure S79. UV-Vis-NIR spectrum of **3-U** in toluene (2 mM) between 6,000-27,000 cm^{-1} (1,667-370 nm).

7. General Crystallographic Methods

The crystallographic data for [Hf(Cp″)$_2$Cl$_2$], **2-Nd**, **2-U**, and **3-M** (M = Ti, Zr, La, Ce, Nd, U) are compiled in Tables S1–S3. Crystals of [Hf(Cp″)$_2$Cl$_2$] were examined using a Rigaku Xcalibur2 diffractometer, equipped with an Atlas CCD area detector and a sealed tube source with graphite-monochromated Mo Kα radiation (λ = 0.71073 Å). Intensities were integrated from a sphere of data recorded on narrow (1°) frames by *ω* rotation. Cell parameters were refined from the observed positions of all strong reflections in each data set. Gaussian grid face-indexed absorption corrections with a beam profile correction were applied. Crystals of **3-La** were examined using an Agilent Supernova diffractometer, equipped with an Eos CCD area detector and a Microfocus source with Mo Kα radiation (λ = 0.71073 Å). Crystals of **2-Nd**, **2-U**, and **3-M** (M = Ti, Zr, Ce, Nd, U) were examined using a Rigaku FR-X diffractometer, equipped with a HyPix 6000HE photon counting pixel array detector with mirror-monochromated Mo Kα (λ = 0.71073 Å) or Cu Kα (λ = 1.5418 Å) radiation. Intensities were integrated from data recorded on 0.5° (**3-U**) or 1° ([Hf(Cp″)$_2$Cl$_2$], **2-Nd**, **2-U**, **3-M** (M = Ti, Zr, La, Ce, Nd)) frames by ω rotation. Cell parameters were refined from the observed positions of all strong reflections in each data set. A Gaussian grid face-indexed with a beam profile was applied for all structures.[23] The structures were solved using SHELXT;[24] the datasets were refined by full-matrix least-squares on all unique F^2 values,[24] with anisotropic displacement parameters for all non-hydrogen atoms, and with constrained riding hydrogen geometries; U_{iso}(H) was set at 1.2 (1.5 for methyl groups) times U_{eq} of the parent atom. The largest features in final difference syntheses were close to heavy atoms and were of no chemical significance. CrysAlis PRO[23] was used for control and integration, and SHELX[24,25] was employed through OLEX2[26] for structure solution and refinement. ORTEP-3[27] and POV-Ray[28] were employed for molecular graphics.

8. Crystallographic Data

Table S1. Crystallographic data for [Hf(Cp″)$_2$Cl$_2$], **2-Nd**, **2-U** and **3-Ti**.

	[Hf(Cp″)$_2$Cl$_2$]	**2-Nd**	**2-U**	**3-Ti**
Formula	$C_{22}H_{42}Cl_2HfSi_4$	$C_{44}H_{84}I_2Nd_2Si_8$	$C_{44}H_{84}I_2Si_8U_2$	$C_{31}H_{69}Si_8Ti$
Fw	668.30	1380.11	1567.69	714.48
cryst size, mm	0.364 x 0.115 x 0.086	0.16 x 0.048 x 0.047	0.26 x 0.072 x 0.051	0.398 x 0.144 x 0.138
cryst syst	Orthorhombic	triclinic	triclinic	monoclinic
space group	Pbcn	P-1	P-1	Cc
collection temperature, K	150(2)	200(2)	100(2)	100(2)
a, Å	17.8746(12)	10.7367(3)	10.6833(4)	10.2297(2)
b, Å	9.0854(5)	11.5169(3)	11.4914(4)	23.3116(4)
c, Å	18.3865(12)	13.3589(4)	13.2297(5)	18.6778(4)
α, °	90	73.257(3)	73.117(4)	90
β, °	90	83.329(2)	83.273(3)	101.213(2)
γ, °	90	78.423(2)	78.875(4)	90
V, Å^3	2985.9(3)	1546.66(8)	1521.72(10)	4369.09(17)
Z	4	1	1	4
ρ_{calcd}, g cm^{-3}	1.487	1.482	1.711	1.086
μ, mm^{-1}	3.841	22.162	24.523	0.434
no. of reflections made	13683	18441	15458	26334
no. of unique reflns, R_{int}	3652, 0.0702	6153, 0.0294	5530, 0.0380	8020, 0.0343
no. of reflns with $F^2 > 2\sigma(F^2)$	2308	5784	5285	7530
transmn coeff range	0.499-0.736	0.137-0.587	0.072-0.739	0.565-1.000
R, R_w[a] ($F^2 > 2\sigma(F^2)$)	0.0533, 0.1095	0.0374, 0.0980	0.0441, 0.1140	0.0279, 0.0696
R, R_w[a] (all data)	0.0964, 0.1303	0.0391, 0.0993	0.0454, 0.1151	0.0310, 0.0708
S[a]	1.064	1.058	1.035	1.033
Parameters, Restraints	138, 0	265, 0	265, 0	383, 2
max., min. diff map, e Å^{-3}	3.405, -0.988	3.273, -1.191	6.777, -2.703	0.365, -0.192

[a] Conventional $R = \Sigma||F_o| - |F_c||/\Sigma|F_o|$; $R_w = [\Sigma w(F_o^2 - F_c^2)^2/\Sigma w(F_o^2)^2]^{1/2}$; $S = [\Sigma w(F_o^2 - F_c^2)^2/\text{no. data} - \text{no. params})]^{1/2}$ for all data.

Table S2. Crystallographic data for **3-Zr**, **3-La**, **3-Ce** and **3-Nd**.

	**3-Zr**	**3-La**	**3-Ce**	**3-Nd**
Formula	$C_{31}H_{69}Si_8Zr$	$C_{31}H_{69}LaSi_8$	$C_{31}H_{69}CeSi_8$	$C_{31}H_{69}NdSi_8$
Fw	757.80	805.49	806.70	810.82
cryst size, mm	0.249 x 0.106 x 0.065	0.323 x 0.074 x 0.028	0.47 x 0.116 x 0.105	0.226 x 0.119 x 0.05
cryst syst	Orthorhombic	monoclinic	monoclinic	monoclinic
space group	$Pna2_1$	$P2_1/n$	$P2_1/n$	$P2_1/n$
collection temperature, K	100(2)	150(2)	100(2)	100(2)
a, Å	37.0378(4)	17.9162(10)	17.8079(6)	17.7679(5)
b, Å	11.77080(10)	13.3218(7)	13.2758(3)	13.2234(3)
c, Å	20.1996(3)	20.3331(16)	20.3529(6)	20.3700(6)
α, °	90	90	90	90
β, °	90	111.465(8)	111.045(3)	110.891(3)
γ, °	90	90	90	90
V, $Å^3$	8806.31(18)	4516.4(5)	4490.8(2)	4471.3(2)
Z	8	4	4	4
ρ_{calcd}, g cm^{-3}	1.143	1.185	1.193	1.204
μ, mm^{-1}	4.260	1.176	10.002	11.038
no. of reflections made	57189	8258	8916	7945
no. of unique reflns, R_{int}	14550, 0.0393	8258, 0.0466	8916, 0.1030	7945, 0.0457
no. of reflns with $F^2 > 2\sigma(F^2)$	13759	6553	8056	7409
transmn coeff range	0.420-1.000	0.569-1.000	0.069-0.861	0.134-0.649
R, $R_w{}^a$ ($F^2 > 2\sigma(F^2)$)	0.0398, 0.1029	0.0604, 0.1375	0.0679, 0.1921	0.0680, 0.2133
R, $R_w{}^a$ (all data)	0.0425, 0.1045	0.0802, 0.1479	0.0712, 0.1936	0.0704, 0.2147
S^a	1.045	1.089	1.068	1.065
Parameters, Restraints	764, 1	491, 948	501, 666	500, 830
max., min. diff map, e $Å^{-3}$	1.684, -0.716	2.796, -1.470	2.909, -1.272	2.074, -1.875

[a] Conventional $R = \Sigma||F_o| - |F_c||/\Sigma|F_o|$; $R_w = [\Sigma w(F_o{}^2 - F_c{}^2)^2/\Sigma w(F_o{}^2)^2]^{1/2}$; $S = [\Sigma w(F_o{}^2 - F_c{}^2)^2/\text{no. data} - \text{no. params})]^{1/2}$ for all data.

Table S3. Crystallographic data for **3-U**.

	**3-U**
Formula	$C_{31}H_{69}Si_8U$
Fw	904.61
cryst size, mm	0.151 x 0.046 x 0.033
cryst syst	monoclinic
space group	$P2_1/n$
collection temperature, K	150(2)
a, Å	17.9833(8)
b, Å	13.2292(5)
c, Å	20.4047(13)
α, °	90
β, °	111.407(6)
γ, °	90
V, $Å^3$	4519.5(4)
Z	4
ρ_{calcd}, g cm^{-3}	1.329
μ, mm^{-1}	12.266
no. of reflections made	43377
no. of unique reflns, R_{int}	8268, 0.0961
no. of reflns with $F^2 > 2\sigma(F^2)$	6715
transmn coeff range	0.662-1.000
R, R_w[a] ($F^2 > 2\sigma(F^2)$)	0.0503, 0.1228
R, R_w[a] (all data)	0.0621, 0.1288
S[a]	1.033
Parameters, Restraints	500, 168
max., min. diff map, e $Å^{-3}$	1.059, -2.289

[a] Conventional $R = \Sigma||F_o| - |F_c||/\Sigma|F_o|$; $R_w = [\Sigma w(F_o^2 - F_c^2)^2/\Sigma w(F_o^2)^2]^{1/2}$; $S = [\Sigma w(F_o^2 - F_c^2)^2/\text{no. data} - \text{no. params})]^{1/2}$ for all data.

9. Molecular Structures

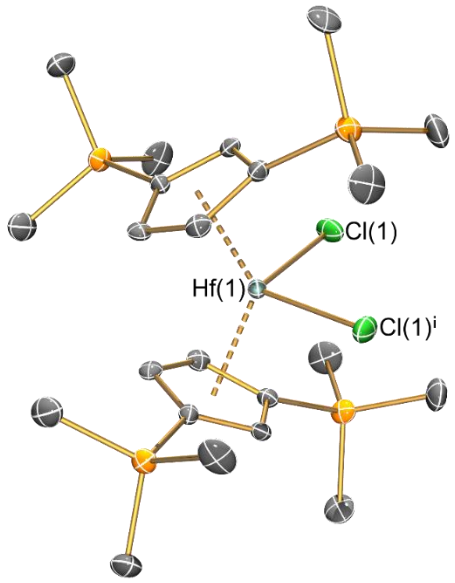

Figure S80. Molecular structure of [Hf(Cp″)$_2$Cl$_2$] determined at 150 K, with selective atom labelling. Displacement ellipsoids set at 30% probability level and hydrogen atoms removed for clarity. Symmetry operation (i) = 1–*x*, +*y*, 1/2–*z*.

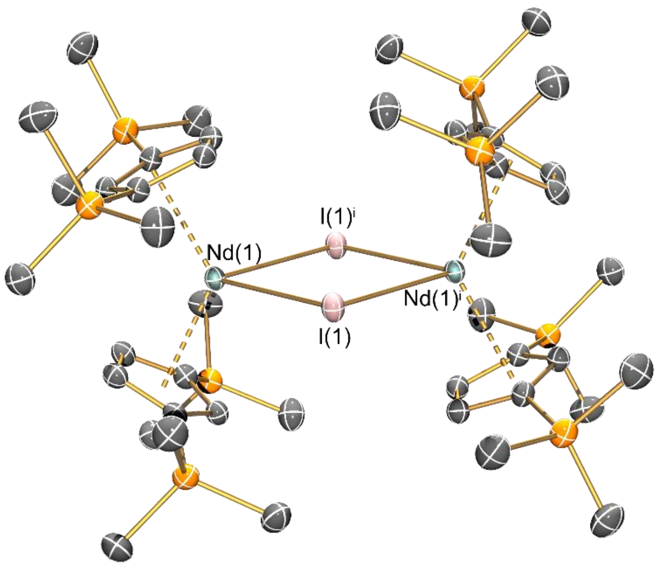

Figure S81. Molecular structure of **2-Nd** determined at 200 K, with selective atom labelling. Displacement ellipsoids set at 30% probability level and hydrogen atoms removed for clarity. Symmetry operation (i) = 1–*x*, 1–*y*, 1–*z*.

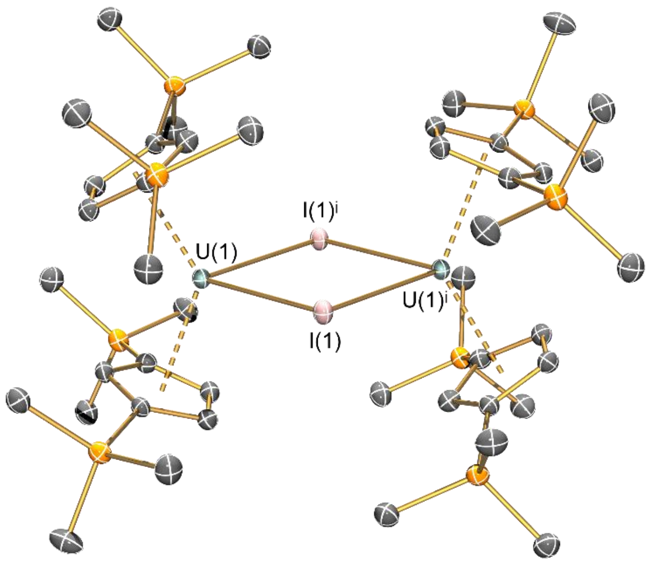

Figure S82. Molecular structure of **2-U** determined at 100 K, with selective atom labelling. Displacement ellipsoids set at 30% probability level and hydrogen atoms removed for clarity. Symmetry operation (i) = 1–*x*, 1–*y*, –*z*.

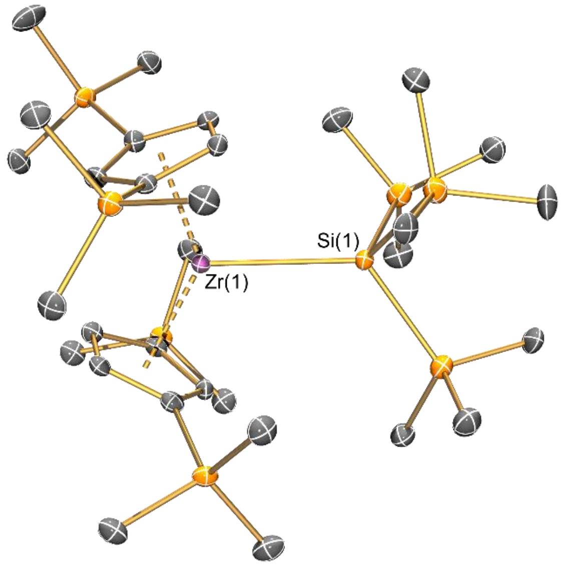

Figure S83. Molecular structure of **3-Zr** determined at 100 K, with selective atom labelling. Displacement ellipsoids set at 30% probability level and hydrogen atoms removed for clarity.

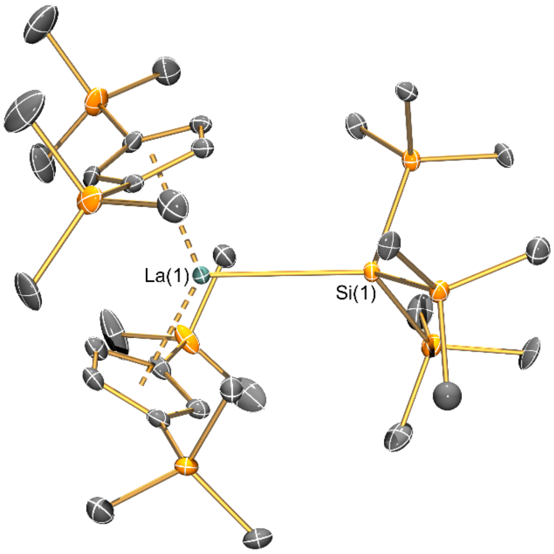

Figure S84. Molecular structure of **3-La** determined at 150 K, with selective atom labelling. Displacement ellipsoids set at 30% probability level and hydrogen atoms removed for clarity.

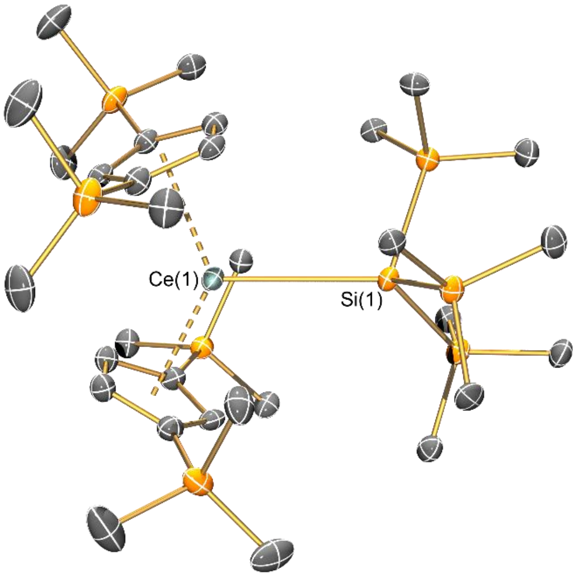

Figure S85. Molecular structure of **3-Ce** determined at 100 K, with selective atom labelling. Displacement ellipsoids set at 30% probability level and hydrogen atoms removed for clarity.

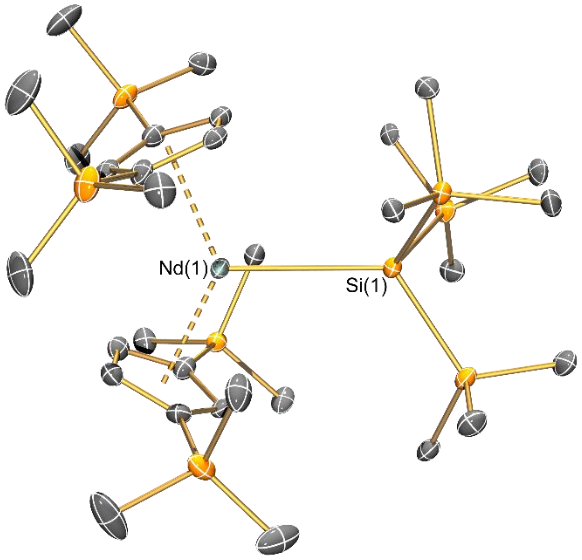

Figure S86. Molecular structure of **3-Nd** determined at 100 K, with selective atom labelling. Displacement ellipsoids set at 30% probability level and hydrogen atoms removed for clarity.

10. Static SQUID Magnetometry

Magnetic measurements were performed on a Quantum Design MPMS3 superconducting quantum interference device (SQUID) magnetometer. Finely ground powder samples (30–40 mg, Table S4) were restrained in eicosane (15–20 mg, Table S4) and flame sealed in a borosilicate tube under vacuum. Sealed samples were loaded into plastic straws and held in place by friction between diamagnetic tape at the top of the tube and the straw. Raw magnetic data were scaled for the shape of the sample using a Quantum Design MPMS3 Geometry Correction Simulator, corrected for the diamagnetic contribution of the sample holder (straw + borosilicate tube), and corrected for the mass of eicosane. The molar susceptibility was corrected for the intrinsic diamagnetic contribution of the sample, estimated as the molecular weight (g mol^{-1}) multiplied by 0.5×10^{-6} cm^3 K mol^{-1}; for weakly paramagnetic **3-Zr** the diamagnetic contribution was corrected for by subtracting the moment measured for a sample of **3-La** measured under the same conditions.

The equilibrium susceptibility was measured in temperature settle mode on cooling under a static applied field. Due to the insulating nature of the borosilicate tube, especially at low temperatures, the cooling rate must be kept slow to allow for complete thermalisation of the sample; the sequence used was optimised on a $GdCl_3$ sample in a borosilicate tube. From 300 to 100 K, measurements were recorded every 20 K with a cooling rate of 5 K min^{-1}. From 90 to 60 K, measurements were recorded every 10 K with a cooling rate of 2 K min^{-1}. From 50 to 1.8 K, 21 points were measured, evenly spaced in log *T*. For 50–10 K and 10–1.8 K, the cooling rates were 2 and 1 K min^{-1}, respectively. Additional delays were required below 3 K: 2 min at 2.96 K, 4 min at 2.51 K, 10 min at 2.13 K and 18 min at 1.80 K. Equilibrium magnetisation *vs*. field measurements were performed in settle mode at 2 and 4 K and 0 to 7 T. The field sweep rate was 500 Oe s^{-1} and at each field point (every 2 kOe from 0–10 kOe, every 5 kOe from 20–70 kOe), time delays of 10 min (2 K) or 5 min (4 K) were applied.

Measurements on a $GdCl_3$ sample in borosilicate glass indicated these delays were necessary to reach equilibrium.

Two measurement methods are available in the Quantum Design MPMS3 SQUID magnetometer. The vibrating sample magnetometer (VSM) mode can accurately measure weak magnetic moments and provide simple and accurate background corrections; however, this is susceptible to frictional heating, particularly at low temperatures, and shape corrections can vary significantly from unity. The direct current (DC) scan mode is less susceptible to frictional heating, and sample shape corrections do not vary as much, but it is unable to accurately measure weakly magnetic samples without complex background corrections involving voltage curve subtractions, impacting the accuracy of susceptibility curves at high temperature in particular. As a result, we have chosen to use a mixture of DC and VSM measurement methods depending on the sample.

For highly magnetic **3-Nd** and **3-U** samples all measurements were performed in DC scan mode with 40 mm scan length and 6 s scan time; susceptibility measurements were performed in 1 kOe static field. For the intermediate magnetic **3-Ce** sample, magnetisation *vs*. field measurements were performed in DC scan mode with 40 mm scan length and 6 s scan time; susceptibility measurements were performed in 1 kOe static field in VSM mode with 3 mm amplitude and 5 s averaging time. For weakly magnetic **1-Ti** and **3-Ti**, magnetisation *vs*. field measurements were performed in DC scan mode with 30 mm scan length and 4 s scan time; susceptibility measurements were performed in 2 kOe static field in VSM mode with 3 mm amplitude and 5 s averaging time. For weakly magnetic **3-Zr** and **3-La**, both susceptibility (2 kOe) and magnetisation measurements were performed in VSM mode with 3 mm amplitude and 5 s averaging time. Complex **2-Zr** appeared diamagnetic below 50 K and reliable measurements could not be obtained in this temperature range, therefore susceptibility (10 kOe) was performed in VSM mode with 5 mm amplitude and 5 s averaging time.

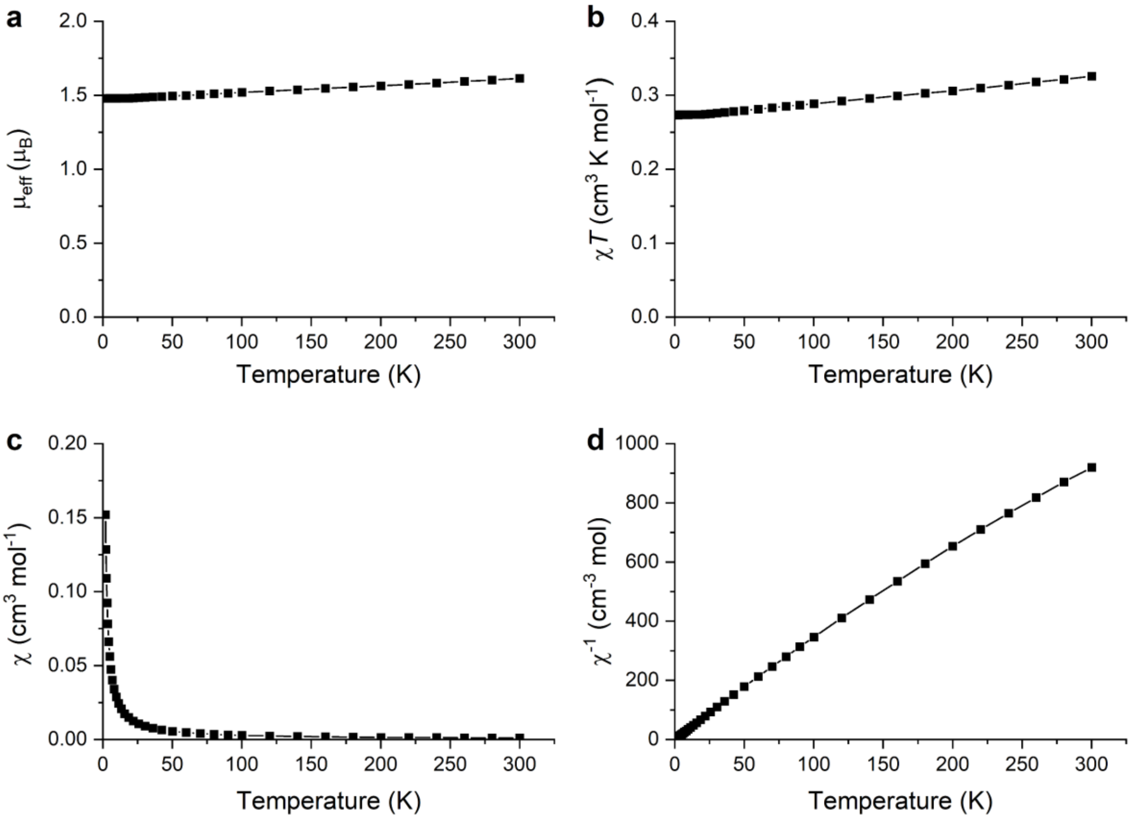

Figure S87. Variable-temperature SQUID magnetic data for powdered **1-Ti** in a 0.2 T applied magnetic field, presented as: (a) μ_{eff} *vs.* T, (b) χT *vs.* T, (c) χ *vs.* T, (d) χ^{-1} *vs.* T.

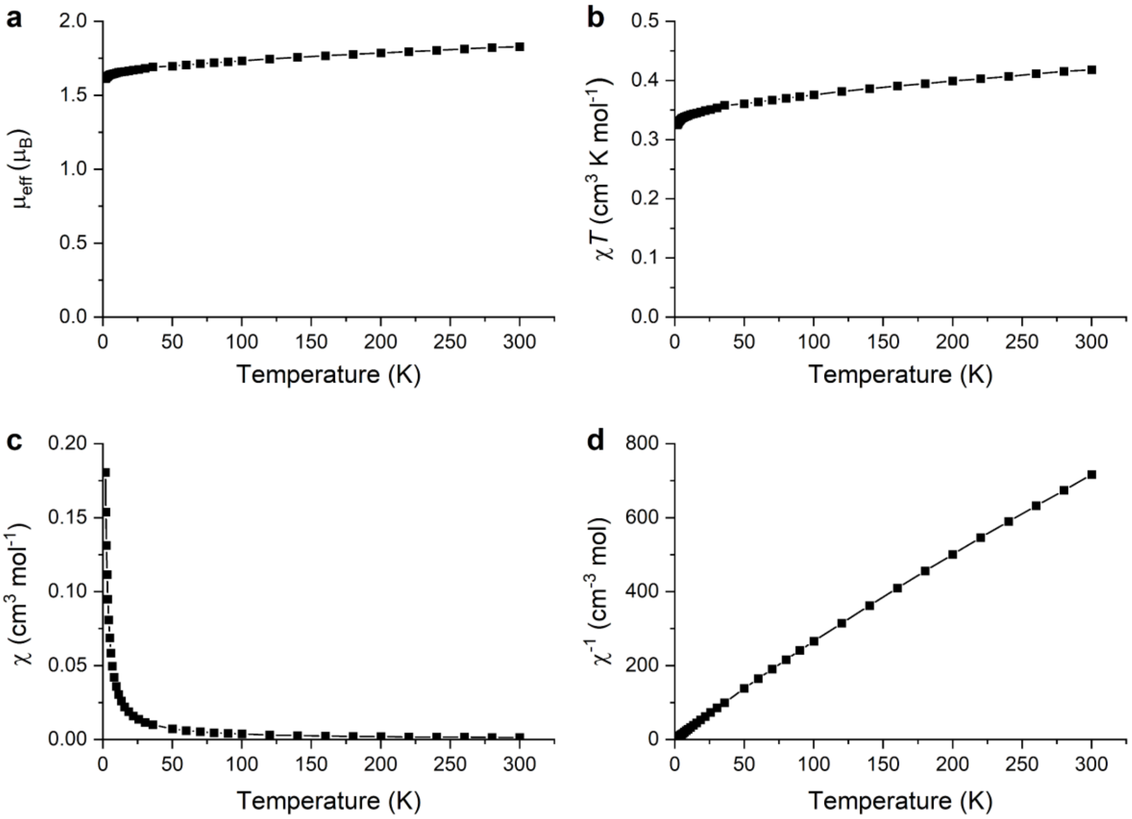

Figure S88. Variable-temperature SQUID magnetic data for powdered **3-Ti** in a 0.2 T applied magnetic field, presented as: (a) μ_{eff} *vs.* T, (b) χT *vs.* T, (c) χ *vs.* T, (d) χ^{-1} *vs.* T.

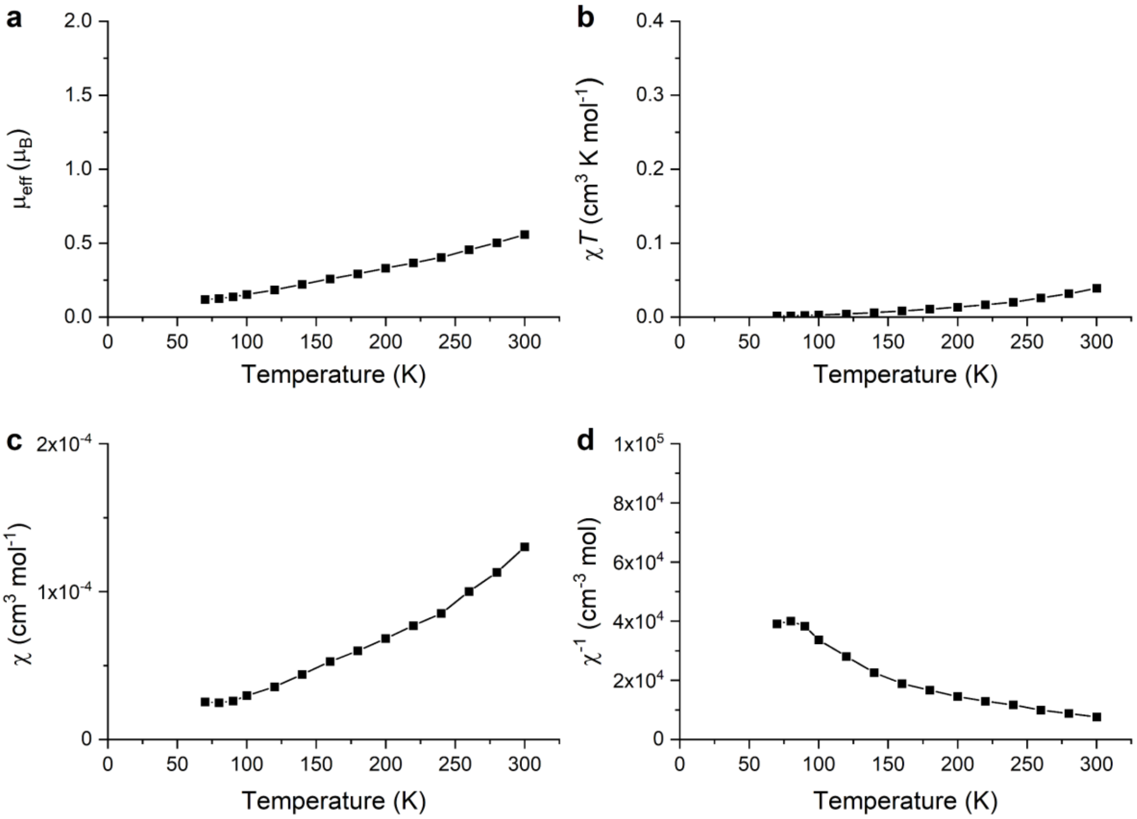

Figure S89. Variable-temperature SQUID magnetic data for powdered **2-Zr** in a 1.0 T applied magnetic field, presented as: (a) μ_{eff} *vs.* T, (b) χT *vs.* T, (c) χ *vs.* T, (d) χ^{-1} *vs.* T. Measurements below 50 K were unstable, seemingly because the sample moment was vanishingly small compared to the background signal.

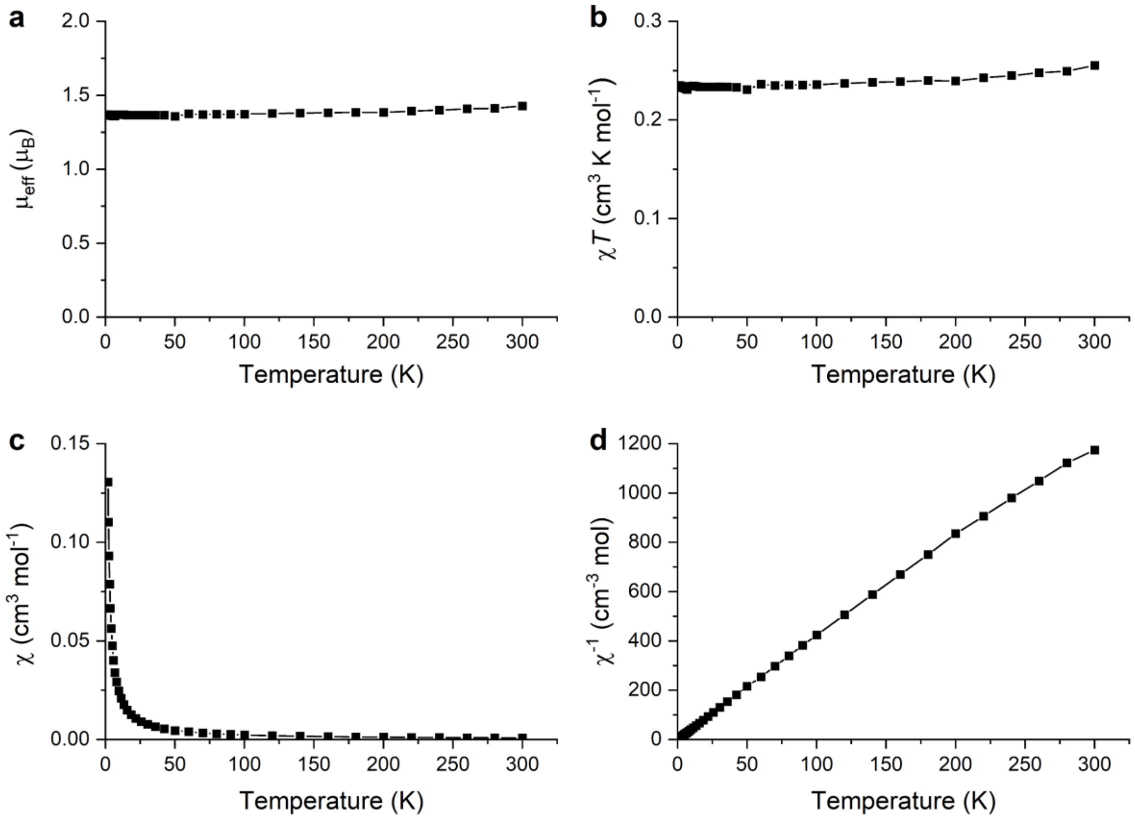

Figure S90. Variable-temperature SQUID magnetic data for powdered **3-Zr** in a 0.2 T applied magnetic field, presented as: (a) μ_{eff} *vs.* T, (b) χT *vs.* T, (c) χ *vs.* T, (d) χ^{-1} *vs.* T.

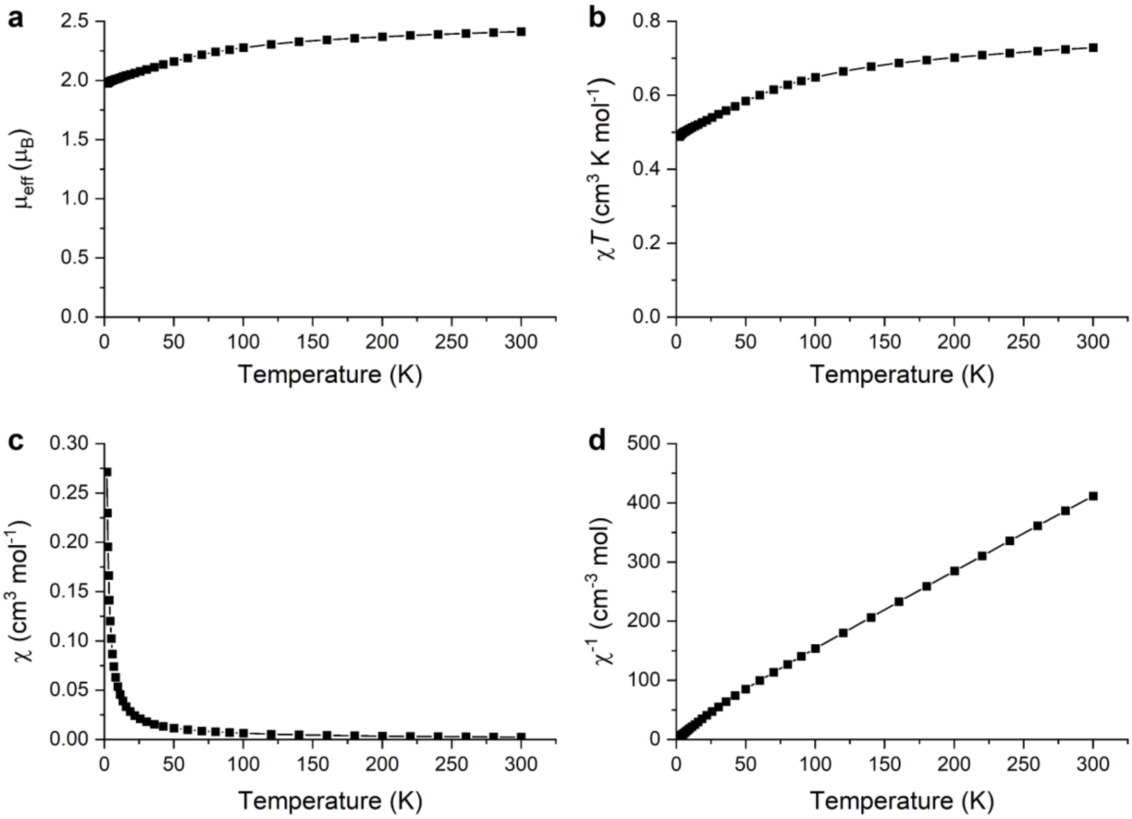

Figure S91. Variable-temperature SQUID magnetic data for powdered **3-Ce** in a 0.1 T applied magnetic field, presented as: (a) μ_{eff} *vs.* T, (b) χT *vs.* T, (c) χ *vs.* T, (d) χ^{-1} *vs.* T.

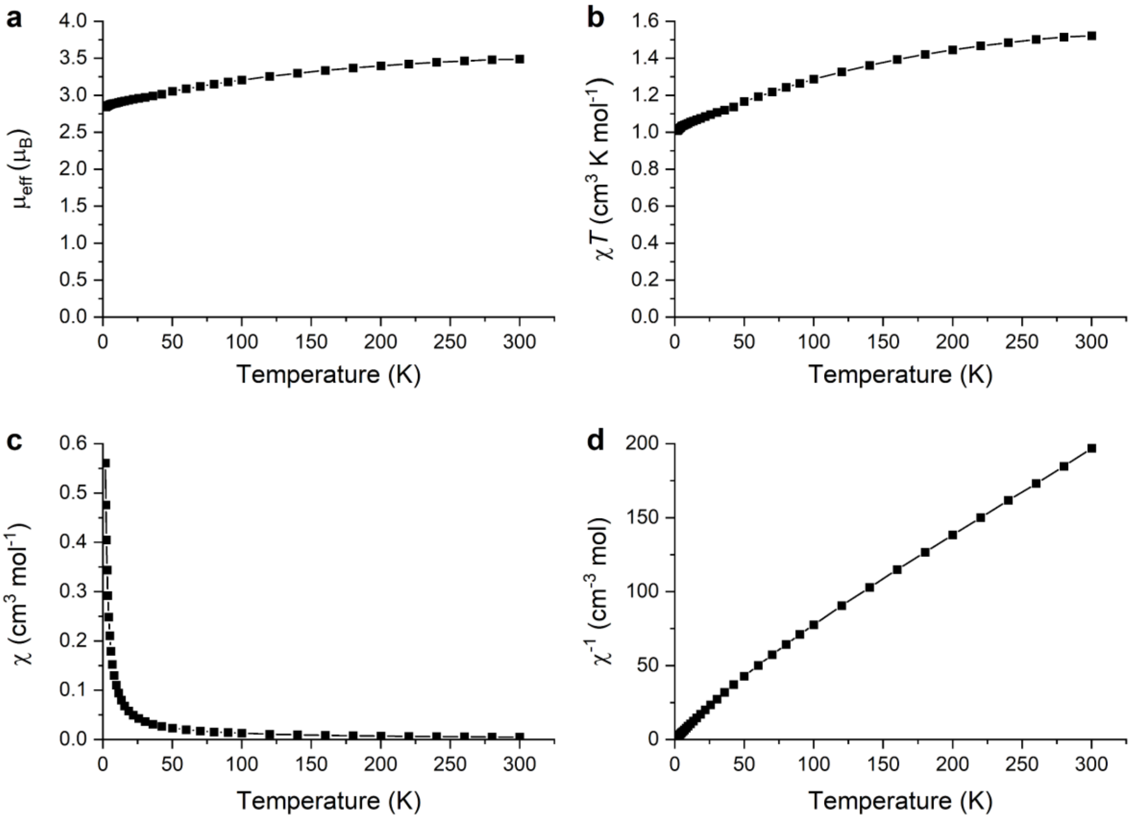

Figure S92. Variable-temperature SQUID magnetic data for powdered **3-Nd** in a 0.1 T applied magnetic field, presented as: (a) μ_{eff} *vs.* T, (b) χT *vs.* T, (c) χ *vs.* T, (d) χ^{-1} *vs.* T.

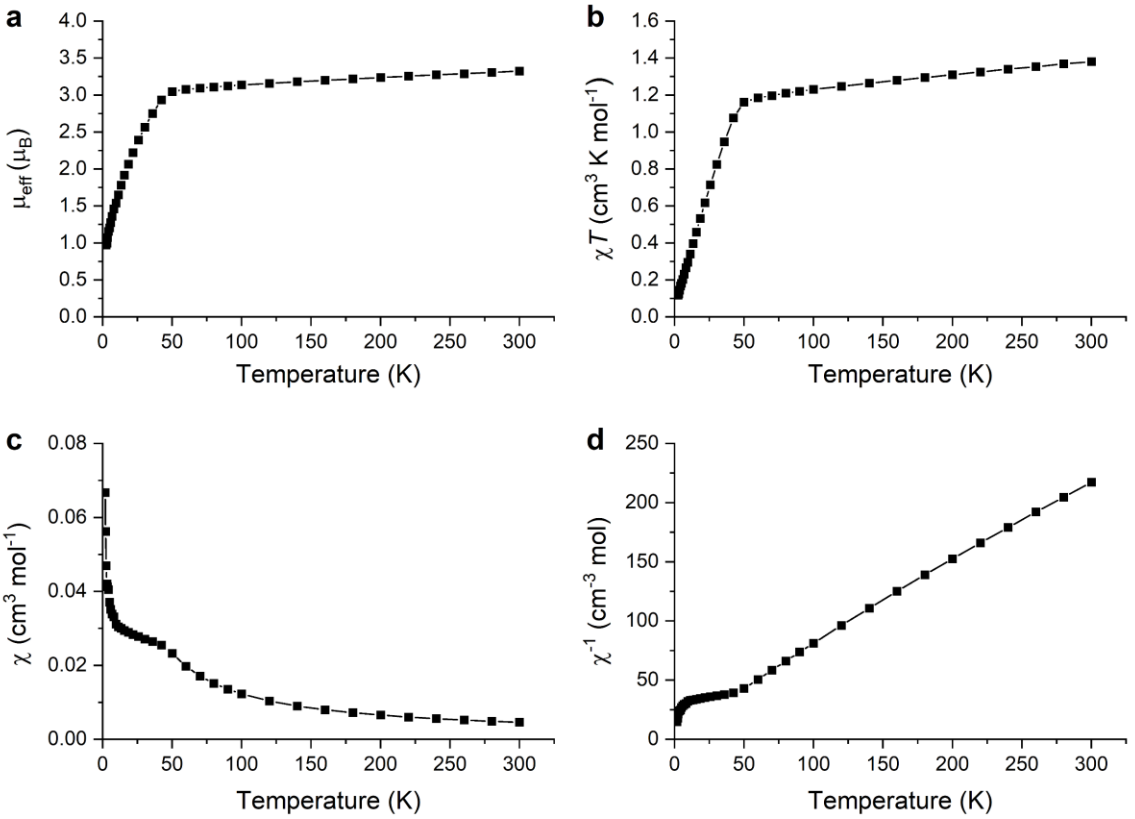

Figure S93. Variable-temperature SQUID magnetic data for powdered **3-U** in a 0.1 T applied magnetic field, presented as: (a) μ_{eff} *vs.* T, (b) χT *vs.* T, (c) χ *vs.* T, (d) χ^{-1} *vs.* T. The increase in χ at the lowest temperatures reflects the slower sweep rate below 10 K (1 K min^{-1}) and time delays before measuring the lowest four points.

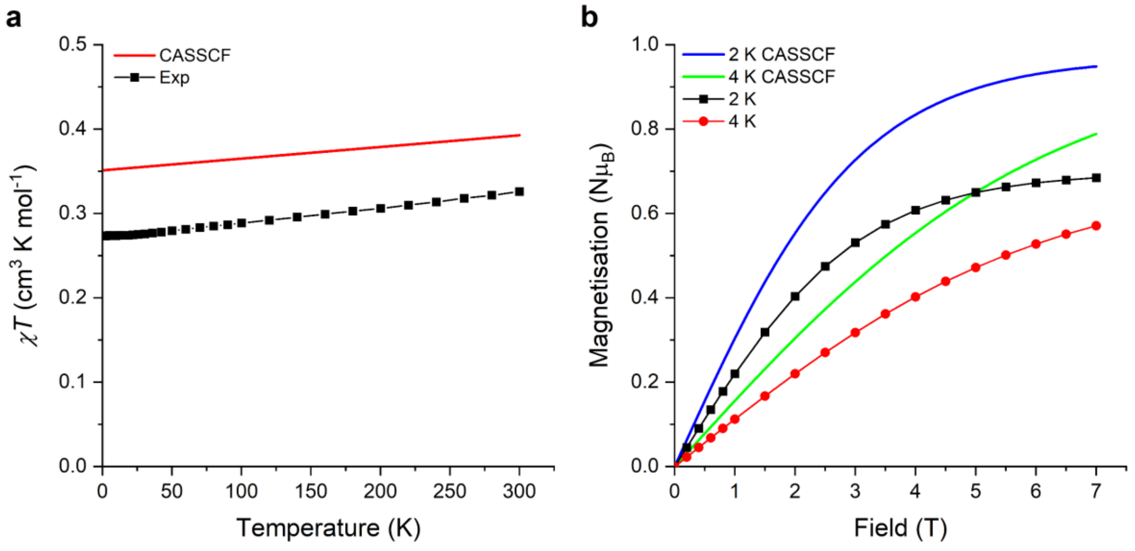

Figure S94. Comparison of SQUID magnetic data with CASSCF calculated trace for **1-Ti** showing: (a) χT *vs.* T, (b) magnetisation *vs.* field.

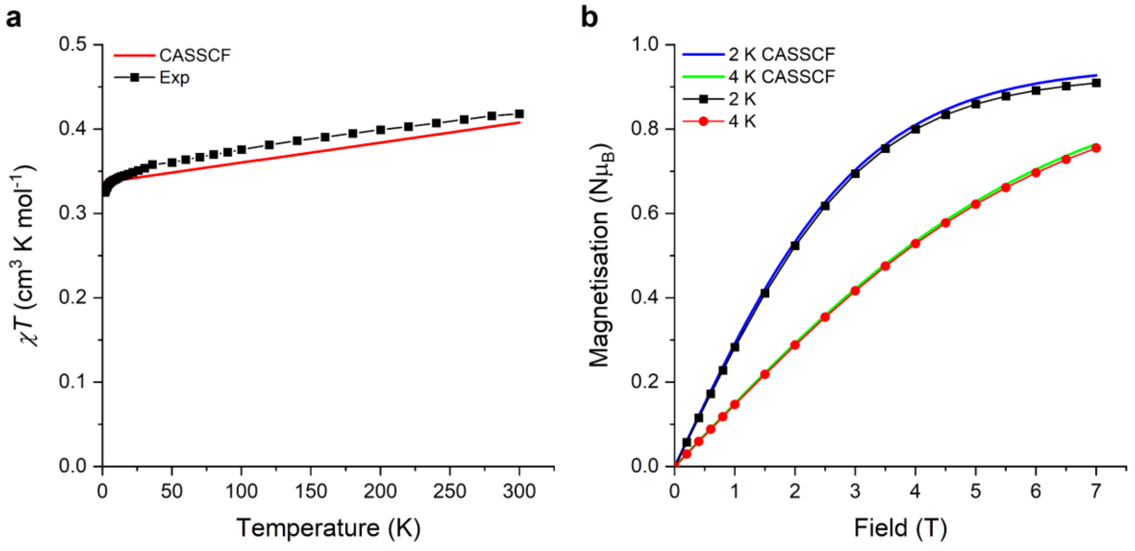

Figure S95. Comparison of SQUID magnetic data with CASSCF calculated trace for **3-Ti** showing: (a) χT *vs.* T, (b) magnetisation *vs.* field.

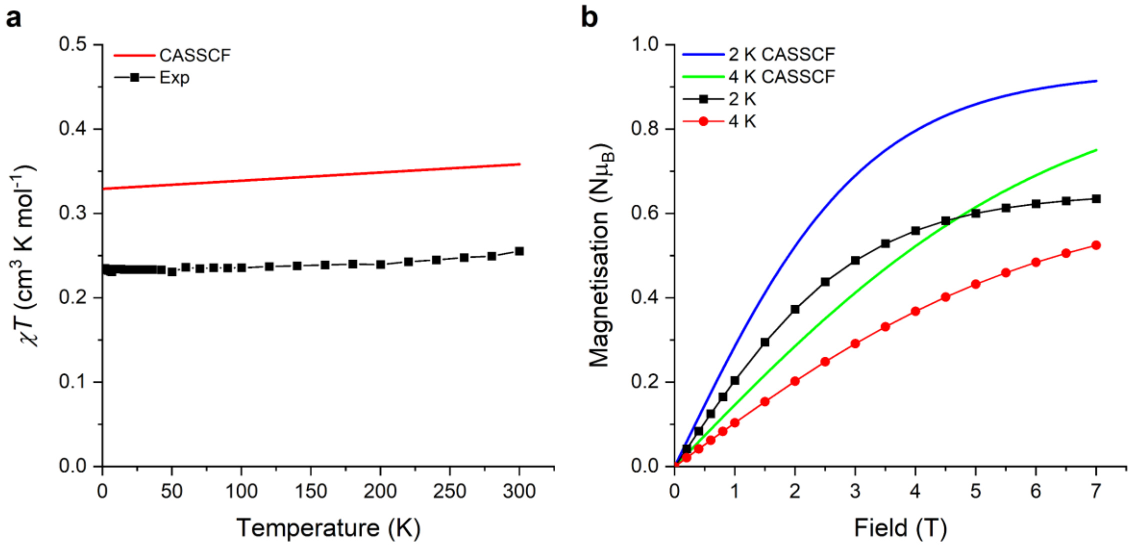

Figure S96. Comparison of SQUID magnetic data with CASSCF calculated trace for **3-Zr** showing: (a) χT *vs.* T, (b) magnetisation *vs.* field.

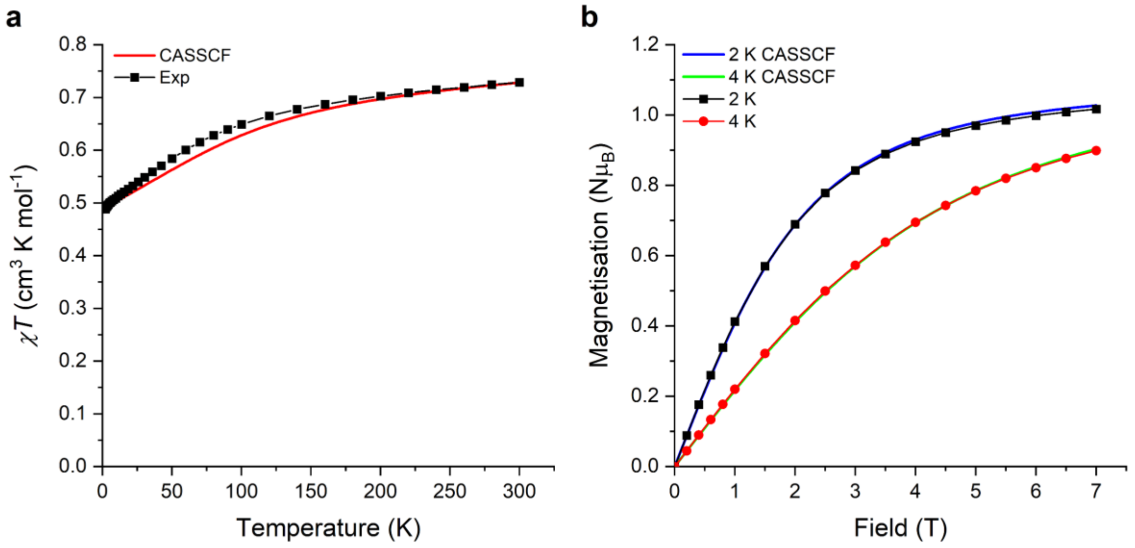

Figure S97. Comparison of SQUID magnetic data with CASSCF calculated trace for **3-Ce** showing: (a) χT *vs.* T, (b) magnetisation *vs.* field.

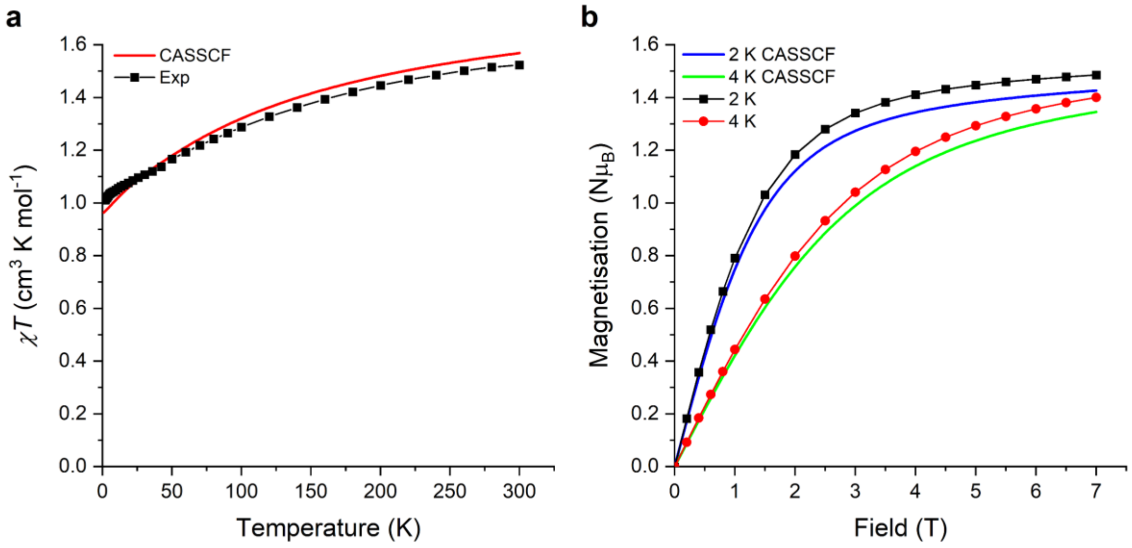

Figure S98. Comparison of SQUID magnetic data with CASSCF calculated trace for **3-Nd** showing: (a) χT *vs.* T, (b) magnetisation *vs.* field.

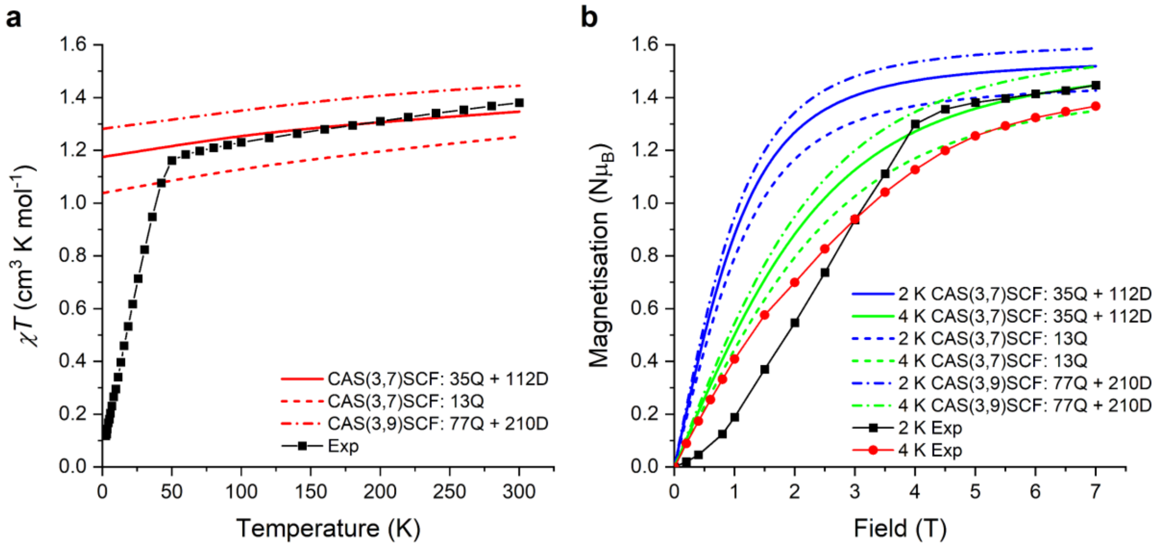

Figure S99. Comparison of SQUID magnetic data with CASSCF calculated trace for **3-U** showing: (a) χT *vs.* T, (b) magnetisation *vs.* field. Step in 2 K data reflects slow relaxation of **3-U**. Three calculations for **3-U** are shown: CAS(3,7)SCF averaging over 35 spin quartets and 112 spin doublets (solid line), CAS(3,7)SCF averaging over 13 spin quartets (dashed line) and CAS(3,9)SCF averaging over 77 spin quartets and 210 spin doublets (dash-dotted line).

Table S4. Experimental details pertaining to the SQUID analysis of **1-3-M** presented in this work.

Complex	Molecular Weight (g mol^{-1})	Sample Mass (mg)	Eicosane mass (mg)	Shape correction factor, DC [a]	Shape correction factor, VSM [a]
**1-Ti**	502.24	36.9	17.4	0.975	0.936
**3-Ti**	744.51	34.8	16.2	0.969	0.931
**2-Zr**	1151.32	35.6	16.0	n/a	1.008
**3-Zr**	787.87	32.4	17.1	n/a	0.935
**3-Ce**	806.69	36.3	17.2	0.951	0.882
**3-Nd**	840.89	38.9	19.3	0.991	n/a
**3-U**	904.6	36.4	17.2	0.997	n/a

a = Calculated using height and diameter of cylindrical sample using MPMS3 Geometry Correction Simulator.

Table S5. Magnetisation saturation values ($N\mu_B$) at 2 K and 7 T, measured by SQUID magnetometry, determined by CASSCF calculations.

Complex	SQUID Magnetometry	CASSCF Calculations
**1-Ti**	0.68	0.95
**3-Ti**	0.91	0.93
**3-Zr**	0.64	0.91
**3-Ce**	1.02	1.03
**3-Nd**	1.49	1.43
**3-U**	1.45	1.52[a]

a = CAS(3,7) averaging over 35 spin quartets and 112 spin doublets (all f^3 configurations)

11. Dynamic SQUID Magnetometry on 3-U

The magnetisation *vs*. field and susceptibility measurements on **3-U** suggested magnetic blocking at low temperature (Figure S99). This was investigated further with hysteresis and dynamic susceptibility measurements. The sample of **3-U** was prepared for the zero-field cooled susceptibility by holding the sample at 100 K for 10 min in zero field, rapidly cooling to 10 K (30 K min^{-1}) and holding for 5 min and then cooling to 2 K at 5 K min^{-1} and holding for 45 min. The field was then ramped to 1 kOe at 700 Oe s^{-1} and held constant, at which point the temperature was swept continually to 100 K at a warming rate of +0.5 K min^{-1} and the moment measured every 1 K. After waiting for 10 min at 100 K, the field-cooled trace was obtained by measuring the susceptibility every 1 K while continually cooling at –0.5 K min^{-1}. The zero-field cooled (ZFC) and field-cooled (FC) susceptibilities diverge at 56 K with the ZFC susceptibility peaking at 23 K (Figure S100). This is an unusually high temperature for divergence in U(III) complexes.[29]

Hysteresis measurements were made by magnetising the sample at 70 kOe and sweeping the field through zero to –70 kOe, waiting 10 mins, then sweeping back through zero to 70 kOe at constant temperature. Measurements were made in continuous field sweep mode, with slight delays between sections of different rates. For $2 < |H| \leq 7$ T, measurements were recorded in approximately 1250 Oe steps with an average sweep rate of 87 Oe s^{-1}. For $1 < |H| \leq 2$ T, measurements were recorded approximately every 1000 Oe with an average sweep rate of 48 Oe s^{-1}. For $-1 \leq H \leq 1$ T, measurements were recorded approximately every 250 Oe with an average sweep rate of 21.5 Oe s^{-1}. The measurement sequence was repeated at 2 K and 4 K. The hysteresis measurements were closed around the important zero-field region at 2 and 4 K (Figure S101).

Ac susceptibility measurements were performed on the Quantum Design MPM3 SQUID magnetometer with an oscillating field of 2 Oe and either a zero dc field or a 1 kOe

static dc field. Measurements were recorded every 0.5 K between 2 and 5 K and for 25 frequencies between 1 and 1000 Hz (0.1–1000 Hz for 2 K in 1 kOe dc field). Ac susceptibilities were found to adjust very slowly to changes in temperature, so before recording variable-frequency ac data, the 1000 Hz ac susceptibility was measured continually until a constant value was obtained (15–40 min for a change of 0.5 K). The frequency dependence of the ac susceptibility (1–1000 Hz) in zero dc field (H_{dc} = 0), revealed peaks in the out-of-phase susceptibility between 2–4.5 K. In an applied field of 1 kOe, the peaks shifted but could still only be observed between 2–5 K. Ac data was analysed in CC-FIT2,[30] fitting the χ' vs χ'' Cole-Cole plot to the generalised Debye model, to obtain relaxation times (τ) and distribution of relaxation times (α) (Figures S102–S105, Tables S6–S7).

The extracted relaxation times for **3-U** were fit in CC-FIT2, which uses the α values to assign errors to the relaxation rates that correspond to one estimated standard deviation in the distribution of the rates.[30] The relaxation profile in zero dc field was fit to a combined quantum tunnelling of magnetisation (QTM) and Raman rate equation (Equation S1, Figure S106).

$$\tau^{-1} = \tau_{QTM}^{-1} + \tau_{Raman}^{-1} = \tau_{QTM}^{-1} + CT^{n} \qquad \text{Equation S1}$$

The relaxation profile in 1 kOe dc field was fit to a Raman rate equation (Equation S2, Figure S106).

$$\tau^{-1} = \tau_{Raman}^{-1} = CT^{n} \qquad \text{Equation S2}$$

The following parameters were obtained: $C = 0.79^{+40.28}_{-0.78}$ s^{-1} K^{-n}, n = 5.9 ± 2.7 and $\tau_{\mathrm{QTM}} = (2.7^{+5.8}_{-1.8}) \times 10^{-3}$s for H_{dc} = 0 and $C = 0.92^{+1.69}_{-0.59}$ s^{-1} K^{-n} and n = 5.2 ± 0.8 for H_{dc} = 1 kOe; the large errors reflect both the distribution of relaxation rates and errors associated with fitting over a narrow temperature range.

We conclude that as **3-U** does not exhibit a Orbach process in the observable temperature and frequency range, it may not be considered to be a single-molecule magnet by some definitions in the literature.[31] Typically, U(III) SMMs display small U_{eff} values of 3.8–23

cm^{-1}.[32–41] The butterfly-shaped hysteresis observed at 2 and 4 K (Figure S101) is a result of slow relaxation via a Raman process, in addition to QTM in near-zero field. As the slow relaxation is only observed below 5 K, the divergence of ZFC/FC susceptibilities at 56 K (Figure S100) and sharp drop in χT below 50 K (Figure S102) must be the result of slow sample thermalisation. The magnetic moment is in equilibrium at the sample temperature (when above 5 K) but this temperature lags behind the bath temperature.

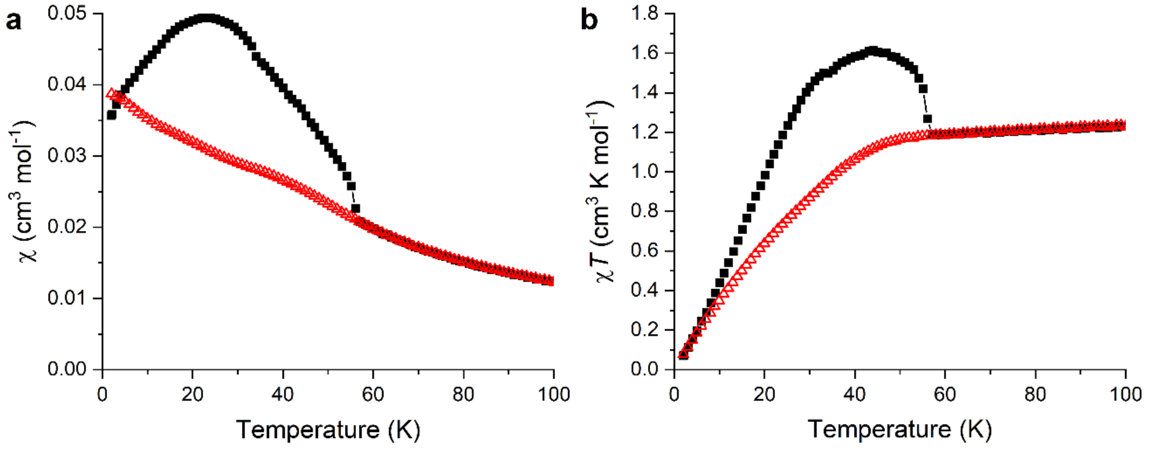

Figure S100. Temperature dependence of (a) magnetic susceptibility (χ) and (b) product of magnetic susceptibility and temperature (χT) for **3-U** measured on warming after cooling in zero field (solid black circles) and measured on cooling in field (open red triangles).

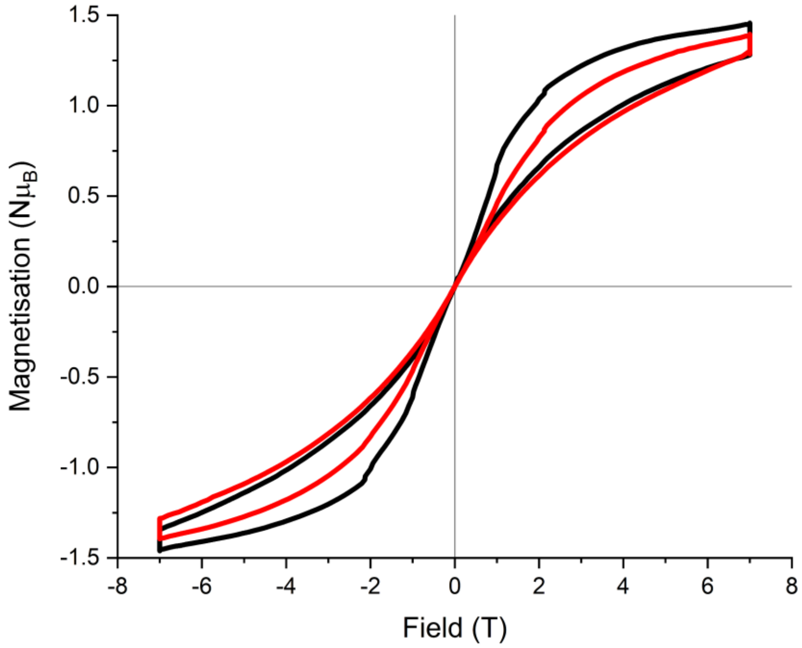

Figure S101. Hysteresis loops of **3-U** at 2 and 4 K. Sweep rate is 21.5 Oe s^{-1} for $|H| \leq 1$ T, 48 Oe s^{-1} for $1 < |H| \leq 2$ T and 87 Oe s^{-1} for $2 < |H| \leq 7$ T.

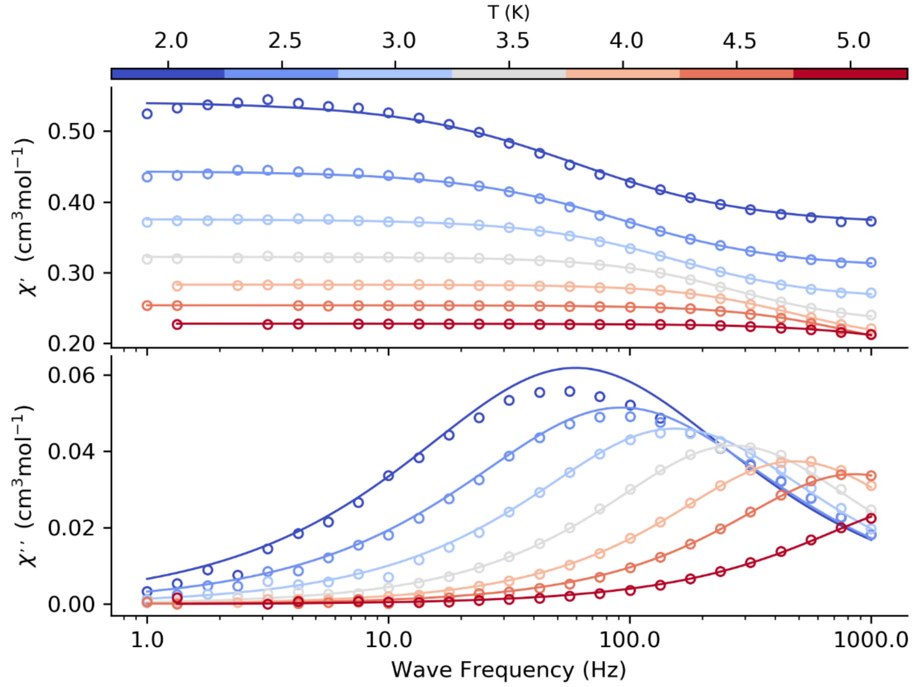

Figure S102. Fitting of ac data for **3-U** in zero field to generalised Debye model in CC-FIT2.[30]

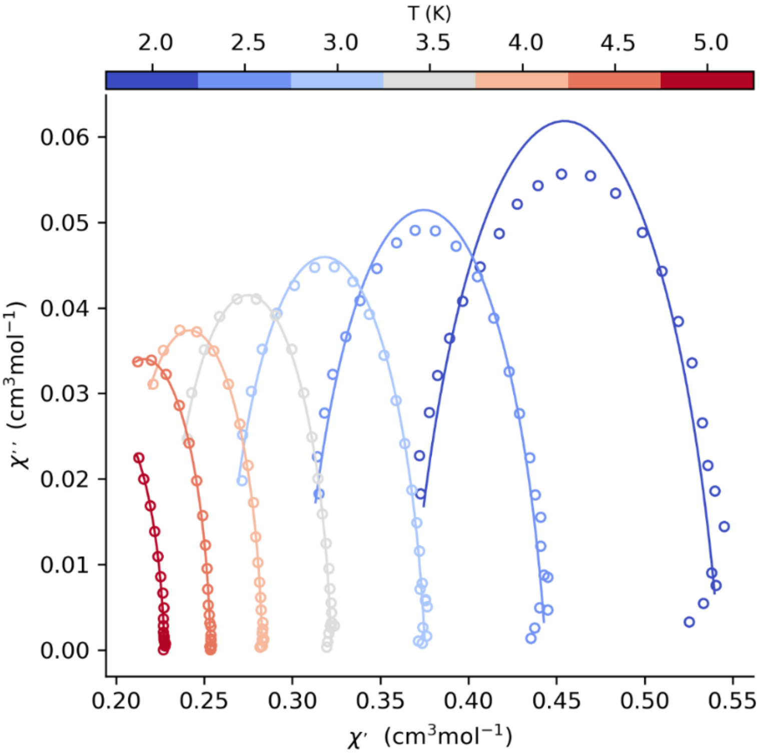

Figure S103. Cole-Cole plot showing fitting of ac data for **3-U** in zero dc field to generalised Debye model in CC-FIT2.[30]

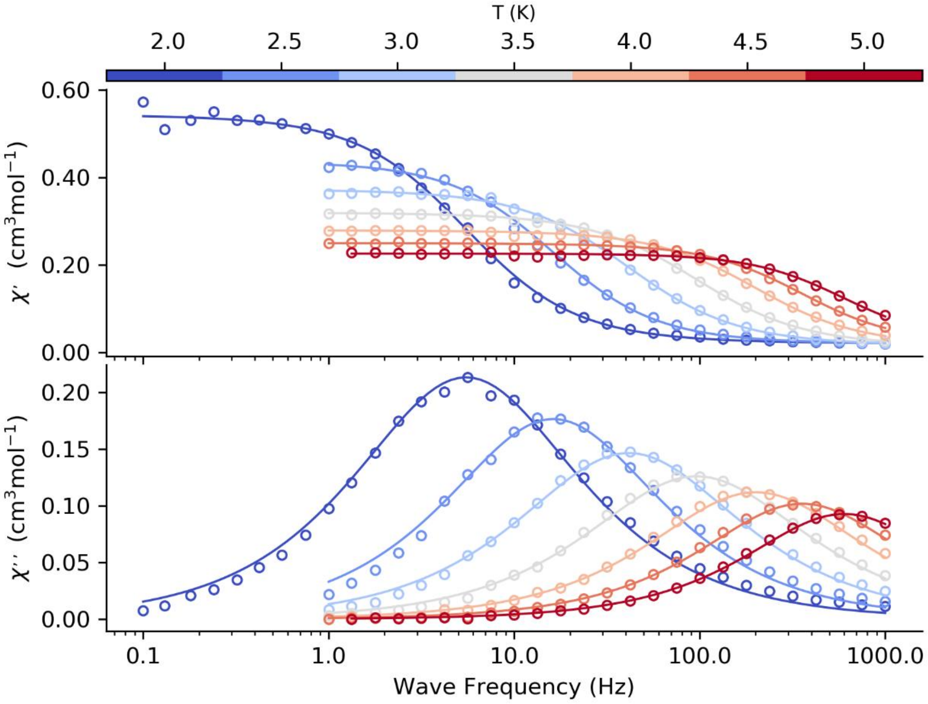

Figure S104. Fitting of ac data for **3-U** in 1 kOe field to generalised Debye model in CC-FIT2.[30]

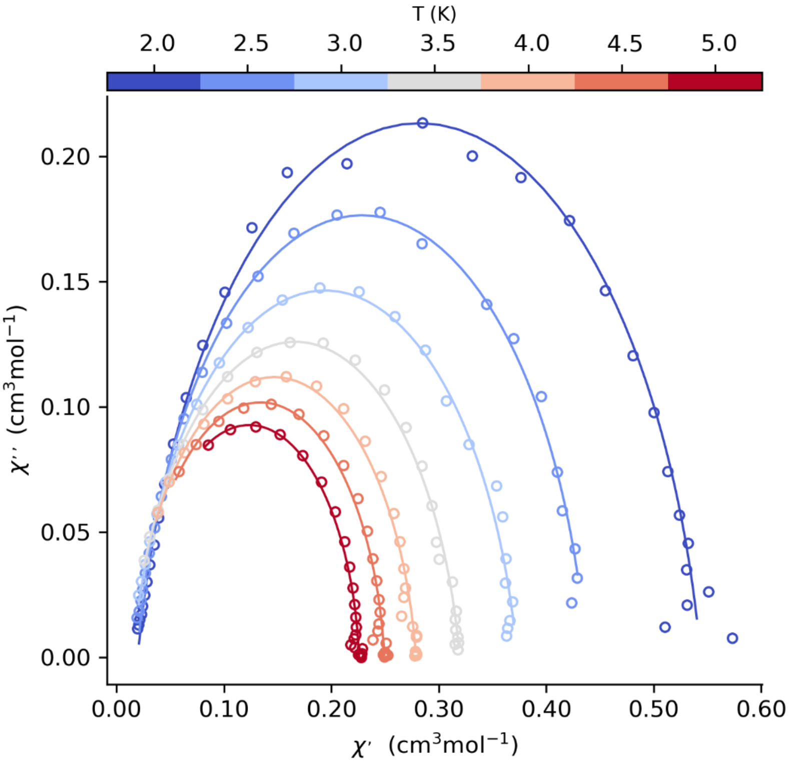

Figure S105. Cole-Cole plot showing fitting of ac data for **3-U** in 1 kOe dc field to generalised Debye model in CC-FIT2.[30]

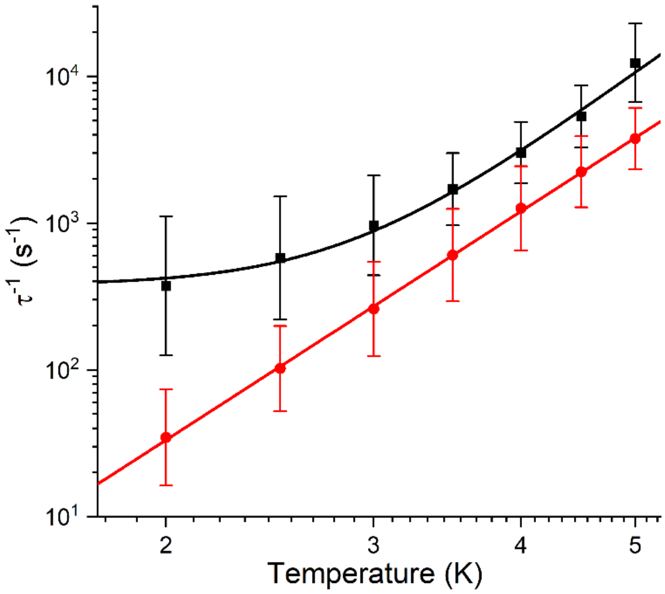

Figure S106. Relaxation profile for **3-U** in zero dc field (black squares) and 1 kOe applied dc field (red circles) with fits described above.

Table S6. Best fit parameters to the generalised Debye model for **3-U** in zero dc field.

T / K	τ / s	$\tau_{LN}^{err\_upper}$/ s	$\tau_{LN}^{err\_lower}$/ s	τ_{Debye}^{err}/ s	χ_S	χ_S^{err}	χ_T	χ_T^{err}	α	α^{err}
2.0	2.67E-03	7.96E-03	8.98E-04	1.07E-04	3.67E-01	2.65E-03	5.42E-01	1.88E-03	2.19E-01	1.87E-02
2.5	1.73E-03	4.53E-03	6.58E-04	4.33E-05	3.05E-01	1.49E-03	4.44E-01	8.36E-04	1.86E-01	1.21E-02
3.0	1.04E-03	2.27E-03	4.74E-04	1.60E-05	2.61E-01	8.91E-04	3.75E-01	3.82E-04	1.38E-01	7.83E-03
3.5	5.87E-04	1.04E-03	3.33E-04	8.07E-06	2.28E-01	7.95E-04	3.22E-01	2.54E-04	8.21E-02	7.24E-03
4.0	3.31E-04	5.35E-04	2.04E-04	4.60E-06	2.01E-01	7.89E-04	2.83E-01	1.63E-04	6.16E-02	6.49E-03
4.5	1.87E-04	3.04E-04	1.15E-04	3.70E-06	1.79E-01	1.05E-03	2.54E-01	1.17E-04	6.31E-02	6.80E-03
5.0	8.08E-05	1.50E-04	4.36E-05	7.79E-06	1.65E-01	4.05E-03	2.28E-01	1.40E-04	9.43E-02	1.72E-02

Table S7. Best fit parameters to the generalised Debye model for **3-U** in 1 kOe dc field

T / K	τ / s	$\tau_{LN}^{err\_upper}$/ s	$\tau_{LN}^{err\_lower}$/ s	τ_{Debye}^{err}/ s	χ_S	χ_S^{err}	χ_T	χ_T^{err}	α	α^{err}
2.0	2.89E-02	6.15E-02	1.36E-02	5.04E-04	1.99E-02	2.41E-03	5.44E-01	2.96E-03	1.30E-01	8.96E-03
2.5	9.79E-03	1.91E-02	5.02E-03	1.74E-04	1.94E-02	2.26E-03	4.38E-01	3.12E-03	1.08E-01	9.98E-03
3.0	3.84E-03	8.05E-03	1.83E-03	5.12E-05	1.53E-02	1.79E-03	3.73E-01	1.57E-03	1.26E-01	7.31E-03
3.5	1.66E-03	3.42E-03	8.01E-04	1.72E-05	1.37E-02	1.47E-03	3.19E-01	8.36E-04	1.22E-01	5.63E-03
4.0	7.92E-04	1.53E-03	4.10E-04	1.13E-05	1.46E-02	2.09E-03	2.79E-01	7.76E-04	1.05E-01	7.44E-03
4.5	4.47E-04	7.80E-04	2.56E-04	7.67E-06	1.87E-02	2.54E-03	2.49E-01	6.30E-04	7.95E-02	8.42E-03
5.0	2.65E-04	4.29E-04	1.64E-04	6.10E-06	2.14E-02	3.28E-03	2.26E-01	5.19E-04	6.17E-02	9.61E-03

12. EPR Spectroscopy

Continuous-wave (CW) electron paramagnetic resonance (EPR) spectra were recorded at X-band (*ca.* 9.4 GHz), K-band (*ca.* 24 GHz) and Q-band (*ca.* 34 GHz) frequencies on a Bruker EMXPlus spectrometer with 1.8 T electromagnet and Stinger closed-cycle helium gas cryostat. Some room temperature and 130 K X-band spectra were recorded on a Bruker EMX Micro X-band spectrometer with 1.0 T electromagnet and liquid nitrogen cryostat. Powders of **2-Zr** and **3-M** were finely ground in a glovebox, and 1–2 cm of sample was loaded into to 4 mm (X-band) and 1.9 mm diameter (Q-band) quartz tubes and sealed under vacuum. For **1-Ti** the low melting point of the sample limited the amount of grinding that could be achieved, and spectra were recorded on a powder sample similarly prepared in a 4 mm diameter quartz tube at X-band and K-band. Spectra were obtained for two rotations at ~90 degrees to one another to identify any features due to polycrystallinity.

Solutions of **3-Ce**, **3-Nd** and **3-U** in 9:1 toluene:hexane (15 mM), were prepared in a glovebox. Quartz tubes of diameter 4 mm (X-band) were filled with ~3 cm of solution, which was immediately frozen and flame-sealed under vacuum. Solution samples were stored in liquid nitrogen until measurement.

Solutions of **1-Ti**, **3-Ti**, **2-Zr** (referred to here as **1-Zr**, as observed in solution) and **3-Zr** in 9:1 toluene:hexane (5 mM), were prepared in a glovebox, transferred to 4 mm (X-band) and 1.9 mm diameter (Q-band) quartz tubes, frozen in liquid nitrogen and sealed under vacuum. Measurement of **3-Ti**, **1-Zr** and **3-Zr** solutions was challenging as they gradually decompose in solution and EPR is very sensitive to minor impurities. To counteract this, solvent and quartz tubes were pre-cooled at –20°C and solutions were immediately frozen after dissolution and removal from the glovebox. After sealing, samples were rapidly transferred to the spectrometer within *ca.* 15 mins. Measurements on **3-Ti** and **1-Zr** were additionally performed at 50 K to slow decomposition, except for **3-Ti** at X-band for which the best spectrum was obtained for a

1 mM solution at 130 K. An X-band fluid solution spectra was obtained at 295 K for **1-Ti**. Reliable fluid solution spectra of **3-Ti**, **1-Zr** and **3-Zr** could not be obtained due to decomposition at room temperature.

All Ce, Nd and U spectra were obtained at base temperature (5–8 K). For Ti and Zr samples, frozen solution and powder spectra were typically obtained at 50 or 130 K, depending on the temperature stability of the individual sample, and the temperature at which saturation of the EPR signal was minimised. The field was corrected using a strong pitch sample (g = 2.0028).

Spectra were simulated in EasySpin 6.0.0-dev.26, dev.30 or dev.39.[42] Powder and frozen solution spectra were simulated in the EasySpin function pepper. The spectra were simulated as an S = ½ (nd^1) or an effective S = ½ (4f^1 and nf^3), with rhombic g values, $g = [g_1, g_2, g_3]$. In the case of **2-Zr**, g-values were not resolved, and an isotropic g-value and isotropic g strain was used. Where hyperfine coupling was resolved, an anisotropic hyperfine coupling, $A = [A_1, A_2, A_3]$, was included in the model, where A is assumed to be collinear with g. Hyperfine coupling constants are defined for the most abundant isotope and were scaled for other isotopes based on nuclear g-factors, $A_i(^{145}\text{Nd}) = 0.615A_i(^{143}\text{Nd})$.[42] Line broadening is the result of a distribution of g-values, unresolved hyperfine coupling/a distribution of A-values, and dipolar coupling, amongst other effects. Anisotropic line broadenings have been modelled with phenomenological g strains (distribution of g values) or H strains (unresolved orientation-dependent hyperfine coupling, used for **1-Ti** and **3-Ti**) to account for all broadening effects. For powder spectra of **2-Zr** and **3-Zr**, a peak-to-peak isotropic Lorentzian linewidth combined with g strains provided a better simulation. Simultaneous simulations were performed on multi-frequency spectra of the same sample using the same g and A values. In most cases, broadening parameters were allowed to vary between the two frequencies. Separate simulations were

performed on frozen solution and powder spectra, as initial simulations indicated a slight shift in g-values upon dissolution, consistent with minor changes in geometry.

Spectra of **3-Ce** (solution only), **3-Nd** and **3-U** indicated some g-values lay above the accessible field range at X-band ($g < 0.4$). Spectra were fit with an effective S = ½ model, with g-values and g strains for the observed g-features used as fitting parameters. Unobserved g-values were fixed to the CASSCF predicted values, or to 0.2 for **3-Ce**. Unobserved g strains were fixed at 0.02 for **3-Ce** and **3-Nd**, and $gStrain_2 = 0.01$, $gStrain_3 = 0.001$ for **3-U**. For the frozen solution spectrum of **3-U**, two g_1 features were observed, and the simulation was performed with two S = ½ components, identical except for the g_1, $gStrain_1$ and relative weighting. For **3-Nd**, hyperfine coupling to Nd was only observed on the g_1 feature. The A_1 hyperfine coupling was included as a modelling parameter and A_2 and A_3 were set to zero.

The fluid spectrum of **1-Ti** was simulated in the isotropic regime using the EasySpin function garlic. The spectrum was simulated as two S = ½ components, with variable relative weights, isotropic g-value (g_{iso}) and peak-to-peak isotropic Voightian linewidths, containing a Gaussian and Lorentzian component. The final parameters (Table S8) were obtained by least-squares fitting in the EasySpin program esfit. Hyperfine coupling to ^{47}Ti (I = 5/2, 7.4 %) and ^{49}Ti (I = 7/2, 5.4 %) were not resolved for either component and lie within the linewidth. The major component, $g_{iso} = 1.9620$, corresponds to the same species measured in frozen solution, with $g_{ave} = (g_1 + g_2 + g_3)/3 = 1.9624$.

An X-band EPR spectrum including dipole coupling was simulated for **3-U**, to confirm that dipole coupling is not responsible for additional features at 67 and 175 mT. Dipole interactions between two S_{eff} = ½ states were calculated using the following equation:[43]

$$\bar{\bar{D}}_{AB} = \frac{{\mu_B}^2}{r^3}\left(\bar{\bar{g}}_A \cdot \bar{\bar{g}}_B - 3(\bar{\bar{g}}_A \cdot \vec{R}) \cdot (\vec{R}^T \cdot \bar{\bar{g}}_B)\right) \qquad \text{Equation S3}$$

Where μ_B is the Bohr magneton, r is the distance between the two U centres in metres, $\bar{\bar{g}}_A$ and $\bar{\bar{g}}_B$ are the g-matrices of the two U centres A and B in orthogonal crystal coordinates (rotated

from the g-frame to crystal coordinates with Euler angles fit in PHI), $\vec{R}$ is the directional unit vector from A to B in orthogonal crystal coordinates. The g unit vectors were taken from the CAS(3,7)SCF calculation averaging over 35 quartets and 112 doublets, the g_1 value was taken from experiment (6.055) and g_2 and g_3 were taken from CASSCF. The EPR spectrum was simulated in PHI for pairs of **3-U** in the crystal, with $\overline{\overline{D}}_{AB}$ multiplied by –0.5 to be consistent with the –2J exchange formalism used in PHI.[44] The strongest exchange interaction was with the {1–x, 1–y, –z} symmetry-generated molecule, with 10.712 Å between U centres (Figure S121). The dipolar exchange cannot account for the additional features and would likely result in line broadening once combined with smaller exchange contributions from other symmetry-equivalent molecules.

Equations describing EPR in d¹ complexes

In a d[1] transition metal complex with d_{z^2} ground state the g anisotropy ($\Delta g_i = g_i - g_e$) and hyperfine coupling constants (A_i) are given as follows.[45] This approximation considers only the d-orbitals (weighted by molecular orbital coefficients) and assumes no mixing of the d-orbitals:

$$\Delta g_x = -\frac{6\delta^2{\gamma_2}^2\lambda}{E_{yz}}$$

$$\Delta g_y = -\frac{6\delta^2{\gamma_1}^2\lambda}{E_{xz}}$$

$$\Delta g_z = 0$$

Equations S4–S6

$$A_z = P\left[-\delta^2\kappa + \frac{4}{7}\delta^2 + \frac{3\delta^2{\gamma_1}^2\lambda}{7E_{xz}} + \frac{3\delta^2{\gamma_2}^2\lambda}{7E_{yz}}\right]$$

$$A_x = P\left[-\delta^2\kappa - \frac{2}{7}\delta^2 + \Delta g_x - \frac{3\delta^2{\gamma_1}^2\lambda}{7E_{xz}}\right]$$

$$A_y = P\left[-\delta^2\kappa - \frac{2}{7}\delta^2 + \Delta g_y - \frac{3\delta^2{\gamma_2}^2\lambda}{7E_{yz}}\right]$$

Equations S7–S9

Where δ is the molecular orbital coefficient of the d_{z^2} orbital, γ_1 and γ_2 are the molecular orbital coefficients of the d_{xz} and d_{yz} orbitals in their respective molecular orbitals with energies E_{xz} and E_{yz} relative to the d_{z^2} molecular orbital ground state, λ is the spin orbital coupling constant for the complex (which depends on quantum numbers *n* and *l*), *P* is a scaling factor linked to the radial extent of the ground state wavefunction and κ is a dimensionless constant expressing the s orbital contribution.

The first term in the equations for hyperfine coupling constants, $-\delta^2\kappa$, is isotropic and represents the Fermi contact. The second term is determined by the ground state electronic structure, the third term reflects *g*-anisotropy (missing in equation for A_z as in this model $\Delta g_z = 0$) and the last term(s) represent second-order perturbation contribution(s). These equations can be recast to show the shift of A_i from the isotropic value, $\Delta A_i = A_i - A_{iso}$. Where $A_{iso} = P(-\delta^2\kappa + \Delta g_{iso})$ and $\Delta g_{iso} = g_{iso} - g_e$. Isotropic values A_{iso} and g_{iso} are typically measured in fluid solution and are equal to the average values $A_{ave} = (A_x + A_y + A_z)/3$ and $g_{ave} = (g_x + g_y + g_z)/3$, respectively, such that $\Delta g_{iso} = \Delta g_{ave} = g_{ave} - g_e$. We were unable to measure A_{iso} as **1-Zr** and **3-Zr** are unstable in fluid solution and so we have assumed all hyperfine constants are positive in the calculation of A_{ave}.

$$\Delta A_z = P\left[+\frac{4}{7}\delta^2 + \frac{3\delta^2{\gamma_1}^2\lambda}{7E_{xz}} + \frac{3\delta^2{\gamma_2}^2\lambda}{7E_{yz}}\right]$$

$$\Delta A_x = P\left[-\frac{2}{7}\delta^2 + \Delta g_x - \frac{3\delta^2{\gamma_1}^2\lambda}{7E_{xz}}\right]$$

$$\Delta A_y = P\left[-\frac{2}{7}\delta^2 + \Delta g_y - \frac{3\delta^2{\gamma_2}^2\lambda}{7E_{yz}}\right]$$

Equations S10–S12

Assuming the second-order perturbations are small compared to the other contributions, the *A* anisotropy is primarily governed by the ground state electron density and the *g*-anisotropy. For **1-Zr** and **3-Zr**, Δg_2 is small (–0.0189 and –0.0265, respectively) while Δg_3 is large (–0.1405

and –0.2847, respectively). In **3-Zr** the contributions of the ground state electron density and the *g*-anisotropy to A_3 are of a comparable magnitude ($\delta \leq 1$) resulting in a smaller A_3 than A_2.

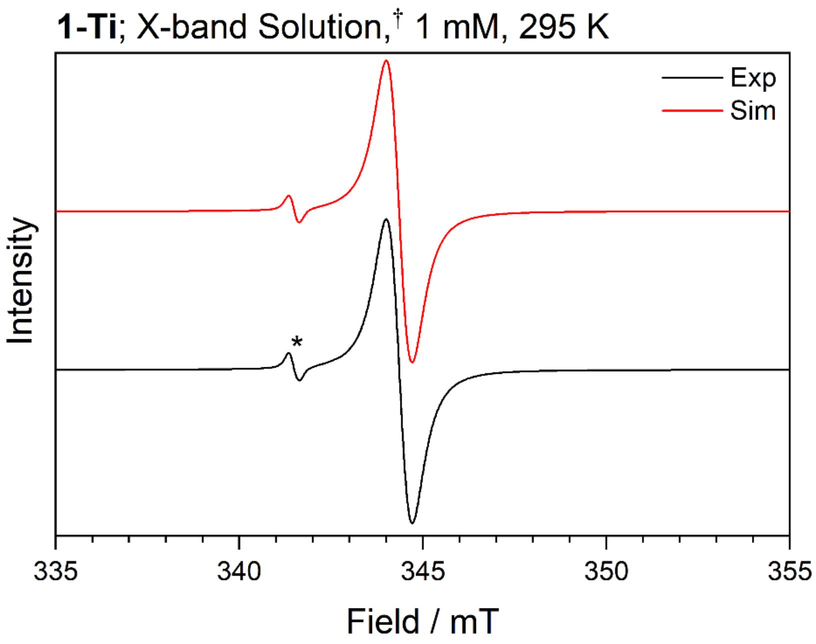

Figure S107. CW EPR spectrum of **1-Ti** measured at 295 K. Simulation shown in red † = solution measured in toluene:hexane (9:1). Asterisk (*) denotes the 1% impurity measured attributed to a THF solvated complex, **1-Ti.THF** which remained as part of the synthesis.[46]

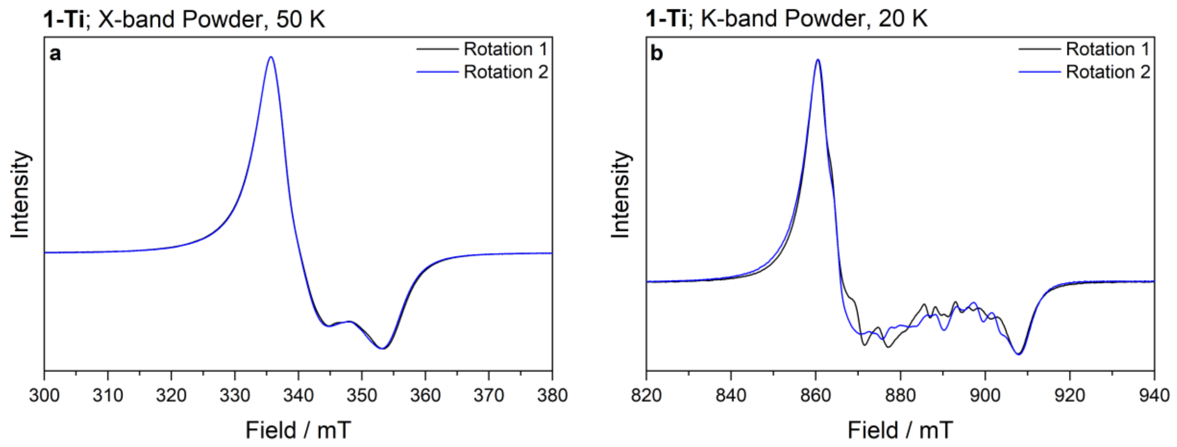

Figure S108. CW EPR spectra of **1-Ti** powder measured at (a) X-band, 50 K; (b) K-band, 20 K. Two rotations present indicate significant polycrystallinity at K-band.

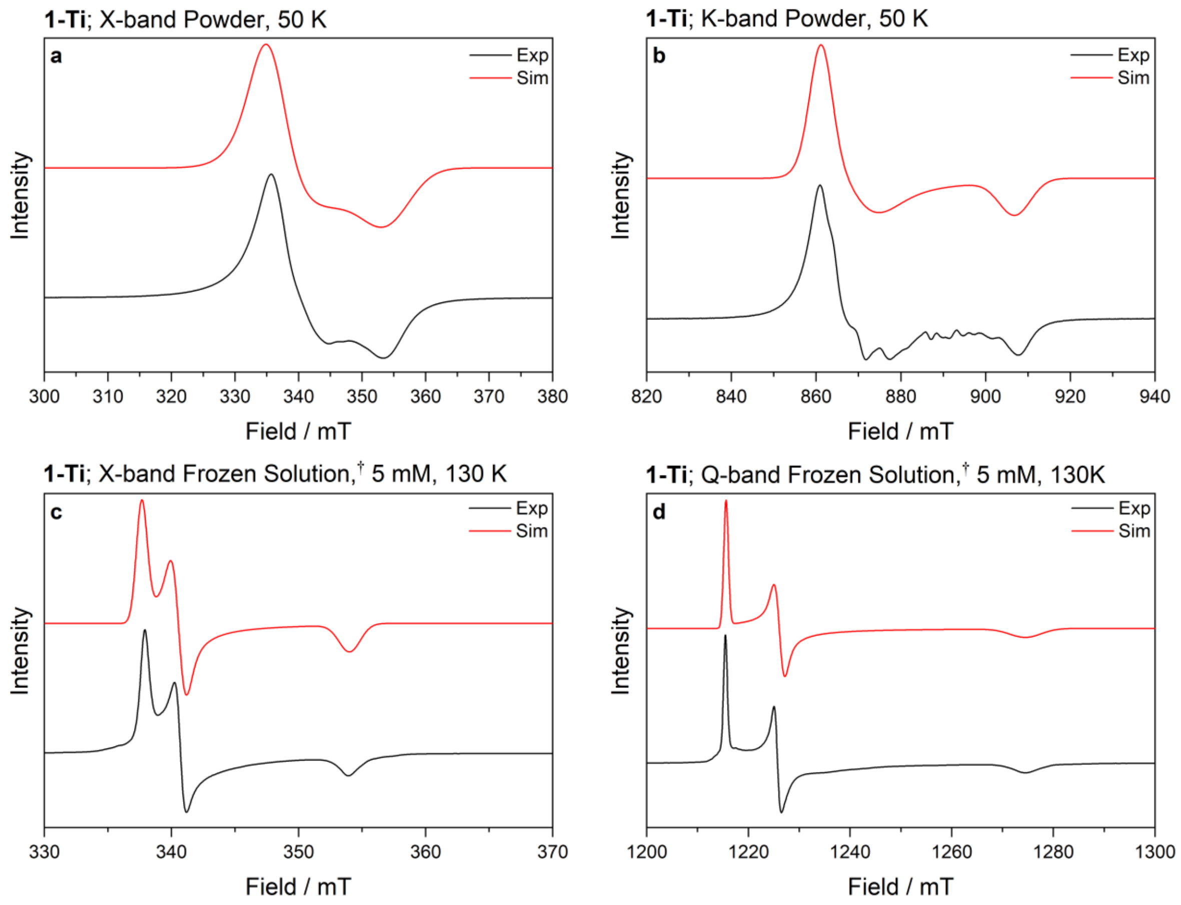

Figure S109. CW EPR spectrum of **1-Ti**. (a) X-band powder, 50 K; (b) K-band powder, 50 K; (c) X-band frozen solution, 130 K; (d) Q-band frozen solution, 130 K. Simulation shown in red. † = solution measured in toluene:hexane (9:1).

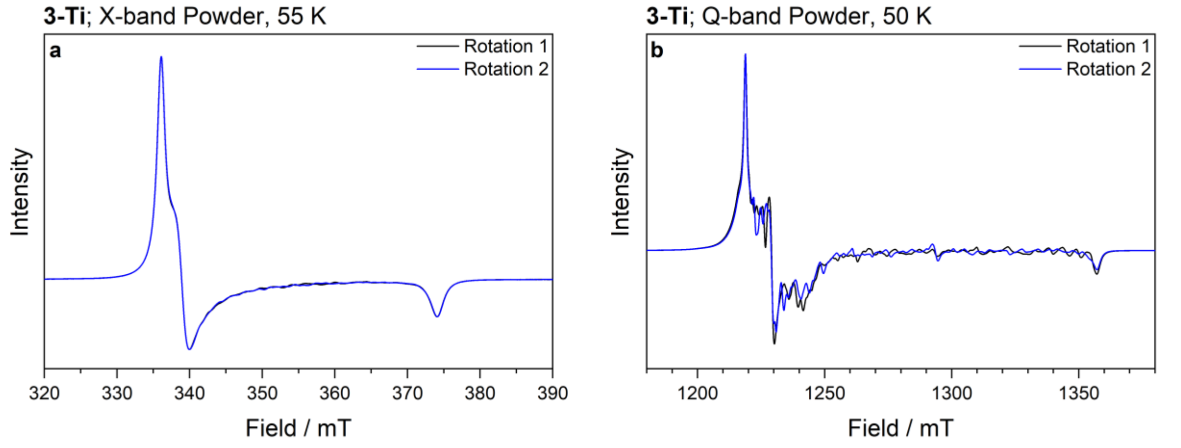

Figure S110. CW EPR spectra of **3-Ti** powder measured at (a) X-band, 55 K; (b) Q-band, 50 K. Two rotations present indicate significant polycrystallinity at Q-band.

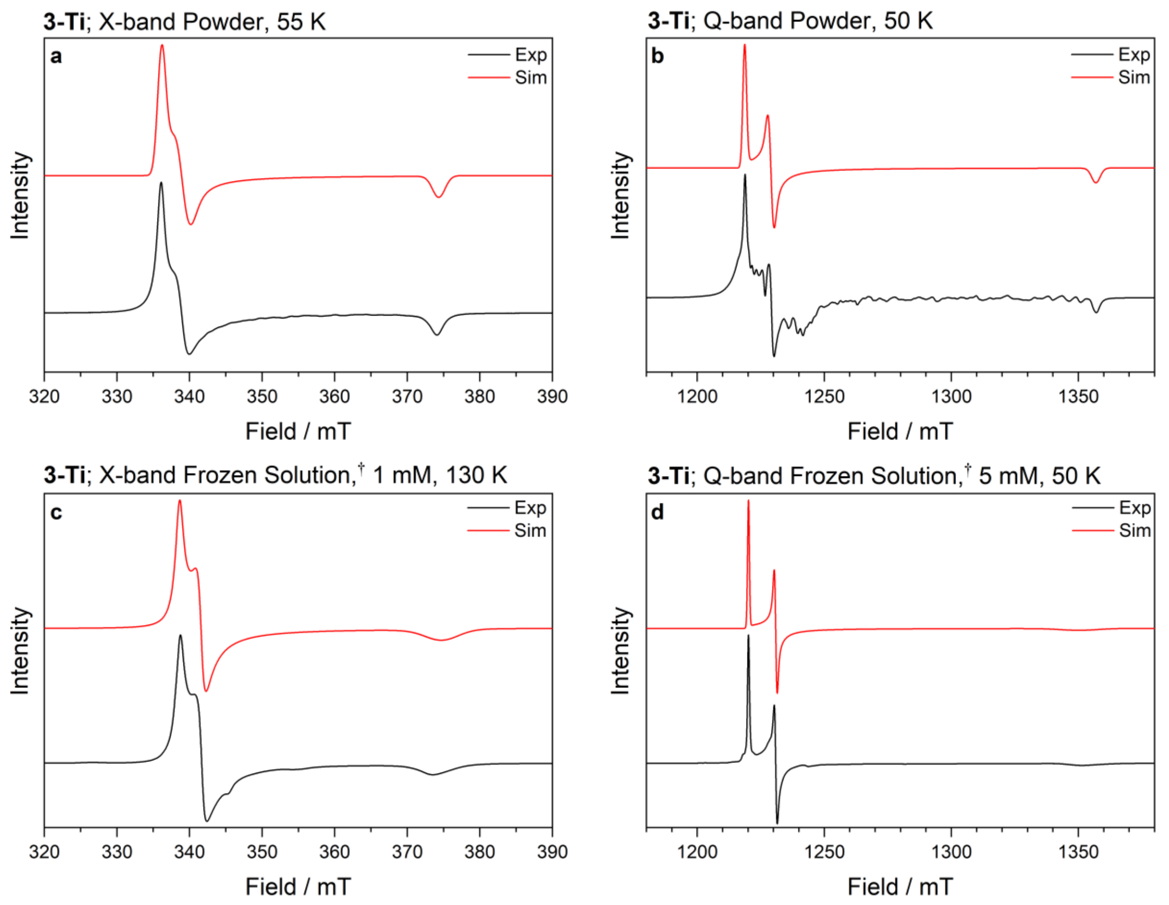

Figure S111. CW EPR spectra of **3-Ti**. (a) X-band powder, 55 K; (b) Q-band powder, 50 K; (c) X-band frozen solution, 130 K; (d) Q-band frozen solution, 50 K. Simulation shown in red. † = solution measured in toluene:hexane (9:1).

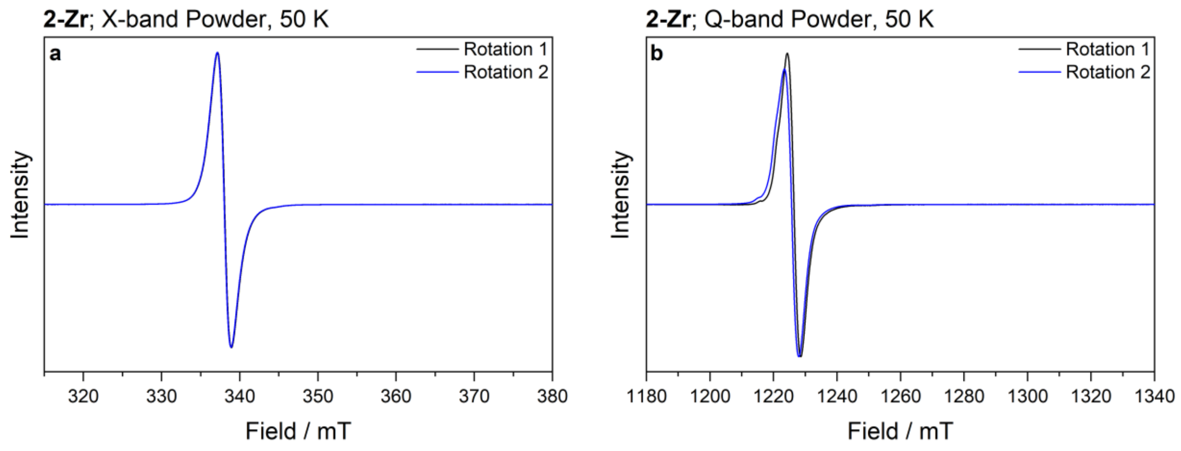

Figure S112. CW EPR spectra of **2-Zr** powder measured at (a) X-band, 50 K; (b) Q-band, 50 K. Two rotations indicate minimal polycrystallinity. Range is the same as Figure S115a and b.

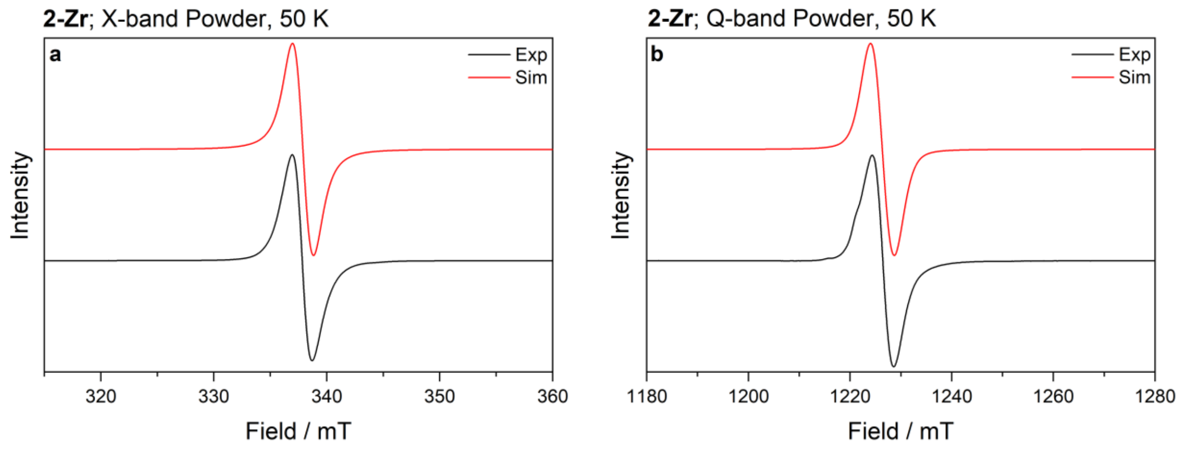

Figure S113. CW EPR spectra of **2-Zr**. (a) X-band powder, 50 K; (b) Q-band powder, 50 K. Simulation shown in red.

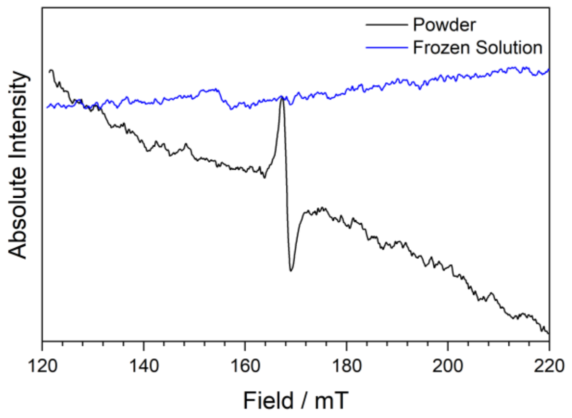

Figure S114. Half-field region in X-band CW EPR spectra of **2-Zr** as powder (black) and frozen solution (blue). Absolute intensities shown with no background corrections performed.

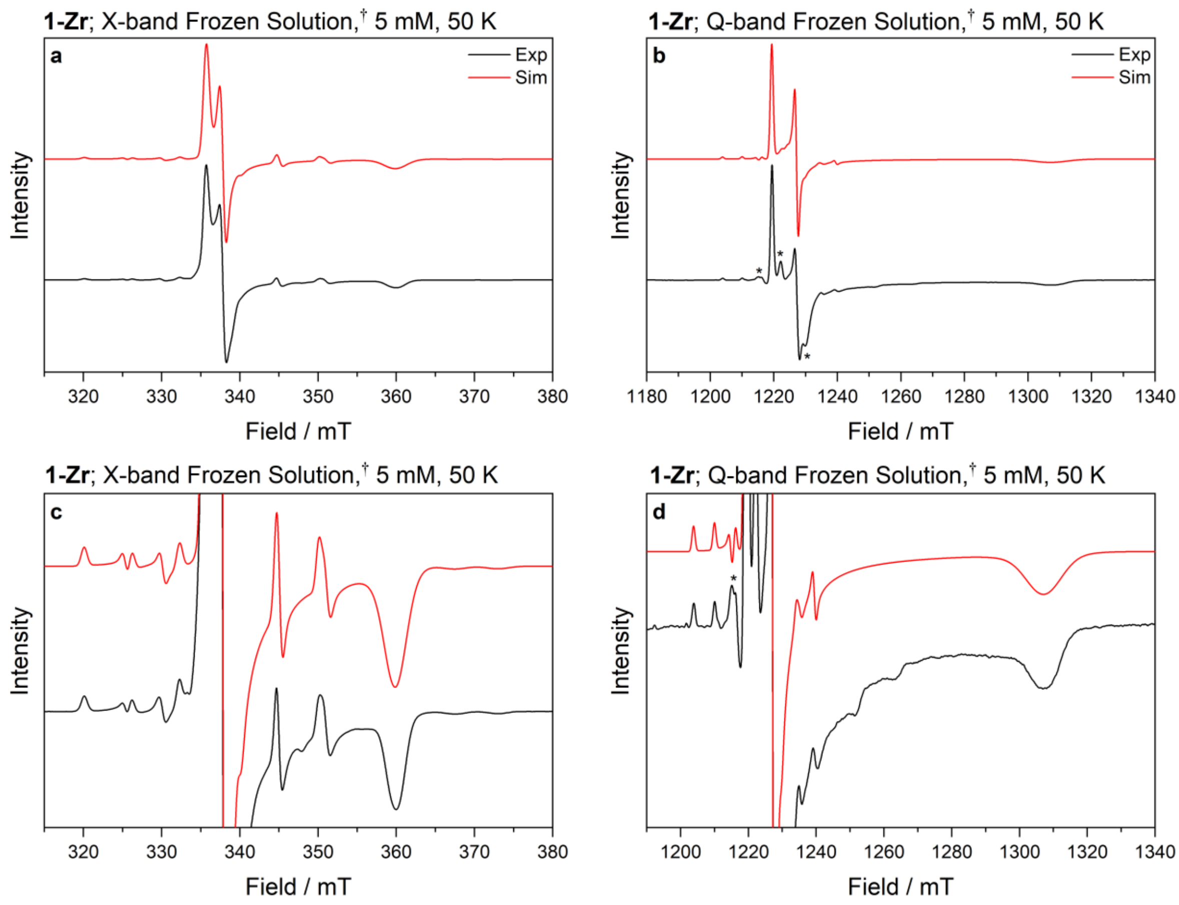

Figure S115. CW EPR spectra of frozen solutions of **1-Zr**. (a) X-band, 50 K, showing full spectrum and (c) magnified baseline; (b) Q-band, 50 K, showing full spectrum and (d) magnified baseline. Simulation shown in red. † = solution measured in toluene:hexane (9:1). Solutions were prepared in cold solvent, frozen at 77 K and measured immediately at 50 K. Despite these precautions, a small fraction of a second species is evident in the Q-band sample, indicated by asterisks (*).

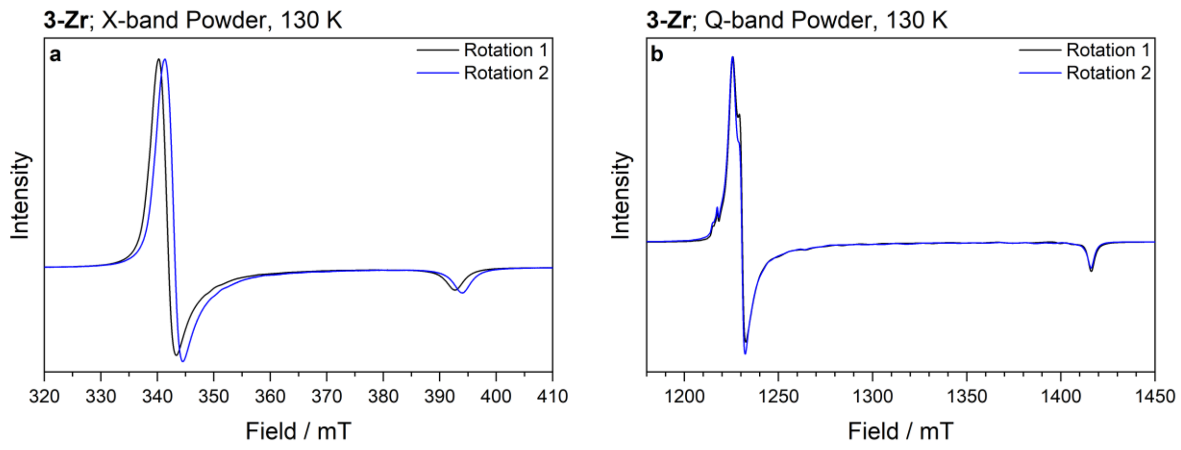

Figure S116. CW EPR spectra of **3-Zr** powder measured at (a) X-band, 130 K; (b) Q-band, 130 K. Offset at X-band is due to a frequency shift, minimal polycrystallinity is observed.

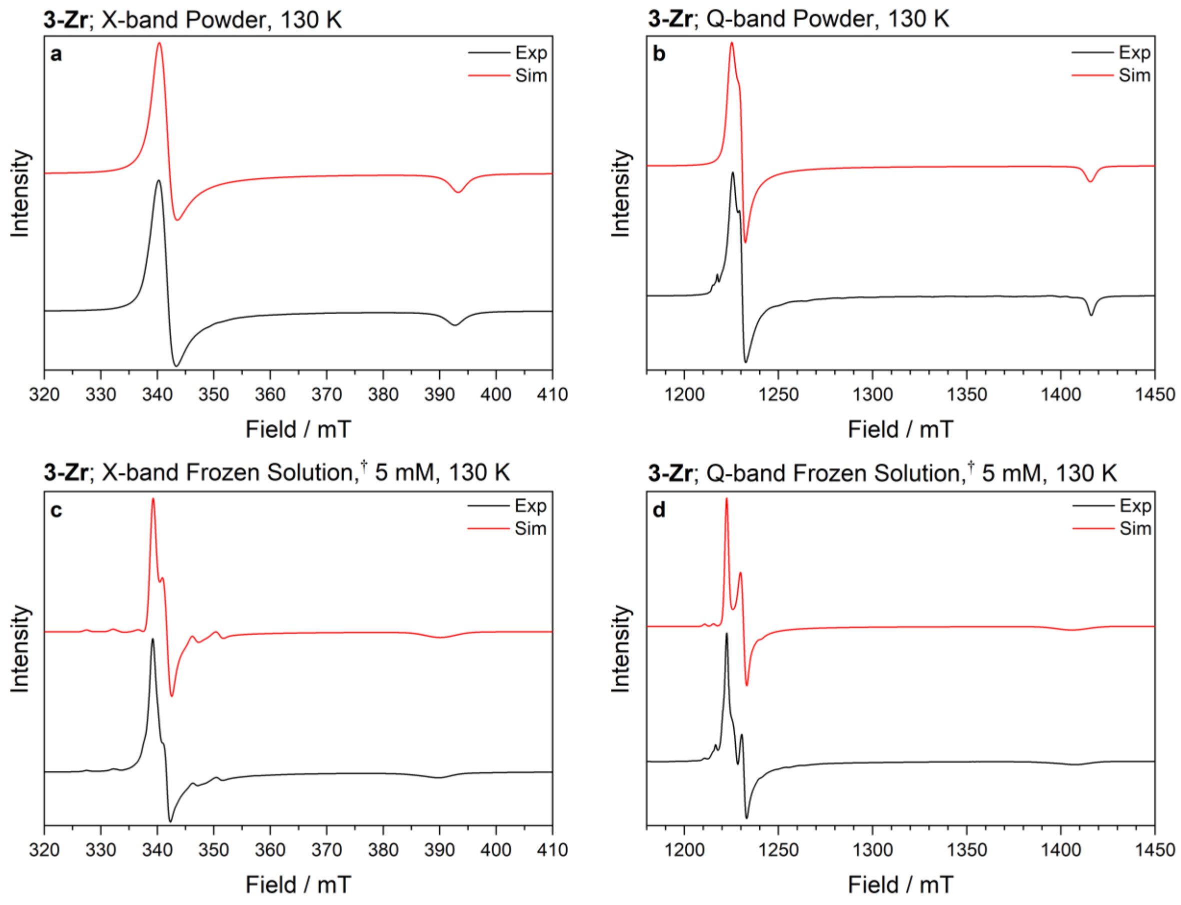

Figure S117. CW EPR spectra of **3-Zr**. (a) X-band powder, 130 K; (b) Q-band powder, 130 K; (c) X-band frozen solution, 130 K; (d) Q-band frozen solution, 130 K. Simulation shown in red. † = solution measured in toluene:hexane (9:1).

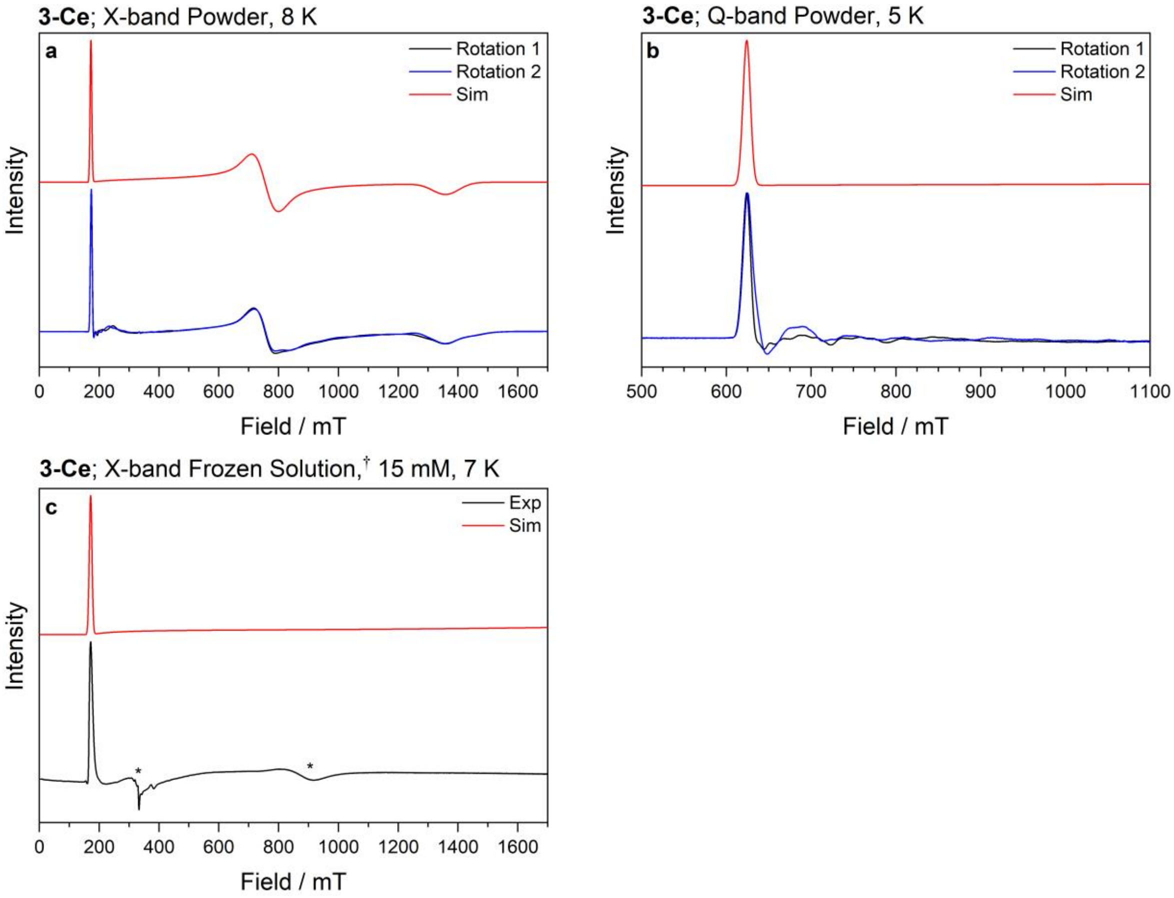

Figure S118. CW EPR spectra of **3-Ce**. (a) X-band powder, 8 K; (b) Q-band powder, 5 K; (c) X-band frozen solution, 7 K. Simulation shown in red. Two rotations show minimal polycrystallinity. † = solution measured in toluene:hexane (9:1). Asterisk (*) indicates feature intrinsic to cavity.

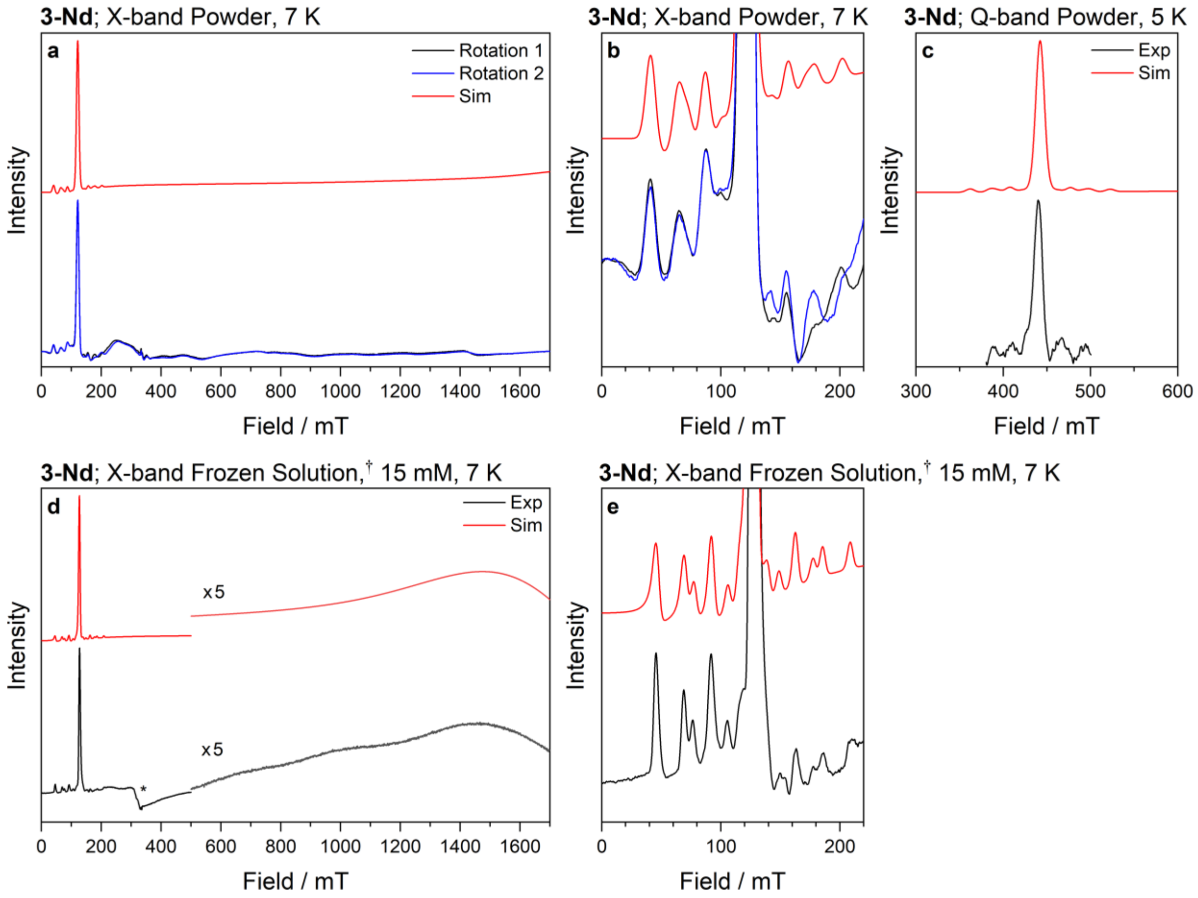

Figure S119. CW EPR spectra of **3-Nd**. (a) X-band powder, 7 K, showing full spectrum and (b) magnified baseline; (c) Q-band powder, 5 K; (d) X-band frozen solution, 7 K, showing full spectrum and (e) magnified baseline. Simulation shown in red. † = solution measured in toluene:hexane (9:1). Asterisk (*) indicates feature intrinsic to cavity.

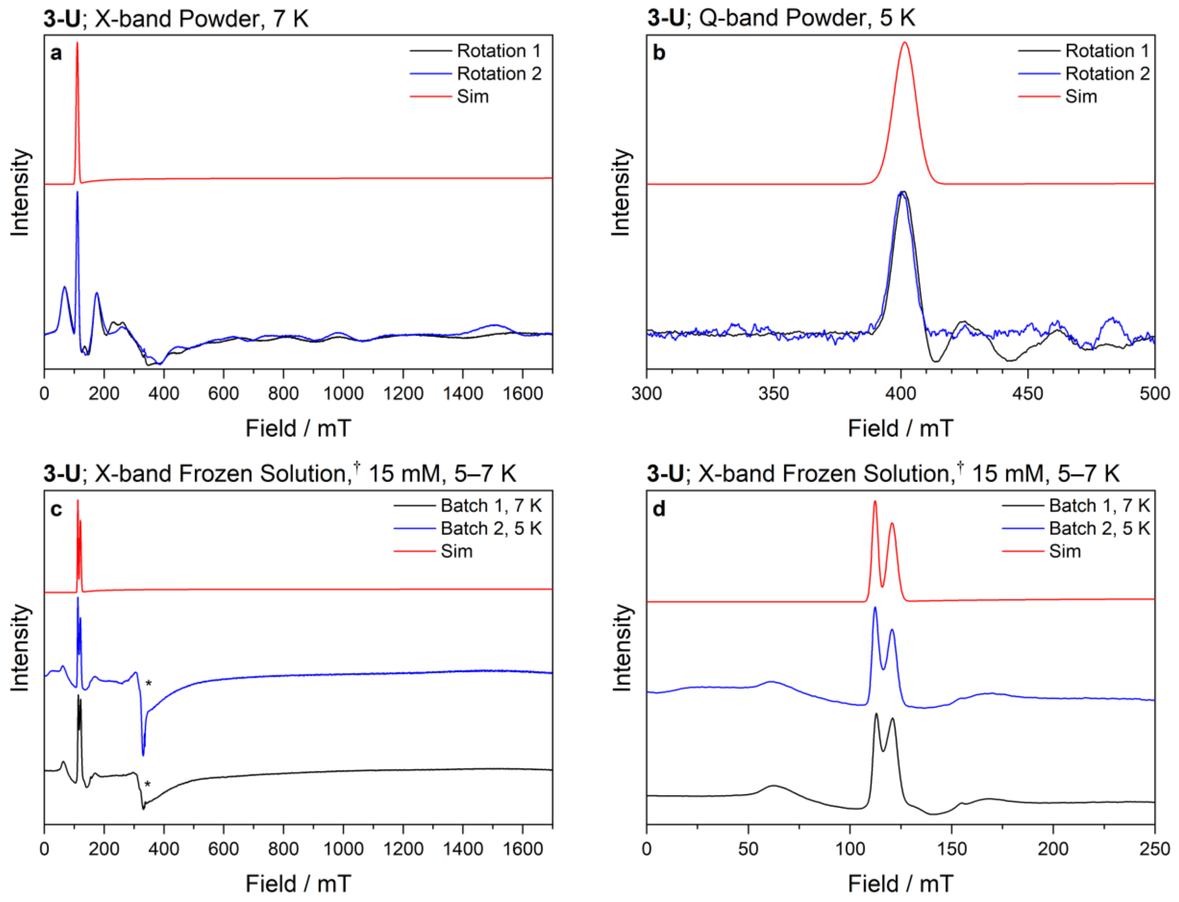

Figure S120. CW EPR spectra of **3-U**. (a) X-band powder, 7 K; (b) Q-band powder, 5 K; (c) X-band of two separate frozen solutions (5 K and 7 K) showing full spectra and (d) low field region. Simulation shown in red. Two rotations show minimal polycrystallinity. Asterisk (*) indicates feature intrinsic to cavity. † = solution measured in toluene:hexane (9:1).

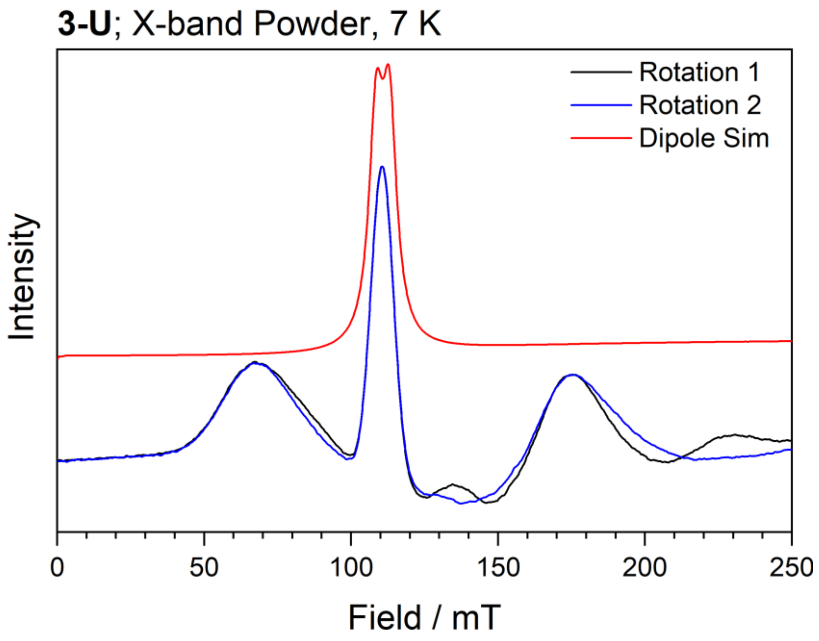

Figure S121. Low field CW EPR spectra of **3-U** powder at X-band with simulation (red) including dipolar coupling between symmetry equivalent molecules at {x, y, z} and {1–x, 1–y, –z}, as detailed in text.

Table S8. Fitting parameters for **1-Ti** fluid solution at X-band.

	g_{iso}	Gaussian Linewidth (mT)	Lorentzian Linewidth (mT)	Weight (%)
Major component	1.9620	0.348	0.532	98.93
Minor component	1.9785	0.203	0.142	1.07

Table S9. Simulation parameters for **1-Ti** and **3-Ti** powder and frozen solution EPR spectra.

Sample	Freq.	Temp. (K)	g_1	g_2	g_3	$HStrain_1$	$HStrain_2$ (MHz)	$HStrain_3$
**1-Ti** powder	X & K	50	1.9957	1.9800	1.8964	159	372	230
**1-Ti** frozen solution	X & Q	130	1.9990	1.9818	1.9065	27.9 (X) / 28.9 (Q)	31.6 (X) / 53.5 (Q)	52.0 (X) / 190 (Q)
**3-Ti** powder	X & Q	55 (X) / 50 (Q)	1.9946	1.9778	1.7914	37 (X) / 48 (Q)	60 (X) / 64 (Q)	51 (X) / 98 (Q)
**3-Ti** frozen solution	X & Q	130 (X) / 50 (Q)	1.9955	1.9781	1.8030	35 (X) / 21 (Q)	38 (X) / 28 (Q)	120 (X) / 425 (Q)

Table S10. Simulation parameters for **1-Zr** frozen solution, **2-Zr** powder and **3-Zr** powder and frozen solution and EPR spectra.

Sample	Freq.	Temp. (K)	g_1	g_2	g_3	$gStrain_1$	$gStrain_2$	$gStrain_3$	A_1	A_2 (MHz)	A_3	LW^a (mT)
**1-Zr** frozen solution	X & Q	50	1.9961	1.9834	1.8618	0.0053 (X) / 0.0017 (Q)	0.0045 (X) / 0.0016 (Q)	0.017 (X) / 0.017 (Q)	173.5	137	138	n/a
**2-Zr** powder	X & Q	50	1.9825^b	1.9825^b	1.9825^b	0.0076^b	0.0076^b	0.0076^b	n/a	n/a	n/a	1.20
**3-Zr** powder	X & Q	130	1.9853	1.9758	1.7176	0.0050	0.0013	0.0049	n/a	n/a	n/a	1.76
**3-Zr** frozen solution	X & Q	130	1.9874	1.9728	1.7280	0.0070 (X) / 0.0036 (Q)	0.0086 (X) / 0.0048 (Q)	0.027 (X) / 0.023 (Q)	131	91	80^c	n/a

a = Lorentzian linewidth. *b* = Isotropic *g*-values and *g* strains as *g*-values are not resolved. *c* = Upper limit of A_3, hyperfine not resolved within linewidth.

Table S11. Hyperfine constants (A_i) and g-values recast to show anisotropy. $g_{ave} = (g_1 + g_2 + g_3)/3$, $\Delta g_i = g_i - g_e$, $A_{ave} = (A_1 + A_2 + A_3)/3$ and assumes A_i are positive, $\Delta A = A_i - A_{ave}$.

Complex	State/ Temperature	g_{ave}	Δg_{ave}	Δg_1	Δg_2	Δg_3	A_{ave}	ΔA_1	ΔA_2	ΔA_3
**1-Ti**	FS/ 130 K	1.9624	–0.0399	–0.0033	–0.0205	–0.0958	-	-	-	-
	Powder/ 50 K	1.9574	–0.0449	–0.0066	–0.0223	–0.1059	-	-	-	-
	CASSCF	1.9340	–0.0683	–0.0029	–0.0368	–0.1653	-	-	-	-
**3-Ti**	FS/ 130, 50 K	1.9255	–0.0768	–0.0068	–0.0242	–0.1993	-	-	-	-
	Powder/ 55, 50 K	1.9213	–0.0810	–0.0077	–0.0245	–0.2109	-	-	-	-
	CASSCF	1.8906	–0.1117	–0.0073	–0.0428	–0.2850	-	-	-	-
**1-Zr**	FS/ 50 K	1.9471	–0.0552	–0.0062	–0.0189	–0.1405	149.5	24.0	–12.5	–11.5
	CASSCF	1.9237	–0.0786	–0.0050	–0.0499	–0.1809	-	-	-	-
**2-Zr**	Powder/ 50 K [a]	1.9825	–0.0198	–0.0198	–0.0198	–0.0198	-	-	-	-
	CASSCF [b]	1.9143	–0.0880	–0.0079	–0.0799	–0.1761	-	-	-	-
**3-Zr**	FS/ 130 K	1.8961	–0.1062	–0.0149	–0.0295	–0.2743	100.7	30.3	–9.7	–20.7
	Powder/ 130 K	1.8929	–0.1094	–0.0170	–0.0265	–0.2847	-	-	-	-
	CASSCF	1.8685	–0.1338	–0.0126	–0.0585	–0.3304	-	-	-	-

a = Isotropic model used to fit spectrum. *b* = CASSCF calculations on **2-Zr′**

Table S12. Simulation parameters for $4f^1$ and nf^3 EPR spectra.

Spectrum	Freq.	Temp. (K)	Weight (%)	g_1	g_2	g_3	A_1	$gStrain_1$	$gStrain_2$	$gStrain_3$
**3-Ce** powder	X & Q	8 (X) / 5 (Q)	-	3.884[a]	0.888[b]	0.493[b]	-	0.15 (X) / 0.06 (Q)	0.095[b]	0.037[b]
**3-Ce** frozen solution	X	7	-	3.907	0.200[c]	0.200[c]	-	0.24	0.02[d]	0.02[d]
**3-Nd** powder	X & Q	7 (X) / 5 (Q)	-	5.490[e]	0.296[f]	0.146[f]	1755	0.43 (X) / 0.14 (Q)	0.02[d]	0.02[d]
**3-Nd** frozen solution	X	7	-	5.225	0.360[b,g]	0.146[f]	1695	0.20	0.124[b]	0.02[d]
**3-U** powder	X & Q	7 (X) / 5 (Q)	-	6.055	0.0763[f]	0.0072[f]	-	0.47 (X) / 0.15 (Q)	0.01[d]	0.001[d]
**3-U** frozen solution[h]	X	5	44.2	5.949	0.0763[f]	0.0072[f]	-	0.175	0.01[d]	0.001[d]
			55.8	5.536	0.0763[f]	0.0072[f]	-	0.25	0.01[d]	0.001[d]

a = Q-band spectrum favoured when simulating g_1, simulating only the X-band spectrum gives g_1 of 3.857, suggesting a small error in the field-correction. *b* = *g*-value not observed at Q-band, fitting based on X-band only. *c* = *g*-value not observed experimentally, fixed to arbitrary value < 0.4. *d* = *g*-value not observed experimentally, *g* strain fixed. *e* = X-band spectrum was favoured when simulating g_1 as Q-band spectra is very weak, simulating only the Q-band spectrum gives g_1 of ~5.515. *f* = *g*-value not observed experimentally, value fixed to CASSCF value. *g* = Large uncertainty in *g*-value as only part of the feature is observed in the accessible field range. *h* = two similar species observed in solution, fit as the sum of two $S = ½$ components with identical g_2, g_3, $gStrain_2$ and $gStrain_3$ and different g_1, $gStrain_1$ and relative weighting.

13. Density Functional Theory (DFT) Calculations

DFT calculations were performed on the theorised complex, [Zr(Cp″)$_2$Cl] (**1-Zr**), in Gaussian 09 (rev. D).[47] Three initial geometries were used: (a) **1-Ti** with Ti substituted for Zr, (b) **2-Zr** taking one Zr centre only and replacing the two chloride ions with a single chloride placed along the bisector of the original Cl–Zr–Cl angle with a 2.985 Å Zr–Cl bond distance (same as in **2-Zr**), (c) **3-Zr** with {Si(SiMe$_3$)}$^{-}$ replaced with chloride and a 2.985 Å Zr–Cl bond distance. The geometry was optimised and frequencies calculated with the PBE functional,[48,49] Grimme's dispersion correction with Becke-Johnson damping (D3-BJ),[50,51] cc-pVDZ basis sets on all atoms except Zr.[52,53] For Zr a Stuttgart RSC 1997 effective core potential (ECP) was used for the 28 core electrons with corresponding valence basis set, obtained from the Basis Set Exchange.[54–58] Geometries (b) and (c) converged to the same structure, which had an imaginary frequency, distorting along this mode and reoptimising did not remove the imaginary frequency. The initial optimised geometry of (a) was lower in energy and had no imaginary frequencies, it was further optimised with the same method except with cc-pVTZ basis sets on all atoms.[52,53] The second optimised structure (Table S13), also without imaginary frequencies, was used in CASSCF-SO calculations. The final electronic energy of this structure was –2527.64915801 Hartree.

Table S13. Optimised coordinates of **1-Zr**.

Zr	0.00000	0.00000	0.00000
Cl	2.45341	0.00000	0.00000
Si	-1.12173	2.10183	-3.09983
Si	1.02593	2.96072	2.31546
Si	-1.12225	-2.10182	3.09987
Si	1.02624	-2.96090	-2.31503
C	-0.93157	1.93225	-1.23709
C	0.16986	2.43629	-0.46290
C	-0.10703	2.35082	0.93126
C	-1.41760	1.78531	1.03773
C	-1.92155	1.55074	-0.26880
C	-2.10267	3.68635	-3.43448
C	0.59288	2.24478	-3.87454
C	-2.07646	0.64213	-3.83084
C	2.57313	3.68288	1.51645

C	0.07597	4.28708	3.27367
C	1.50765	1.56296	3.49013
C	-0.93176	-1.93217	1.23717
C	0.16978	-2.43628	0.46319
C	-0.10689	-2.35091	-0.93102
C	-1.41745	-1.78541	-1.03774
C	-1.92158	-1.55069	0.26869
C	-2.07689	-0.64207	3.83091
C	0.59220	-2.24517	3.87485
C	-2.10353	-3.68621	3.43421
C	2.57351	-3.68268	-1.51582
C	1.50777	-1.56327	-3.48994
C	0.07653	-4.28760	-3.27303
H	1.10679	2.79325	-0.88285
H	-1.94989	1.58765	1.96401
H	-2.90161	1.14289	-0.50167
H	-1.59097	4.55898	-3.00494
H	-3.10446	3.63073	-2.98532
H	-2.22321	3.85830	-4.51401
H	0.51890	2.31810	-4.96901
H	1.21689	1.37428	-3.63341
H	1.11145	3.14513	-3.51560
H	-1.49349	-0.28620	-3.77472
H	-2.31174	0.83167	-4.88814
H	-3.02729	0.48166	-3.30237
H	2.32726	4.51274	0.83916
H	3.10070	2.90916	0.94104
H	3.26131	4.06608	2.28327
H	0.68938	4.69855	4.08825
H	-0.83973	3.87152	3.71822
H	-0.21687	5.11543	2.61373
H	2.06751	0.78773	2.94941
H	0.62783	1.09574	3.95276
H	2.14641	1.94872	4.29846
H	1.10665	-2.79321	0.88331
H	-1.94961	-1.58785	-1.96412
H	-2.90167	-1.14281	0.50138
H	-1.49375	0.28618	3.77507
H	-2.31242	-0.83175	4.88813
H	-3.02758	-0.48136	3.30227
H	0.51804	-2.31844	4.96931
H	1.21647	-1.37484	3.63379
H	1.11060	-3.14566	3.51602
H	-1.59191	-4.55889	3.00466
H	-3.10523	-3.63037	2.98488
H	-2.22427	-3.85824	4.51370
H	2.32773	-4.51246	-0.83840
H	3.10093	-2.90877	-0.94052
H	3.26178	-4.06591	-2.28254
H	2.06745	-0.78784	-2.94934
H	0.62788	-1.09629	-3.95272
H	2.14664	-1.94906	-4.29817
H	0.69001	-4.69907	-4.08756
H	-0.83926	-3.87230	-3.71763
H	-0.21614	-5.11591	-2.61297

14. Complete Active Space Self-Consistent Field (CASSCF) Calculations

CASSCF-SO calculations were performed with the program OpenMolcas[57,59] on XRD structures of **1-Ti**, **3-Ti**, **3-Zr** (one molecule selected only), **3-Ce**, **3-Nd** and **3-U**. In cases of disorder, the major component was selected. To investigate the EPR of **2-Zr** in frozen solution, we performed calculations on the XRD structure of **2-Zr** with one Zr substituted for Y (**2-Zr′**), and a DFT-optimised geometry of **1-Zr** (Table S13). The coordinates of monometallic complexes were aligned with the metal centre at the origin, the *z*-axis perpendicular to the plane defined by the two Cp″ centroids and the coordinating heteroatom, the *x*-axis along the metal-heteroatom bond and the *y*-axis tangential to Cp″–M–Cp″. The dinuclear complex, **2-Zr′**, was orientated in an equivalent orientation with the *x*-axis along the Zr–Y vector, the *z*-axis perpendicular to *x* in the $ZrYCl_2$ plane and the *y*-axis approximately along the Cp″–M–Cp″ axis. Basis sets from the ANO-RCC library[60,61] were used with VTZP quality on the metal atom, VDZP quality on the coordinating atoms and VDZ quality on all other atoms, employing the second-order DKH transformation. Cholesky decomposition of the two-electron integrals with a threshold of 10^{-8} was performed to save disk space and reduce computational demand.

The molecular orbitals (MOs) were averaged in state-averaged CASSCF calculations. For mononuclear d^1 complexes, the MOs were averaged over the lowest five doublets in a CAS(7,8) calculation, where the active space consisted of five d orbitals (3d for Ti; 4d for Zr) and three metal-ligand bonding orbitals containing significant (17–25%) d-character (Figures S122–S125). For the dinuclear $4d^1$ complex, **2-Zr′**, the MOs were averaged over the lowest five doublets in a CAS(15,12) calculation, where the active space consisted of five Zr 4d orbitals, four metal-ligand bonding orbitals containing significant (15–19%) d-character and three Zr 4p orbitals that push the metal-ligand bonding orbitals out of the active space if not included (Figure S126). Four M–L bonding orbitals are required rather than three for **1-Ti** and **3-M** because the d_{xz} orbital has stronger bonding and anti-bonding interactions with chloride

σ-orbitals. For *n*d[1] complexes, the states are dominated by a single configuration, with one singly occupied orbital that can be identified using the natural orbitals of that state. Approximate relative orbital energies can be determined from correlating the state energies to the singly-occupied orbitals (Figures S127–S133, Tables S14–S18). Orbital images created with Pegamoid v2.6.1.[62]

For **3-Ce** the MOs were averaged over the lowest seven doublets in a CAS(1,7) calculation, where the active space was the seven 4f orbitals. For **3-Nd** CAS(3,7) calculations were performed on each possible spin state, where the active space was the seven 4f orbitals: averaging over 35 quartets or 112 doublets in a calculation. A variety of active spaces and states were tested for **3-U**: (a) CAS(3,7) with seven 5f orbitals averaging over 13 quartets, arising from the ^{4}I ground term, (b) CAS(3,7) with seven 5f orbitals averaging over 35 quartets and 112 doublets, arising from all f^3 configurations, (c) CAS(3,9) with seven 5f orbitals and two 6d orbitals, averaging over 77 quartets and 210 doublets, arising from all $5f^3$ and all $5f^2 6d^1$ configurations. Method (a) provided the best reproduction of M_{sat}. Method (b) gave the best reproduction of magnetic susceptibility and g_1, but with small (<5%) contributions of 6d orbitals to the state-averaged orbitals. Method (c) showed the $6d_{z^2}$ and $6d_{xz}$ are lowest 6d orbitals and was used to give rough $5f^3 \rightarrow 5f^2 6d^1$ transition energies to compare to UV-vis-NIR. Adding 6d orbitals to the active space and averaging over more states resulted in a poorer reproduction of the ground state magnetic properties and a decreased stabilisation of the $^4I_{9/2}$ ground state. Including all five 6d orbitals orbitals did not add any quartet states below 22,000 cm^{-1} compared to the CAS(3,9) calculation in method (c).

The spin-free wavefunctions obtained from CASSCF calculations were mixed by spin-orbit coupling in the RASSI module. The *g*-values, magnetisation *vs*. field and magnetic susceptibility were calculated using SINGLE_ANISO, and the spin-orbit wavefunctions of Ce, Nd and U complexes were decomposed into their CF wavefunctions, with the quantisation axis

defined by the g_1 direction in the ground doublet.[63] The g-tensors are shown in Figures 7–8 and S134–S135, with images generated using Diamond.[63] In the tables below (Tables S14–S23) we report the energy, g-values and composition of the doubly degenerate Kramers doublet states, and the angle between the g_1 direction in the excited state and ground state. Finally we show the state averaged orbitals in the CAS(3,9)SCF calculation on **3-U** (Figure S136–S137) and the state energies for the CAS(3,7)SCF calculation over 35 quartets and 112 doublets, and the CAS(3,9)SCF calculation (Figure S138).

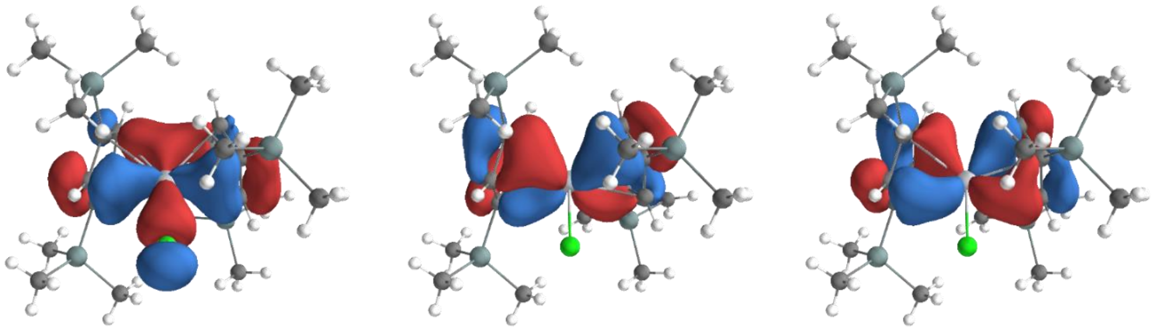

Occupancy: 1.98 Occupancy: 1.97 Occupancy: 1.98

Figure S122. State-averaged molecular orbitals of **1-Ti** included in the CAS(7,8) active space along with five 3d orbitals. Isosurface at 0.04 e Å^{-3}.

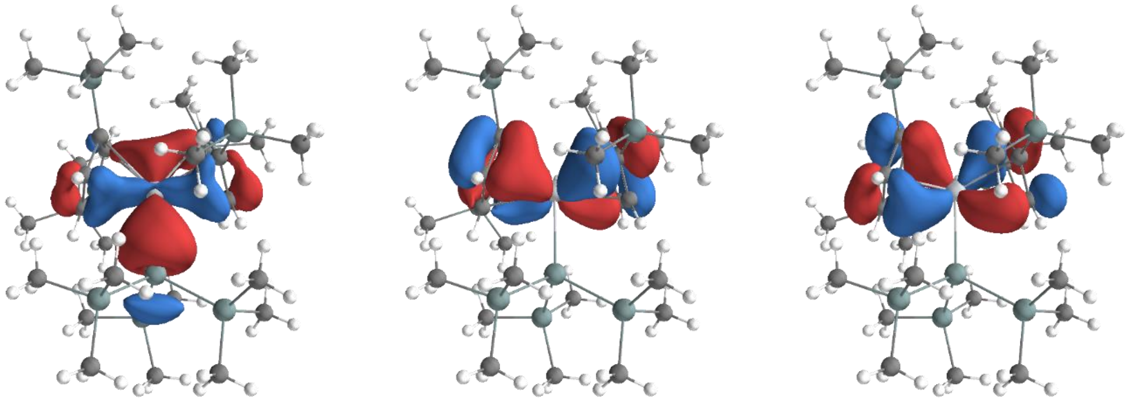

Occupancy: 1.96 Occupancy: 1.97 Occupancy: 1.98

Figure S123. State-averaged molecular orbitals of **3-Ti** included in the CAS(7,8) active space along with five 3d orbitals. Isosurface at 0.04 e Å^{-3}.

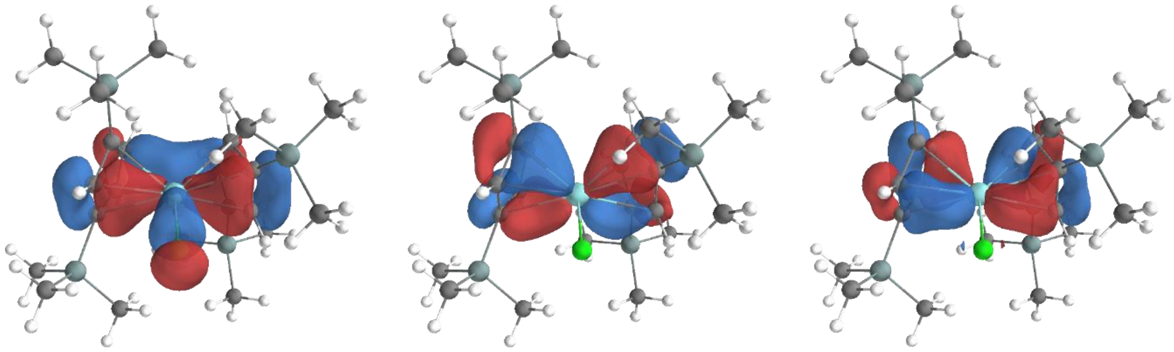

Occupancy: 1.99 Occupancy: 1.99 Occupancy: 1.99

Figure S124. State-averaged molecular orbitals of **1-Zr** included in the CAS(7,8) active space along with five 3d orbitals. Isosurface at 0.04 e Å^{-3}.

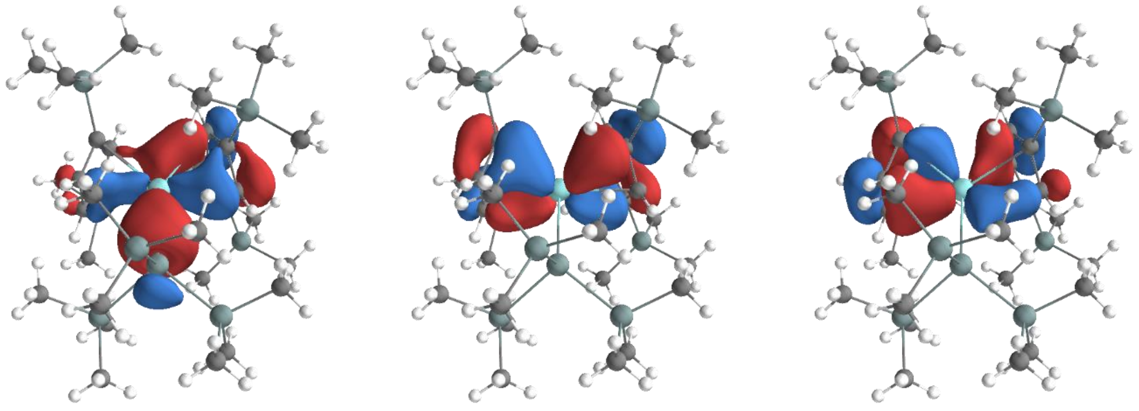

Occupancy: 1.99 Occupancy: 1.99 Occupancy: 1.99

Figure S125. State-averaged molecular orbitals of **3-Zr** included in the CAS(7,8) active space along with five 3d orbitals. Isosurface at 0.04 e Å^{-3}.

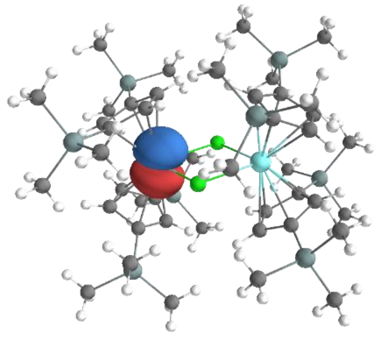

Occupancy: 1.99

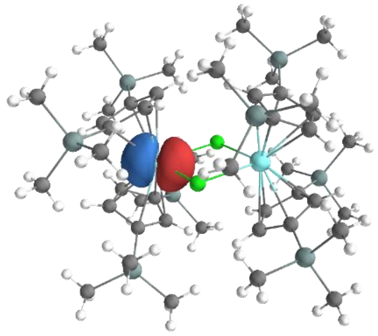

Occupancy: 1.99

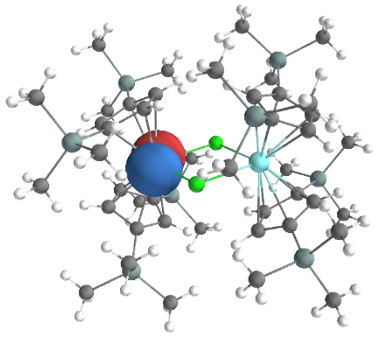

Occupancy: 1.99

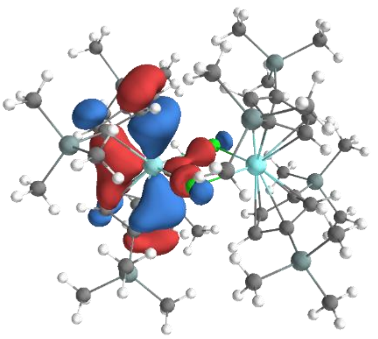

Occupancy: 1.99

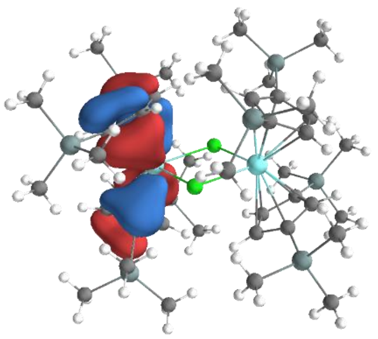

Occupancy: 1.99

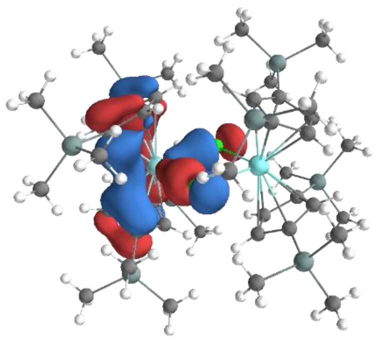

Occupancy: 1.99

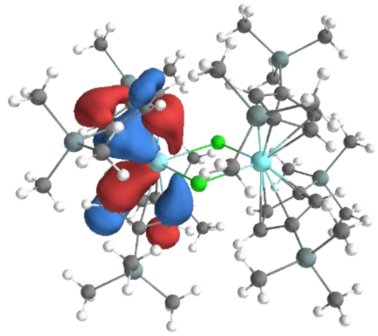

Occupancy: 1.99

Figure S126. State-averaged molecular orbitals of **2-Zr′** included in the CAS(7,8) active space along with five 4d orbitals. Isosurface at 0.04 e Å^{-3}.

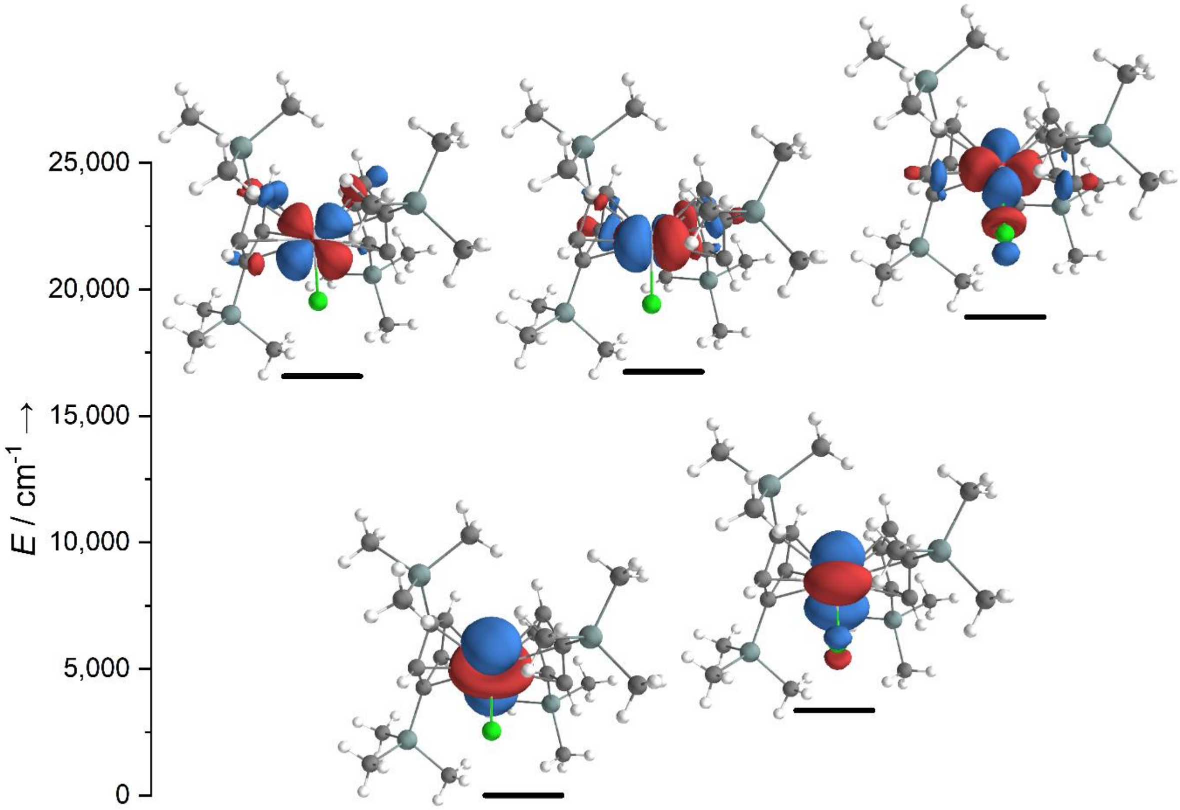

Figure S127. Approximate 3d-orbital energy diagram of **1-Ti** derived from energies of singly occupied state-specific natural orbitals. Isosurface at 0.04 e Å^{-3}. Near-degenerate 3d$_{xy}$ and 3d$_{yz}$ orbitals are significantly mixed.

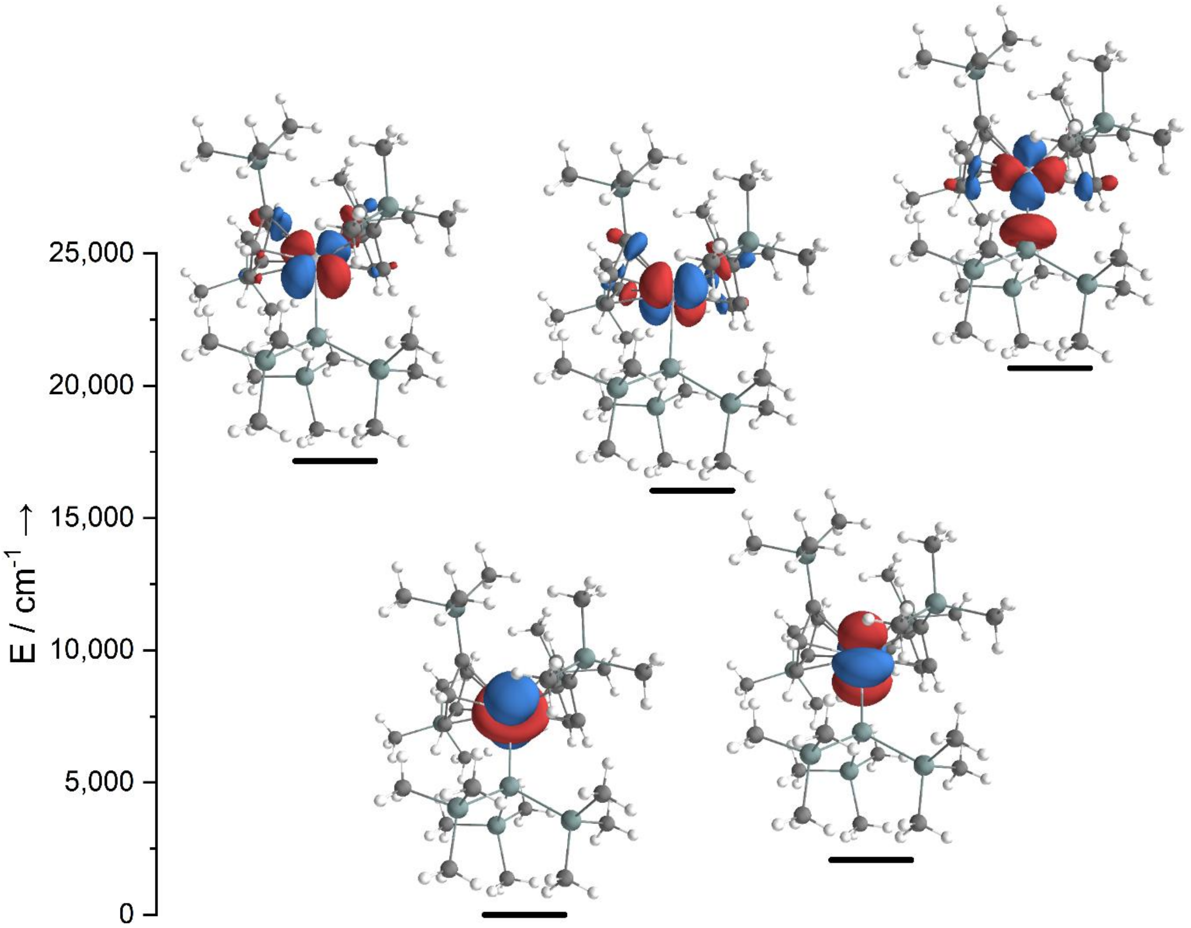

Figure S128. Approximate 3d orbital energy diagram of **3-Ti** derived from energies of singly occupied state-specific natural orbitals. Isosurface at 0.04 e Å^{-3}.

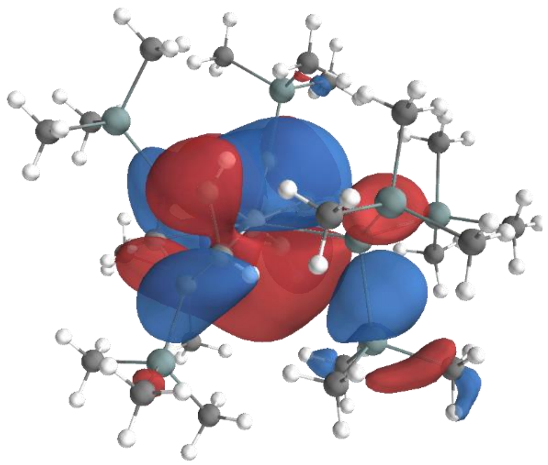

Figure S129. Singly occupied state-specific natural orbital for second root of **3-Ti**. Isosurface at 0.005 e Å^{-3}.

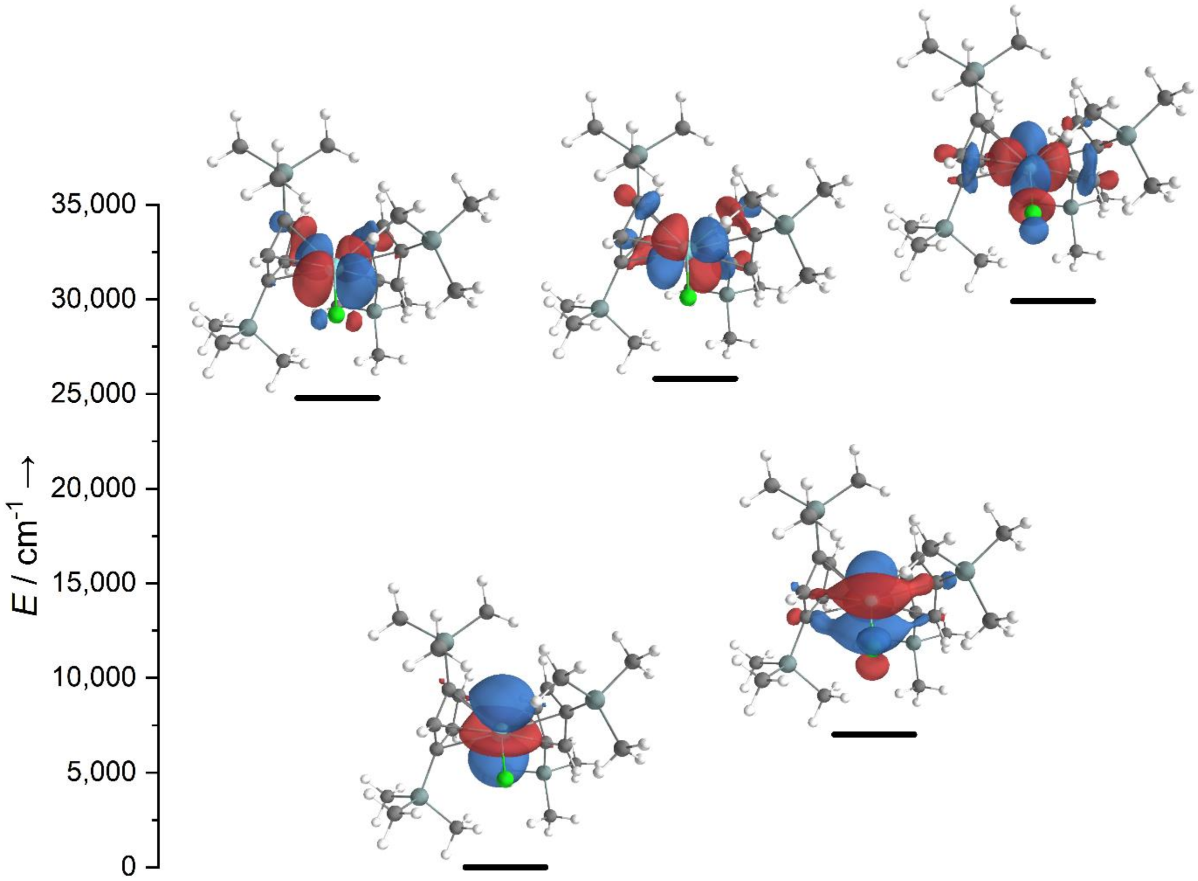

Figure S130. Approximate 4d orbital energy diagram of **1-Zr** derived from energies of singly occupied state-specific natural orbitals. Isosurface at 0.04 e Å^{-3}.

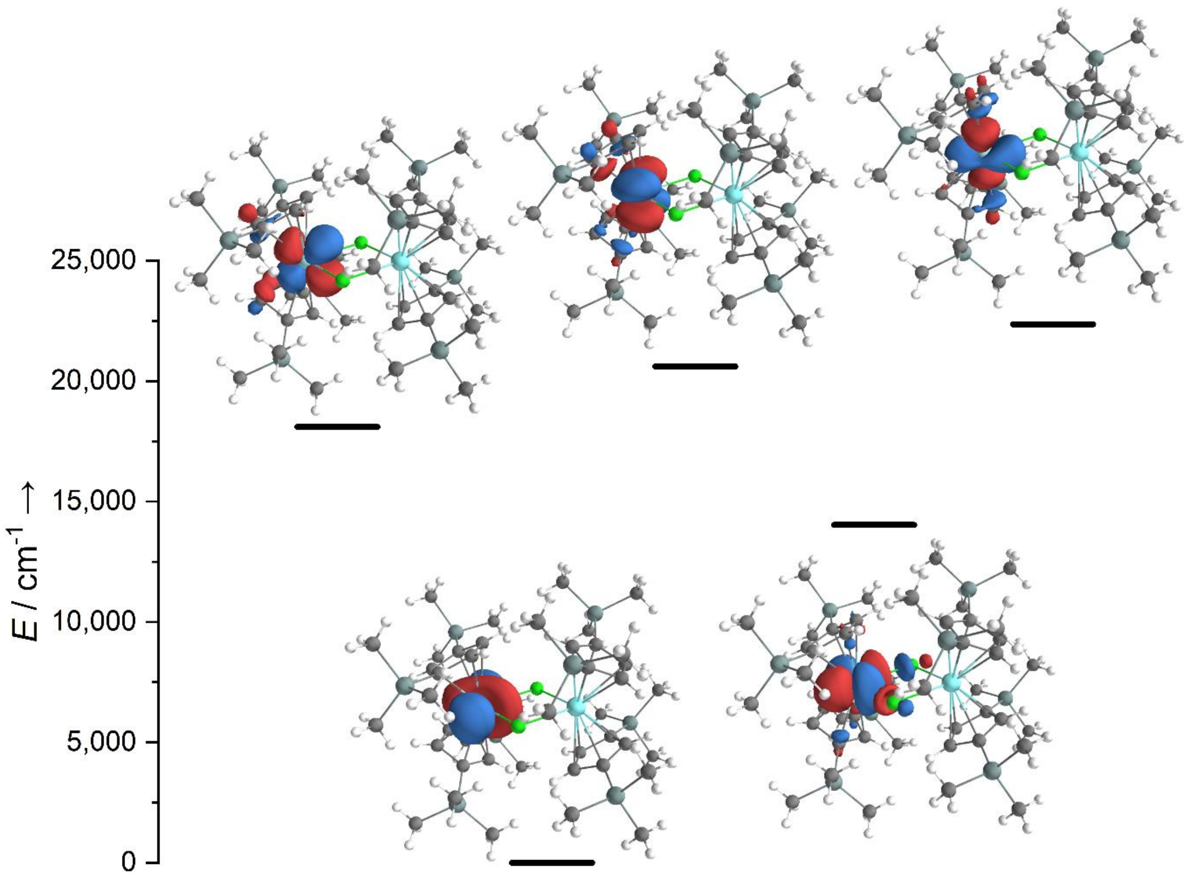

Figure S131. Approximate 4d orbital energy diagram of **2-Zr′** derived from energies of singly occupied state-specific natural orbitals. Isosurface at 0.04 e Å^{-3}. Note that orbitals are orientated differently to other orbital energy diagrams.

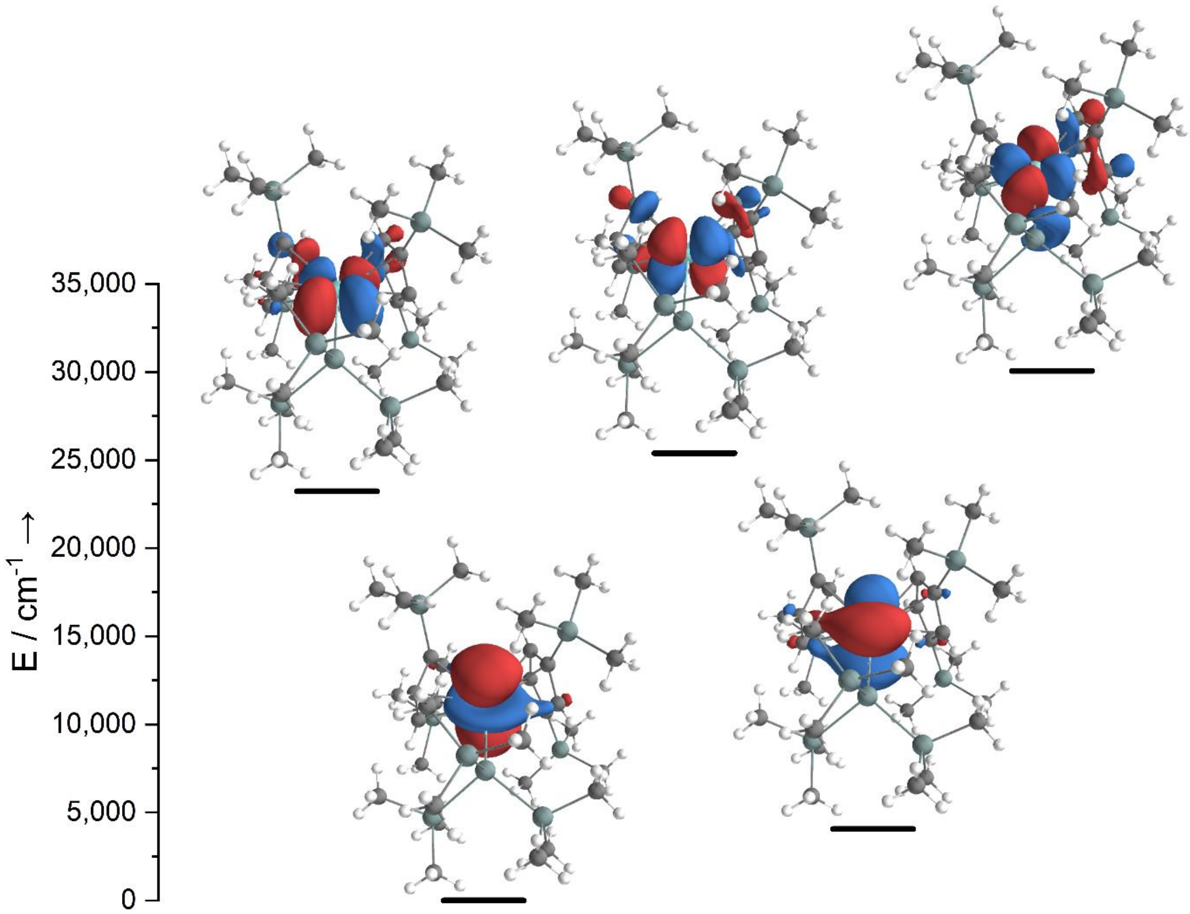

Figure S132. Approximate 4d orbital energy diagram of **3-Zr** derived from energies of singly occupied state-specific natural orbitals. Isosurface at 0.04 e Å^{-3}.

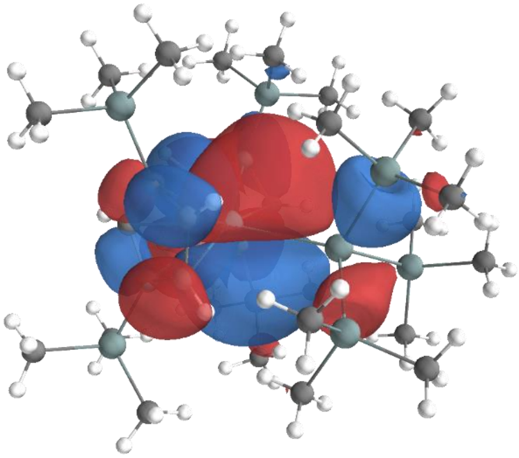

Figure S133. Singly occupied state-specific natural orbital for second root of **3-Zr**. Isosurface at 0.01 e Å^{-3}.

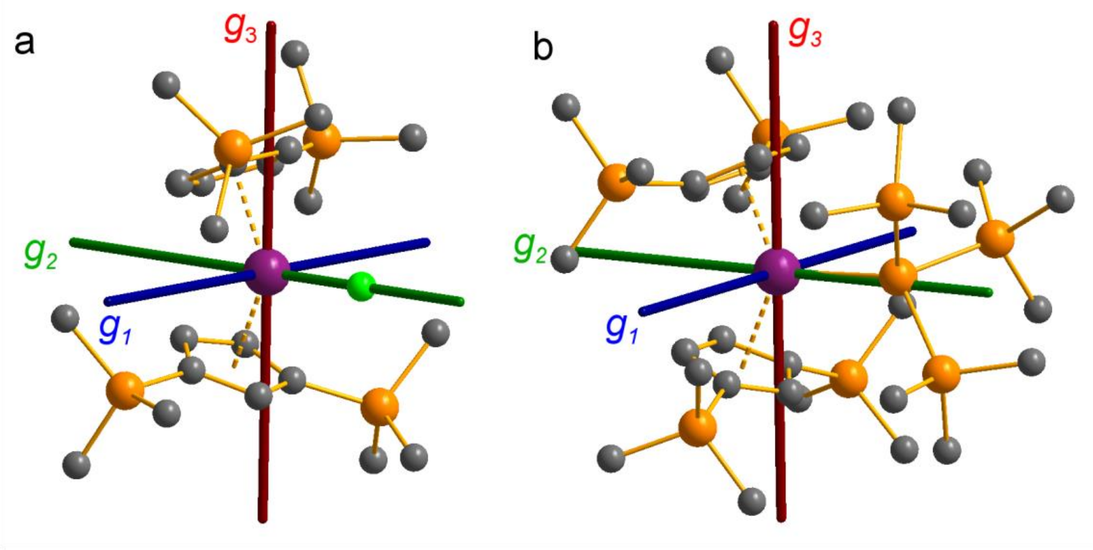

Figure S134. CASSCF-calculated magnetic axes (blue: g_1, most magnetic; green: g_2, intermediate; red: g_3, least magnetic) for (a) **1-Zr** and (b) **3-Zr**. Transition metal, silicon, chlorine and carbon shown as purple, orange, green and grey respectively. Hydrogen atoms are excluded for clarity.

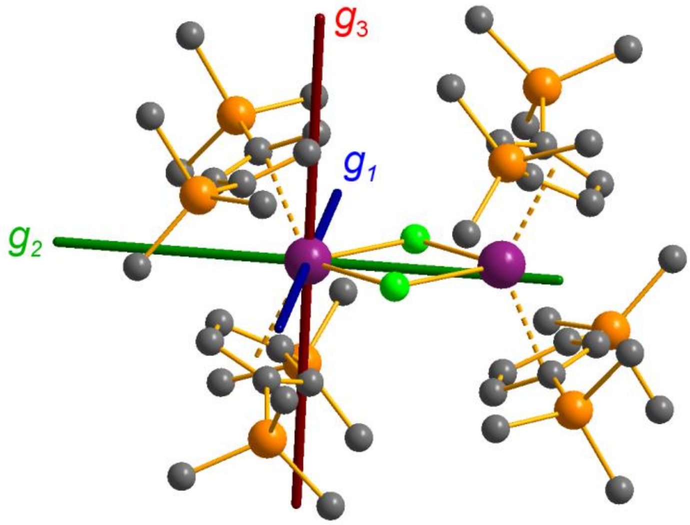

Figure S135. CASSCF-calculated magnetic axes (blue: g_1, most magnetic; green: g_2, intermediate; red: g_3, least magnetic) for **2-Zr′**. Transition metal, silicon, chlorine and carbon shown as purple, orange, green and grey respectively. Hydrogen atoms are excluded for clarity.

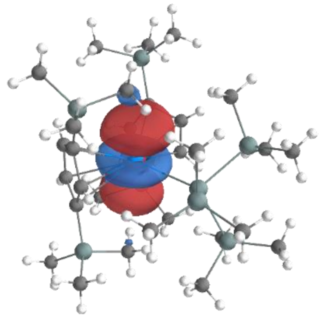

Occupancy: 0.27

Spin quartet

Composition: 63% U 6d_{z^2}+ 4% U 7s

+ 4% U 6d_{xz} + 3% U 8s + 2% U 7d_{z^2}

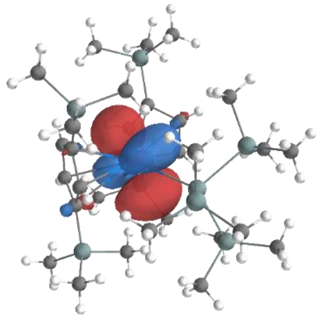

Occupancy: 0.27

Spin quartet

Composition: 68% 6d_{xz} + 4% 6d_{z^2}

+ 3% Si 3d_{xz} + 2% U 7p_z

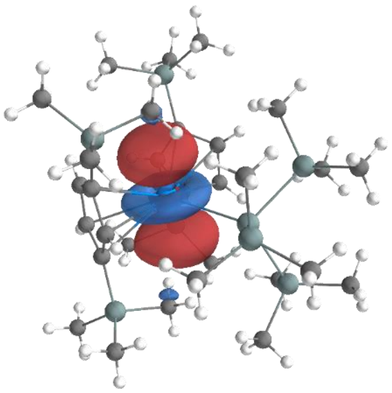

Occupancy: 0.24

Spin doublet

Composition: 61% U 6d_{z^2} + 6% U 6d_{xz}

+ 4% U 7s + 3% U 8s

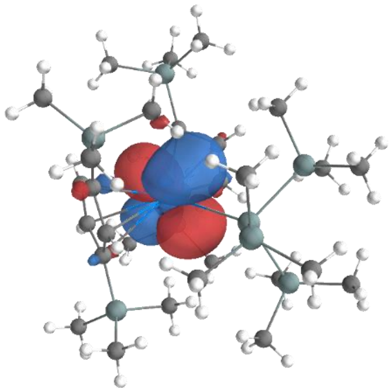

Occupancy: 0.24

Spin doublet

Composition: 65% 6d_{xz} + 5% 6d_{z^2}

+ 3% Si 3d_{xz} + 2% U 7p_z

Figure S136. 6d-based state-averaged molecular orbitals of **3-U** included in the CAS(3,9) active space along. Isosurface at 0.04 e Å^{-3}, wavefunction composition cut off is 2%.

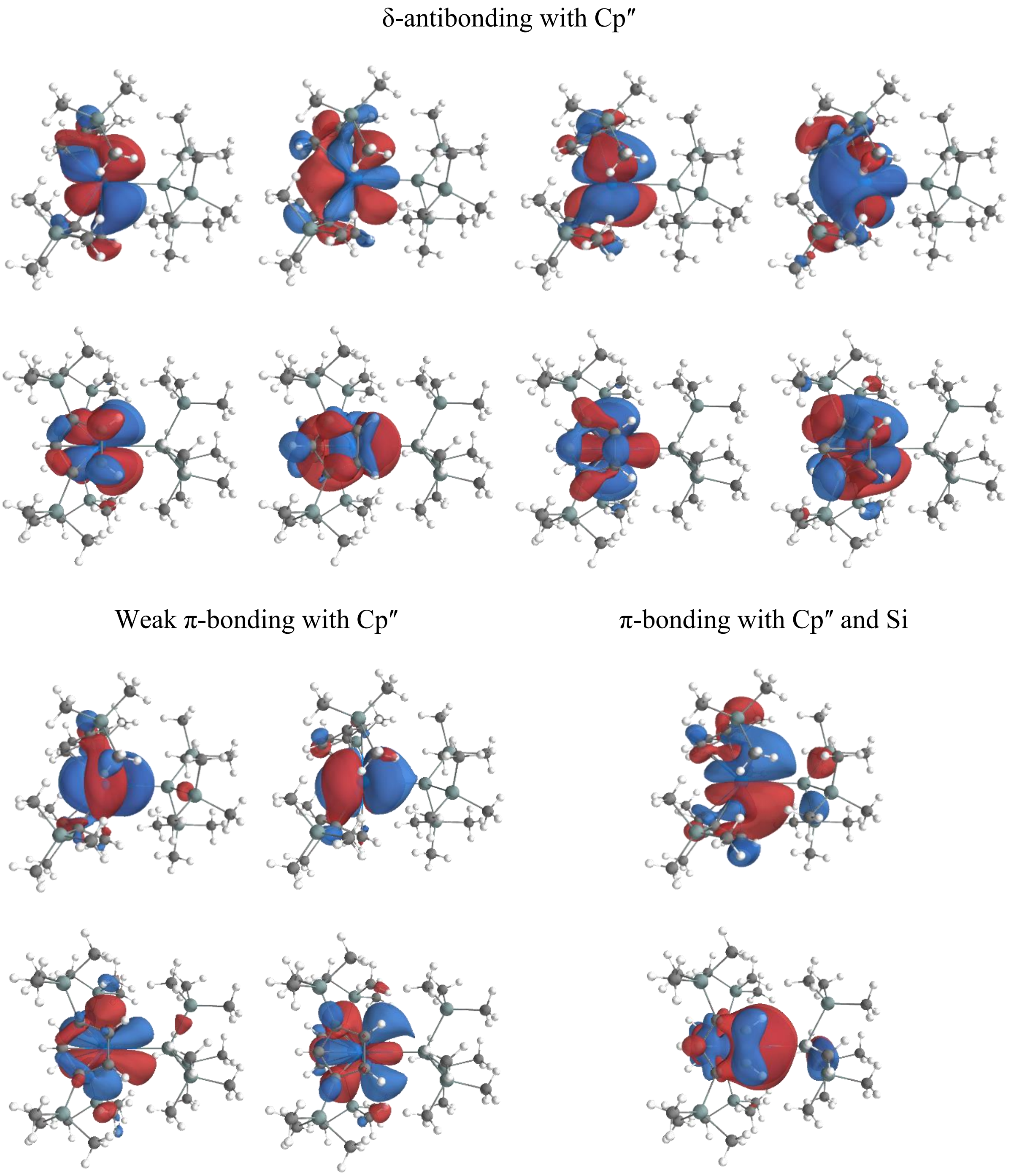

Figure S137. 5f-based state-averaged spin quartet molecular orbitals of **3-U** included in the CAS(3,9) active space. Two orientations shown. Isosurface at 0.005 e Å^{-3}.

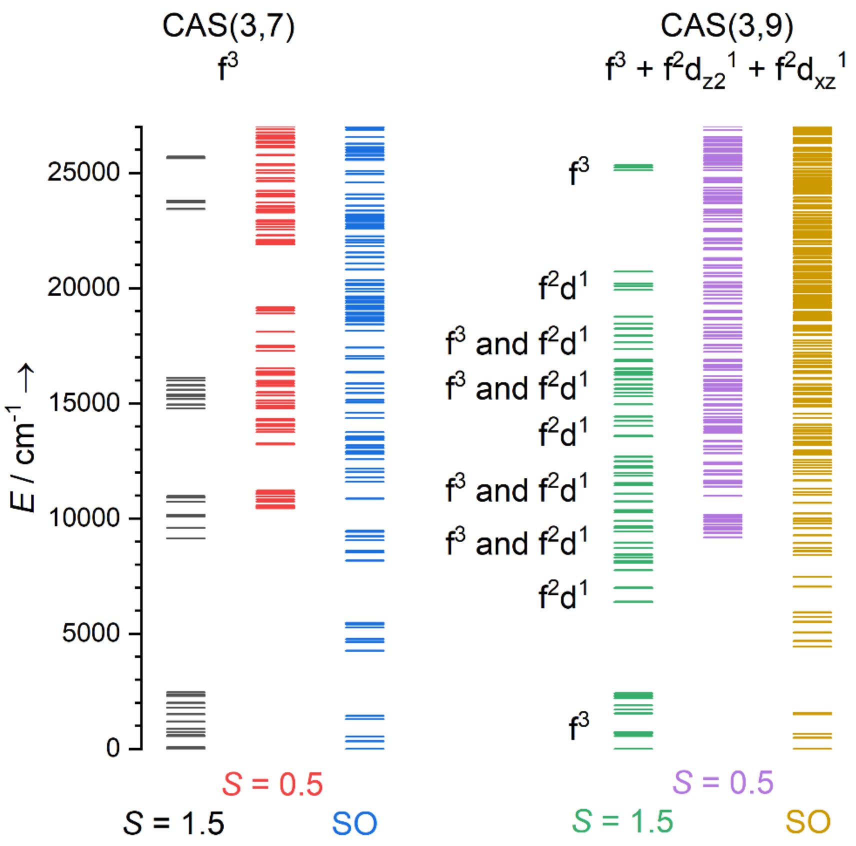

Figure S138. Energies of spin quartets (*S* = 1.5), spin doublets (*S* = 0.5) and spin–orbit (SO) states for **3-U**, calculated using CAS(3,7) active space averaging over 35 quartets and 112 doublets (left) and CAS(3,9) active space averaging over 77 quartets and 210 doublets (right). Approximate state composition indicated for spin quartet states for CAS(3,9) calculation.

Table S14. Electronic structure of **1-Ti** as calculated with CASSCF-SO.

**Energy (cm^{-1})**	g_1	g_2	g_3	**Angle (°)**	**Singly occupied orbital** [a]
0	1.9994	1.9655	1.8370	-	88% $3d_{z^2}$ + 1.2% 4s
3357.17	2.1553	1.9874	1.9827	89.73	86% $3d_{xz}$ + 3% $4d_{xz}$
16569.48	1.4419	1.1642	0.5537	59.67	43% $3d_{xy}$ + 29% $3d_{yz}$ + 6% $4d_{xy}$ + 5% $4d_{yz}$
16744.97	3.4279	1.359	1.2862	74.25	41% $3d_{yz}$ + 30% $3d_{xy}$ + 6% $4d_{yz}$ + 4% $4d_{xy}$
18906.60	2.3111	2.0646	2.0055	4.13	69% $3d_{x^2-y^2}$ + 11% $4d_{x^2-y^2}$ + 2% $3d_{yz}$

a = Major (>1%) Ti orbital contributions to singly occupied state-specific natural orbital. *x*-axis is Ti–Cl bond, *y*-axis is tangential to Cp''_{cent}–Ti–Cp''_{cent}, *z*-axis is perpendicular to (Cl,Cp''_{cent},Cp''_{cent}) plane.

Table S15. Electronic structure of **3-Ti** as calculated with CASSCF-SO.

**Energy (cm^{-1})**	g_1	g_2	g_3	**Angle (°)**	**Singly occupied orbital** [a]
0	1.9950	1.9595	1.7173	-	89% $3d_{z^2}$ + 1.2% 4s
2073.58	2.2776	1.9843	1.9798	89.86	90% $3d_{xz}$ + 1.1% $4p_z$ + 1.0% $4d_{xz}$
16037.19	2.0058	1.9833	1.7905	24.66	71% $3d_{yz}$ + 10% $4d_{yz}$ + 2% $3d_{xy}$ + 1.0% $5d_{yz}$
17156.93	2.2102	1.9946	1.7962	89.94	72% $3d_{xy}$ + 9% $4d_{xy}$ + 1.5% $3d_{yz}$
20665.63	2.2018	2.0326	2.0082	4.23	65% $3d_{x^2-y^2}$ + 13% $4d_{x^2-y^2}$ + 1.3% $5d_{x^2-y^2}$

a = Major (>1%) Ti orbital contributions to singly occupied state-specific natural orbital. *x*-axis is Ti–Si bond, *y*-axis is tangential to Cp''_{cent}–Ti–Cp''_{cent}, *z*-axis is perpendicular to (Si,Cp''_{cent},Cp''_{cent}) plane.

Table S16. Electronic structure of **1-Zr** as calculated with CASSCF-SO.

Energy (cm^{-1})	g_1	g_2	g_3	Angle (°)	Singly occupied orbital [a]
0	1.9973	1.9524	1.8214	-	74% $4d_{z^2}$ + 4% 5s + 1.0% 6s
7010.58	2.1639	1.9845	1.9676	89.65	72% $4d_{xz}$ + 2% $5d_{xz}$ + 2% $5p_z$ + 1.1% $6d_{xz}$
24787.88	1.8751	1.5109	1.1915	89.99	60% $4d_{xy}$ + 12% $5d_{xy}$ + 1.2% $6d_{xy}$
25811.97	2.7861	1.8191	1.7895	80.92	58% $4d_{yz}$ + 12% $5d_{yz}$ + 1.4% $5f_{xyz}$ + 1.2% $4f_{xyz}$
29895.60	2.3079	2.1031	2.0027	1.67	55% $4d_{x^2-y^2}$ + 14% $5d_{x^2-y^2}$

a = Major (>1%) Zr orbital contributions to singly occupied state-specific natural orbital. *x*-axis is Zr–Cl bond, *y*-axis is tangential to Cp″$_{cent}$–Zr–Cp″$_{cent}$, *z*-axis is perpendicular to (Cl,Cp″$_{cent}$,Cp″$_{cent}$) plane.

Table S17. Electronic structure of **2-Zr′** as calculated with CASSCF-SO.

Energy (cm^{-1})	g_1	g_2	g_3	Angle (°)	Singly occupied orbital [a]
0	1.9944	1.9224	1.8262	-	78% $4d_{z^2}$ + 5% $5d_{z^2}$ + 3% $4d_{x^2-y^2}$
14030.54	2.1120	1.9219	1.8523	89.14	66% $4d_{xz}$ + 13% $5d_{xz}$ + 1.1% $5f_{z(x^2-y^2)}$
18101.45	2.0774	1.5092	1.2769	88.53	64% $4d_{xy}$ + 14% $5d_{xy}$
20608.77	2.3920	1.8059	1.4109	86.79	61% $4d_{yz}$ + 17% $5d_{yz}$
22362.47	2.6055	2.4552	1.9599	77.15	58% $4d_{x^2-y^2}$ + 18% $5d_{x^2-y^2}$ + 1.8% $4d_{z^2}$

a = Major (>1%) Zr orbital contributions to singly occupied state-specific natural orbital. *x*-axis is along Zr–Y, *y*-axis is tangential to Cp″$_{cent}$–Zr–Cp″$_{cent}$, *z*-axis is perpendicular to (Y,Cp″$_{cent}$,Cp″$_{cent}$) plane, in $ZrYCl_2$ plane.

Table S18. Electronic structure of **3-Zr** as calculated with CASSCF-SO.

**Energy (cm^{-1})**	g_1	g_2	g_3	**Angle (°)**	**Singly occupied orbital** [a]
0	1.9897	1.9438	1.6719	-	75% $4d_{z^2}$ + 3% 5s
4055.54	2.3173	1.9774	1.9640	89.95	74% $4d_{xz}$ + 3% $4p_z$
23224.19	1.9933	1.7607	1.6475	87.75	59% $4d_{xy}$ + 8% $5d_{xy}$ + 1.8% $5f_{y(3x^2-y^2)}$ + 1.4% $4f_{y(3x^2-y^2)}$ + 1.0% $4d_{yz}$
25401.96	2.3478	1.9706	1.9303	87.14	58% $4d_{yz}$ + 11% $5d_{yz}$+ 1.5% $5f_{xyz}$ + 1.2% $4f_{xyz}$ + 1.0% $4d_{xy}$
30058.01	2.2055	2.0792	2.0077	5.32	51% $4d_{x^2-y^2}$ + 16% $5d_{x^2-y^2}$ + 1.4% $6d_{x^2-y^2}$

a = Major (>1%) Zr orbital contributions to singly occupied state-specific natural orbital. *x*-axis is Zr–Si bond, *y*-axis is tangential to Cp″$_{cent}$–Zr–Cp″$_{cent}$, *z*-axis is perpendicular to (Si,Cp″$_{cent}$,Cp″$_{cent}$) plane.

Table S19. Electronic structure of **3-Ce** as calculated with CASSCF-SO.

**Energy (cm^{-1})**	g_1	g_2	g_3	**Angle (°)**	**Wavefunction** [a]	**$\langle J_z \rangle$**
0	3.7754	1.0161	0.5616	-	90%$\lvert\pm 5/2\rangle$ + 8%$\lvert\pm 1/2\rangle$	$\pm$2.252
266.12	2.3951	1.2682	1.0668	89.80	72%$\lvert\pm 3/2\rangle$ + 21%$\lvert\mp 1/2\rangle$ + 7%$\lvert\mp 5/2\rangle$	$\pm$0.799
995.48	4.2294	0.3332	0.087	89.99	69%$\lvert\pm 1/2\rangle$ + 24%$\lvert\mp 3/2\rangle$	$\pm$0.052

a = Contributions > 5%.

Table S20. Electronic structure of **3-Nd** as calculated with CASSCF-SO.

**Energy (cm^{-1})**	g_1	g_2	g_3	**Angle (°)**	**Wavefunction** [a]	**<J_z>**
0	5.5262	0.2961	0.1460	-	76%$\lvert\pm 9/2\rangle$ + 15%$\lvert\pm 5/2\rangle$ + 5%$\lvert\pm 1/2\rangle$	$\pm$3.811
65.49	3.5461	2.4364	0.0254	72.01	46%$\lvert\pm 7/2\rangle$ + 38%$\lvert\pm 3/2\rangle$ + 8%$\lvert\mp 9/2\rangle$	$\pm$1.800
176.45	2.8242	1.9832	1.3770	56.29	44%$\lvert\pm 5/2\rangle$ + 28%$\lvert\pm 1/2\rangle$ + 15%$\lvert\pm 9/2\rangle$ + 8%$\lvert\mp 7/2\rangle$	$\pm$1.614
465.06	4.7213	1.4527	0.0925	84.97	29%$\lvert\pm 3/2\rangle$ + 25%$\lvert\mp 1/2\rangle$ + 22%$\lvert\pm 7/2\rangle$ + 16%$\lvert\mp 5/2\rangle$	$\pm$0.724
477.22	4.7800	1.8207	1.1046	80.75	33%$\lvert\pm 1/2\rangle$ + 26%$\lvert\mp 3/2\rangle$ + 20%$\mp 7/2\rangle$ + 15%$\pm 3/2\rangle$	$\pm$0.582

a = Contributions > 5%.

Table S21. Electronic structure of **3-U** as calculated with CAS(3,7)SCF-SO, with seven 5f orbitals, averaging over 35 quartets and 112 doublets.

**Energy (cm^{-1})**	g_1	g_2	g_3	**Angle (°)**	**Wavefunction** [a]	**<J_z>**
0	6.1303	0.0763	0.0072	-	95%$\lvert\pm 9/2\rangle$	$\pm$4.364
330.04	3.3128	1.6192	0.1407	3.66	49%$\lvert\pm 7/2\rangle$ + 46%$\lvert\pm 3/2\rangle$	$\pm$2.368
524.06	2.4919	1.0846	0.9060	0.86	59%$\lvert\pm 5/2\rangle$ + 34%$\lvert\pm 1/2\rangle$	$\pm$1.726
1288.38	5.2370	0.3269	0.1794	88.17	56%$\lvert\pm 1/2\rangle$ + 31%$\lvert\pm 5/2\rangle$ + 8%$\lvert\mp 7/2\rangle$	$\pm$0.714
1426.90	3.0702	2.3986	1.9288	87.29	46%$\lvert\pm 3/2\rangle$ + 41%$\lvert\pm 7/2\rangle$ + 9%$\lvert\mp 1/2\rangle$	$\pm$2.007

a = Contributions > 5%.

Table S22. Electronic structure of **3-U** as calculated with CAS(3,7)SCF-SO, with seven 5f orbitals, averaging over 13 quartets.

Energy (cm^{-1})	g_1	g_2	g_3	Angle (°)	Wavefunction [a]	<J_z>
0	5.7623	0.0485	0.0339	-	89%$\vert\pm 9/2\rangle$ + 6%$\vert\pm 5/2\rangle$	$\pm$4.226
291.63	3.2091	1.5306	0.0513	5.07	49%$\vert\pm 3/2\rangle$ + 43%$\vert\pm 7/2\rangle$	$\pm$2.342
475.71	2.5727	0.9921	0.8290	6.61	52%$\vert\pm 5/2\rangle$ + 37%$\vert\pm 1/2\rangle$ + 7%$\vert\pm 9/2\rangle$	$\pm$1.717
1315.21	4.3401	1.2332	1.1242	88.3	55%$\vert\pm 1/2\rangle$ + 36%$\vert\pm 5/2\rangle$ + 6%$\vert\mp 7/2\rangle$	$\pm$0.979
1535.97	2.8019	2.1935	1.4945	3.82	48%$\vert\pm 7/2\rangle$ + 44%$\vert\pm 3/2\rangle$ + 5%$\vert\mp 1/2\rangle$	$\pm$2.253

a = Contributions > 5%.

Table S23. Electronic structure of **3-U** as calculated with CAS(3,9)SCF-SO, with seven 5f orbitals, $6d_{z^2}$ and $6d_{xz}$, averaging over 77 quartets and 210 doublets.

Energy (cm^{-1})	g_1	g_2	g_3	Angle (°)	Wavefunction [a]	<J_z>
0	6.4034	0.0181	0.0068	-	99%$\vert\pm 9/2\rangle$	$\pm$4.456
483.04	3.6040	2.4136	0.7440	59.92	45%$\vert\pm 3/2\rangle$ + 43%$\vert\pm 7/2\rangle$ + 5% $\vert\mp 5/2\rangle$	$\pm$2.057
656.11	2.6424	1.6064	1.4910	56.41	61%$\vert\pm 5/2\rangle$ + 26%$\vert\pm 1/2\rangle$ + 6% $\vert\mp 7/2\rangle$	$\pm$1.436
1506.60	4.2991	0.8477	0.1833	82.13	55%$\vert\pm 1/2\rangle$ + 27%$\vert\pm 5/2\rangle$ + 9%$\vert\mp 7/2\rangle$ + 6%$\vert\mp 3/2\rangle$	$\pm$0.577
1551.97	3.3707	2.7444	1.1555	87.93	43%$\vert\pm 3/2\rangle$ + 40%$\vert\pm 7/2\rangle$ + 11%$\vert\mp 1/2\rangle$	$\pm$1.926

a = Contributions > 5%.

15. References

1 B. L. L. Réant, V. E. J. Berryman, J. A. Seed, A. R. Basford, A. Formanuik, A. J. Wooles, N. Kaltsoyannis, S. T. Liddle and D. P. Mills, *Chem. Commun.*, 2020, **56**, 12620–12623.

2 B. L. L. Réant, V. E. J. Berryman, A. R. Basford, L. E. Nodaraki, A. J. Wooles, F. Tuna, N. Kaltsoyannis, D. P. Mills and S. T. Liddle, *J. Am. Chem. Soc.*, 2021, **143**, 9813–9824.

3 P. B. Hitchcock, M. F. Lappert, L. Maron and A. V. Protchenko, *Angew. Chem. Int. Ed.*, 2008, **47**, 1488–1491.

4 L. E. Manxzer, J. Deaton, P. Sharp and R. R. Schrock, *Inorg. Synth.*, 1983, **21**, 135–140.

5 T. Cantat, B. L. Scott and J. L. Kiplinger, *Chem. Commun.*, 2010, **46**, 919–921.

6 D. Patel, A. J. Wooles, E. Hashem, H. Omorodion, R. J. Baker and S. T. Liddle, *New J. Chem.*, 2015, **39**, 7559–7562.

7 N. A. Jones, S. T. Liddle, C. Wilson and P. L. Arnold, *Organometallics*, 2007, **26**, 755–757.

8 K. Izod, S. T. Liddle and W. Clegg, *Inorg. Chem.*, 2004, **43**, 214–218.

9 A. J. Gaunt, A. E. Enriquez, S. D. Reilly, B. L. Scott and M. P. Neu, *Inorg. Chem.*, 2008, **47**, 26–28.

10 D. E. Bergbreiter and J. M. Killough, *J. Am. Chem. Soc.*, 1978, **100**, 2126–2134.

11 M. J. Harvey, T. P. Hanusa and M. Pink, *J. Chem. Soc. Dalton Trans.*, 2001, 1128–1130.

12 Y. A. Ustynyuk, Y. N. Luzikov, V. I. Mstislavsky, A. A. Azizov and I. M. Pribytkova, *J. Organomet. Chem.*, 1975, **96**, 335–353.

13 P. Jutzi and R. Sauer, *J. Organomet. Chem.*, 1973, **50**, C29–C30.

14 C. Marschner, *Eur. J. Inorg. Chem.*, 1998, 221–226.

15 A. Antiñolo, M. F. Lappert, A. Singh, D. J. W. Winterborn, L. M. Engelhardt, C. L. Raston, A. H. White, A. J. Carty and N. J. Taylor, *J. Chem. Soc. Dalton Trans.*, 1987,

1, 1463–1472.

16 P. C. Blake, M. F. Lappert, R. G. Taylor, J. L. Atwood, W. E. Hunter and H. Zhang, *J. Chem. Soc. Dalton Trans.*, 1995, 3335–3341.

17 M. F. Lappert, W. P. Leung, R. A. Bartlett and P. P. Power, *Polyhedron*, 1989, **8**, 1883.

18 P. B. Hitchcock, M. F. Lappert, G. A. Lawless, H. Olivier and E. J. Ryan, *J. Chem. Soc. Chem. Commun.*, 1992, 474–476.

19 F. Ortu, J. M. Fowler, M. Burton, A. Formanuik and D. P. Mills, *New J. Chem.*, 2015, **39**, 7633–7639.

20 J. K. Peterson, M. R. Macdonald, J. W. Ziller and W. J. Evans, *Organometallics*, 2013, **32**, 2625–2631.

21 R. R. Langeslay, M. E. Fieser, J. W. Ziller, F. Furche and W. J. Evans, *Chem. Sci.*, 2014, **6**, 517–521.

22 W. J. Evans, R. A. Keyer and J. W. Ziller, *J. Organomet. Chem.*, 1990, **394**, 87–97.

23 *CrysAlis PRO*, Agilent Technologies Ltd, Yarnton, Oxfordshire, England, 2014.

24 G. M. Sheldrick, *Acta Crystallogr. Sect. A*, 2015, **71**, 3–8.

25 G. M. Sheldrick, *Acta Crystallogr. Sect. C*, 2015, **71**, 3–8.

26 O. V Dolomanov, L. J. Bourhis, R. J. Gildea, J. A. K. Howard and H. Puschmann, *J. Appl. Crystallogr.*, 2009, **42**, 339–341.

27 L. J. Farrugia, *J. Appl. Crystallogr.*, 2012, **45**, 849–854.

28 Persistence of Vision Raytracer Pty. Ltd., *Persistence of Vision Raytracer*, v.3.7, 2013. Retrieved from http://www.povray.org/download/.

29 S. T. Liddle and J. van Slageren, *Chem. Soc. Rev.*, 2015, **44**, 6655–6669.

30 D. Reta and N. F. Chilton, *Phys. Chem. Chem. Phys.*, 2019, **21**, 23567–23575.

31 N. F. Chilton, *Annu. Rev. Mater. Res.*, 2022, **52**, 79–101.

32 J. D. Rinehart and J. R. Long, *J. Am. Chem. Soc.*, 2009, **131**, 12558–12559.

33 J. D. Rinehart, K. R. Meihaus and J. R. Long, *J. Am. Chem. Soc.*, 2010, **132**, 7572–7573.

34 K. R. Meihaus, J. D. Rinehart and J. R. Long, *Inorg. Chem.*, 2011, **50**, 8484–8489.

35 M. A. Antunes, L. C. J. Pereira, I. C. Santos, M. Mazzanti, J. Marçalo and M. Almeida, *Inorg. Chem.*, 2011, **50**, 9915–9917.

36 J. D. Rinehart and J. R. Long, *Dalton Trans.*, 2012, **41**, 13572–13574.

37 J. T. Coutinho, M. A. Antunes, L. C. J. Pereira, H. Bolvin, J. Marçalo, M. Mazzanti and M. Almeida, *Dalton Trans.*, 2012, **41**, 13568–13571.

38 F. Moro, D. P. Mills, S. T. Liddle and J. Van Slageren, *Angew. Chem. Int. Ed.*, 2013, **52**, 3430–3433.

39 J. T. Coutinho, M. A. Antunes, L. C. J. Pereira, J. Marçalo and M. Almeida, *Chem. Commun.*, 2014, **50**, 10262–10264.

40 J. J. Le Roy, S. I. Gorelsky, I. Korobkov and M. Murugesu, *Organometallics*, 2015, **34**, 1415–1418.

41 R. F. Higgins, C. J. Tatebe, S. C. Bart and M. P. Shores, *Chem. Commun.*, 2019, **55**, 10611–10614.

42 S. Stoll and A. Schweiger, *J. Magn. Reson.*, 2006, **178**, 42–55.

43 M. J. Giansiracusa, E. Moreno-Pineda, R. Hussain, R. Marx, M. M. Prada, P. Neugebauer, S. Al-Badran, D. Collison, F. Tuna, J. Van Slageren, S. Carretta, T. Guidi, E. J. L. McInnes, R. E. P. Winpenny and N. F. Chilton, *J. Am. Chem. Soc.*, 2018, **140**, 2504–2513.

44 N. F. Chilton, R. P. Anderson, L. D. Turner, A. Soncini and K. S. Murray, *J. Comput. Chem.*, 2013, **34**, 1164–1175.

45 F. E. Mabbs and D. Collison, *Electron Paramagnetic Resonance of d Transition Metal Compounds*, Elsevier Science, Amsterdam, 1992.

46 M. Křižan, J. Honzíček, J. Vinklárek, Z. Růžičková and M. Erben, *New J. Chem.*, 2014,

39, 576–588.

47 M. J. Frisch, G. W. Trucks, H. B. Schlegel, G. E. Scuseria, M. A. Robb, J. R. Cheeseman, G. Scalmani, V. Barone, G. A. Petersson, H. Nakatsuji, X. Li, M. Caricato, A. Marenich, J. Bloino, B. G. Janesko, R. Gomperts, B. Mennucci, H. P. Hratchian, J. V Ortiz, A. F. Izmaylov, J. L. Sonnenberg, D. Williams-Young, F. Ding, F. Lipparini, F. Egidi, J. Goings, B. Peng, A. Petrone, T. Henderson, D. Ranasinghe, V. G. Zakrzewski, J. Gao, N. Rega, G. Zheng, W. Liang, M. Hada, M. Ehara, K. Toyota, R. Fukuda, J. Hasegawa, M. Ishida, T. Nakajima, Y. Honda, O. Kitao, H. Nakai, T. Vreven, K. Throssell, J. J. A. Montgomery, J. E. Peralta, F. Ogliaro, M. Bearpark, J. J. Heyd, E. Brothers, K. N. Kudin, V. N. Staroverov, T. Keith, R. Kobayashi, J. Normand, K. Raghavachari, A. Rendell, J. C. Burant, S. S. Iyengar, J. Tomasi, M. Cossi, J. M. Millam, M. Klene, C. Adamo, R. Cammi, J. W. Ochterski, R. L. Martin, K. Morokuma, O. Farkas, J. B. Foresman and D. J. Fox, *Gaussian 09 (revision D.01)*, Gaussian, Inc., 2016.

48 J. P. Perdew, K. Burke and M. Ernzerhof, *Phys. Rev. Lett.*, 1996, **77**, 3865–3868.

49 J. P. Perdew, K. Burke and M. Ernzerhof, *Phys. Rev. Lett.*, 1997, **78**, 1396.

50 S. Grimme, J. Antony, S. Ehrlich and H. Krieg, *J. Chem. Phys.*, 2010, **132**, 154104.

51 S. Grimme, S. Ehrlich and L. Goerigk, *J. Comput. Chem.*, 2011, **32**, 1456–1465.

52 T. H. Dunning, *J. Chem. Phys.*, 1989, **90**, 1007–1023.

53 D. E. Woon and T. H. Dunning, *J. Chem. Phys.*, 1993, **98**, 1358–1371.

54 K. L. Schuchardt, B. T. Didier, T. Elsethagen, L. Sun, V. Gurumoorthi, J. Chase, J. Li and T. L. Windus, *J. Chem. Inf. Model.*, 2007, **47**, 1045–1052.

55 D. Feller, *J. Comput. Chem.*, 1996, **17**, 1571–1586.

56 B. P. Pritchard, D. Altarawy, B. Didier, T. D. Gibson and T. L. Windus, *J. Chem. Inf. Model.*, 2019, **59**, 4814–4820.

57 I. F. Galván, M. Vacher, A. Alavi, C. Angeli, F. Aquilante, J. Autschbach, J. J. Bao, S.

I. Bokarev, N. A. Bogdanov, R. K. Carlson, L. F. Chibotaru, J. Creutzberg, N. Dattani, M. G. Delcey, S. S. Dong, A. Dreuw, L. Freitag, L. M. Frutos, L. Gagliardi, F. Gendron, A. Giussani, L. González, G. Grell, M. Guo, C. E. Hoyer, M. Johansson, S. Keller, S. Knecht, G. Kovačević, E. Källman, G. L. Manni, M. Lundberg, Y. Ma, S. Mai, J. P. Malhado, P. Å. Malmqvist, P. Marquetand, S. A. Mewes, J. Norell, M. Olivucci, M. Oppel, Q. M. Phung, K. Pierloot, F. Plasser, M. Reiher, A. M. Sand, I. Schapiro, P. Sharma, C. J. Stein, L. K. Sørensen, D. G. Truhlar, M. Ugandi, L. Ungur, A. Valentini, S. Vancoillie, V. Veryazov, O. Weser, T. A. Wesołowski, P. O. Widmark, S. Wouters, A. Zech, J. P. Zobel and R. Lindh, *J. Chem. Theory Comput.*, 2019, **15**, 5925–5964.

58 D. Andrae, U. Häußermann, M. Dolg, H. Stoll and H. Preuß, *Theor. Chim. Acta*, 1990, **77**, 123–141.

59 F. Aquilante, J. Autschbach, A. Baiardi, S. Battaglia, V. A. Borin, L. F. Chibotaru, I. Conti, L. De Vico, M. Delcey, I. F. Galván, N. Ferré, L. Freitag, M. Garavelli, X. Gong, S. Knecht, E. D. Larsson, R. Lindh, M. Lundberg, P. Å. Malmqvist, A. Nenov, J. Norell, M. Odelius, M. Olivucci, T. B. Pedersen, L. Pedraza-González, Q. M. Phung, K. Pierloot, M. Reiher, I. Schapiro, J. Segarra-Martí, F. Segatta, L. Seijo, S. Sen, D. C. Sergentu, C. J. Stein, L. Ungur, M. Vacher, A. Valentini and V. Veryazov, *J. Chem. Phys.*, 2020, **152**, 214117.

60 B. O. Roos, R. Lindh, P. Å. Malmqvist, V. Veryazov and P.-O. Widmark, *J. Phys. Chem. A*, 2004, **105**, 2851–2858.

61 B. O. Roos, R. Lindh, P. Å. Malmqvist, V. Veryazov and P.-O. Widmark, *J. Phys. Chem. A*, 2005, **109**, 6575–6579.

62 *Jellby*, Pegamoid, 2018. Retrieved from https://gitlab.com/Jellby/Pegamoid.

63 L. Ungur and L. F. Chibotaru, *Chem. Eur. J.*, 2017, **23**, 3708–3718.

63 H. Putz and K. Brandenburg, *Diamond - Crystal and Molecular Structure Visualization*, v.4.6.8., Crystal Impact, 2018. Retrieved from https://www.crystalimpact.de/diamond.
